# Kharasch-Type Haloalkylation of Alkenes by Photoinduced Copper Catalysis

**DOI:** 10.1021/jacs.5c05699

**Published:** 2025-05-20

**Authors:** Yuan Cai, Mahiob Dawor, Gaurav Gaurav, Tobias Ritter

**Affiliations:** † 28314 Max-Planck-Institut für Kohlenforschung, D-45470 Mülheim an der Ruhr, Germany; ‡ Institute of Organic Chemistry, RWTH Aachen University, 52074 Aachen, Germany

## Abstract

The simultaneous construction of C–C and C–X bonds in a single step facilitates multistep synthesis through rapid carbon chain growth and subsequent transformations of halide functionalities. Traditional Kharasch addition requires simple polyhalogenated compounds or those with electron-withdrawing groups at the α-carbon. Herein, we present a Kharasch-type reaction utilizing a broad range of carboxylic acid-derived redox-active esters as the alkyl source, which enables the efficient introduction of highly functionalized alkyl groups. Our method produces α-halo carbonyls that enable versatile nucleophilic substitutions for synthesizing valuable compounds, such as unnatural α-amino acids.

## Graphical abstract

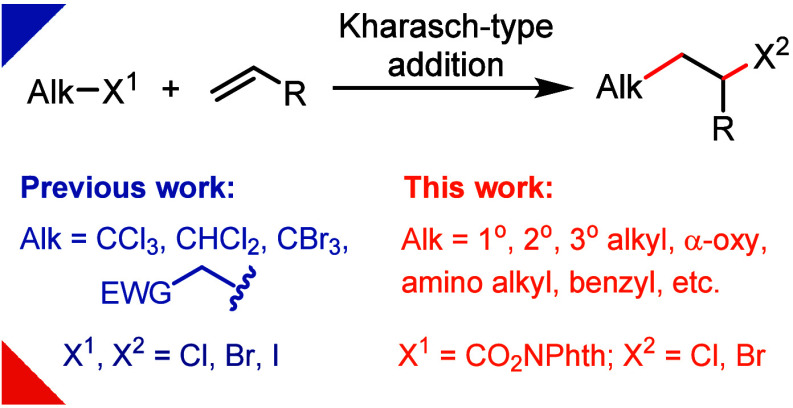

The Kharasch addition was pioneered nearly 80 years ago and provides a strategy for the simultaneous formation of two new sigma bonds, one C­(sp^3^)–C­(sp^3^) and one C­(sp^3^)–X bond.^1^ The resulting alkyl halide can readily undergo substitution-, elimination-, and reduction reactions, or function as a precursor for further radical reactions. Nevertheless, the Kharasch addition remains underutilized in organic synthesis owing to the typical requirement for polyhalogenated substrates such as carbontetrachloride or those with electron-withdrawing groups (EWGs) at the α-carbon, which limits its synthetic applications and general versatility.^2^ Herein, we describe a Kharasch-type haloalkylation of alkenes with aliphatic carboxylic acids as alkyl sources, readily available in large diversity, which enables a different and expanded substrate scope when compared to the traditional Kharasch addition. Our transformation differs fundamentally from, and complements, the traditional Kharasch addition by employing nucleophilic, unstabilized alkyl radicals and electron-deficient alkenes, whereas conventional Kharasch additions utilize electrophilic, stabilized alkyl radicals and electron-rich alkenes. Key to the successful development is the use of an electron-rich phosphine-ligated copper complex, which circumvents the undesired two-component direct decarboxylative halogenation, and also functions as both a photocatalyst and a group transfer catalyst. Our approach generates α-chloro- and bromo- carbonyls that can engage in versatile nucleophilic substitutions for constructing C–N, C–O, and C–S bonds that cannot be accessed by traditional Kharasch additions or the Giese reactions. Both these traditional reactions typically yield unactivated secondary alkyl halides or hydroalkylation products lacking halide functionality, both of which are less suitable for further synthetically valuable transformations.

The 1,2-difunctionalization of alkenes is a key approach in synthetic chemistry, which has inspired solutions from traditional halohydrin formation to modern transition metal^3^ and photoredox catalysis.^4^ 1,2-Carbohalogenation of alkenes through a radical pathway^5^ can be advantageous compared to transition metal-catalyzed organometallic pathways^6^ due to the absence of an alkyl-metal intermediate, which might otherwise favor β-H elimination and lead to the formation of Heck coupling byproducts. Since Kharasch’s initial report in 1945 on the initiator-promoted radical-chain addition of alkyl halides to alkenes,^1^ now known as atom transfer radical addition/cyclization (ATRA/ATRC),^2^ advances leveraging photocatalysis^7^ and transition metal catalysis^8^ have significantly broadened the substrate scope and synthetic applications. However, substrates for Kharasch addition must be selected to ensure that if radical addition to an alkene occurs, the resulting new radical is less stabilized than the initial radical to undergo an essentially irreversible halogen atom transfer (XAT) reaction from substrate or metal halide to form product (Figure 1a). As a consequence, substrates for Kharasch addition are typically limited to polyhalogenated compounds or α-halo carbonyls, such as diethyl bromomalonate and 2-bromoacetophenone.^2,7,8^ These substrates have a less negative reduction potential and weak C–X bonds, which facilitate both the formation of stabilized radical **A** and the XAT process from unstabilized radical **B** (Figure 1). Unactivated alkyl halides, which lack EWGs at the α-carbon and can introduce more diverse, functionalized alkyl groups, are generally out of scope for Kharasch addition due to their highly negative reduction potential, strong C–X bonds, and propensity to generate undesired oligomeric byproducts.^9^ To the best of our knowledge, the only example of a Kharasch addition to alkenes using an unactivated alkyl bromide was reported by Mitani in 1983, catalyzed by a ^*n*^Bu_3_P-copper complex under harsh UV irradiation with five simple examples, as shown in Figure 1b.^10^ Organotin, organosilane, and α-amino radicals can activate inert alkyl halides to form unstabilized alkyl radicals,^11^ yet, such reactions are typically constrained to the reductive hydroalkylation of alkenes, known as the Giese reaction (Figure 1c).^12^ Despite being discovered approximately 40 years after the Kharasch addition, the Giese reaction is more broadly utilized, which is likely attributed to its compatibility with a wider array of functionalized alkyl groups. However, the Giese reaction forfeits the advantage of difunctionalization, which is the benefit of the Kharasch addition.^13^ Here, we overcome the limitations of the traditional Kharasch addition and Giese reaction, and develop a versatile method for introducing a broader range of alkyl groups to olefins, yet, keeping the original ability of diflunctionalization of the Kharasch addition. Such a transformation is, to the best of our knowledge, hitherto unknown.

**Figure 1:**
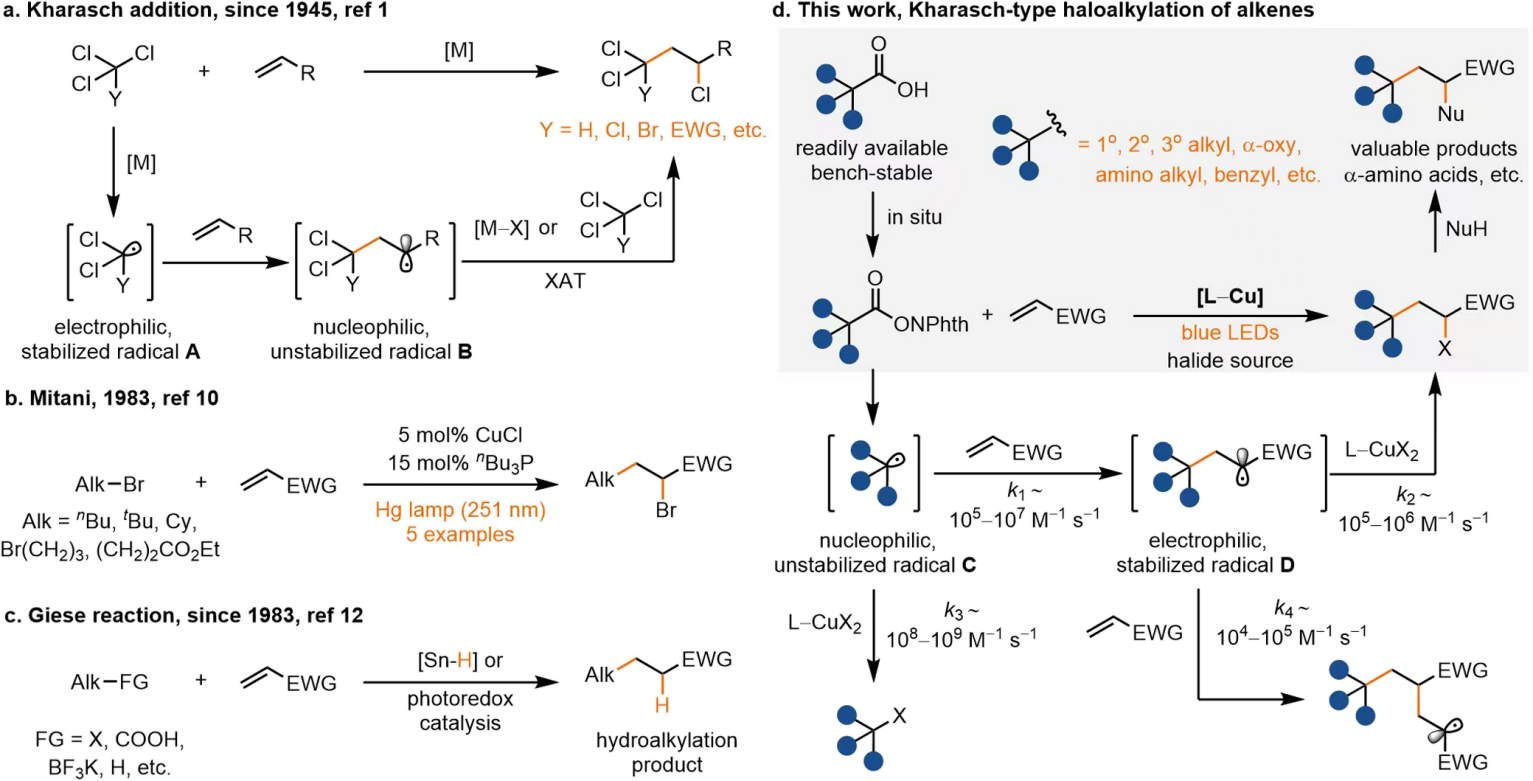
Kharasch-type haloalkylation and Giese-type hydroalkylation of alkene. XAT, halogen atom transfer; Alk, alkyl group; EWG, electron-withdraw group; X, halides; Cy, cyclohexyl; FG, functional group; NuH, nucleophile; NPhth, phthalimidoyl. *k*_2_ was taken from ref 21; *k*_4_ was taken from ref 18.

Alkyl carboxylic acid-, boron-, nitrogen-, oxygen-, silicon-, and sulfur-based modern precursors for generating unstabilized alkyl radicals via single electron transfer have emerged as valuable alternatives to traditional tin-based alkyl halides,^14^ in what has been described as a ″flight from the tyranny of tin.″^15^ Significant progress has been made with these modern unstabilized alkyl radical precursors to introduce a broad range of alkyl groups,^14^ yet, radical Karrasch-type haloalkylation of alkenes with these precursors remains unrealized. Compared to unactivated alkyl halides, these precursors offer advantages in Kharasch reactions owing to their less negative reduction potential, which not only facilitates the generation of unstabilized alkyl radicals but also helps prevent competitive oligomerization of the haloalkylation product. Despite these advantages, a fundamental challenge in applying these radical precursors to Kharasch additions is the high probability of competing direct halogenation between the alkyl radical precursor and the halide,^16^ a challenge that does not occur in traditional two-component Kharasch additions. For example, copper also induces the Kharasch addition (Figure 1d), but the rate of halogen atom transfer from L-CuX_2_ to unstabilized alkyl radical **C** is often faster (*k*_3_ ∼ 10^8^–10^9^ M^–1^ s^–1^)^17^ than the rate of addition of the radical to Michael acceptors (*k*_1_ ∼ 10^5^–10^7^ M^–1^ s^–1^)^18^ and α-olefins (10^4^–10^5^ M^–1^ s^–1^).^19^ We propose that a copper complex with an strong electron-donating ligand could be key to solving the problem and favoring the sequential C–C and C–X bond formation involved in radical Kharasch additions for the following reasons: An electron-rich copper complex would decrease the undesired XAT rate *k*_3_ between the L-CuX_2_ and the nucleophilic alkyl radical **C**, considering the polarity mismatch,^20^ while, in turn, it would favor the nucleophilic radical conjugation addition to the electron-deficient alkene (refer to *k*_1_), leading to the formation of a new electrophilic alkyl radical **D**. Conversely, it would accelerate the desired XAT rate *k*_2_ between **D** and L-CuX_2_, considering the polarity match,^20^ while also disfavoring the alkene oligomerization side reaction (refer to *k*_4_). While the Meerwein 1,2-haloarylation of alkenes has been developed with various aryl radical precursors, such as aryl diazonium,^5a^ iodonium,^5b,5c^ and thianthrenium salts,^5d^ the 1,2-carbohalogenation of alkenes with unstabilized alkyl radicals remains unexplored. We present here a Kharasch-type haloalkylation of alkenes using readily available alkyl carboxylic acid-derived redox-active esters (RAEs) as radical precursor (Figure 1d).

Optimization of reaction conditions using a photocatalysis strategy in the presence of a copper complex afforded product *rac*-**2a** in up to 84% yield with RAE **1**, acrylonitrile, and silyl chloride as starting materials. The major byproduct under optimized conditions is the direct chlorination product **2**′, which is formed in about 10% yield. BINAP-type ligands perform superior to other P and N-based ligands, which supports the hypothesis of the critical role of an electron-rich copper complex (Table S1).^22^ Even more electron-donating ligands, such as (*R*)-SEGPHOS and (*R*)-DTBM-SEGPHOS, result in less byproduct **2′**, which is consistent with the hypothesis that electron-rich ligands either decrease the rate of XAT to nucleophilic alkyl radicals or increase the rate to electrophilic ones (Figure 2). While copper-phenanthroline photoredox catalysts perform well in traditional Kharasch addition reactions,^8c,8d^ they show negligible reactivity in our reaction. Kharasch-type bromoalkylation with trimethylsilyl bromide (TMSBr) was also successful although gave slightly lower yields than chloroalkylation owing to faster competitive decarboxylative bromination (Table S1).^17^ Yields between 85–93% were achieved in both chloro- and bromoalkylation when acrylonitrile was used as the limiting reagent (Table S1). Given the ready accessibility and ease of handling of silyl chlorides compared to bromides, we primarily focus on the chloroalkylation reaction. The reaction could be readily accomplished on a gram scale, in open air, or with the RAE generated in situ from carboxylic acid and *N*-hydroxyphthalimide, which demonstrates the practicality of the methodology (Table S1).

**Figure 2:**
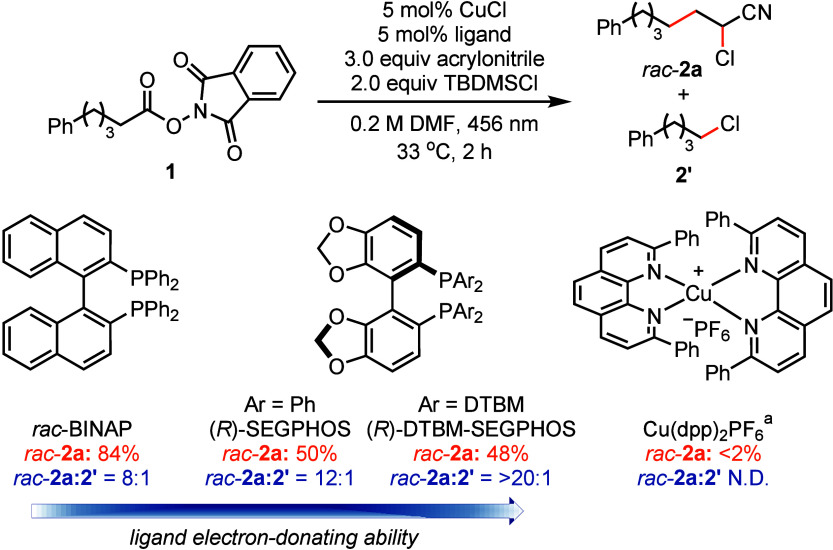
Optimized conditions and the influence of BINAP-type ligands. ^a^5 mol % Cu­(dpp)_2_PF_6_ was used without CuCl. TBDMSCl, *tert*-butyldimethylsilyl chloride; DTBM = 3,5-di*-tert*-butyl-4-methoxyphenyl; N.D. not detected.

The copper-catalyzed chloroalkylation tolerates a variety of RAEs, which can be synthesized in situ from widely available aliphatic carboxylic acids (Figure 3), including strained small ring compounds, protected natural amino acids, drug molecules, and natural products. The strained rings can be medically important cyclopropyl, cyclobutyl, and azetidinyl, as well as sp^3^-rich bioisosteres of phenyl, such as bicyclo[1.1.1]­pentane, cubane, and adamantane. The alkyl radical formed from the carboxylic acid can be unstabilized primary, secondary, tertiary, or stabilized benzylic, α-oxy, or α-amino alkyl radicals. In traditional Kharasch additions, only polyhalogenated and electron-withdrawing-group-stabilized alkyl radical precursors can be used.^2,7,8^ Numerous functional groups are tolerated, including alkyl and aryl chlorides, ethers, aldehydes, ketones, esters, amides, nitriles, boronic ester, trideuteromethyl, multifluoroalkyl, polyunsaturated double bonds, protected aliphatic amines, alcohols, indoles, and protic groups.

**Figure 3:**
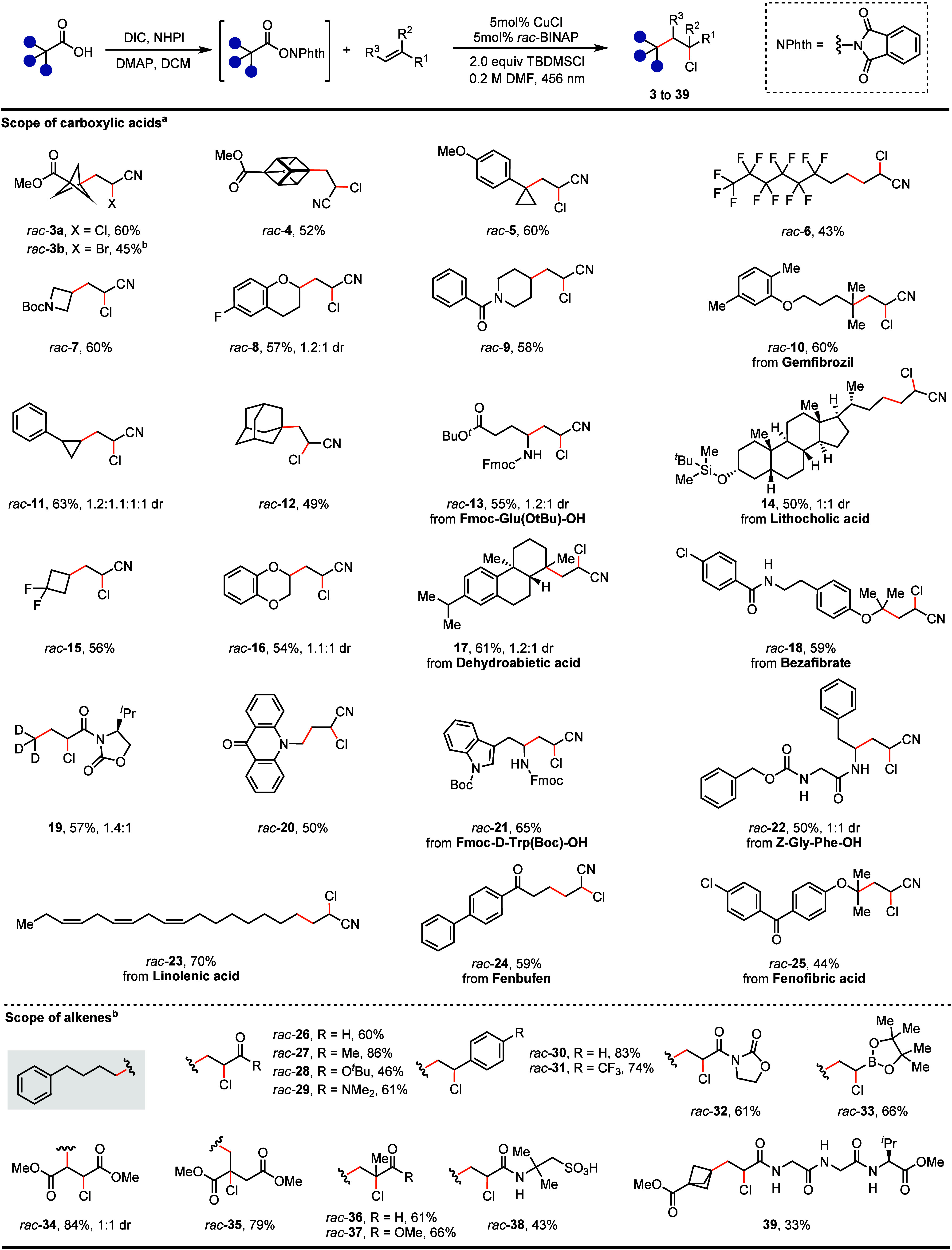
Substrate scope. ^a^1.0 equiv of RAE and 3.0 equiv of acrylonitrile were used; ^b^2.0 equiv of RAE and 1.0 equiv of alkene were used. Fmoc, fluorenylmethyloxycarbonyl; Boc, *tert*-butyloxycarbonyl; DIC, *N,N*′-diisopropylcarbodiimide; DMAP, 4-dimethylaminopyridine; NHPI, *N*-hydroxyphthalimide.

Traditional Kharasch additions generally employ electron-rich alkenes because of the electrophilic radicals involved.^2,7,8^ While electron-deficient alkenes can be used in some cases, they typically result in low yields as a result of oligomerization.^2,7,8^ In our study, a variety of widely available electron-deficient alkenes were tolerated, such as acrylonitrile, acrolein, enones, acrylates, acrylamides, and styrenes (Figure 3). The alkenes can be mono-, 1,1-di-, or 1,2-disubstituted, producing more complex alkyl halides, which would otherwise require multiple steps to synthesize. Alkenes with lower electrophilicity than acrylonitrile, such as acrylamide and styrene,^23^ produced moderate yields due to the formation of significant amounts of decarboxylative halogenation byproducts; however, yields improved substantially when the alkenes, rather than the RAE, were used as the limiting reagents. A tripeptide-derived acrylamide was also compatible and provided a α-chloro amide **39** that could potentially be used for tetrapeptide synthesis. Electron-rich alkenes are less effective on account of the slow addition rate between the nucleophilic alkyl radical and the electron-rich double bonds.^19^ The low diastereoselectivity observed for product **41** may be attributed to reversible halogen atom transfer between the stabilized alkyl radical and the Cu­(II)­Cl_2_ species under light.^24^

With electron-deficient alkenes as radical acceptors, our strategy produces α-halo carbonyls that are suitable for further transformations, whereas traditional Kharasch addition typically employs electron-rich alkenes and yields unactivated secondary alkyl halides that are generally not sufficiently reactive for subsequent substitution.^25^ As shown in Scheme 1, using acrylamide with an Evans auxiliary as the substrate, the resulting α-chloro amide enabled successful substitution reactions with nitrogen-, oxygen-, and sulfur-based nucleophiles, yielding valuable precursors to α-amino-, oxy-, and thio carboxylic acids. Notably, a high diastereoselective nucleophilic substitution via dynamic kinetic resolution^26^ was achieved using morpholine as the nucleophile, potentially enabling chiral amino acid synthesis upon subsequent hydrolysis of the amide bond. For substitution products with low diastereoselectivity, such as **44**, the two diastereomers can be separated through column chromatography (page S47).

**Scheme 1:**
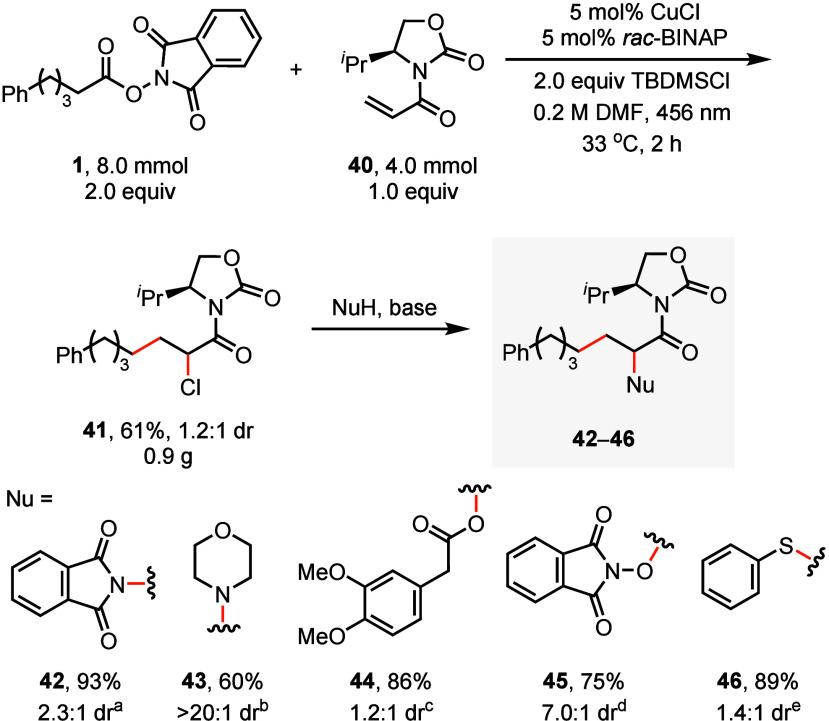
Gram-scale Kharasch-type chloroalkylation of acrylamide with an Evans auxiliary and transformations of the alkyl halide product^a^

a Potassium phthalimide, TBAI, MeCN, 80 °C, 2 h. ^*b*^Morpholine, DIPEA, TBAI, MeCN, 80 °C, 60 h. ^*c*^3,4-Dimethoxyphenylacetic acid, K_2_CO_3_, TBAI, DMF, rt, 24 h. ^*d*^NHPI, DIPEA, TBAI, MeCN, 80 °C, 2 h. ^*e*^Thiophenol, DIPEA, TBAI, MeCN, 80 °C, 2 h.

Preliminary mechanistic studies suggest that the *rac*-BINAP-CuCl complex functions as a photocatalyst, as evidenced by UV–vis spectroscopy (Figure S2) and Stern–Volmer quenching experiments (Figure 4a). The quenching experiments also reveal that TBDMSCl, a Lewis-acidic silyl reagent, activates RAE **1**, possibly by interacting with its Lewis-basic carbonyl group, resulting in a more than 500-fold increase in the quenching constant (K_sv_) (Figure 4a). This observation aligns with Reisman’s findings on silyl-accelerated, nickel-catalyzed reductive coupling by lowering the reduction potential of RAE.^27^ Based on previous work in photoinduced copper catalysis^28^ and copper-catalyzed XAT reactions,^17,29^ we propose an operative mechanism outlined in Figure 4b. Upon excitation of *rac*-BINAP-CuCl with blue light, the excited copper complex reduces silyl-activated RAE and generates a nucleophilic alkyl radical while producing silyl phthalimide and releasing CO_2_. The nucleophilic radical then adds to the electron-deficient alkene to form an electrophilic radical. The electrophilic radical competes with the initial nucleophilic radical for a chlorine atom from *rac*-BINAP-CuCl_2_,^24^ resulting in the formation of product. The direct chlorination product, such as **2′**, is not a competent intermediate for photoreduction under optimized conditions, suggesting that further reduction of the alkyl chloride by the copper photocatalyst is unlikely despite the high reduction potential of the excited BINAP–Cu­(I) complex.^22,24^

**Figure 4:**
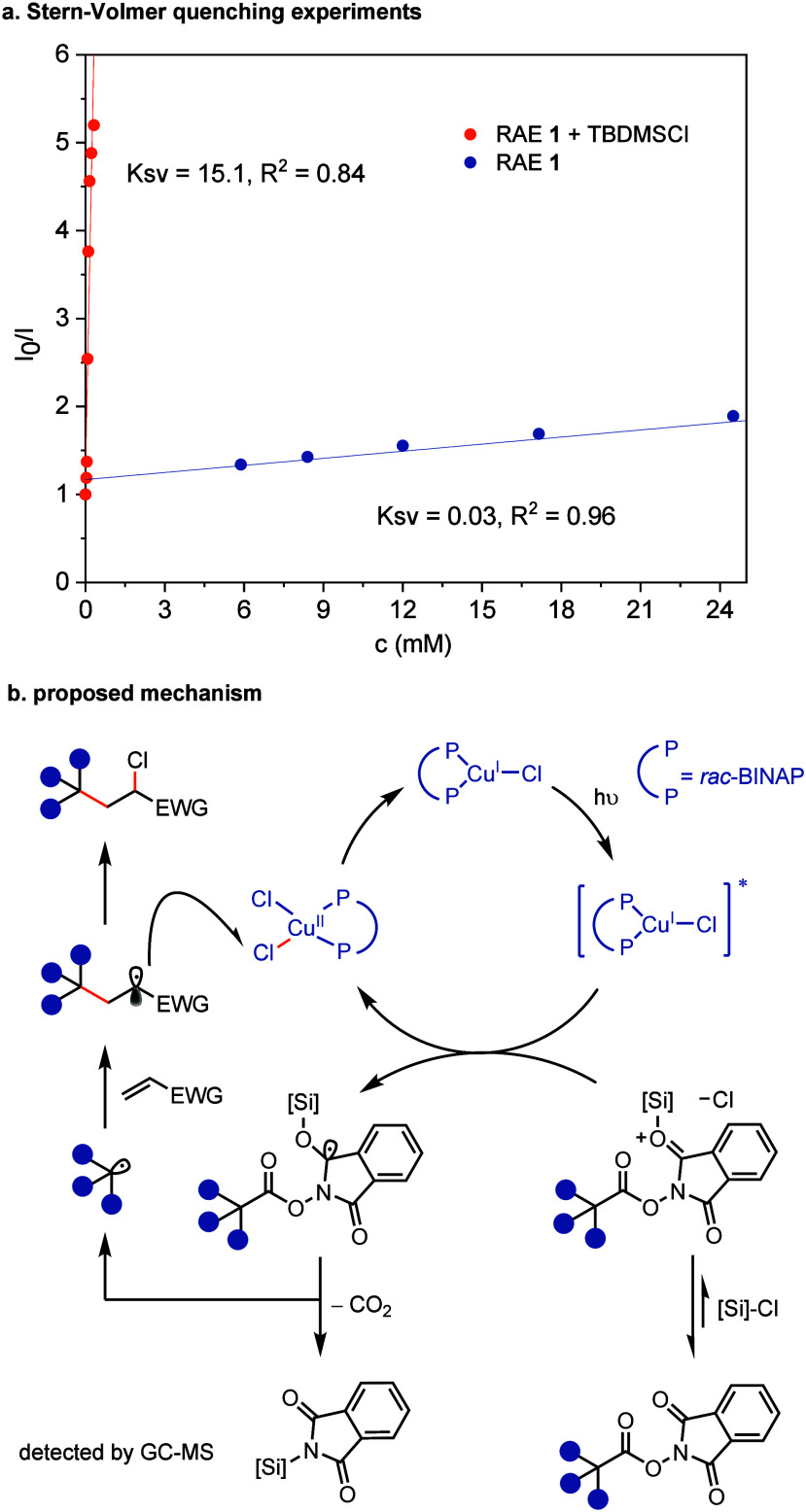
Stern–Volmer quenching experiments and proposed mechanism.

In conclusion, we have successfully achieved a Kharasch-type haloalkylation of electron-deficient alkenes and styrenes with unstabilized, nucleophilic alkyl radicals generated from carboxylic acid-derived redox-active esters. This approach contrasts with the traditional Kharasch addition, which employs electron-rich alkenes and stabilized, electrophilic alkyl radicals derived from polyhalogenated compounds or those with electron-withdrawing groups at the α-carbon. Our method enables the introduction of a wide range of highly functionalized alkyl groups that were previously unattainable with traditional Kharasch addition. Application of this approach in complex synthesis is anticipated, given the practical, robust reaction conditions and the feasibility of subsequent transformations.

## Supplementary Material



## Notes

Experimental procedures, spectroscopic data, and NMR spectra of all products (PDF)
