# Supplementary material for: Kharasch-Type Haloalkylation of Alkenes by Photoinduced Copper Catalysis
Source: J Am Chem Soc. 2025 May 20;147(22):18438–44. doi: 10.1021/jacs.5c05699 (PMC12147127; doi:10.1021/jacs.5c05699)
Supplement: Supplementary file 1 [file ja5c05699_si_001.pdf]

SUPPORTING INFORMATION

Kharasch-type haloalkylation of alkenes by photoinduced copper catalysis

Yuan Cai†, Mahiob Dawor†, Gaurav Gaurav†, ‡, and Tobias Ritter*,†

†Max-Planck-Institut für Kohlenforschung, D-45470 Mülheim an der Ruhr, Germany.

‡Institute of Organic Chemistry, RWTH Aachen University, 52074 Aachen, Germany.

*E-mail: ritter@kofo.mpg.de

TABLE OF CONTENTS

TABLE OF CONTENTS 1

MATERIALS AND METHODS 6

EXPERIMENTAL DATA 7

General procedure of Kharasch-type chloroalkylation of alkenes 7

Substrate scope of carboxylic acids 7

rac-α-Chloronitrile **2a** 8

rac-α-Bromonitrile **2b** 8

rac-Bicyclopentane-derived α-chloronitrile **3a** 9

rac-Bicyclopentane-derived α-bromonitrile **3b** 10

rac-Cubane-derived α-chloronitrile **4** 11

rac-Cyclopropane-derived α-chloronitrile **5** 11

rac-Multifluoroalkyl-derived α-chloronitrile **6** 12

rac-Azetidine-derived α-chloronitrile **7** 13

rac-Fluorochromane-derived α-chloronitrile **8** 14

rac-Benzoylpiperidine-derived α-chloronitrile **9** 15

rac-Gemfibrozil-derived α-chloronitrile **10** 16

rac-Cyclopropane-derived α-chloronitrile **11** 17

rac-Adamantane-derived α-chloronitrile **12** 18

rac-Glutamic acid-derived α-chloronitrile **13** 18

Lithocholic-derived α-chloronitrile **14** 20

rac-Difluorocyclobutane-derived α-chloronitrile **15** 21

rac-Dihydrobenzodioxine-derived α-chloronitrile **16** 22

Dehydroabietic-derived α-chloronitrile **17** 23

rac-Bezafibrate-derived α-chloronitrile **18** 24

(Methyl-d_3)-derived α-chloro amide **19** 25

rac-Oxoacridine-derived α-chloronitrile **20** 26

rac-Tryptophan-derived α-chloronitrile **21** 27

rac-Dipeptide-derived α-chloronitrile **22** 28

rac-Linolenic-derived α-chloronitrile **23** 29

rac-Fenbufen-derived α-chloronitrile **24** 30

rac-Fenofibric acid-derived α-chloronitrile **25** 31

General procedure 32

Alkene Scope 32

rac-2-Chloro-7-phenylheptanal **26** 32

rac-4-Chloro-8-phenyloctan-2-one **27** 33

rac-tert-Butyl 2-chloro-7-phenylheptanoate **28** 34
rac-2-Chloro-*N,N*-dimethyl-7-phenylheptanamide **29** 34
rac-(1-Chlorohexane-1,6-diyl)dibenzene **30** 35
rac-1-(1-Chloro-6-phenylhexyl)-4-(trifluoromethyl)benzene **31** 36
rac-3-(2-Chloro-7-phenylheptanoyl)oxazolidin-2-one **32** 37
rac-2-(1-Chloro-6-phenylhexyl)-4,4,5,5-tetramethyl-1,3,2-dioxaborolane **33** 37
rac-Dimethyl 2-chloro-3-(4-phenylbutyl)succinate **34** 38
rac-Dimethyl 2-chloro-2-(5-phenylpentyl)succinate **35** 39
rac-2-Chloro-2-methyl-7-phenylheptanal **36** 40
rac-Methyl 2-chloro-2-methyl-7-phenylheptanoate **37** 40
rac-2-(2-Chloro-7-phenylheptanamido)-2-methylpropane-1-sulfonic acid **38** 41
Bicyclopentane-derived tripeptide α-chloronitrile **39** 42

Reaction condition optimization 44

General procedure for condition optimization 44

Gram-scale synthesis 45

Evans auxiliary-derived α-chloro amide **41** 45

Transformations of the alkyl halide product 46

5-Phenylpentanoyl-derived α- amide **42** 46
5-Phenylpentanoyl-derived α-morpholine amide **43** 47
5-Phenylpentanoyl-derived α-phenylester amide **44** 47
5-Phenylpentanoyl-derived α-oxophthalimid **45** 48
5-Phenylpentanoyl-derived α-phenylthio amide **46** 49

Mechanistic study 50

UV-vis absorption spectroscopy studies 50
Cyclic voltammograms 53
Stern-volmer luminescence quenching studies 55

SPECTROSCOPIC DATA 58

^{1}H NMR of *rac*-α-chloronitrile **2a** 58
^{13}C NMR of *rac*-α-chloronitrile **2a** 59
^{1}H NMR of *rac*-α-bromonitrile **2b** 60
^{13}C NMR of *rac*-α-bromonitrile **2b** 61
^{1}H NMR of *rac*-bicyclopentane-derived α-chloronitrile **3a** 62
^{13}C NMR of *rac*-bicyclopentane-derived α-chloronitrile **3a** 63
^{1}H NMR of *rac*-bicyclopentane-derived α-bromonitrile **3b** 64
^{13}C NMR of *rac*-bicyclopentane-derived α-bromonitrile **3b** 65

^{1}H NMR of *rac*-cubane-derived α-chloronitrile **4** 66
^{13}C NMR of *rac*-cubane-derived α-chloronitrile **4** 67
^{1}H NMR of *rac*-cyclopropane-derived α-chloronitrile **5** 68
^{13}C NMR of *rac*-cyclopropane-derived α-chloronitrile **5** 69
^{1}H NMR of *rac*-multifluoroalkyl-derived α-chloronitrile **6** 70
^{19}F NMR of *rac*-multifluoroalkyl-derived α-chloronitrile **6** 71
$^{13}C\{^{19}F\}$ NMR of *rac*-multifluoroalkyl-derived α-chloronitrile **6** 72
^{1}H NMR of *rac*-azetidine-derived α-chloronitrile **7** 73
^{13}C NMR of *rac*-azetidine-derived α-chloronitrile **7** 74
^{1}H NMR of *rac*-fluorochromane-derived α-chloronitrile **8** 75
^{19}F NMR of *rac*-fluorochromane-derived α-chloronitrile **8** 76
^{13}C NMR of *rac*-fluorochromane-derived α-chloronitrile **8** 77
^{1}H NMR of *rac*-benzoylpiperidine-derived α-chloronitrile **9** 78
^{13}C NMR of *rac*-benzoylpiperidine-derived α-chloronitrile **9** 79
^{1}H NMR of *rac*-gemfibrozil-derived α-chloronitrile **10** 80
^{13}C NMR of *rac*-gemfibrozil-derived α-chloronitrile **10** 81
^{1}H NMR of *rac*-cyclopropane-derived α-chloronitrile **11** 82
^{13}C NMR of *rac*-cyclopropane-derived α-chloronitrile **11** 83
^{1}H NMR of *rac*-adamantane-derived α-chloronitrile **12** 84
^{13}C NMR of *rac*-adamantane-derived α-chloronitrile **12** 85
^{1}H NMR of *rac*-glutamic acid-derived α-chloronitrile **13** 86
^{13}C NMR of *rac*-glutamic acid-derived α-chloronitrile **13** 87
^{1}H NMR of lithocholic-derived α-chloronitrile **14** 88
^{13}C NMR of lithocholic-derived α-chloronitrile **14** 89
^{1}H NMR of *rac*-difluorocyclobutane-derived α-chloronitrile **15** 90
^{19}F NMR of *rac*-difluorocyclobutane-derived α-chloronitrile **15** 91
^{13}C NMR of *rac*-difluorocyclobutane-derived α-chloronitrile **15** 92
^{1}H NMR of *rac*-dihydrobenzodioxine-derived α-chloronitrile **16** 93
^{13}C NMR of *rac*-dihydrobenzodioxine-derived α-chloronitrile **16** 94
^{1}H NMR of dehydroabietic-derived α-chloronitrile **17** 95
^{13}C NMR of dehydroabietic-derived α-chloronitrile **17** 96
^{1}H NMR of *rac*-bezafibrate-derived α-chloronitrile **18** 97
^{13}C NMR of *rac*-bezafibrate-derived α-chloronitrile **18** 98
^{1}H NMR of (methyl-d_3)-derived α-chloroisopropyloxazolidone **19** 99
^{13}C NMR of (methyl-d_3)-derived α-chloroisopropyloxazolidone **19** 100
^{1}H NMR of *rac*-oxoacridin-derived α-chloronitrile **20** 101
^{13}C NMR of *rac*-oxoacridin-derived α-chloronitrile **20** 102
^{1}H NMR of *rac*-tryptophan-derived α-chloronitrile **21** 103

^{13}C NMR of *rac*-tryptophan-derived α-chloronitrile **21** 104
^{1}H NMR of *rac*-dipeptide-derived α-chloronitrile **22** 105
^{13}C NMR of *rac*-dipeptide-derived α-chloronitrile **22** 106
^{1}H NMR of *rac*-linolenic-derived α-chloronitrile **23** 107
^{13}C NMR of *rac*-linolenic-derived α-chloronitrile **23** 108
^{1}H NMR of *rac*-fenbufen-derived α-chloronitrile **24** 109
^{13}C NMR of *rac*-fenbufen-derived α-chloronitrile **24** 110
^{1}H NMR of *rac*-fenofibric acid-derived α-chloronitrile **25** 111
^{13}C NMR of *rac*-fenofibric acid-derived α-chloronitrile **25** 112
^{1}H NMR of *rac*-2-chloro-7-phenylheptanal **26** 113
^{13}C NMR of *rac*-2-chloro-7-phenylheptanal **26** 114
^{1}H NMR of *rac*-4-chloro-8-phenyloctan-2-one **27** 115
^{13}C NMR of *rac*-4-chloro-8-phenyloctan-2-one **27** 116
^{1}H NMR of *rac*-*tert*-butyl 2-chloro-7-phenylheptanoate **28** 117
^{13}C NMR of *rac*-*tert*-butyl 2-chloro-7-phenylheptanoate **28** 118
^{1}H NMR of *rac*-2-chloro-*N,N*-dimethyl-7-phenylheptanamide **29** 119
^{13}C NMR of *rac*-2-chloro-*N,N*-dimethyl-7-phenylheptanamide **29** 120
^{1}H NMR of *rac*-(1-chlorohexane-1,6-diyl)dibenzene **30** 121
^{13}C NMR of *rac*-(1-chlorohexane-1,6-diyl)dibenzene **30** 122
^{1}H NMR of *rac*-1-(1-chloro-6-phenylhexyl)-4-(trifluoromethyl)benzene **31** 123
^{19}F NMR of *rac*-1-(1-chloro-6-phenylhexyl)-4-(trifluoromethyl)benzene **31** 124
^{13}C NMR of *rac*-1-(1-chloro-6-phenylhexyl)-4-(trifluoromethyl)benzene **31** 125
^{1}H NMR of *rac*-3-(2-chloro-7-phenylheptanoyl)oxazolidin-2-one **32** 126
^{13}C NMR of *rac*-3-(2-chloro-7-phenylheptanoyl)oxazolidin-2-one **32** 127
^{1}H NMR of *rac*-2-(1-chloro-6-phenylhexyl)-4,4,5,5-tetramethyl-1,3,2-dioxaborolane **33** 128
^{13}C NMR of *rac*-2-(1-chloro-6-phenylhexyl)-4,4,5,5-tetramethyl-1,3,2-dioxaborolane **33** 129
^{1}H NMR of *rac*-dimethyl 2-chloro-3-(4-phenylbutyl)succinate **34** 130
^{13}C NMR of *rac*-dimethyl 2-chloro-3-(4-phenylbutyl)succinate **34** 131
^{1}H NMR of *rac*-dimethyl 2-chloro-2-(5-phenylpentyl)succinate **35** 132
^{13}C NMR of *rac*-dimethyl 2-chloro-2-(5-phenylpentyl)succinate **35** 133
^{1}H NMR of *rac*-2-chloro-2-methyl-7-phenylheptanal **36** 134
^{13}C NMR of *rac*-2-chloro-2-methyl-7-phenylheptanal **36** 135
^{1}H NMR of *rac*-methyl 2-chloro-2-methyl-7-phenylheptanoate **37** 136
^{13}C NMR of *rac*-methyl 2-chloro-2-methyl-7-phenylheptanoate **37** 137
^{1}H NMR of *rac*-2-(2-chloro-7-phenylheptanamido)-2-methylpropane-1-sulfonic acid **38** 138
^{13}C NMR of *rac*-2-(2-chloro-7-phenylheptanamido)-2-methylpropane-1-sulfonic acid **38** 139
^{1}H NMR of bicyclopentane-derived tripeptide α-chloronitrile **39** 140
^{13}C NMR of bicyclopentane-derived tripeptide α-chloronitrile **39** 141

^{1}H NMR of 5-phenylpentanoyl-derived α-chloro amide **41** 142
^{13}C NMR of 5-phenylpentanoyl-derived α-chloro amide **41** 143
^{1}H NMR of 5-phenylpentanoyl-derived α-amide **42** 144
^{13}C NMR of 5-phenylpentanoyl-derived α-amide **42** 145
^{1}H NMR of 5-phenylpentanoyl-derived α-morpholine amide **43** 146
^{13}C NMR of 5-phenylpentanoyl-derived α-morpholine amide **43** 147
^{1}H NMR of 5-phenylpentanoyl-derived α-phenylester amide **44** 148
^{13}C NMR of 5-phenylpentanoyl-derived α-phenylester amide **44** 149
^{1}H NMR of 5-phenylpentanoyl-derived α-phenylester amide **44’** 150
^{13}C NMR of 5-phenylpentanoyl-derived α-phenylester amide **44’** 151
^{1}H NMR of 5-phenylpentanoyl-derived α-oxo amide **45** 152
^{13}C NMR of 5-phenylpentanoyl-derived α-oxo amide **45** 153
^{1}H NMR of 5-phenylpentanoyl-derived α- phenylthio amide **46** 154
^{13}C NMR of 5-phenylpentanoyl-derived α- phenylthio amide **46** 155

REFERENCES 156

MATERIALS AND METHODS

All air- and moisture-insensitive reactions were carried out under ambient atmosphere and monitored by thin-layer chromatography (TLC). Concentration under reduced pressure was performed by rotary evaporation at 25–40 °C at an appropriate pressure. Purified compounds were further dried under high vacuum (0.010–0.005 mbar). Yields refer to purified and spectroscopically pure compounds. All air- and moisture-sensitive manipulations were performed using oven-dried glassware (120 °C for a minimum of 12 hours) and standard Schlenk techniques under an atmosphere of argon.

Solvents

DMF was purchased from *Sigma-Aldrich*. Acetonitrile was purchased from *Fisher Scientific*. Anhydrous solvents were obtained from Phoenix Solvent Drying Systems. All deuterated solvents were purchased from Euriso-Top.

Chromatography

Thin layer chromatography (TLC) was performed using EMD TLC plates pre-coated with 250 µm thickness silica gel 60 F254 plates and visualized by fluorescence quenching under 254 nm UV light, permanganate stain, cerium ammonium molybdate stain, or phosphomolybdic acid stain. Flash chromatography was performed using silica gel (40–63 µm particle size) purchased from Geduran®

Spectroscopy and Instruments

NMR spectra were recorded on a Bruker Ascend TM 500 spectrometer operating at 500 MHz, 471 MHz and 126 MHz, for ^{1}H, ^{19}F, and ^{13}C acquisitions, respectively. Chemical shifts are reported in ppm with the solvent residual peak as the internal standard. For ^{1}H NMR: $CDCl_3$, 7.26; CD_3CN, 1.96; CD_2Cl_2, 5.32. For ^{13}C NMR: $CDCl_3$, 77.16; CD_3CN, 1.32; CD_2Cl_2, 53.84.1 Data is reported as follows: s = singlet, d = doublet, t = triplet, q = quartet, quin = quintet, sext = sextet, sept = septet, m = multiplet, bs = broad singlet; coupling constants in Hz; integration.

Starting materials

All substrates were used as received from commercial suppliers or prepared according to published procedures, unless otherwise stated. Chemicals were purchased from *Sigma-Aldrich*, *TCI*, or *Alfa Aesar*, *BLD pharma* and *Apollo scientific*. Acryloylglycylglycine **S47** [1], 3-acryloyl-4-isopropyloxazolidin-2-one (**40**) [2], *rac*-BINAPCuCl [3], redox-active ester **1** [4], redox-active ester **48** [5] were prepared according to the literature.

EXPERIMENTAL DATA

General procedure of Kharasch-type chloroalkylation of alkenes

Substrate scope of carboxylic acids

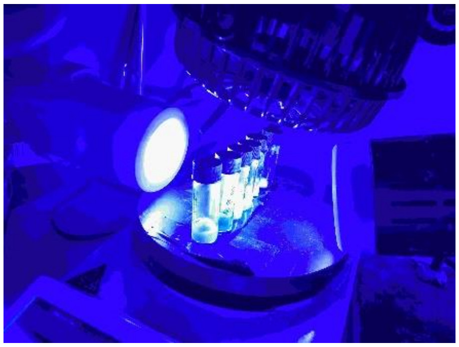

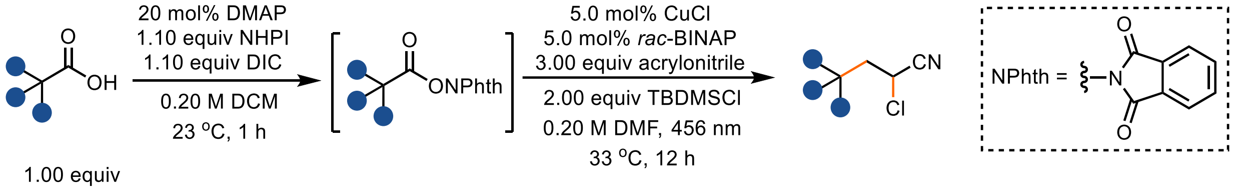

Under an ambient atmosphere, to a 4-mL borosilicate vial equipped with a Teflon-coated magnetic stir bar were added carboxylic acid (0.200 mmol, 1.00 equiv), *N*-hydroxyphthalimide (NHPI) (35.8 mg, 0.220 mmol, 1.10 equiv), 4-dimethylamino-pyridine (DMAP) (4.9 mg, 40 μmol, 20 mol%), *N*,*N*'-diisopropylcarbodiimide (DIC) (27.7 mg, 0.220 mmol, 1.10 equiv), and dichloromethane (DCM) (1.0 mL, c = 0.20 M). After stirring the mixture at 23 °C for 1 h, the solvent was removed under reduced pressure. Without purification of the crude redox active ester, copper chloride (CuCl) (1.0 mg, 10 μmol, 5.0 mol%), *rac*-2,2'-bis-(diphenylphosphino)-1,1'-binaphthyl (*rac*-BINAP) (6.2 mg, 10 μmol, 5.0 mol%), and *tert*-butyldimethylchlorosilane (TBDMSCl) (60.3 mg, 0.400 mmol, 2.00 equiv) were dissolved in *N*,*N*-dimethylformamide (DMF) (1.0 mL, c = 0.20 M) and then acrylonitrile (40.2 μL , 31.9 mg, 0.600 mmol, 3.00 equiv) was added. Roughly 100 mL of argon was bubbled through the reaction mixture using a Schlenk line for 30 seconds. The reaction mixture was irradiated with blue LEDs (456 nm, 2 × 40 W) for 12 h at 33 °C. Following irradiation, ethyl acetate (30 mL) and brine (30 mL) were added. The mixture was extracted once with ethyl acetate (30 mL), and the organic layer was washed with brine (30 mL), dried over Na_2SO_4 and concentrated under reduced pressure. The diastereomeric ratio of the crude product was determined using NMR or HPLC spectroscopy, where applicable. The resulting residue was purified by flash column chromatography on silica gel to afford the desired product.

rac-α-Chloronitrile 2a

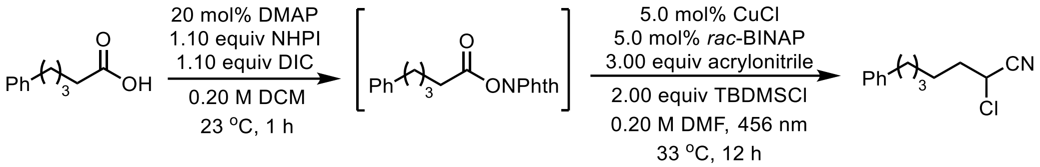

S1 **1** *rac*-**2a**, 75%

Under an ambient atmosphere, to a 4-mL borosilicate vial equipped with a Teflon-coated magnetic stir bar were added 5-phenylpentanoic acid **S1** (35.6 mg, 0.20 mmol, 1.00 equiv), *N*-hydroxyphthalimide (NHPI) (35.8 mg, 0.220 mmol, 1.10 equiv), 4-dimethylamino-pyridine (DMAP) (4.90 mg, 40 µmol, 20 mol%), *N*,*N*'-diisopropylcarbodiimide (DIC) (27.7 mg, 0.220 mmol, 1.10 equiv), and dichloromethane (DCM) (1.0 mL, c = 0.20 M). After stirring the mixture at 23 °C for 1 h, the solvent was removed under reduced pressure. Without purification of the crude redox active ester, copper chloride (CuCl) (1.0 mg, 10 µmol, 5.0 mol%), *rac*-2,2'-bis-(diphenylphosphino)-1,1'-binaphthyl (*rac*-BINAP) (6.2 mg, 10 µmol, 5.0 mol%), and *tert*-butyldimethylchlorosilane (TBDMSCl) (60.3 mg, 0.400 mmol, 2.00 equiv) were dissolved in *N*,*N*-dimethylformamide (DMF) (1.0 mL, c = 0.20 M) and then acrylonitrile (40.2 µL, 31.9 mg, 0.600 mmol, 3.00 equiv) was added. Roughly 100 mL of argon was bubbled through the reaction mixture using a Schlenk line for 30 seconds. The reaction mixture was irradiated with blue LEDs (456 nm, 2 × 40 W) for 12 h at 33 °C. Following irradiation, ethyl acetate (30 mL) and brine (30 mL) were added. The mixture was extracted once with ethyl acetate (30 mL), and the organic layer was washed with brine (30 mL), dried over Na_2SO_4 and concentrated under reduced pressure. The resulting residue was purified by flash column chromatography on silica gel (hexanes/EtOAc 10:1) to afford *rac*-**2a** as colorless solid (40.8 mg, 75%).

R*f* = 0.50 (hexanes/EtOAc 10:1).

NMR Spectroscopy:

^{1}H NMR (500 MHz, $CDCl_3$, 23 °C, δ): 7.35 – 7.25 (m, 2H), 7.23 – 7.15 (m, 3H), 4.42 (t, *J* = 6.8 Hz, 1H), 2.64 (t, *J* = 7.7 Hz, 2H), 2.11 – 1.99 (m, 2H), 1.73 – 1.54 (m, 4H), 1.47 – 1.35 (m, 2H).

^{13}C NMR (125 MHz, $CDCl_3$, 23 °C, δ): 142.2, 128.5, 128.5, 125.9, 117.2, 42.5, 36.2, 35.7, 31.0, 28.1, 25.6.

HRMS-EI (m/z) calc'd for $C_{13}H_{16}ClN$ $[M]^+$, 221.0965; found, 221.0966; deviation: +0.3 ppm.

rac-α-Bromonitrile 2b

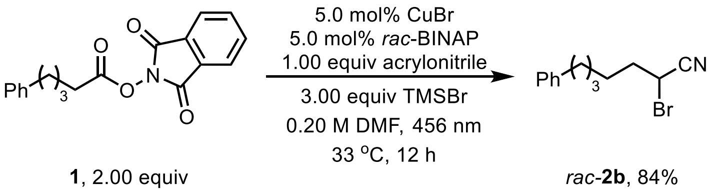

1, 2.00 equiv *rac*-**2b**, 84%

Under an ambient atmosphere, to a 4-mL borosilicate vial equipped with a Teflon-coated magnetic stir bar were added 1,3-dioxoisoindolin-2-yl 5-phenylpentanoate **1** (129 mg, 0.400 mmol, 2.00 equiv), copper bromine

(CuBr) (1.4 mg, 10 µmol, 5.0 mol%), *rac*-2,2'-bis-(diphenylphosphino)-1,1'-binaphthyl (*rac*-BINAP) (6.2 mg, 10 µmol, 5.0 mol%), and bromotrimethylsilane (TMSBr) (91.8 mg, 0.600 mmol, 3.00 equiv) were dissolved in *N*,*N*-dimethylformamide (DMF) (1.0 mL, c = 0.20 M) and then acrylonitrile (13.3 µL, 10.6 mg, 0.200 mmol, 1.00 equiv) was added. The vial was evacuated and the solvent was bubbled with inert gas using a Schlenk line under 0.50 mbar pressure for 30 seconds. The reaction mixture was irradiated with blue LEDs (456 nm, 2 × 40 W) at 33 °C for 2 h. Following irradiation, ethyl acetate (30 mL) and brine (30 mL) were added. The mixture was extracted once with ethyl acetate (30 mL), and the organic layer was washed with brine (30 mL), dried over Na_2SO_4 and concentrated under reduced pressure. The resulting residue was purified by flash column chromatography on silica gel (Pentane/EtOAc 10:1) to afford *rac*-**2b** as yellow oil (45.1 mg, 84%).

R*f* = 0.53 (Pentane/EtOAc 10:1).

NMR Spectroscopy:

^{1}H NMR (500 MHz, $CDCl_3$, 23 °C, δ): 7.30 (t, *J* = 7.5 Hz, 2H), 7.21 – 7.12 (m, 3H), 4.27 (t, *J* = 6.9 Hz, 1H), 2.64 (t, *J* = 7.6 Hz, 2H), 2.32 – 1.97 (m, 2H), 1.81 – 1.64 (m, 2H), 1.64 – 1.56 (m, 2H), 1.41 (q, *J* = 7.7 Hz, 2H).

^{13}C NMR (125 MHz, $CDCl_3$, 23 °C, δ): 142.2, 128.5, 128.5, 125.9, 117.5, 36.5, 35.7, 31.1, 28.1, 27.2, 26.9.

GC-CI Ammonia (m/z) calc'd for $C_{13}H_{20}BrN_2$ $[M+NH_4]^+$, 283.0806; found, 283.0804; deviation: +0.8 ppm.

rac-Bicyclopentane-derived α-chloronitrile 3a

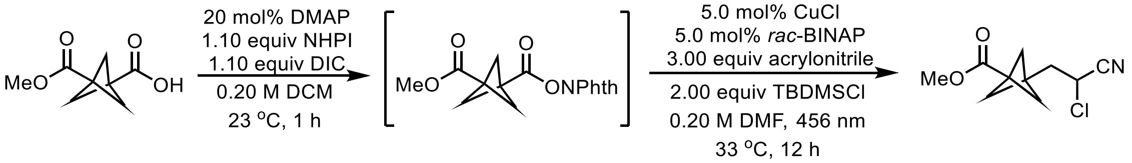

S3 *rac*-**3a**, 60%

Under an ambient atmosphere, to a 4-mL borosilicate vial equipped with a Teflon-coated magnetic stir bar were added bicyclopentane-carboxylic acid **S3** (34.0 mg, 0.20 mmol, 1.00 equiv), *N*-hydroxyphthalimide (NHPI) (35.8 mg, 0.220 mmol, 1.10 equiv), 4-dimethylamino-pyridine (DMAP) (4.90 mg, 40 µmol, 20 mol%), *N*,*N*'-diisopropylcarbodiimide (DIC) (27.7 mg, 0.220 mmol, 1.10 equiv), and dichloromethane (DCM) (1.0 mL, c = 0.20 M). After stirring the mixture at 23 °C for 1 h, the solvent was removed under reduced pressure. Without purification of the crude redox active ester, copper chloride (CuCl) (1.0 mg, 10 µmol, 5.0 mol%), *rac*-2,2'-bis-(diphenylphosphino)-1,1'-binaphthyl (*rac*-BINAP) (6.2 mg, 10 µmol, 5.0 mol%), and *tert*-butyldimethylchlorosilane (TBDMSCl) (60.3 mg, 0.400 mmol, 2.00 equiv) were dissolved in *N*,*N*-dimethylformamide (DMF) (1.0 mL, c = 0.20 M) and then acrylonitrile (40.2 µL , 31.9 mg, 0.600 mmol, 3.00 equiv) was added. Roughly 100 mL of argon was bubbled through the reaction mixture using a Schlenk line for 30 seconds. The reaction mixture was irradiated with blue LEDs (456 nm, 2 × 40 W) for 12 h at 33 °C. Following irradiation, ethyl acetate (30 mL) and brine (30 mL) were added. The mixture was extracted once with ethyl acetate (30 mL), and the organic layer was washed with brine (30 mL), dried over Na_2SO_4 and concentrated under reduced pressure. The resulting residue was purified by flash column chromatography on

silica gel (hexanes/EtOAc 6:1) to afford *rac*-**3a** as colorless oil (25.8 mg, 60%).

R_f = 0.20 (hexanes/EtOAc 6:1).

NMR Spectroscopy:

^{1}H NMR (500 MHz, $CDCl_3$, 23 °C, δ): 4.41 (t, *J* = 6.8 Hz, 1H), 3.67 (s, 3H), 2.29 (qd, *J* = 14.9, 6.8 Hz, 2H), 2.13 (s, 6H).

^{13}C NMR (125 MHz, $CDCl_3$, 23 °C, δ): 169.6, 116.9, 52.6, 51.8, 40.2, 38.5, 37.7, 36.9.

HRMS-ESIpos (m/z) calc'd for $C_{10}H_{12}ClNO_2Na$ $[M+Na]^+$, 236.0448; found, 236.0449; deviation: +0.4 ppm.

rac-Bicyclopentane-derived α-bromonitrile 3b

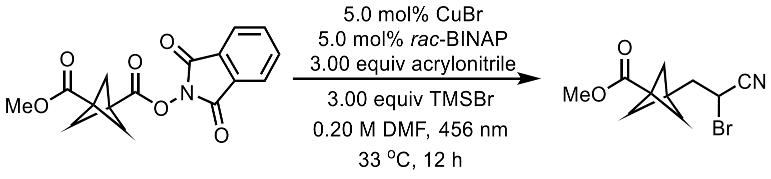

S3 *rac*-**3b**, 45%

Under an ambient atmosphere, to a 4-mL borosilicate vial equipped with a Teflon-coated magnetic stir bar were added bicyclopentane-carboxylic acid **S3** (34.0 mg, 0.20 mmol, 1.00 equiv), copper bromine (CuBr) (1.4 mg, 10 µmol, 5.0 mol%), *rac*-2,2'-bis-(diphenylphosphino)-1,1'-binaphthyl (*rac*-BINAP) (6.2 mg, 10 µmol, 5.0 mol%), and bromotrimethylsilane (TMSBr) (91.8 mg, 0.600 mmol, 3.00 equiv) were dissolved in *N*,*N*-dimethylformamide (DMF) (1.0 mL, c = 0.20 M) and then acrylonitrile (13.3 µL, 10.6 mg, 0.200 mmol, 1.00 equiv) was added. The vial was evacuated and the solvent was bubbled with inert gas using a Schlenk line under 0.50 mbar pressure for 30 seconds. The reaction mixture was irradiated with blue LEDs (456 nm, 2 × 40 W) at 33 °C for 2 h. Following irradiation, ethyl acetate (30 mL) and brine (30 mL) were added. The mixture was extracted once with ethyl acetate (30 mL), and the organic layer was washed with brine (30 mL), dried over Na_2SO_4 and concentrated under reduced pressure. The resulting residue was purified by flash column chromatography on silica gel (Pentane/EtOAc 10:1) to afford *rac*-**3b** as colorless oil (23.5 mg, 45%).

R_f = 0.28 (hexanes/EtOAc 10:1).

NMR Spectroscopy:

^{1}H NMR (500 MHz, $CDCl_3$, 23 °C, δ): 4.24 (dd, *J* = 7.7, 6.4 Hz, 1H), 3.66 (s, 3H), 2.36 (qd, *J* = 15.0, 7.0 Hz, 2H), 2.13 (s, 6H).

^{13}C NMR (125 MHz, $CDCl_3$, 23 °C, δ): 169.9, 117.6, 52.8, 52.1, 38.8, 38.29, 38.1, 23.9.

HRMS-ESIpos (m/z) calc'd for $C_{10}H_{12}BrNO_2Na$ $[M+Na]^+$, 279.9943; found, 279.9945; deviation: +0.8 ppm.

rac-Cubane-derived α-chloronitrile 4

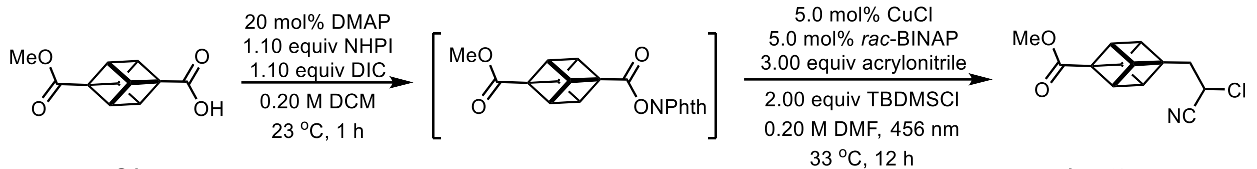

S4 *rac*-**4**, 52%

Under an ambient atmosphere, to a 4-mL borosilicate vial equipped with a Teflon-coated magnetic stir bar were added 4-(methoxycarbonyl)cubane carboxylic acid **S4** (41.2 mg, 0.20 mmol, 1.00 equiv), *N*-hydroxyphthalimide (NHPI) (35.8 mg, 0.220 mmol, 1.10 equiv), 4-dimethylamino-pyridine (DMAP) (4.90 mg, 40 µmol, 20 mol%), *N*,*N*'-diisopropylcarbodiimide (DIC) (27.7 mg, 0.220 mmol, 1.10 equiv), and dichloromethane (DCM) (1.0 mL, c = 0.20 M). After stirring the mixture at 23 °C for 1 h, the solvent was removed under reduced pressure. Without purification of the crude redox active ester, copper chloride (CuCl) (1.0 mg, 10 µmol, 5.0 mol%), *rac*-2,2'-bis-(diphenylphosphino)-1,1'-binaphthyl (*rac*-BINAP) (6.2 mg, 10 µmol, 5.0 mol%), and *tert*-butyldimethylchlorosilane (TBDMSCl) (60.3 mg, 0.400 mmol, 2.00 equiv) were dissolved in *N*,*N*-dimethylformamide (DMF) (1.0 mL, c = 0.20 M) and then acrylonitrile (40.2 µL , 31.9 mg, 0.600 mmol, 3.00 equiv) was added. Roughly 100 mL of argon was bubbled through the reaction mixture using a Schlenk line for 30 seconds. The reaction mixture was irradiated with blue LEDs (456 nm, 2 × 40 W) for 12 h at 33 °C. Following irradiation, ethyl acetate (30 mL) and brine (30 mL) were added. The mixture was extracted once with ethyl acetate (30 mL), and the organic layer was washed with brine (30 mL), dried over Na_2SO_4 and concentrated under reduced pressure. The resulting residue was purified by flash column chromatography on silica gel (Pentane/EtOAc 6:1) to afford *rac*-**4** as colorless oil (26.x mg, 52%).

$\mathbf{R}_f$ = 0.40 (Pentane/EtOAc 6:1).

NMR Spectroscopy:

^{1}H NMR (500 MHz, $CDCl_3$, 23 °C, δ): 4.49 (t, *J* = 7.2 Hz, 1H), 4.25 – 4.14 (m, 3H), 3.97 (t, *J* = 5.0 Hz, 3H), 3.70 (s, 3H), 2.58 – 2.32 (m, 2H).

^{13}C NMR (125 MHz, $CDCl_3$, 23 °C, δ): 172.4, 117.2, 56.4, 56.1, 51.6, 46.6, 46.5, 39.6, 38.7.

HRMS-ESIpos (m/z) calc'd for $C_{13}H_{12}ClNO_2Na$ $[M+Na]^+$, 272.0448; found, 272.0449; deviation: +0.3 ppm.

rac-Cyclopropane-derived α-chloronitrile 5

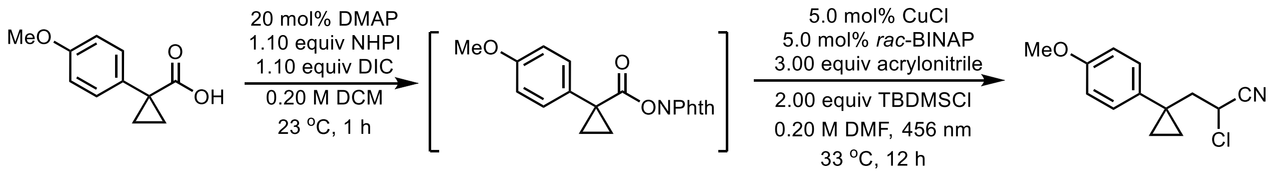

S5 *rac*-**5**, 60%

Under an ambient atmosphere, to a 4-mL borosilicate vial equipped with a Teflon-coated magnetic stir bar were added 1-(4-methoxyphenyl)cyclopropane-1-carboxylic acid **S5** (38.4 mg, 0.20 mmol, 1.00 equiv), *N*-

hydroxyphthalimide (NHPI) (35.8 mg, 0.220 mmol, 1.10 equiv), 4-dimethylamino-pyridine (DMAP) (4.90 mg, 40 µmol, 20 mol%), *N*,*N*'-diisopropylcarbodiimide (DIC) (27.7 mg, 0.220 mmol, 1.10 equiv), and dichloromethane (DCM) (1.0 mL, c = 0.20 M). After stirring the mixture at 23 °C for 1 h, the solvent was removed under reduced pressure. Without purification of the crude redox active ester, copper chloride (CuCl) (1.0 mg, 10 µmol, 5.0 mol%), *rac*-2,2'-bis-(diphenylphosphino)-1,1'-binaphthyl (*rac*-BINAP) (6.2 mg, 10 µmol, 5.0 mol%), and *tert*-butyldimethylchlorosilane (TBDMSCl) (60.3 mg, 0.400 mmol, 2.00 equiv) were dissolved in *N*,*N*-dimethylformamide (DMF) (1.0 mL, c = 0.20 M) and then acrylonitrile (40.2 µL , 31.9 mg, 0.600 mmol, 3.00 equiv) was added. Roughly 100 mL of argon was bubbled through the reaction mixture using a Schlenk line for 30 seconds. The reaction mixture was irradiated with blue LEDs (456 nm, 2 × 40 W) for 12 h at 33 °C. Following irradiation, ethyl acetate (30 mL) and brine (30 mL) were added. The mixture was extracted once with ethyl acetate (30 mL), and the organic layer was washed with brine (30 mL), dried over Na_2SO_4 and concentrated under reduced pressure. The resulting residue was purified by flash column chromatography on silica gel (hexanes/EtOAc 20:1) to afford *rac*-**5** as colorless oil (28.1 mg, 60%).

R_f = 0.30 (hexanes/EtOAc 20:1).

NMR Spectroscopy:

^{1}H NMR (500 MHz, $CDCl_3$, 23 °C, δ): 7.32 – 7.25 (m, 2H), 6.89 – 6.83 (m, 2H), 4.15 (dd, *J* = 8.8, 6.9 Hz, 1H), 3.80 (s, 3H), 2.38 (ddd, *J* = 14.0, 6.9, 1.2 Hz, 1H), 2.12 (dd, *J* = 14.1, 8.8 Hz, 1H), 1.04 – 0.92 (m, 2H), 0.90 – 0.81 (m, 2H).

^{13}C NMR (125 MHz, $CDCl_3$, 23 °C, δ): 158.9, 133.6, 117.5, 114.3, 55.4, 47.1, 40.9, 22.8, 13.0, 12.0.

HRMS-EI(m/z) calc'd for $C_{13}H_{14}ClNO$ $[M]^+$, 235.0758; found, 235.0759; deviation: +0.4 ppm.

rac-Multifluoroalkyl-derived α-chloronitrile 6

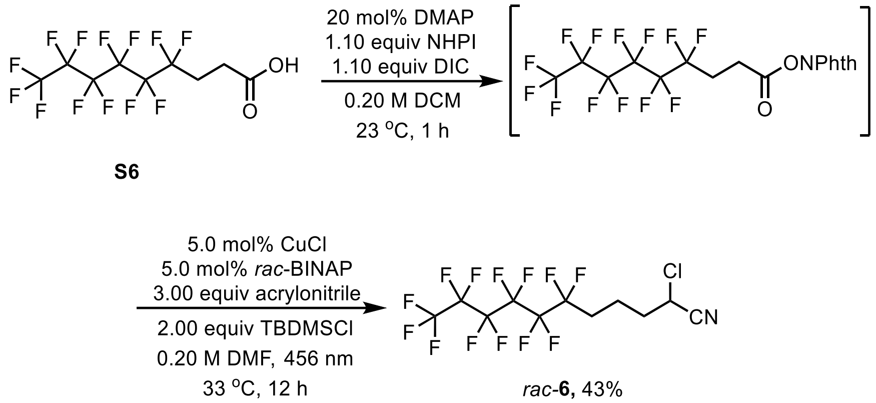

S6

rac-**6,** 43%

Under an ambient atmosphere, to a 4-mL borosilicate vial equipped with a Teflon-coated magnetic stir bar were added tridecafluorononanoic acid **S6** (78.4 mg, 0.20 mmol, 1.00 equiv), *N*-hydroxyphthalimide (NHPI) (35.8 mg, 0.220 mmol, 1.10 equiv), 4-dimethylamino-pyridine (DMAP) (4.90 mg, 40 µmol, 20 mol%), *N*,*N*'-diisopropylcarbodiimide (DIC) (27.7 mg, 0.220 mmol, 1.10 equiv), and dichloromethane (DCM) (1.0 mL, c = 0.20 M). After stirring the mixture at 23 °C for 1 h, the solvent was removed under reduced pressure. Without

purification of the crude redox active ester, copper chloride (CuCl) (1.0 mg, 10 µmol, 5.0 mol%), *rac*-2,2'-bis-(diphenylphosphino)-1,1'-binaphthyl (*rac*-BINAP) (6.2 mg, 10 µmol, 5.0 mol%), and *tert*-butyldimethylchlorosilane (TBDMSCl) (60.3 mg, 0.400 mmol, 2.00 equiv) were dissolved in *N*,*N*-dimethylformamide (DMF) (1.0 mL, c = 0.20 M) and then acrylonitrile (40.2 µL , 31.9 mg, 0.600 mmol, 3.00 equiv) was added. Roughly 100 mL of argon was bubbled through the reaction mixture using a Schlenk line for 30 seconds. The reaction mixture was irradiated with blue LEDs (456 nm, 2 × 40 W) for 12 h at 33 °C. Following irradiation, ethyl acetate (30 mL) and brine (30 mL) were added. The mixture was extracted once with ethyl acetate (30 mL), and the organic layer was washed with brine (30 mL), dried over Na_2SO_4 and concentrated under reduced pressure. The resulting residue was purified by flash column chromatography on silica gel (hexanes/EtOAc 10:1) to afford *rac*-**6** as yellow solid (37.2 mg, 43%).

R_f = 0.36 (hexanes/EtOAc 10:1).

NMR Spectroscopy:

^{1}H NMR (500 MHz, $CDCl_3$, 23 °C, δ): 4.51 (t, *J* = 6.4 Hz, 1H), 2.25 – 2.10 (m, 4H), 2.01 – 1.90 (m, 2H).

^{13}C{^{19}F} NMR (151 MHz, $CDCl_3$, 23 °C, δ): 118.1 (q, *J* = 3.8 Hz), 117.3, 116.8 – 116.3 (m), 111.2, 111.0, 110.4, 108.6, 42.0 (dt, *J* = 158.5, 4.3 Hz), 36.4 – 35.4 (m), 30.0 (t, *J* = 129.7 Hz), 19.0 – 14.9 (m).

^{19}F NMR (470 MHz, $CDCl_3$, 23 °C, δ): –80.8 (t, *J* = 9.9 Hz, 3F), –114.2 (ddt, *J* = 18.6, 13.9, 4.7 Hz, 2F), –121.5 – –122.3 (m, 2F), –122.9 (tqd, *J* = 14.8, 10.4, 4.9 Hz, 2F), –123.5 (td, *J* = 14.1, 7.0 Hz, 2F), –126.0 – –126.2 (m, 2F).

HRMS-EI (m/z) calc'd for $C_{11}H_7F_{13}ClN$ $[M]^+$, 435.0053; found, 435.0058; deviation: +0.9 ppm.

rac-Azetidine-derived α-chloronitrile 7

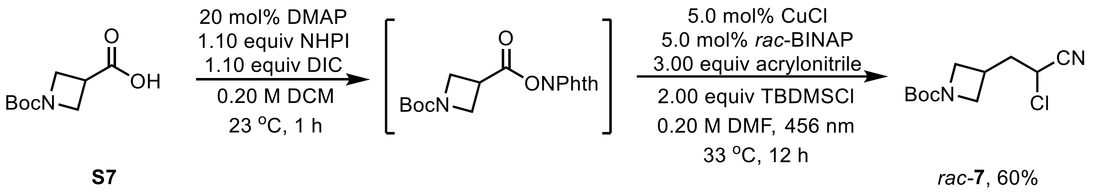

S7 *rac*-**7**, 60%

Under an ambient atmosphere, to a 4-mL borosilicate vial equipped with a Teflon-coated magnetic stir bar were added 1-(tert-butoxycarbonyl)azetidine-3-carboxylic acid **S7** (40.2 mg, 0.20 mmol, 1.00 equiv), *N*-hydroxyphthalimide (NHPI) (35.8 mg, 0.220 mmol, 1.10 equiv), 4-dimethylamino-pyridine (DMAP) (4.90 mg, 40 µmol, 20 mol%), *N*,*N*'-diisopropylcarbodiimide (DIC) (27.7 mg, 0.220 mmol, 1.10 equiv), and dichloromethane (DCM) (1.0 mL, c = 0.20 M). After stirring the mixture at 23 °C for 1 h, the solvent was removed under reduced pressure. Without purification of the crude redox active ester, copper chloride (CuCl) (1.0 mg, 10 µmol, 5.0 mol%), *rac*-2,2'-bis-(diphenylphosphino)-1,1'-binaphthyl (*rac*-BINAP) (6.2 mg, 10 µmol, 5.0 mol%), and *tert*-butyldimethylchlorosilane (TBDMSCl) (60.3 mg, 0.400 mmol, 2.00 equiv) were dissolved in *N*,*N*-dimethylformamide (DMF) (1.0 mL, c = 0.20 M) and then acrylonitrile (40.2 µL , 31.9 mg, 0.600 mmol, 3.00 equiv) was added. Roughly 100 mL of argon was bubbled through the reaction mixture using a Schlenk

line for 30 seconds. The reaction mixture was irradiated with blue LEDs (456 nm, 2 × 40 W) for 12 h at 33 °C. Following irradiation, ethyl acetate (30 mL) and brine (30 mL) were added. The mixture was extracted once with ethyl acetate (30 mL), and the organic layer was washed with brine (30 mL), dried over Na_2SO_4 and concentrated under reduced pressure. The resulting residue was purified by flash column chromatography on silica gel (hexanes/EtOAc 4:1) to afford *rac*-**7** as colorless oil (29.7 mg, 60%).

R*f* = 0.20 (hexanes/EtOAc 4:1).

NMR Spectroscopy:

^{1}H NMR (500 MHz, $CDCl_3$, 23 °C, δ): 4.44 (t, *J* = 6.5 Hz, 1H), 4.12 (td, *J* = 8.5, 3.4 Hz, 2H), 3.69 (ddd, *J* = 8.4, 5.6, 2.2 Hz, 2H), 2.85 (tdd, *J* = 9.8, 7.8, 4.0 Hz, 1H), 2.38 (t, *J* = 7.0 Hz, 2H), 1.43 (s, 9H).

^{13}C NMR (125 MHz, $CDCl_3$, 23 °C, δ): 156.2, 116.5, 79.9, 40.8, 40.2, 28.4, 26.1.

HRMS-ESIpos (m/z) calc'd for $C_{11}H_{17}ClN_2O_2Na$ $[M+Na]^+$, 267.0870; found, 267.0871; deviation: +0.3 ppm.

rac-Fluorochromane-derived α-chloronitrile 8

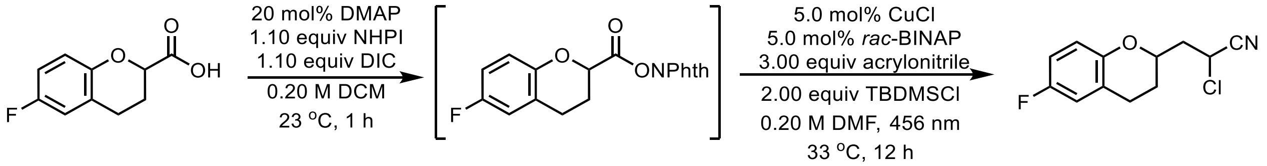

S8 *rac*-**8**, 58%, 1:1.2 dr

Under an ambient atmosphere, to a 4-mL borosilicate vial equipped with a Teflon-coated magnetic stir bar were added 6-fluorochromane-2-carboxylic acid **S8** (39.2 mg, 0.20 mmol, 1.00 equiv), *N*-hydroxyphthalimide (NHPI) (35.8 mg, 0.220 mmol, 1.10 equiv), 4-dimethylamino-pyridine (DMAP) (4.90 mg, 40 µmol, 20 mol%), *N*,*N*'-diisopropylcarbodiimide (DIC) (27.7 mg, 0.220 mmol, 1.10 equiv), and dichloromethane (DCM) (1.0 mL, c = 0.20 M). After stirring the mixture at 23 °C for 1 h, the solvent was removed under reduced pressure. Without purification of the crude redox active ester, copper chloride (CuCl) (1.0 mg, 10 µmol, 5.0 mol%), *rac*-2,2'-bis-(diphenylphosphino)-1,1'-binaphthyl (*rac*-BINAP) (6.2 mg, 10 µmol, 5.0 mol%), and *tert*-butyldimethylchlorosilane (TBDMSCl) (60.3 mg, 0.400 mmol, 2.00 equiv) were dissolved in *N*,*N*-dimethylformamide (DMF) (1.0 mL, c = 0.20 M) and then acrylonitrile (40.2 µL , 31.9 mg, 0.600 mmol, 3.00 equiv) was added. Roughly 100 mL of argon was bubbled through the reaction mixture using a Schlenk line for 30 seconds. The reaction mixture was irradiated with blue LEDs (456 nm, 2 × 40 W) for 12 h at 33 °C. Following irradiation, ethyl acetate (30 mL) and brine (30 mL) were added. The mixture was extracted once with ethyl acetate (30 mL), and the organic layer was washed with brine (30 mL), dried over Na_2SO_4 and concentrated under reduced pressure. The diastereomeric ratio of the crude product was determined using ^{1}H NMR spectroscopy. The resulting residue was purified by flash column chromatography on silica gel (hexanes/EtOAc 10:1) to afford *rac*-**8** as colorless oil (27.7 mg, 58%).

R*f* = 0.28 (hexanes/EtOAc 10:1).

NMR Spectroscopy:

^{1}H NMR (500 MHz, $CDCl_3$, 23 °C, δ): 6.84 – 6.70 (m, 3H), 4.88 (ddd, *J* = 24.1, 10.6, 4.2 Hz, 1H), 4.23 (tdt, *J* = 10.2, 4.9, 2.4 Hz, 1H), 2.96 – 2.85 (m, 1H), 2.77 (ddt, *J* = 16.9, 6.1, 3.2 Hz, 1H), 2.49 – 2.38 (m, 1H), 2.38 – 2.25 (m, 1H), 2.03 (ddq, *J* = 11.8, 6.1, 3.1 Hz, 1H), 1.86 – 1.74 (m, 1H).

^{13}C NMR (125 MHz, $CDCl_3$, 23 °C, δ): 157.2 (d, *J* = 238.9 Hz), 149.8, 122.7 (d, *J* = 13.8 Hz), 117.7 (d, *J* = 7.9 Hz), 117.4, 116.8, 115.6 (d, *J* = 23.2 Hz), 114.3 (d, *J* = 23.3 Hz), 72.2 (minor), 70.6 (major), 42.5 (minor), 41.9 (major), 39.2, 27.1 (minor), 26.9 (major), 24.6.

^{19}F NMR (470 MHz, $CDCl_3$, 23 °C, δ): –123.4.

HRMS-EI (m/z) calc'd for $C_{12}H_{11}FClNO$ $[M]^+$, 239.0507; found, 239.0511; deviation: +1.6 ppm.

rac-Benzoylpiperidine-derived α-chloronitrile 9

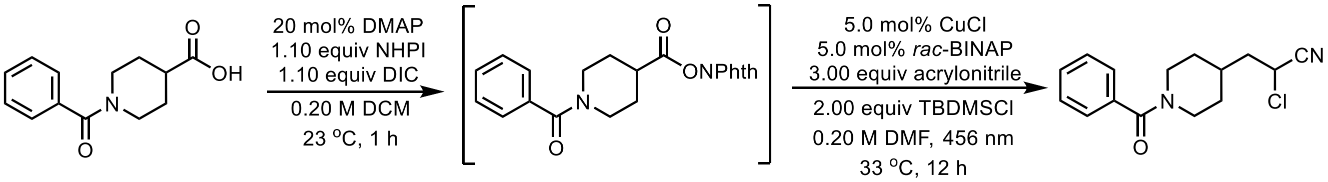

S9 *rac*-**9**, 58%

Under an ambient atmosphere, to a 4-mL borosilicate vial equipped with a Teflon-coated magnetic stir bar were added phenyl benzoylpiperidine -carboxylic acid **S9** (46.6 mg, 0.200 mmol, 1.00 equiv), *N*-hydroxyphthalimide (NHPI) (35.8 mg, 0.220 mmol, 1.10 equiv), 4-dimethylamino-pyridine (DMAP) (4.90 mg, 40 µmol, 20 mol%), *N*,*N*'-diisopropylcarbodiimide (DIC) (27.7 mg, 0.220 mmol, 1.10 equiv), and dichloromethane (DCM) (1.0 mL, c = 0.20 M). After stirring the mixture at 23 °C for 1 h, the solvent was removed under reduced pressure. Without purification of the crude redox active ester, copper chloride (CuCl) (1.0 mg, 10 µmol, 5.0 mol%), *rac*-2,2'-bis-(diphenylphosphino)-1,1'-binaphthyl (*rac*-BINAP) (6.2 mg, 10 µmol, 5.0 mol%), and *tert*-butyldimethylchlorosilane (TBDMSCl) (60.3 mg, 0.400 mmol, 2.00 equiv) were dissolved in *N*,*N*-dimethylformamide (DMF) (1.0 mL, c = 0.20 M) and then acrylonitrile (40.2 µL , 31.9 mg, 0.600 mmol, 3.00 equiv) was added. Roughly 100 mL of argon was bubbled through the reaction mixture using a Schlenk line for 30 seconds. The reaction mixture was irradiated with blue LEDs (456 nm, 2 × 40 W) for 12 h at 33 °C. Following irradiation, ethyl acetate (30 mL) and brine (30 mL) were added. The mixture was extracted once with ethyl acetate (30 mL), and the organic layer was washed with brine (30 mL), dried over Na_2SO_4 and concentrated under reduced pressure. The resulting residue was purified by flash column chromatography on silica gel (hexanes/EtOAc 5:1) to afford *rac*-**9** as colorless oil (32.0 mg, 58%).

R_f = 0.21 (hexanes/EtOAc 5:1).

NMR Spectroscopy:

^{1}H NMR (500 MHz, $CDCl_3$, 23 °C, δ): 7.48 – 7.35 (m, 5H), 4.77 (s, 1H), 4.53 (t, *J* = 7.5 Hz, 1H), 3.81 (s, 1H), 3.04 (s, 1H), 2.81 (s, 1H), 2.06 (td, *J* = 7.1, 2.1 Hz, 2H), 2.01 – 1.90 (m, 1H), 1.91 – 1.66 (m, 2H), 1.51 – 1.11 (m, 2H).

^{13}C NMR (125 MHz, $CDCl_3$, 23 °C, δ): 170.4, 135.9, 129.7, 128.5, 126.8, 117.0, 47.5, 42.4, 41.9, 40.1, 33.2.

HRMS-EI (m/z) calc'd for $C_{15}H_{17}ClNO_2$ $[M]^+$, 276.1024; found, 276.1024; deviation: +0.1 ppm.

rac-Gemfibrozil-derived α-chloronitrile 10

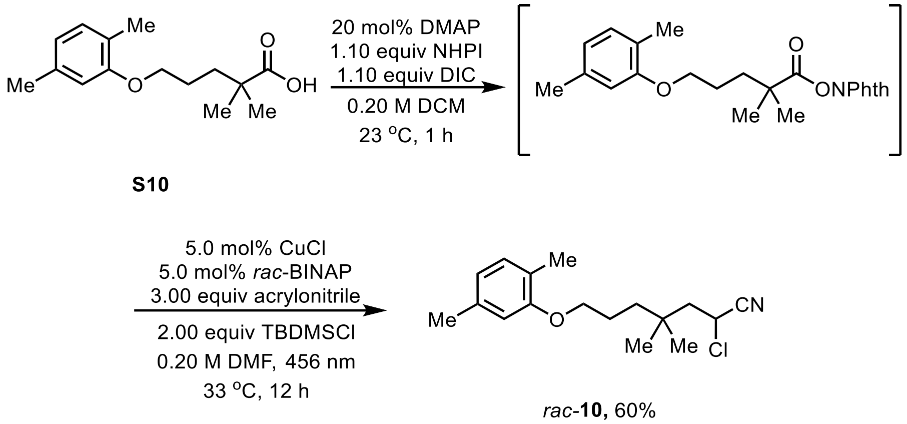

S10

rac-**10,** 60%

Under an ambient atmosphere, to a 4-mL borosilicate vial equipped with a Teflon-coated magnetic stir bar were added phenyl gemfibrozil-carboxylic acid **S10** (50.0 mg, 0.200 mmol, 1.00 equiv), *N*-hydroxyphthalimide (NHPI) (35.8 mg, 0.220 mmol, 1.10 equiv), 4-dimethylamino-pyridine (DMAP) (4.90 mg, 40 µmol, 20 mol%), *N*,*N*'-diisopropylcarbodiimide (DIC) (27.7 mg, 0.220 mmol, 1.10 equiv), and dichloromethane (DCM) (1.0 mL, c = 0.20 M). After stirring the mixture at 23 °C for 1 h, the solvent was removed under reduced pressure. Without purification of the crude redox active ester, copper chloride (CuCl) (1.0 mg, 10 µmol, 5.0 mol%), *rac*-2,2'-bis-(diphenylphosphino)-1,1'-binaphthyl (*rac*-BINAP) (6.2 mg, 10 µmol, 5.0 mol%), and *tert*-butyldimethylchlorosilane (TBDMSCl) (60.3 mg, 0.400 mmol, 2.00 equiv) were dissolved in *N*,*N*-dimethylformamide (DMF) (1.0 mL, c = 0.20 M) and then acrylonitrile (40.2 µL , 31.9 mg, 0.600 mmol, 3.00 equiv) was added. Roughly 100 mL of argon was bubbled through the reaction mixture using a Schlenk line for 30 seconds. The reaction mixture was irradiated with blue LEDs (456 nm, 2 × 40 W) for 12 h at 33 °C. Following irradiation, ethyl acetate (30 mL) and brine (30 mL) were added. The mixture was extracted once with ethyl acetate (30 mL), and the organic layer was washed with brine (30 mL), dried over Na_2SO_4 and concentrated under reduced pressure. The resulting residue was purified by flash column chromatography on silica gel (hexanes/EtOAc 10:1) to afford *rac*-**10** as colorless oil (35.2 mg, 60%).

$\mathbf{R}_f$ = 0.25 (hexanes/EtOAc 10:1).

NMR Spectroscopy:

^{1}H NMR (500 MHz, $CDCl_3$, 23 °C, δ): 7.05 (d, *J* = 7.5 Hz, 1H), 6.71 (d, *J* = 7.5 Hz, 1H), 6.65 (d, *J* = 1.6 Hz, 1H), 4.51 (dd, *J* = 8.8, 5.4 Hz, 1H), 4.03 – 3.90 (m, 2H), 2.35 (s, 3H), 2.25 (dd, *J* = 14.5, 8.8 Hz, 1H), 2.22 (s, 3H), 2.06 (dd, *J* = 14.6, 5.4 Hz, 1H), 1.87 – 1.76 (m, 2H), 1.59 – 1.49 (m, 2H), 1.10 (s, 6H).

^{13}C NMR (125 MHz, $CDCl_3$, 23 °C, δ): 156.8, 136.5, 130.4, 123.5, 120.8, 118.1, 112.0, 67.8, 48.2, 39.1, 38.2, 33.5, 27.1, 27.0, 24.1, 21.4, 15.8.

HRMS-EI (m/z) calc'd for $C_{17}H_{24}ClNO$ $[M]^+$, 293.1541; found, 293.1546; deviation: –1.8 ppm.

rac-Cyclopropane-derived α-chloronitrile 11

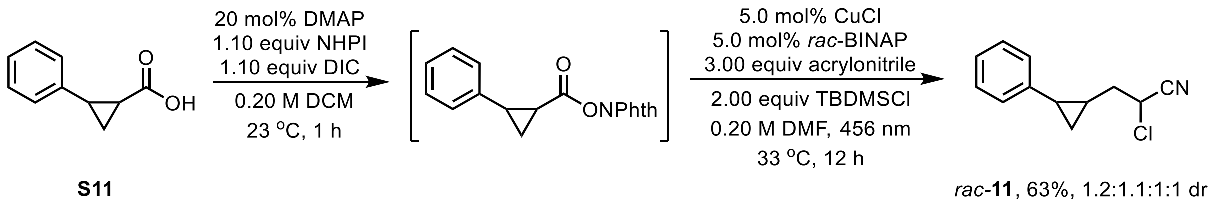

Under an ambient atmosphere, to a 4-mL borosilicate vial equipped with a Teflon-coated magnetic stir bar were added phenyl cyclopropane-carboxylic acid **S11** (32.4 mg, 0.20 mmol, 1.00 equiv), *N*-hydroxyphthalimide (NHPI) (35.8 mg, 0.220 mmol, 1.10 equiv), 4-dimethylamino-pyridine (DMAP) (4.90 mg, 40 µmol, 20 mol%), *N*,*N*'-diisopropylcarbodiimide (DIC) (27.7 mg, 0.220 mmol, 1.10 equiv), and dichloromethane (DCM) (1.0 mL, c = 0.20 M). After stirring the mixture at 23 °C for 1 h, the solvent was removed under reduced pressure. Without purification of the crude redox active ester, copper chloride (CuCl) (1.0 mg, 10 µmol, 5.0 mol%), *rac*-2,2'-bis-(diphenylphosphino)-1,1'-binaphthyl (*rac*-BINAP) (6.2 mg, 10 µmol, 5.0 mol%), and *tert*-butyldimethylchlorosilane (TBDMSCl) (60.3 mg, 0.400 mmol, 2.00 equiv) were dissolved in *N*,*N*-dimethylformamide (DMF) (1.0 mL, c = 0.20 M) and then acrylonitrile (40.2 µL , 31.9 mg, 0.600 mmol, 3.00 equiv) was added. Roughly 100 mL of argon was bubbled through the reaction mixture using a Schlenk line for 30 seconds. The reaction mixture was irradiated with blue LEDs (456 nm, 2 × 40 W) for 12 h at 33 °C. Following irradiation, ethyl acetate (30 mL) and brine (30 mL) were added. The mixture was extracted once with ethyl acetate (30 mL), and the organic layer was washed with brine (30 mL), dried over Na_2SO_4 and concentrated under reduced pressure. The diastereomeric ratio of the crude product was determined using HPLC spectroscopy. The resulting residue was purified by flash column chromatography on silica gel (hexanes/EtOAc 10:1) to afford *rac*-**11** as brown oil (26.0 mg, 63%).

R*f* = 0.51 (hexanes/EtOAc 10:1).

NMR Spectroscopy:

^{1}H NMR (500 MHz, $CDCl_3$, 23 °C, δ): 7.28 (t, *J* = 7.7 Hz, 2H), 7.24 – 7.16 (m, 1H), 7.14 – 7.07 (m, 2H), 4.53 (td, *J* = 7.0, 3.7 Hz, 1H), 2.23 – 2.09 (m, 2H), 1.95 – 1.84 (m, 1H), 1.35 – 1.17 (m, 1H), 1.11 (ddt, *J* = 8.4, 6.8, 5.3 Hz, 1H), 0.96 (dq, *J* = 8.7, 5.6 Hz, 1H).

^{13}C NMR (125 MHz, $CDCl_3$, 23 °C, δ): 141.6, 128.5, 126.1, 126.0, 117.2, 42.3, 42.2, 41.0, 40.9, 23.0, 22.9, 19.3, 19.3, 15.0.

HRMS-EI (m/z) calc'd for $C_{12}H_{12}ClN$ $[M]^+$, 205.0652; found, 205.0650; deviation: –0.9 ppm.

HPLC (OJ-3R, Methanol /Water 70:30 for 10 min then 90:10, 298 K, 220 nm): tR (isomer 1) = 12.5 min, tR (isomer 2) = 13.0 min, tR (isomer 3) = 13.2 min, tR (isomer 4) = 14.2 min.

rac-Adamantane-derived α-chloronitrile 12

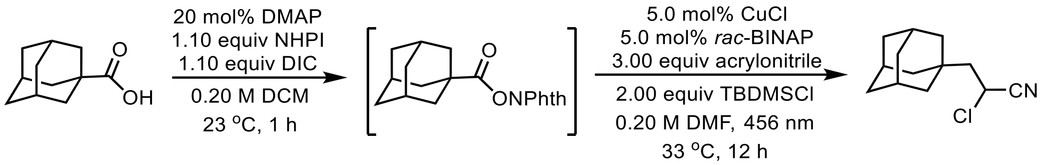

S12 *rac*-**12**, 49%

Under an ambient atmosphere, to a 4-mL borosilicate vial equipped with a Teflon-coated magnetic stir bar were added adamantane-1-carboxylic acid **S12** (36.0 mg, 0.20 mmol, 1.00 equiv), *N*-hydroxyphthalimide (NHPI) (35.8 mg, 0.220 mmol, 1.10 equiv), 4-dimethylamino-pyridine (DMAP) (4.90 mg, 40 µmol, 20 mol%), *N*,*N*'-diisopropylcarbodiimide (DIC) (27.7 mg, 0.220 mmol, 1.10 equiv), and dichloromethane (DCM) (1.0 mL, c = 0.20 M). After stirring the mixture at 23 °C for 1 h, the solvent was removed under reduced pressure. Without purification of the crude redox active ester, copper chloride (CuCl) (1.0 mg, 10 µmol, 5.0 mol%), *rac*-2,2'-bis-(diphenylphosphino)-1,1'-binaphthyl (*rac*-BINAP) (6.2 mg, 10 µmol, 5.0 mol%), and *tert*-butyldimethylchlorosilane (TBDMSCl) (60.3 mg, 0.400 mmol, 2.00 equiv) were dissolved in *N*,*N*-dimethylformamide (DMF) (1.0 mL, c = 0.20 M) and then acrylonitrile (40.2 µL , 31.9 mg, 0.600 mmol, 3.00 equiv) was added. Roughly 100 mL of argon was bubbled through the reaction mixture using a Schlenk line for 30 seconds. The reaction mixture was irradiated with blue LEDs (456 nm, 2 × 40 W) for 12 h at 33 °C. Following irradiation, ethyl acetate (30 mL) and brine (30 mL) were added. The mixture was extracted once with ethyl acetate (30 mL), and the organic layer was washed with brine (30 mL), dried over Na_2SO_4 and concentrated under reduced pressure. The resulting residue was purified by flash column chromatography on silica gel (Pentane/EtOAc 40:1) to afford *rac*-**12** as colorless solid (22.0 mg, 49%).

$\mathbf{R}_f$ = 0.25 (Pentane/EtOAc 40:1).

NMR Spectroscopy:

^{1}H NMR (500 MHz, $CDCl_3$, 23 °C, δ): 4.48 (dd, *J* = 8.9, 5.2 Hz, 1H), 2.19 – 1.95 (m, 4H), 1.84 (dd, *J* = 14.6, 5.2 Hz, 1H), 1.73 (d, *J* = 12.4 Hz, 3H), 1.68 – 1.59 (m, 9H).

^{13}C NMR (125 MHz, $CDCl_3$, 23 °C, δ): 118.5, 51.1, 42.1, 38.3, 36.7, 33.2, 28.4.

HRMS-ESIpos (m/z) calc'd for $C_{13}H_{18}ClNNa$ $[M+Na]^+$, 246.1020; found, 246.1021; deviation: +0.4 ppm.

rac-Glutamic acid-derived α-chloronitrile 13

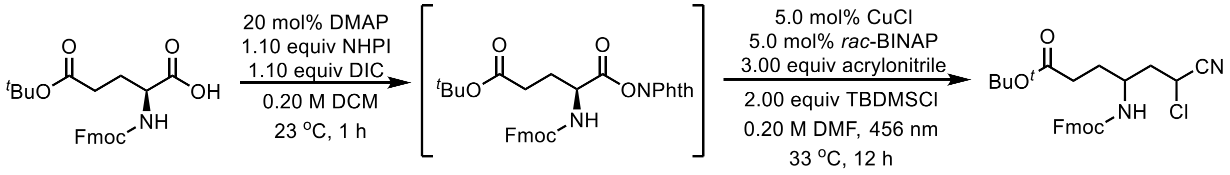

S13 *rac*-**13**, 55%, 1.2:1 dr

Under an ambient atmosphere, to a 4-mL borosilicate vial equipped with a Teflon-coated magnetic stir bar were added Fmoc-Glu(O^{t}Bu)-OH **S13** (84.7 mg, 0.20 mmol, 1.00 equiv), *N*-hydroxyphthalimide (NHPI) (35.8

mg, 0.220 mmol, 1.10 equiv), 4-dimethylamino-pyridine (DMAP) (4.90 mg, 40 µmol, 20 mol%), *N*,*N*'-diisopropylcarbodiimide (DIC) (27.7 mg, 0.220 mmol, 1.10 equiv), and dichloromethane (DCM) (1.0 mL, c = 0.20 M). After stirring the mixture at 23 °C for 1 h, the solvent was removed under reduced pressure. Without purification of the crude redox active ester, copper chloride (CuCl) (1.0 mg, 10 µmol, 5.0 mol%), *rac*-2,2'-bis-(diphenylphosphino)-1,1'-binaphthyl (*rac*-BINAP) (6.2 mg, 10 µmol, 5.0 mol%), and *tert*-butyldimethylchlorosilane (TBDMSCl) (60.3 mg, 0.400 mmol, 2.00 equiv) were dissolved in *N*,*N*-dimethylformamide (DMF) (1.0 mL, c = 0.20 M) and then acrylonitrile (40.2 µL , 31.9 mg, 0.600 mmol, 3.00 equiv) was added. Roughly 100 mL of argon was bubbled through the reaction mixture using a Schlenk line for 30 seconds. The reaction mixture was irradiated with blue LEDs (456 nm, 2 × 40 W) for 12 h at 33 °C. Following irradiation, ethyl acetate (30 mL) and brine (30 mL) were added. The mixture was extracted once with ethyl acetate (30 mL), and the organic layer was washed with brine (30 mL), dried over Na_2SO_4 and concentrated under reduced pressure. The diastereomeric ratio of the crude product was determined using HPLC spectroscopy. The resulting residue was purified by flash column chromatography on silica gel (hexanes/EtOAc 9:1) to afford *rac*-**13** as colorless oil (52.0 mg, 55%).

R_f = 0.16 (hexanes/EtOAc 9:1).

NMR Spectroscopy:

^{1}H NMR (600 MHz, CD_3CN, 23 °C, δ): 7.83 (d, *J* = 7.6 Hz, 2H), 7.66 (d, *J* = 7.5 Hz, 2H), 7.42 (t, *J* = 7.5 Hz, 2H), 7.34 (t, *J* = 7.5 Hz, 2H), 5.58 (dd, *J* = 35.0, 9.3 Hz, 1H), 4.64 (dt, *J* = 9.3, 4.5 Hz, 1H), 4.39 (dd, *J* = 7.3, 4.5 Hz, 2H), 4.22 (t, *J* = 6.8 Hz, 1H), 3.77 (ddp, *J* = 13.4, 9.0, 4.7 Hz, 1H), 2.35 – 2.09 (m, 5H), 1.81 – 1.59 (m, 2H), 1.41 (s, 9H).

^{13}C NMR (151 MHz, CD_3CN , 23 °C, δ): 173.0, 157.1, 145.1, 142.2, 128.6, 128.0, 126.2, 120.9, 80.9, 66.8, 49.1, 48.4, 48.2, 42.6, 42.0, 41.5, 41.5, 32.3, 32.3, 30.8, 30.6, 29.4, 28.2, 27.6.

HRMS-ESIpos (m/z) calc'd for $C_{26}H_{29}ClN_2O_4Na$ $[M+Na]^+$, 491.1708; found, 491.1706; deviation: –0.3 ppm.

HPLC (Eclipse Plus C18, Methanol /Water 70% - 5' - 95%, 308 K, 220 nm): t_R (minor) = 2.9 min, t_R (major) = 3.0 min, dr = 1.2:1

Lithocholic-derived α-chloronitrile 14

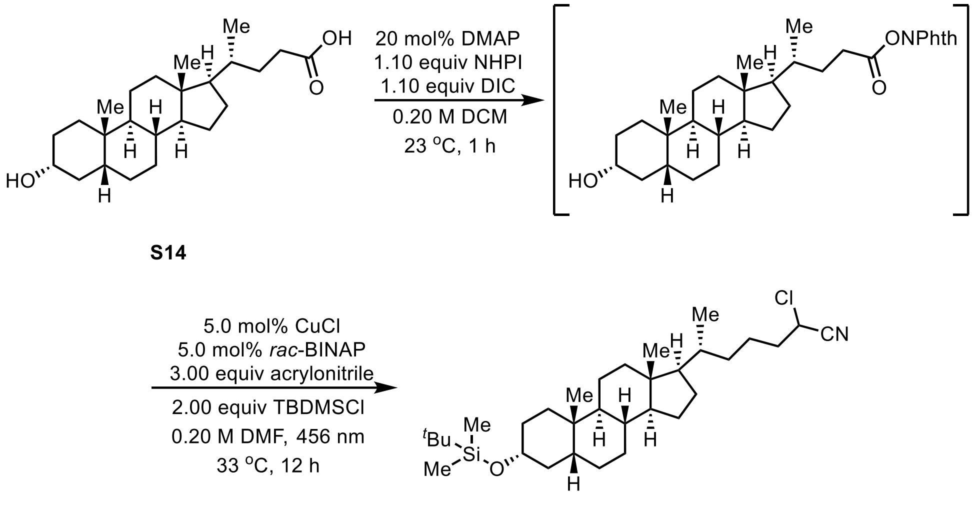

S14

14, 50%, 1:1 dr

Under an ambient atmosphere, to a 4-mL borosilicate vial equipped with a Teflon-coated magnetic stir bar were added Lithocholic-carboxylic acid **S14** (75.3 mg, 0.20 mmol, 1.00 equiv), *N*-hydroxyphthalimide (NHPI) (35.8 mg, 0.220 mmol, 1.10 equiv), 4-dimethylamino-pyridine (DMAP) (4.90 mg, 40 µmol, 20 mol%), *N*,*N*'-diisopropylcarbodiimide (DIC) (27.7 mg, 0.220 mmol, 1.10 equiv), and dichloromethane (DCM) (1.0 mL, c = 0.20 M). After stirring the mixture at 23 °C for 1 h, the solvent was removed under reduced pressure. Without purification of the crude redox active ester, copper chloride (CuCl) (1.0 mg, 10 µmol, 5.0 mol%), *rac*-2,2'-bis-(diphenylphosphino)-1,1'-binaphthyl (*rac*-BINAP) (6.2 mg, 10 µmol, 5.0 mol%), and *tert*-butyldimethylchlorosilane (TBDMSCl) (60.3 mg, 0.400 mmol, 2.00 equiv) were dissolved in *N*,*N*-dimethylformamide (DMF) (1.0 mL, c = 0.20 M) and then acrylonitrile (40.2 µL , 31.9 mg, 0.600 mmol, 3.00 equiv) was added. Roughly 100 mL of argon was bubbled through the reaction mixture using a Schlenk line for 30 seconds. The reaction mixture was irradiated with blue LEDs (456 nm, 2 × 40 W) for 12 h at 33 °C. Following irradiation, ethyl acetate (30 mL) and brine (30 mL) were added. The mixture was extracted once with ethyl acetate (30 mL), and the organic layer was washed with brine (30 mL), dried over Na_2SO_4 and concentrated under reduced pressure. The diastereomeric ratio of the crude product was determined using ^{1}H NMR spectroscopy. The resulting residue was purified by flash column chromatography on silica gel (Pentane/EtOAc 20:1) to afford **14** as colorless oil (60.0 mg, 56%).

R_f = 0.40 (Pentane/EtOAc 10:1).

NMR Spectroscopy:

^{1}H NMR (500 MHz, $CDCl_3$, 23 °C, δ): 4.43 (td, *J* = 6.9, 1.0 Hz, 1H), 3.58 (tt, *J* = 10.9, 4.6 Hz, 1H), 2.28 – 1.90 (m, 3H), 1.89 – 1.71 (m, 4H), 1.68 – 1.59 (m, 0H), 1.55 (ddd, *J* = 14.3, 6.3, 3.6 Hz, 1H), 1.50 – 1.30 (m, 7H), 1.28 – 1.18 (m, 2H), 1.17 – 1.01 (m, 5H), 0.98 – 0.81 (m, 17H), 0.63 (s, 3H), 0.06 (s, 6H).

^{13}C NMR (125 MHz, $CDCl_3$, 23 °C, δ): 117.3, 72.9, 56.5, 56.2, 42.8, 42.6, 42.4, 40.3, 40.3, 37.0, 36.9, 36.0, 35.7, 34.9, 34.7, 31.1, 28.5, 27.4, 26.5, 26.1, 24.3, 23.5, 22.6, 22.5, 20.9, 18.6, 18.4, 12.1, –4.4.

HRMS-ESIpos (m/z) calc'd for $C_{32}H_{56}ClNOSiNa$ $[M+Na]^+$, 556.3711; found, 556.3715; deviation: +0.6 ppm.

rac-Difluorocyclobutane-derived α-chloronitrile 15

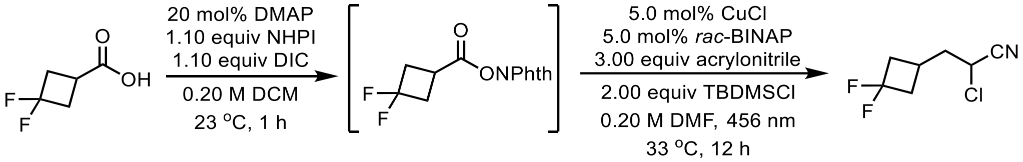

S15 *rac*-**15**, 56%

Under an ambient atmosphere, to a 4-mL borosilicate vial equipped with a Teflon-coated magnetic stir bar were added 3,3-difluorocyclobutane-1-carboxylic acid **S15** (27.2 mg, 0.20 mmol, 1.00 equiv), *N*-hydroxyphthalimide (NHPI) (35.8 mg, 0.220 mmol, 1.10 equiv), 4-dimethylamino-pyridine (DMAP) (4.90 mg, 40 µmol, 20 mol%), *N*,*N*'-diisopropylcarbodiimide (DIC) (27.7 mg, 0.220 mmol, 1.10 equiv), and dichloromethane (DCM) (1.0 mL, c = 0.20 M). After stirring the mixture at 23 °C for 1 h, the solvent was removed under reduced pressure. Without purification of the crude redox active ester, copper chloride (CuCl) (1.0 mg, 10 µmol, 5.0 mol%), *rac*-2,2'-bis-(diphenylphosphino)-1,1'-binaphthyl (*rac*-BINAP) (6.2 mg, 10 µmol, 5.0 mol%), and *tert*-butyldimethylchlorosilane (TBDMSCl) (60.3 mg, 0.400 mmol, 2.00 equiv) were dissolved in *N*,*N*-dimethylformamide (DMF) (1.0 mL, c = 0.20 M) and then acrylonitrile (40.2 µL , 31.9 mg, 0.600 mmol, 3.00 equiv) was added. Roughly 100 mL of argon was bubbled through the reaction mixture using a Schlenk line for 30 seconds. The reaction mixture was irradiated with blue LEDs (456 nm, 2 × 40 W) for 12 h at 33 °C. Following irradiation, ethyl acetate (30 mL) and brine (30 mL) were added. The mixture was extracted once with ethyl acetate (30 mL), and the organic layer was washed with brine (30 mL), dried over Na_2SO_4 and concentrated under reduced pressure. The resulting residue was purified by flash column chromatography on silica gel (Pentane/MTBE 3:1) to afford *rac*-**15** as yellow oil (20 mg, 56%).

R_f = 0.20 (Pentane/MTBE 3:1).

NMR Spectroscopy:

^{1}H NMR (500 MHz, $CDCl_3$, 23 °C, δ): 4.42 (t, *J* = 6.7 Hz, 1H), 2.90 – 2.76 (m, 2H), 2.54 – 2.40 (m, 1H), 2.39 – 2.31 (m, 2H), 2.28 (t, *J* = 7.0 Hz, 2H).

^{13}C NMR (125 MHz, $CDCl_3$, 23 °C, δ): 119.5 (dd, *J* = 282.9, 274.6 Hz), 116.6, 41.6 (d, *J* = 2.2 Hz), 41.0, 40.9 – 40.5 (m), 20.5 (dd, *J* = 13.0, 6.4 Hz).

^{19}F NMR (470 MHz, $CDCl_3$, 23 °C, δ): –82.79 (d, *J* = 195.1 Hz, 1F), –95.74 (d, *J* = 195.1 Hz, 1F).

HRMS-ESIpos (m/z) calc'd for $C_7H_8F_2ClNNa$ $[M+Na]^+$, 202.0205; found, 202.0205; deviation: +0.2 ppm.

rac-Dihydrobenzodioxine-derived α-chloronitrile 16

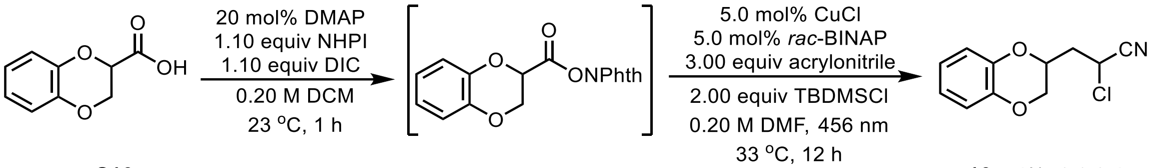

S16 *rac*-**16**, 54%, 1:1.1 dr

Under an ambient atmosphere, to a 4-mL borosilicate vial equipped with a Teflon-coated magnetic stir bar were added dihydrobenzodioxine carboxylic acid **S16** (36.0 mg, 0.200 mmol, 1.00 equiv), *N*-hydroxyphthalimide (NHPI) (35.8 mg, 0.220 mmol, 1.10 equiv), 4-dimethylamino-pyridine (DMAP) (4.90 mg, 40 µmol, 20 mol%), *N*,*N*'-diisopropylcarbodiimide (DIC) (27.7 mg, 0.220 mmol, 1.10 equiv), and dichloromethane (DCM) (1.0 mL, c = 0.20 M). After stirring the mixture at 23 °C for 1 h, the solvent was removed under reduced pressure. Without purification of the crude redox active ester, copper chloride (CuCl) (1.0 mg, 10 µmol, 5.0 mol%), *rac*-2,2'-bis-(diphenylphosphino)-1,1'-binaphthyl (*rac*-BINAP) (6.2 mg, 10 µmol, 5.0 mol%), and *tert*-butyldimethylchlorosilane (TBDMSCl) (60.3 mg, 0.400 mmol, 2.00 equiv) were dissolved in *N*,*N*-dimethylformamide (DMF) (1.0 mL, c = 0.20 M) and then acrylonitrile (40.2 µL , 31.9 mg, 0.600 mmol, 3.00 equiv) was added. Roughly 100 mL of argon was bubbled through the reaction mixture using a Schlenk line for 30 seconds. The reaction mixture was irradiated with blue LEDs (456 nm, 2 × 40 W) for 12 h at 33 °C. Following irradiation, ethyl acetate (30 mL) and brine (30 mL) were added. The mixture was extracted once with ethyl acetate (30 mL), and the organic layer was washed with brine (30 mL), dried over Na_2SO_4 and concentrated under reduced pressure. The diastereomeric ratio of the crude product was determined using ^1H NMR spectroscopy. The resulting residue was purified by flash column chromatography on silica gel (Pentane/EtOAc 10:1) to afford *rac*-**16** as yellow oil (24.1 mg, 54%).

R*f* = 0.20 (Pentane/EtOAc 10:1).

NMR Spectroscopy:

^{1}H NMR (500 MHz, $CDCl_3$, 23 °C, δ): 6.96 – 6.88 (m, 4H), 4.88 (dd, *J* = 11.0, 3.2 Hz, 1H), 4.55 – 4.43 (m, 1H), 4.29 (d, *J* = 2.4 Hz, 1H), 4.05 – 3.98 (m, 1H), 2.56 – 2.43 (m, 1H), 2.38 – 2.24 (m, 1H).

^{13}C NMR (125 MHz, $CDCl_3$, 23 °C, δ): 142.8, 142.8, 141.8, 141.8, 122.1, 122.1, 117.5, 117.4, 117.4, 116.8, 116.3, 69.4, 68.1, 66.7, 66.7, 38.8, 38.7, 37.9, 37.4.

HRMS-EI (m/z) calc'd for $C_{11}H_{10}ClO_2N$ $[M]^+$, 223.0395; found, 223.0399; deviation: –1.9 ppm.

Dehydroabietic-derived α-chloronitrile 17

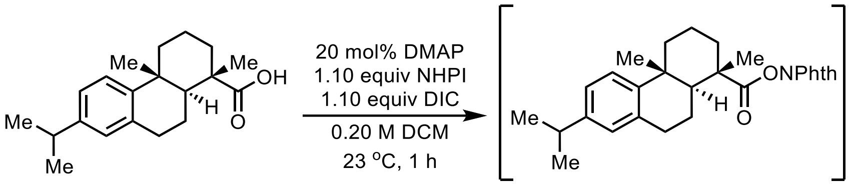

S17

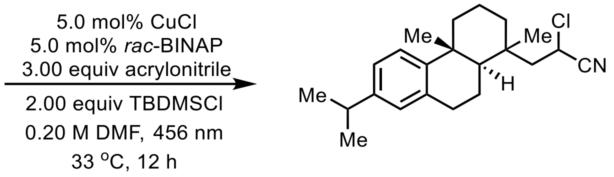

17, 61%, 1.2:1 dr

Under an ambient atmosphere, to a 4-mL borosilicate vial equipped with a Teflon-coated magnetic stir bar were added dehydroabietic-carboxylic acid **S17** (60.1 mg, 0.20 mmol, 1.00 equiv), *N*-hydroxyphthalimide (NHPI) (35.8 mg, 0.220 mmol, 1.10 equiv), 4-dimethylamino-pyridine (DMAP) (4.90 mg, 40 µmol, 20 mol%), *N*,*N*'-diisopropylcarbodiimide (DIC) (27.7 mg, 0.220 mmol, 1.10 equiv), and dichloromethane (DCM) (1.0 mL, c = 0.20 M). After stirring the mixture at 23 °C for 1 h, the solvent was removed under reduced pressure. Without purification of the crude redox active ester, copper chloride (CuCl) (1.0 mg, 10 µmol, 5.0 mol%), *rac*-2,2'-bis-(diphenylphosphino)-1,1'-binaphthyl (*rac*-BINAP) (6.2 mg, 10 µmol, 5.0 mol%), and *tert*-butyldimethylchlorosilane (TBDMSCl) (60.3 mg, 0.400 mmol, 2.00 equiv) were dissolved in *N*,*N*-dimethylformamide (DMF) (1.0 mL, c = 0.20 M) and then acrylonitrile (40.2 µL , 31.9 mg, 0.600 mmol, 3.00 equiv) was added. Roughly 100 mL of argon was bubbled through the reaction mixture using a Schlenk line for 30 seconds. The reaction mixture was irradiated with blue LEDs (456 nm, 2 × 40 W) for 12 h at 33 °C. Following irradiation, ethyl acetate (30 mL) and brine (30 mL) were added. The mixture was extracted once with ethyl acetate (30 mL), and the organic layer was washed with brine (30 mL), dried over Na_2SO_4 and concentrated under reduced pressure. The diastereomeric ratio of the crude product was determined using HPLC spectroscopy. The resulting residue was purified by flash column chromatography on silica gel (hexanes/EtOAc 10:1) to afford **17** as colorless oil (42.3 mg, 61%).

R_f = 0.52 (hexanes/EtOAc 10:1).

NMR Spectroscopy:

^{1}H NMR (500 MHz, $CDCl_3$, 23 °C, δ): 7.18 (dd, *J* = 8.1, 2.4 Hz, 1H), 7.02 (ddd, *J* = 7.9, 4.6, 2.0 Hz, 1H), 6.92 (d, *J* = 2.1 Hz, 1H), 4.48 (dd, *J* = 8.8, 4.6 Hz, 1H), 3.03 – 2.94 (m, 1H), 2.93 – 2.81 (m, 2H), 2.38 – 2.26 (m, 2H), 2.09 (ddd, *J* = 39.8, 14.7, 4.7 Hz, 1H), 1.87 – 1.71 (m, 4H), 1.63 – 1.59 (m, 1H), 1.44 (dtt, *J* = 13.4, 8.0, 4.0 Hz, 3H), 1.27 – 1.22 (m, 9H), 1.09 (d, *J* = 23.4 Hz, 3H).

^{13}C NMR (125 MHz, $CDCl_3$, 23 °C, δ): 146.9, 146.7, 145.9, 145.9, 134.4, 134.3, 127.0, 126.9, 124.1, 124.0, 118.4, 118.4, 51.1, 50.8, 48.2, 47.9, 38.7, 38.2, 38.1, 37.8, 37.7, 37.3, 37.3, 33.5, 29.9, 29.6, 25.5, 25.4, 24.1,

24.0, 22.4, 20.5, 20.2, 19.2, 19.1, 18.7, 18.7, 14.2.

HRMS-EI (m/z) calc'd for $C_{22}H_{30}ClN$ $[M]^+$, 343.2061; found, 343.2059; deviation: –0.5 ppm.

HPLC (Eclipse Plus C18, Methanol /Water 70% - 10' - 95%, 308 K, 220 nm): t_R (major) = 10.7 min, t_R (minor) = 10.8 min, dr = 1.2:1

rac-Bezafibrate-derived α-chloronitrile 18

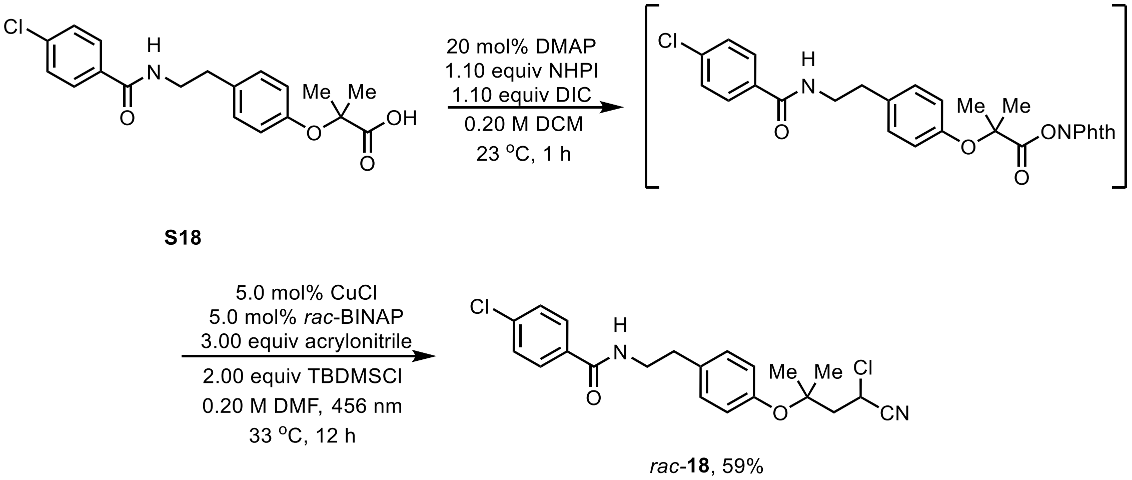

Under an ambient atmosphere, to a 4-mL borosilicate vial equipped with a Teflon-coated magnetic stir bar were added Bezafibrate acid **S18** (72.3 mg, 0.20 mmol, 1.00 equiv), *N*-hydroxyphthalimide (NHPI) (35.8 mg, 0.220 mmol, 1.10 equiv), 4-dimethylamino-pyridine (DMAP) (4.90 mg, 40 µmol, 20 mol%), *N*,*N*'-diisopropylcarbodiimide (DIC) (27.7 mg, 0.220 mmol, 1.10 equiv), and dichloromethane (DCM) (1.0 mL, c = 0.20 M). After stirring the mixture at 23 °C for 1 h, the solvent was removed under reduced pressure. Without purification of the crude redox active ester, copper chloride (CuCl) (1.0 mg, 10 µmol, 5.0 mol%), *rac*-2,2'-bis-(diphenylphosphino)-1,1'-binaphthyl (*rac*-BINAP) (6.2 mg, 10 µmol, 5.0 mol%), and *tert*-butyldimethylchlorosilane (TBDMSCl) (60.3 mg, 0.400 mmol, 2.00 equiv) were dissolved in *N*,*N*-dimethylformamide (DMF) (1.0 mL, c = 0.20 M) and then acrylonitrile (40.2 µL , 31.9 mg, 0.600 mmol, 3.00 equiv) was added. Roughly 100 mL of argon was bubbled through the reaction mixture using a Schlenk line for 30 seconds. The reaction mixture was irradiated with blue LEDs (456 nm, 2 × 40 W) for 12 h at 33 °C. Following irradiation, ethyl acetate (30 mL) and brine (30 mL) were added. The mixture was extracted once with ethyl acetate (30 mL), and the organic layer was washed with brine (30 mL), dried over Na_2SO_4 and concentrated under reduced pressure. The resulting residue was purified by flash column chromatography on silica gel (hexanes/EtOAc 10:2 then 10:3) to afford *rac*-**18** as colorless solid (47.7 mg, 59%).

$\mathbf{R}_f$ = 0.12 (hexanes/EtOAc 10:2 then 10:3).

NMR Spectroscopy:

^{1}H NMR (500 MHz, $CDCl_3$, 23 °C, δ): 7.62 (d, *J* = 8.5 Hz, 2H), 7.37 (d, *J* = 8.4 Hz, 2H), 7.17 – 7.08 (m, 2H), 6.92 (d, *J* = 8.4 Hz, 2H), 6.32 – 6.17 (m, 1H), 4.90 (dd, *J* = 8.6, 4.9 Hz, 1H), 3.91 – 3.51 (m, 2H), 2.88 (t, *J* =

7.0 Hz, 2H), 2.57 (dd, J = 14.6, 8.7 Hz, 1H), 2.39 (dd, J = 14.6, 4.9 Hz, 1H), 1.36 (d, J = 25.3 Hz, 6H).

^{13}C NMR (125 MHz, $CDCl_3$, 23 °C, δ): 166.5, 152.7, 137.7, 133.0, 129.6, 128.9, 128.3, 124.2, 118.1, 78.3, 49.3, 41.3, 38.3, 35.0, 26.8, 26.6.

HRMS-ESIpos (m/z) calc'd for $C_{21}H_{22}Cl_2N_2O_2Na$ $[M+Na]^+$, 427.0950; found, 427.0947; deviation: –0.8 ppm.

(Methyl-d$_3$)-derived α-chloro amide 19

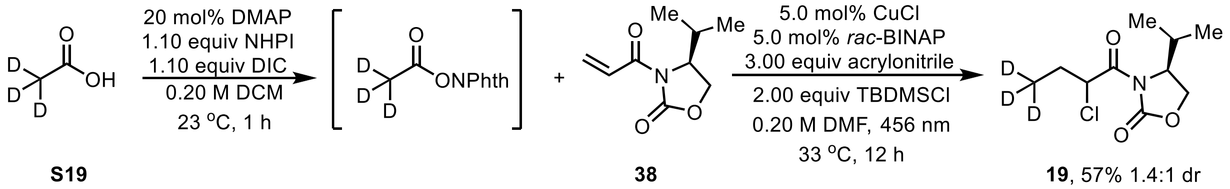

S19 **38** **19**, 57% 1.4:1 dr

Under an ambient atmosphere, to a 4-mL borosilicate vial equipped with a Teflon-coated magnetic stir bar were added acetic-2,2,2-d$_3$ acid **S19** (41.6 mg, 0.20 mmol, 1.00 equiv), *N*-hydroxyphthalimide (NHPI) (35.8 mg, 0.220 mmol, 1.10 equiv), 4-dimethylamino-pyridine (DMAP) (4.90 mg, 40 µmol, 20 mol%), *N*,*N*'-diisopropylcarbodiimide (DIC) (27.7 mg, 0.220 mmol, 1.10 equiv), and dichloromethane (DCM) (1.0 mL, c = 0.20 M). After stirring the mixture at 23 °C for 1 h, the solvent was removed under reduced pressure. Without purification of the crude redox active ester, copper chloride (CuCl) (1.0 mg, 10 µmol, 5.0 mol%), *rac*-2,2'-bis-(diphenylphosphino)-1,1'-binaphthyl (*rac*-BINAP) (6.2 mg, 10 µmol, 5.0 mol%), and *tert*-butyldimethylchlorosilane (TBDMSCl) (60.3 mg, 0.400 mmol, 2.00 equiv) were dissolved in *N*,*N*-dimethylformamide (DMF) (1.0 mL, c = 0.20 M) and then acrylonitrile (40.2 µL , 31.9 mg, 0.600 mmol, 3.00 equiv) was added. Roughly 100 mL of argon was bubbled through the reaction mixture using a Schlenk line for 30 seconds. The reaction mixture was irradiated with blue LEDs (456 nm, 2 × 40 W) for 12 h at 33 °C. Following irradiation, ethyl acetate (30 mL) and brine (30 mL) were added. The mixture was extracted once with ethyl acetate (30 mL), and the organic layer was washed with brine (30 mL), dried over Na_2SO_4 and concentrated under reduced pressure. The diastereomeric ratio of the crude product was determined using ^{1}H NMR spectroscopy. The resulting residue was purified by flash column chromatography on silica gel (hexanes/EtOAc 2:1) to afford **19** as colorless solid (30.0 mg, 50%).

R_f = 0.22 (hexanes/EtOAc 2:1).

NMR Spectroscopy:

^{1}H NMR (500 MHz, $CDCl_3$, 23 °C, δ): 5.58 (dd, J = 7.9, 5.5 Hz, 1H), 4.48 (ddd, J = 8.3, 4.0, 2.9 Hz, 1H), 4.34 (dd, J = 9.2, 8.3 Hz, 1H), 4.25 (dd, J = 9.2, 2.9 Hz, 1H), 2.37 (pd, J = 7.0, 4.0 Hz, 1H), 2.11 (dd, J = 14.2, 5.6 Hz, 1H), 0.91 (dd, J = 24.8, 6.9 Hz, 6H).

^{13}C NMR (125 MHz, $CDCl_3$, 23 °C, δ): 169.2, 153.4, 63.9, 59.1, 57.2, 28.6, 28.0, 18.0, 14.9. The determination of the carbon signal for CD_3 in the NMR spectrum was not successful due to its very high multiplicity, making it difficult to distinguish clearly.

HRMS-ESIpos (m/z) calc'd for $C_{10}H_{13}D_3ClNO_3Na$ $[M+Na]^+$, 259.0899; found, 259.0899; deviation: +0.2 ppm.

rac-Oxoacridine-derived α-chloronitrile 20

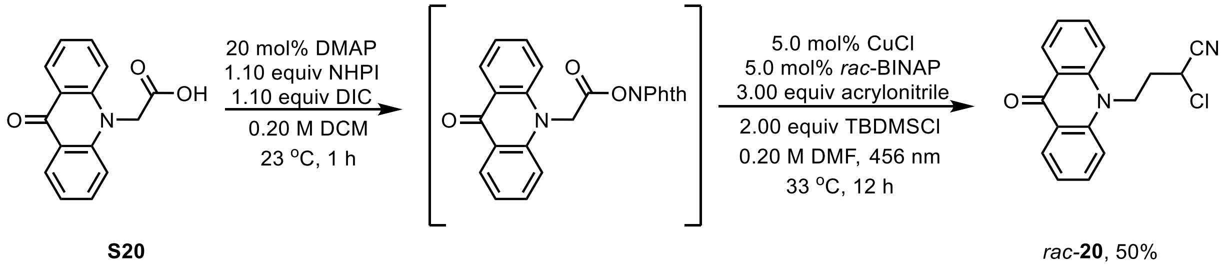

Under an ambient atmosphere, to a 4-mL borosilicate vial equipped with a Teflon-coated magnetic stir bar were added 2-(9-oxoacridin-10(9H)-yl)acetic acid **S20** (50.6 mg, 0.20 mmol, 1.00 equiv), *N*-hydroxyphthalimide (NHPI) (35.8 mg, 0.220 mmol, 1.10 equiv), 4-dimethylamino-pyridine (DMAP) (4.90 mg, 40 µmol, 20 mol%), *N*,*N*'-diisopropylcarbodiimide (DIC) (27.7 mg, 0.220 mmol, 1.10 equiv), and dichloromethane (DCM) (1.0 mL, c = 0.20 M). After stirring the mixture at 23 °C for 1 h, the solvent was removed under reduced pressure. Without purification of the crude redox active ester, copper chloride (CuCl) (1.0 mg, 10 µmol, 5.0 mol%), *rac*-2,2'-bis-(diphenylphosphino)-1,1'-binaphthyl (*rac*-BINAP) (6.2 mg, 10 µmol, 5.0 mol%), and *tert*-butyldimethylchlorosilane (TBDMSCl) (60.3 mg, 0.400 mmol, 2.00 equiv) were dissolved in *N*,*N*-dimethylformamide (DMF) (1.0 mL, c = 0.20 M) and then acrylonitrile (40.2 µL , 31.9 mg, 0.600 mmol, 3.00 equiv) was added. Roughly 100 mL of argon was bubbled through the reaction mixture using a Schlenk line for 30 seconds. The reaction mixture was irradiated with blue LEDs (456 nm, 2 × 40 W) for 12 h at 33 °C. Following irradiation, ethyl acetate (30 mL) and brine (30 mL) were added. The mixture was extracted once with ethyl acetate (30 mL), and the organic layer was washed with brine (30 mL), dried over Na_2SO_4 and concentrated under reduced pressure. The resulting residue was purified by flash column chromatography on silica gel (hexanes/EtOAc 2:1) to afford *rac*-**20** as colorless solid (30.0 mg, 50%).

R_f = 0.22 (hexanes/EtOAc 2:1).

NMR Spectroscopy:

^{1}H NMR (500 MHz, $CDCl_3$, 23 °C, δ): 8.58 (dd, *J* = 8.0, 1.8 Hz, 2H), 7.76 (ddd, *J* = 8.7, 7.0, 1.8 Hz, 2H), 7.51 (d, *J* = 8.7 Hz, 2H), 7.33 (t, *J* = 7.5 Hz, 2H), 4.80 (t, *J* = 5.7 Hz, 1H), 4.73 (ddd, *J* = 8.2, 6.9, 5.3 Hz, 2H), 2.71 – 2.62 (m, 2H).

^{13}C NMR (125 MHz, $CDCl_3$, 23 °C, δ): 177.9, 141.5, 134.6, 128.5, 122.8, 122.0, 116.1, 113.7, 41.5, 40.3, 32.7.

HRMS-EI (m/z) calc'd for $C_{17}H_{13}ClN_2O$ $[M]^+$, 296.0710; found, 296.0713; deviation: +0.9 ppm.

rac-Tryptophan-derived α-chloronitrile 21

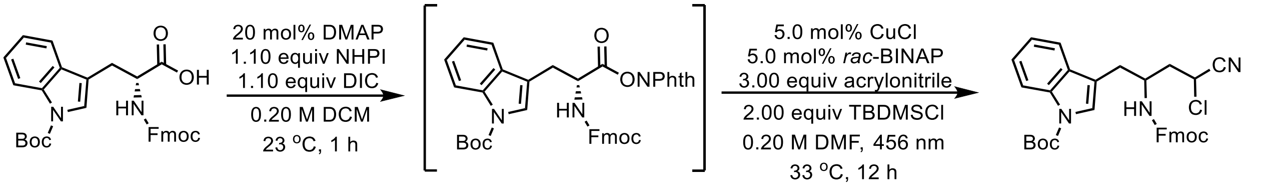

S21 *rac*-**21**, 65%

Under an ambient atmosphere, to a 4-mL borosilicate vial equipped with a Teflon-coated magnetic stir bar were added Fmoc-D-Trp(Boc)-OH **S21** (105 mg, 0.200 mmol, 1.00 equiv), *N*-hydroxyphthalimide (NHPI) (35.8 mg, 0.220 mmol, 1.10 equiv), 4-dimethylamino-pyridine (DMAP) (4.9 mg, 40 µmol, 20 mol%), *N*,*N*'-diisopropylcarbodiimide (DIC) (27.7 mg, 0.220 mmol, 1.10 equiv), and dichloromethane (DCM) (1.0 mL, c = 0.20 M). After stirring the mixture at 23 °C for 1 h, the solvent was removed under reduced pressure. Without purification of the crude redox active ester, copper chloride (CuCl) (1.0 mg, 10 µmol, 5.0 mol%), *rac*-2,2'-bis-(diphenylphosphino)-1,1'-binaphthyl (*rac*-BINAP) (6.2 mg, 10 µmol, 5.0 mol%), and *tert*-butyldimethylchlorosilane (TBDMSCl) (60.3 mg, 0.400 mmol, 2.00 equiv) were dissolved in *N*,*N*-dimethylformamide (DMF) (1.0 mL, c = 0.20 M) and then acrylonitrile (40.2 µL , 31.9 mg, 0.600 mmol, 3.00 equiv) was added. Roughly 100 mL of argon was bubbled through the reaction mixture using a Schlenk line for 30 seconds. The reaction mixture was irradiated with blue LEDs (456 nm, 2 × 40 W) for 12 h at 33 °C. Following irradiation, ethyl acetate (30 mL) and brine (30 mL) were added. The mixture was extracted once with ethyl acetate (30 mL), and the organic layer was washed with brine (30 mL), dried over Na_2SO_4 and concentrated under reduced pressure. The determination of the diastereomeric ratio of the crude product by NMR or HPLC spectroscopy was unsuccessful. The resulting residue was purified by flash column chromatography on silica gel (hexanes/EtOAc 10:1 then 5:1) to afford *rac*-**21** as yellow solid (74.7 mg, 65%).

R_f = 0.15 (hexanes/EtOAc 10:1 then 5:1).

NMR Spectroscopy:

^{1}H NMR (500 MHz, DMSO, 23 °C, δ): 8.12 (d, *J* = 8.4 Hz, 1H), 7.90 (d, *J* = 7.8 Hz, 2H), 7.73 (d, *J* = 7.6 Hz, 2H), 7.66 (d, *J* = 7.8 Hz, 1H), 7.54 (d, *J* = 7.7 Hz, 1H), 7.43 (t, *J* = 7.6 Hz, 2H), 7.37 (q, *J* = 8.5 Hz, 2H), 7.28 (t, *J* = 7.5 Hz, 1H), 4.77 (d, *J* = 5.3 Hz, 1H), 4.41 (dd, *J* = 6.9, 2.4 Hz, 2H), 4.26 (t, *J* = 6.9 Hz, 1H), 4.02 (dt, *J* = 14.0, 7.0 Hz, 1H), 3.08 (dd, *J* = 16.3, 5.5 Hz, 1H), 2.59 – 2.52 (m, 1H), 2.32 (d, *J* = 13.6 Hz, 1H), 2.09 (td, *J* = 12.9, 5.6 Hz, 1H), 1.67 (s, 9H).

^{13}C NMR (125 MHz, DMSO, 23 °C, δ): 155.5, 149.1, 143.8, 140.7, 135.6, 127.7, 127.6, 127.0, 126.5, 125.3, 125.1, 123.0, 120.3, 120.1, 118.8, 117.7, 115.3, 84.9, 65.3, 46.7, 44.2, 33.3, 27.5, 27.2, 26.6, 20.7, 14.0.

HRMS-ESIneg (m/z) calc'd for $C_{17}H_{13}ClN_2O$ [M–H]$^-$, 568.2008; found, 568.2017; deviation: +1.5 ppm.

rac-Dipeptide-derived α-chloronitrile 22

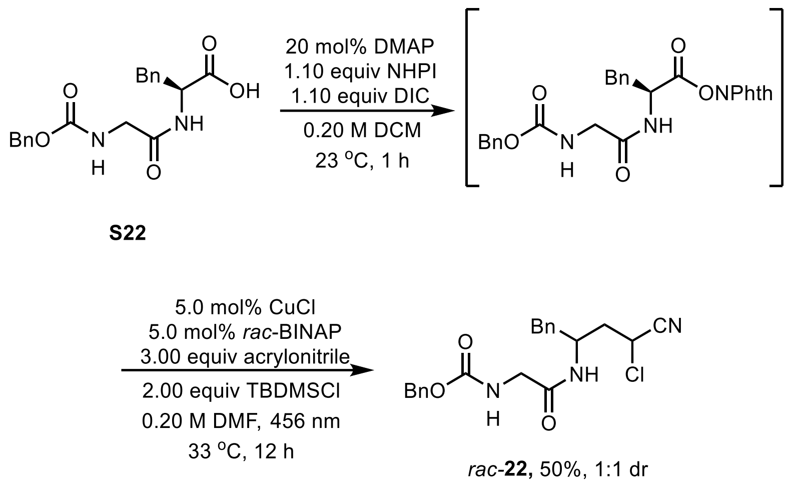

S22

rac-**22**, 50%, 1:1 dr

Under an ambient atmosphere, to a 4-mL borosilicate vial equipped with a Teflon-coated magnetic stir bar were added Z-Gly-Phe-OH **S22** (71.3 mg, 0.20 mmol, 1.00 equiv), *N*-hydroxyphthalimide (NHPI) (35.8 mg, 0.220 mmol, 1.10 equiv), 4-dimethylamino-pyridine (DMAP) (4.90 mg, 40 µmol, 20 mol%), *N*,*N*'-diisopropylcarbodiimide (DIC) (27.7 mg, 0.220 mmol, 1.10 equiv), and dichloromethane (DCM) (1.0 mL, c = 0.20 M). After stirring the mixture at 23 °C for 1 h, the solvent was removed under reduced pressure. Without purification of the crude redox active ester, copper chloride (CuCl) (1.0 mg, 10 µmol, 5.0 mol%), *rac*-2,2'-bis-(diphenylphosphino)-1,1'-binaphthyl (*rac*-BINAP) (6.2 mg, 10 µmol, 5.0 mol%), and *tert*-butyldimethylchlorosilane (TBDMSCl) (60.3 mg, 0.400 mmol, 2.00 equiv) were dissolved in *N*,*N*-dimethylformamide (DMF) (1.0 mL, c = 0.20 M) and then acrylonitrile (40.2 µL , 31.9 mg, 0.600 mmol, 3.00 equiv) was added. Roughly 100 mL of argon was bubbled through the reaction mixture using a Schlenk line for 30 seconds. The reaction mixture was irradiated with blue LEDs (456 nm, 2 × 40 W) for 12 h at 33 °C. Following irradiation, ethyl acetate (30 mL) and brine (30 mL) were added. The mixture was extracted once with ethyl acetate (30 mL), and the organic layer was washed with brine (30 mL), dried over Na_2SO_4 and concentrated under reduced pressure. The diastereomeric ratio of the crude product was determined using HPLC spectroscopy. The resulting residue was purified by flash column chromatography on silica gel (hexanes/EtOAc 1:1) to afford *rac*-**22** as colorless solid (40.0 mg, 50%).

$\mathbf{R}_f$ = 0.28 (hexanes/EtOAc 1:1).

NMR Spectroscopy:

^{1}H NMR (500 MHz, DMSO, 23 °C, δ): 7.92 (dd, *J* = 52.5, 8.6 Hz, 1H), 7.54 – 7.25 (m, 7H), 7.24 – 7.07 (m, 3H), 5.09 (dd, *J* = 9.6, 5.2 Hz, 1H), 5.04 (s, 2H), 4.12 (d, *J* = 8.6 Hz, 1H), 3.57 (qt, *J* = 16.4, 5.7 Hz, 2H), 2.76 (dd, *J* = 14.3, 7.8 Hz, 2H), 2.29 – 2.06 (m, 2H).

^{13}C NMR (125 MHz, DMSO, 23 °C, δ): 169.0, 156.4, 137.8, 137.8, 137.0, 129.2, 129.1, 128.3, 128.3, 127.8, 127.7, 126.3, 126.3, 118.1, 65.5, 47.6, 46.8, 43.7, 40.6, 23.2.

HRMS-ESIpos (m/z) calc'd for $C_{21}H_{22}ClN_3O_3Na$ $[M+Na]^+$, 422.1241; found, 422.1240; deviation: –0.3 ppm.

HPLC (Nucleodur C18 Gravity, Methanol /Water 55% - 12' isocratic then in 1' to 95%, 308 K, 220 nm): t_R (major) = 7.2 min, t_R (minor) = 7.6 min, dr = 1:1

rac-Linolenic-derived α-chloronitrile 23

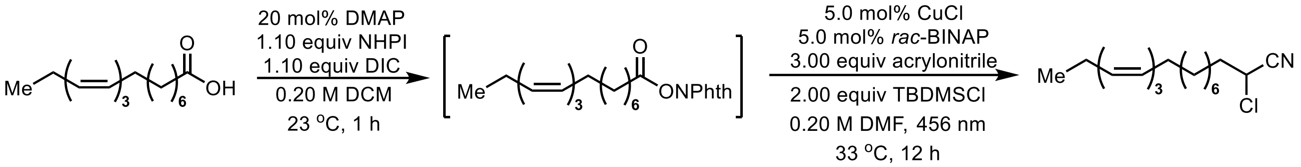

S23 *rac*-**23**, 70%

Under an ambient atmosphere, to a 4-mL borosilicate vial equipped with a Teflon-coated magnetic stir bar were added linolenic acid **S23** (39.0 mg, 0.20 mmol, 1.00 equiv), *N*-hydroxyphthalimide (NHPI) (35.8 mg, 0.220 mmol, 1.10 equiv), 4-dimethylamino-pyridine (DMAP) (4.90 mg, 40 µmol, 20 mol%), *N*,*N*'-diisopropylcarbodiimide (DIC) (27.7 mg, 0.220 mmol, 1.10 equiv), and dichloromethane (DCM) (1.0 mL, c = 0.20 M). After stirring the mixture at 23 °C for 1 h, the solvent was removed under reduced pressure. Without purification of the crude redox active ester, copper chloride (CuCl) (1.0 mg, 10 µmol, 5.0 mol%), *rac*-2,2'-bis-(diphenylphosphino)-1,1'-binaphthyl (*rac*-BINAP) (6.2 mg, 10 µmol, 5.0 mol%), and *tert*-butyldimethylchlorosilane (TBDMSCl) (60.3 mg, 0.400 mmol, 2.00 equiv) were dissolved in *N*,*N*-dimethylformamide (DMF) (1.0 mL, c = 0.20 M) and then acrylonitrile (40.2 µL , 31.9 mg, 0.600 mmol, 3.00 equiv) was added. Roughly 100 mL of argon was bubbled through the reaction mixture using a Schlenk line for 30 seconds. The reaction mixture was irradiated with blue LEDs (456 nm, 2 × 40 W) for 12 h at 33 °C. Following irradiation, ethyl acetate (30 mL) and brine (30 mL) were added. The mixture was extracted once with ethyl acetate (30 mL), and the organic layer was washed with brine (30 mL), dried over Na_2SO_4 and concentrated under reduced pressure. The resulting residue was purified by flash column chromatography on silica gel (pentane/Et_2O 5:1) to afford *rac*-**23** as colorless oil (31.6 mg, 70%).

R_f = 0.62 (pentane/Et_2O 5:1).

NMR Spectroscopy:

^{1}H NMR (500 MHz, $CDCl_3$, 23 °C, δ): 5.53 – 5.19 (m, 6H), 4.43 (t, *J* = 6.8 Hz, 1H), 2.81 (t, *J* = 6.1 Hz, 4H), 2.15 – 1.98 (m, 4H), 1.61 – 1.52 (m, 2H), 1.41 – 1.28 (m, 12H), 0.98 (t, *J* = 7.5 Hz, 3H).

^{13}C NMR (125 MHz, $CDCl_3$, 23 °C, δ): 132.0, 130.3, 128.4, 128.3, 127.8, 127.2, 117.2, 42.6, 36.4, 29.7, 29.4, 29.3, 29.2, 28.6, 27.3, 25.8, 25.7, 25.6, 20.6, 14.4.

HRMS-EI (m/z) calc'd for $C_{20}H_{32}ClN$ $[M]^+$, 321.2217; found, 321.2219; deviation: +0.4 ppm.

rac-Fenbufen-derived α-chloronitrile 24

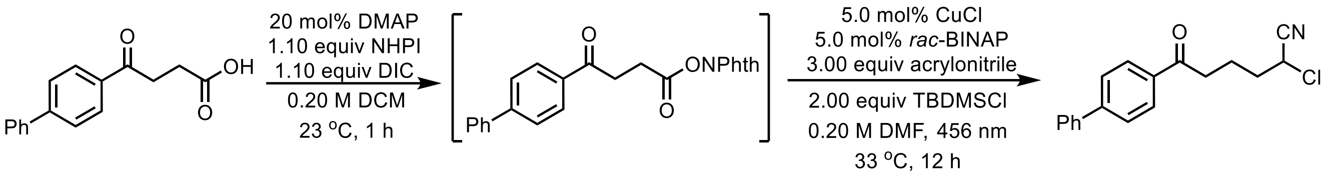

S24 *rac*-**24**, 59%

Under an ambient atmosphere, to a 4-mL borosilicate vial equipped with a Teflon-coated magnetic stir bar were added fenbufen acid **S24** (50.8 mg, 0.20 mmol, 1.00 equiv), *N*-hydroxyphthalimide (NHPI) (35.8 mg, 0.220 mmol, 1.10 equiv), 4-dimethylamino-pyridine (DMAP) (4.90 mg, 40 µmol, 20 mol%), *N*,*N*'-diisopropylcarbodiimide (DIC) (27.7 mg, 0.220 mmol, 1.10 equiv), and dichloromethane (DCM) (1.0 mL, c = 0.20 M). After stirring the mixture at 23 °C for 1 h, the solvent was removed under reduced pressure. Without purification of the crude redox active ester, copper chloride (CuCl) (1.0 mg, 10 µmol, 5.0 mol%), *rac*-2,2'-bis-(diphenylphosphino)-1,1'-binaphthyl (*rac*-BINAP) (6.2 mg, 10 µmol, 5.0 mol%), and *tert*-butyldimethylchlorosilane (TBDMSCl) (60.3 mg, 0.400 mmol, 2.00 equiv) were dissolved in *N*,*N*-dimethylformamide (DMF) (1.0 mL, c = 0.20 M) and then acrylonitrile (40.2 µL , 31.9 mg, 0.600 mmol, 3.00 equiv) was added. Roughly 100 mL of argon was bubbled through the reaction mixture using a Schlenk line for 30 seconds. The reaction mixture was irradiated with blue LEDs (456 nm, 2 × 40 W) for 12 h at 33 °C. Following irradiation, ethyl acetate (30 mL) and brine (30 mL) were added. The mixture was extracted once with ethyl acetate (30 mL), and the organic layer was washed with brine (30 mL), dried over Na_2SO_4 and concentrated under reduced pressure. The resulting residue was purified by flash column chromatography on silica gel (hexanes/EtOAc 10:1) to afford *rac*-**24** as colorless solid (35.2 mg, 59%).

R_f = 0.33 (hexanes/EtOAc 10:1).

NMR Spectroscopy:

^{1}H NMR (500 MHz, $CDCl_3$, 23 °C, δ): 8.03 (d, *J* = 8.4 Hz, 2H), 7.70 (d, *J* = 8.5 Hz, 2H), 7.65 – 7.61 (m, 2H), 7.48 (dd, *J* = 8.3, 6.7 Hz, 2H), 7.43 – 7.39 (m, 2H), 4.54 (t, *J* = 6.7 Hz, 1H), 3.12 (t, *J* = 6.8 Hz, 2H), 2.27 – 2.11 (m, 2H), 2.11 – 1.97 (m, 2H).

^{13}C NMR (125 MHz, $CDCl_3$, 23 °C, δ): 198.2, 146.1, 139.8, 135.3, 129.1, 128.6, 128.4, 127.4, 127.3, 117.0, 42.5, 37.0, 35.7, 20.3.

HRMS-EI (m/z) calc'd for $C_{18}H_{16}ClNO$ $[M]^+$, 297.0914; found, 297.0915; deviation: +0.3 ppm.

rac-Fenofibric acid-derived α-chloronitrile 25

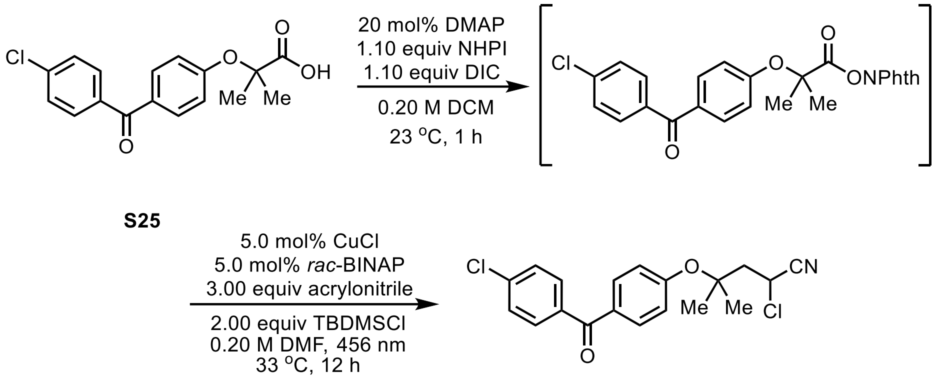

S25

rac-**25**, 44%

Under an ambient atmosphere, to a 4-mL borosilicate vial equipped with a Teflon-coated magnetic stir bar were added fenofibric acid **S25** (63.6 mg, 0.200 mmol, 1.00 equiv), *N*-hydroxyphthalimide (NHPI) (35.8 mg, 0.220 mmol, 1.10 equiv), 4-dimethylamino-pyridine (DMAP) (4.90 mg, 40 µmol, 20 mol%), *N*,*N*'-diisopropylcarbodiimide (DIC) (27.7 mg, 0.220 mmol, 1.10 equiv), and dichloromethane (DCM) (1.0 mL, c = 0.20 M). After stirring the mixture at 23 °C for 1 h, the solvent was removed under reduced pressure. Without purification of the crude redox active ester, copper chloride (CuCl) (1.0 mg, 10 µmol, 5.0 mol%), *rac*-2,2'-bis-(diphenylphosphino)-1,1'-binaphthyl (*rac*-BINAP) (6.2 mg, 10 µmol, 5.0 mol%), and *tert*-butyldimethylchlorosilane (TBDMSCl) (60.3 mg, 0.400 mmol, 2.00 equiv) were dissolved in *N*,*N*-dimethylformamide (DMF) (1.0 mL, c = 0.20 M) and then acrylonitrile (40.2 µL , 31.9 mg, 0.600 mmol, 3.00 equiv) was added. Roughly 100 mL of argon was bubbled through the reaction mixture using a Schlenk line for 30 seconds. The reaction mixture was irradiated with blue LEDs (456 nm, 2 × 40 W) for 12 h at 33 °C. Following irradiation, ethyl acetate (30 mL) and brine (30 mL) were added. The mixture was extracted once with ethyl acetate (30 mL), and the organic layer was washed with brine (30 mL), dried over Na_2SO_4 and concentrated under reduced pressure. The resulting residue was purified by flash column chromatography on silica gel (hexanes/EtOAc 5:1) to afford *rac*-**25** as colorless solid (31.7 mg, 44%).

R_f = 0.27 (hexanes/EtOAc 5:1).

NMR Spectroscopy:

^{1}H NMR (500 MHz, $CDCl_3$, 23 °C, δ): 7.78 (d, *J* = 8.7 Hz, 2H), 7.75 (d, *J* = 8.5 Hz, 2H), 7.49 (d, *J* = 8.5 Hz, 2H), 7.10 (d, *J* = 8.6 Hz, 2H), 4.91 (dd, *J* = 8.7, 4.9 Hz, 1H), 2.67 (dd, *J* = 14.6, 8.8 Hz, 1H), 2.47 (dd, *J* = 14.6, 5.0 Hz, 1H), 1.53 (s, 3H), 1.48 (s, 3H).

^{13}C NMR (125 MHz, $CDCl_3$, 23 °C, δ): 194.3, 158.4, 138.7, 136.0, 132.6, 131.7, 131.3, 128.6, 122.6, 117.8, 79.4, 49.5, 38.1, 26.7, 26.7.

HRMS-ESIpos (m/z) calc'd for $C_{19}H_{17}Cl_2NO_2Na$ $[M+Na]^+$, 384.0529; found, 384.0530; deviation: –0.5 ppm.

General procedure

Alkene Scope

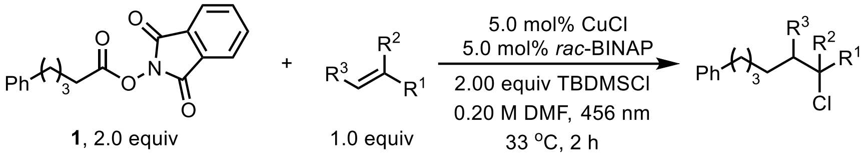

Under an ambient atmosphere, to a 4-mL borosilicate vial equipped with a Teflon-coated magnetic stir bar were added 1,3-dioxoisoindolin-2-yl 5-phenylpentanoate **1** (129 mg, 0.400 mmol, 2.00 equiv), copper chloride (CuCl) (1.0 mg, 10 µmol, 5.0 mol%), *rac*-2,2'-bis-(diphenylphosphino)-1,1'-binaphthyl (*rac*-BINAP) (6.2 mg, 10 µmol, 5.0 mol%), and *tert*-butyldimethylchlorosilane (TBDMSCl) (60.3 mg, 0.400 mmol, 2.00 equiv) were dissolved in *N*,*N*-dimethylformamide (DMF) (1.0 mL, c = 0.20 M) and then alkene (0.200 mmol, 1.00 equiv) was added. The vial was evacuated and the solvent was bubbled with inert gas using a Schlenk line under 0.50 mbar pressure for 30 seconds. The reaction mixture was irradiated with blue LEDs (456 nm, 2 × 40 W) at 33 °C for 2 h. Following irradiation, ethyl acetate (30 mL) and brine (30 mL) were added. The mixture was extracted once with ethyl acetate (30 mL), and the organic layer was washed with brine (30 mL), dried over Na_2SO_4 and concentrated under reduced pressure. The diastereomeric ratio of the crude product was determined using NMR or HPLC spectroscopy. The resulting residue was purified by flash column chromatography on silica gel to afford the desired product.

rac-2-Chloro-7-phenylheptanal 26

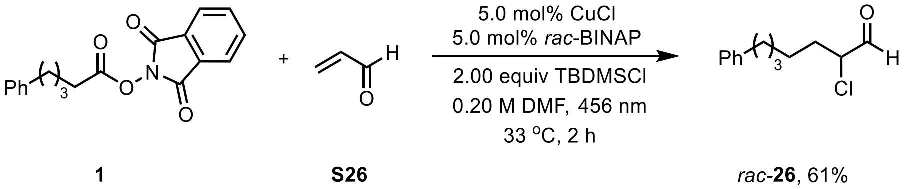

Under an ambient atmosphere, to a 4-mL borosilicate vial equipped with a Teflon-coated magnetic stir bar were added 1,3-dioxoisoindolin-2-yl 5-phenylpentanoate **1** (129 mg, 0.400 mmol, 2.00 equiv), copper chloride (CuCl) (1.0 mg, 10 µmol, 5.0 mol%), *rac*-2,2'-bis-(diphenylphosphino)-1,1'-binaphthyl (*rac*-BINAP) (6.2 mg, 10 µmol, 5.0 mol%), and *tert*-butyldimethylchlorosilane (TBDMSCl) (60.3 mg, 0.400 mmol, 2.00 equiv) were dissolved in *N*,*N*-dimethylformamide (DMF) (1.0 mL, c = 0.20 M) and then acrylaldehyde **S26** (11.2 mg, 13.4 µL, 0.200 mmol, 1.00 equiv) was added. The vial was evacuated and the solvent was bubbled with inert gas using a Schlenk line under 0.50 mbar pressure for 30 seconds. The reaction mixture was irradiated with blue LEDs (456 nm, 2 × 40 W) at 33 °C for 2 h. Following irradiation, ethyl acetate (30 mL) and brine (30 mL) were added. The mixture was extracted once with ethyl acetate (30 mL), and the organic layer was washed with brine (30 mL), dried over Na_2SO_4 and concentrated under reduced pressure. The resulting residue was purified by flash column chromatography on silica gel (Pentane/EtOAc 10:1) to afford *rac*-**26** as pale yellow oil (29.1 mg, 61%).

R*f* = 0.50 (Pentane/EtOAc 10:1).

NMR Spectroscopy:

^{1}H NMR (500 MHz, $CDCl_3$, 23 °C, δ): 9.49 (d, *J* = 2.3 Hz, 1H), 7.29 (dd, *J* = 8.3, 6.8 Hz, 2H), 7.22 – 7.13 (m, 3H), 4.15 (ddd, *J* = 8.1, 5.4, 2.4 Hz, 1H), 2.62 (t, *J* = 7.7 Hz, 2H), 2.07 – 1.77 (m, 2H), 1.65 (p, *J* = 7.6 Hz, 2H), 1.58 – 1.48 (m, 2H), 1.42 – 1.35 (m, 2H).

^{13}C NMR (125 MHz, $CDCl_3$, 23 °C, δ): 195.4, 142.4, 128.5, 128.4, 125.8, 64.0, 35.8, 32.0, 31.1, 28.6, 25.5.

HRMS-EI (m/z) calc'd for $C_{13}H_{17}ClO$ $[M]^+$, 224.0962; found, 224.0960; deviation: –0.8 ppm.

rac-4-Chloro-8-phenyloctan-2-one 27

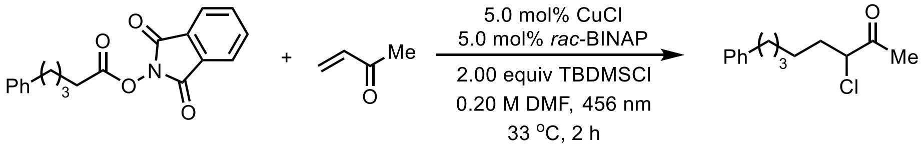

1 **S27** *rac*-**27**, 61%

Under an ambient atmosphere, to a 4-mL borosilicate vial equipped with a Teflon-coated magnetic stir bar were added 1,3-dioxoisoindolin-2-yl 5-phenylpentanoate **1** (129 mg, 0.400 mmol, 2.00 equiv), copper chloride (CuCl) (1.0 mg, 10 μmol, 5.0 mol%), *rac*-2,2'-bis-(diphenylphosphino)-1,1'-binaphthyl (*rac*-BINAP) (6.2 mg, 10 μmol, 5.0 mol%), and *tert*-butyldimethylchlorosilane (TBDMSCl) (60.3 mg, 0.400 mmol, 2.00 equiv) were dissolved in *N*,*N*-dimethylformamide (DMF) (1.0 mL, c = 0.20 M) and then but-3-en-2-one **S27** (14.0 mg, 17.0 μL, 0.200 mmol, 1.00 equiv) was added. The vial was evacuated and the solvent was bubbled with inert gas using a Schlenk line under 0.50 mbar pressure for 30 seconds. The reaction mixture was irradiated with blue LEDs (456 nm, 2 × 40 W) at 33 °C for 2 h. Following irradiation, ethyl acetate (30 mL) and brine (30 mL) were added. The mixture was extracted once with ethyl acetate (30 mL), and the organic layer was washed with brine (30 mL), dried over Na_2SO_4 and concentrated under reduced pressure. The resulting residue was purified by flash column chromatography on silica gel (Pentane/EtOAc 10:1) to afford *rac*-**27** as colorless oil (29.1 mg, 61%).

R*f* = 0.43 (Pentane/EtOAc 10:1).

NMR Spectroscopy:

^{1}H NMR (500 MHz, $CDCl_3$, 23 °C, δ): 7.32 – 7.24 (m, 2H), 7.19 (td, *J* = 7.4, 1.5 Hz, 3H), 4.17 (dd, *J* = 8.5, 5.5 Hz, 1H), 2.62 (t, 2H), 2.31 (s, 3H), 2.00 – 1.77 (m, 2H), 1.65 (p, *J* = 6.5 Hz, 2H), 1.58 – 1.48 (m, 1H), 1.47 – 1.31 (m, 3H).

^{13}C NMR (125 MHz, $CDCl_3$, 23 °C, δ): 203.5, 142.5, 128.4, 128.4, 125.8, 64.3, 35.8, 33.7, 31.2, 28.6, 26.0, 25.9.

HRMS-EI (m/z) calc'd for $C_{14}H_{19}ClO$ $[M]^+$, 238.1118; found, 238.1119; deviation: +0.0 ppm.

rac-tert-Butyl 2-chloro-7-phenylheptanoate 28

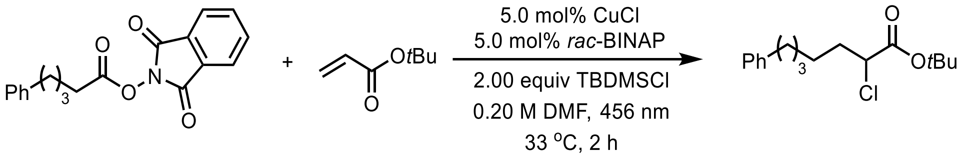

1 S28 *rac*-**28**, 46%

Under an ambient atmosphere, to a 4-mL borosilicate vial equipped with a Teflon-coated magnetic stir bar were added 1,3-dioxoisoindolin-2-yl 5-phenylpentanoate **1** (129 mg, 0.400 mmol, 2.00 equiv), copper chloride (CuCl) (1.0 mg, 10 µmol, 5.0 mol%), *rac*-2,2'-bis-(diphenylphosphino)-1,1'-binaphthyl (*rac*-BINAP) (6.2 mg, 10 µmol, 5.0 mol%), and *tert*-butyldimethylchlorosilane (TBDMSCl) (60.3 mg, 0.400 mmol, 2.00 equiv) were dissolved in *N*,*N*-dimethylformamide (DMF) (1.0 mL, c = 0.20 M) and then *tert*-butyl acrylate **S28** (25.6 mg, 29.3 µL, 0.200 mmol, 1.00 equiv) was added. The vial was evacuated and the solvent was bubbled with inert gas using a Schlenk line under 0.50 mbar pressure for 30 seconds. The reaction mixture was irradiated with blue LEDs (456 nm, 2 × 40 W) at 33 °C for 2 h. Following irradiation, ethyl acetate (30 mL) and brine (30 mL) were added. The mixture was extracted once with ethyl acetate (30 mL), and the organic layer was washed with brine (30 mL), dried over Na_2SO_4 and concentrated under reduced pressure. The resulting residue was purified by flash column chromatography on silica gel (Pentane/Et_2O 20:1) to afford *rac*-**28** as colorless oil (27.1 mg, 46%).

R_f = 0.47 (Pentane/Et_2O 20:1).

NMR Spectroscopy:

^{1}H NMR (500 MHz, $CDCl_3$, 23 °C, δ): 7.32 – 7.25 (m, 2H), 7.21 – 7.10 (m, 3H), 4.16 (dd, *J* = 7.9, 6.1 Hz, 1H), 2.63 (t, 2H), 2.04 – 1.84 (m, 2H), 1.70 – 1.62 (m, 2H), 1.50 (s, 9H), 1.47 – 1.44 (m, 2H), 1.42 – 1.34 (m, 2H).

^{13}C NMR (125 MHz, $CDCl_3$, 23 °C, δ): 168.9, 142.5, 128.4, 128.3, 125.7, 82.5, 58.6, 35.8, 34.9, 31.2, 28.6, 27.9, 25.9.

HRMS-EI (m/z) calc'd for $C_{17}H_{25}ClO_2$ $[M]^+$, 296.1537; found, 296.1538; deviation: +0.1 ppm.

rac-2-Chloro-*N,N*-dimethyl-7-phenylheptanamide 29

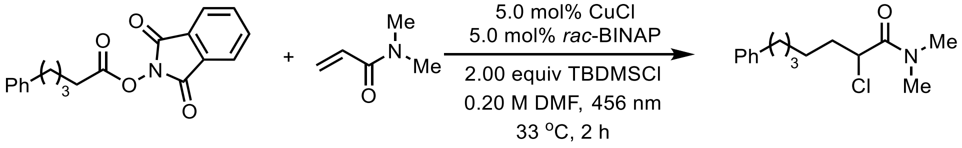

1 S29 *rac*-**29**, 69%

Under an ambient atmosphere, to a 4-mL borosilicate vial equipped with a Teflon-coated magnetic stir bar were added 1,3-dioxoisoindolin-2-yl 5-phenylpentanoate **1** (129 mg, 0.400 mmol, 2.00 equiv), copper chloride (CuCl) (1.0 mg, 10 µmol, 5.0 mol%), *rac*-2,2'-bis-(diphenylphosphino)-1,1'-binaphthyl (*rac*-BINAP) (6.2 mg, 10

µmol, 5.0 mol%), and *tert*-butyldimethylchlorosilane (TBDMSCl) (60.3 mg, 0.400 mmol, 2.00 equiv) were dissolved in *N*,*N*-dimethylformamide (DMF) (1.0 mL, c = 0.20 M) and then *N,N*-dimethylacrylamide **S29** (19.8 mg, 20.6 µL, 0.200 mmol, 1.00 equiv) was added. The vial was evacuated and the solvent was bubbled with inert gas using a Schlenk line under 0.50 mbar pressure for 30 seconds. The reaction mixture was irradiated with blue LEDs (456 nm, 2 × 40 W) at 33 °C for 2 h. Following irradiation, ethyl acetate (30 mL) and brine (30 mL) were added. The mixture was extracted once with ethyl acetate (30 mL), and the organic layer was washed with brine (30 mL), dried over Na_2SO_4 and concentrated under reduced pressure. The resulting residue was purified by flash column chromatography on silica gel (hexanes/EtOAc 5:1) to afford *rac*-**29** as yellow oil (37.0 mg, 69%).

R*f* = 0.30 (hexanes/EtOAc 5:1).

NMR Spectroscopy:

^{1}H NMR (500 MHz, $CDCl_3$, 23 °C, δ): 7.31 – 7.22 (m, 2H), 7.20 – 7.13 (m, 3H), 4.39 (dd, *J* = 7.8, 6.4 Hz, 1H), 3.09 (s, 3H), 2.99 (s, 3H), 2.61 (dd, *J* = 8.6, 6.8 Hz, 2H), 2.16 – 1.84 (m, 2H), 1.71 – 1.58 (m, 2H), 1.54 – 1.47 (m, 1H), 1.43 – 1.35 (m, 3H).

^{13}C NMR (125 MHz, $CDCl_3$, 23 °C, δ): 168.7, 142.6, 128.5, 128.3, 125.7, 54.1, 37.4, 36.3, 35.8, 34.4, 31.3, 28.8, 26.3.

HRMS-EI (m/z) calc'd for $C_{15}H_{22}ClNO$ $[M]^+$, 267.1384; found, 267.1385; deviation: +0.3 ppm.

rac-(1-Chlorohexane-1,6-diyl)dibenzene 30

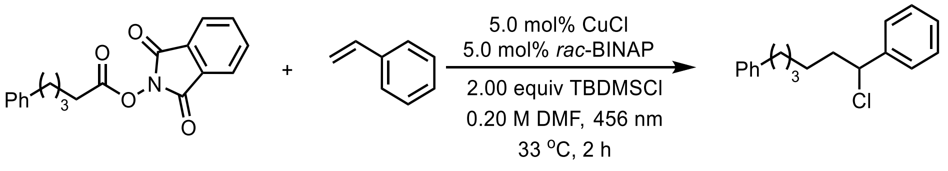

1 **S30** *rac*-**30**, 83%

Under an ambient atmosphere, to a 4-mL borosilicate vial equipped with a Teflon-coated magnetic stir bar were added 1,3-dioxoisoindolin-2-yl 5-phenylpentanoate **1** (129 mg, 0.400 mmol, 2.00 equiv), copper chloride (CuCl) (1.0 mg, 10 µmol, 5.0 mol%), *rac*-2,2'-bis-(diphenylphosphino)-1,1'-binaphthyl (*rac*-BINAP) (6.2 mg, 10 µmol, 5.0 mol%), and *tert*-butyldimethylchlorosilane (TBDMSCl) (60.3 mg, 0.400 mmol, 2.00 equiv) were dissolved in *N*,*N*-dimethylformamide (DMF) (1.0 mL, c = 0.20 M) and then styrene **S30** (20.8 mg, 23.0 µL, 0.200 mmol, 1.00 equiv) was added. The vial was evacuated and the solvent was bubbled with inert gas using a Schlenk line under 0.50 mbar pressure for 30 seconds. The reaction mixture was irradiated with blue LEDs (456 nm, 2 × 40 W) at 33 °C for 2 h. Following irradiation, ethyl acetate (30 mL) and brine (30 mL) were added. The mixture was extracted once with ethyl acetate (30 mL), and the organic layer was washed with brine (30 mL), dried over Na_2SO_4 and concentrated under reduced pressure. The resulting residue was purified by flash column chromatography on silica gel (Hexanes) to afford *rac*-**30** as yellow oil (45.3 mg, 83%).

R_f = 0.30 (Hexanes).

NMR Spectroscopy:

^{1}H NMR (500 MHz, $CDCl_3$, 23 °C, δ): 7.44 – 7.37 (m, 4H), 7.36 – 7.29 (m, 3H), 7.24 – 7.17 (m, 3H), 4.88 (dd, *J* = 8.1, 6.4 Hz, 1H), 2.67 – 2.57 (m, 2H), 2.22 – 1.99 (m, 2H), 1.72 – 1.62 (m, 2H), 1.60 – 1.51 (m, 1H), 1.45 – 1.36 (m, 3H).

^{13}C NMR (125 MHz, $CDCl_3$, 23 °C, δ): 142.6, 142.0, 128.7, 128.3, 127.0, 125.7, 63.9, 40.0, 35.9, 31.3, 27.0.

HRMS-APPIpos (m/z) calc'd for $C_{18}H_{21}Cl$ $[M]^+$, 272.1326; found, 272.1326; deviation: +0.2 ppm.

rac-1-(1-Chloro-6-phenylhexyl)-4-(trifluoromethyl)benzene 31

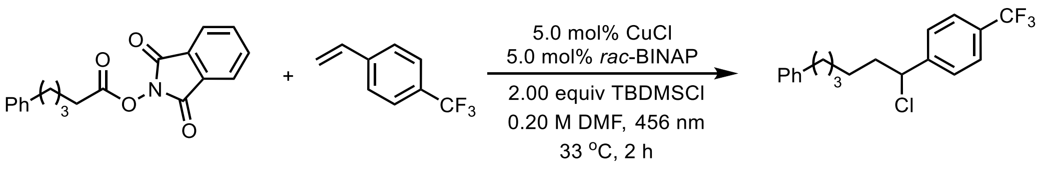

1 **S31** *rac*-**31**, 74%

Under an ambient atmosphere, to a 4-mL borosilicate vial equipped with a Teflon-coated magnetic stir bar were added 1,3-dioxoisoindolin-2-yl 5-phenylpentanoate **1** (129 mg, 0.400 mmol, 2.00 equiv), copper chloride (CuCl) (1.0 mg, 10 µmol, 5.0 mol%), *rac*-2,2'-bis-(diphenylphosphino)-1,1'-binaphthyl (*rac*-BINAP) (6.2 mg, 10 µmol, 5.0 mol%), and *tert*-butyldimethylchlorosilane (TBDMSCl) (60.3 mg, 0.400 mmol, 2.00 equiv) were dissolved in *N*,*N*-dimethylformamide (DMF) (1.0 mL, c = 0.20 M) and then 1-(trifluoromethyl)-4-vinylbenzene **S31** (34.4 mg, 29.6 µL, 0.200 mmol, 1.00 equiv) was added. The vial was evacuated and the solvent was bubbled with inert gas using a Schlenk line under 0.50 mbar pressure for 30 seconds. The reaction mixture was irradiated with blue LEDs (456 nm, 2 × 40 W) at 33 °C for 2 h. Following irradiation, ethyl acetate (30 mL) and brine (30 mL) were added. The mixture was extracted once with ethyl acetate (30 mL), and the organic layer was washed with brine (30 mL), dried over Na_2SO_4 and concentrated under reduced pressure. The resulting residue was purified by flash column chromatography on silica gel (Hexanes) to afford *rac*-**31** as colorless oil (50.3 mg, 74%).

R_f = 0.29 (Hexanes).

NMR Spectroscopy:

^{1}H NMR (500 MHz, $CDCl_3$, 23 °C, δ): 7.64 (d, *J* = 8.1 Hz, 2H), 7.50 (d, *J* = 8.1 Hz, 2H), 7.30 (dd, *J* = 8.2, 6.9 Hz, 2H), 7.20 (ddd, *J* = 14.6, 7.5, 1.3 Hz, 3H), 4.88 (dd, *J* = 8.1, 6.4 Hz, 1H), 2.75 – 2.47 (m, 2H), 2.22 – 1.94 (m, 2H), 1.71 – 1.60 (m, 2H), 1.58 – 1.49 (m, 1H), 1.46 – 1.34 (m, 3H).

^{13}C NMR (125 MHz, $CDCl_3$, 23 °C, δ): 145.9, 142.5, 130.4 (q, *J* = 32.5 Hz), 128.5, 128.4, 127.4, 125.8, 125.7 (q, *J* = 3.8 Hz), 124.1 (q, *J* = 271.9 Hz), 123.0, 62.6, 39.9, 35.8, 31.2, 28.6, 26.9.

^{19}F NMR (470 MHz, $CDCl_3$, 23 °C, δ): –62.5.

HRMS-EI (m/z) calc'd for $C_{19}H_{20}ClF_3$ $[M]^+$, 340.1200; found, 340.1200; deviation: +0.0 ppm.

rac-3-(2-Chloro-7-phenylheptanoyl)oxazolidin-2-one 32

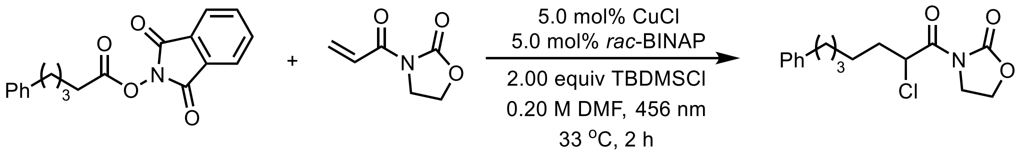

1 **S32** *rac*-**32**, 59%

Under an ambient atmosphere, to a 4-mL borosilicate vial equipped with a Teflon-coated magnetic stir bar were added 1,3-dioxoisoindolin-2-yl 5-phenylpentanoate **1** (129 mg, 0.400 mmol, 2.00 equiv), copper chloride (CuCl) (1.0 mg, 10 µmol, 5.0 mol%), *rac*-2,2'-bis-(diphenylphosphino)-1,1'-binaphthyl (*rac*-BINAP) (6.2 mg, 10 µmol, 5.0 mol%), and *tert*-butyldimethylchlorosilane (TBDMSCl) (60.3 mg, 0.400 mmol, 2.00 equiv) were dissolved in *N*,*N*-dimethylformamide (DMF) (1.0 mL, c = 0.20 M) and then 3-acryloyloxazolidin-2-one **S32** (28.2 mg, 0.200 mmol, 1.00 equiv) was added. The vial was evacuated and the solvent was bubbled with inert gas using a Schlenk line under 0.50 mbar pressure for 30 seconds. The reaction mixture was irradiated with blue LEDs (456 nm, 2 × 40 W) at 33 °C for 2 h. Following irradiation, ethyl acetate (30 mL) and brine (30 mL) were added. The mixture was extracted once with ethyl acetate (30 mL), and the organic layer was washed with brine (30 mL), dried over Na_2SO_4 and concentrated under reduced pressure. The resulting residue was purified by flash column chromatography on silica gel (hexanes/EtOAc 5:1) to afford *rac*-**32** as colorless oil (36.3 mg, 59%).

R_f = 0.26 (hexanes/EtOAc 5:1).

NMR Spectroscopy:

^{1}H NMR (500 MHz, $CDCl_3$, 23 °C, δ): 7.28 (dd, *J* = 8.7, 6.5 Hz, 2H), 7.18 (dd, *J* = 7.7, 5.7 Hz, 3H), 5.59 (dd, *J* = 8.3, 5.5 Hz, 1H), 4.52 – 4.38 (m, 2H), 4.12 – 3.97 (m, 2H), 2.61 (t, *J* = 7.7 Hz, 2H), 2.09 – 1.86 (m, 2H), 1.73 – 1.60 (m, 2H), 1.61 – 1.50 (m, 1H), 1.51 – 1.36 (m, 3H).

^{13}C NMR (125 MHz, $CDCl_3$, 23 °C, δ): 169.4, 152.8, 142.6, 128.5, 128.3, 125.7, 62.3, 55.0, 42.9, 35.8, 34.2, 31.2, 28.6, 26.0.

HRMS-EI (m/z) calc'd for $C_{16}H_{20}ClNO_3$ $[M]^+$, 309.1126; found, 309.1125; deviation: –0.3 ppm.

rac-2-(1-Chloro-6-phenylhexyl)-4,4,5,5-tetramethyl-1,3,2-dioxaborolane 33

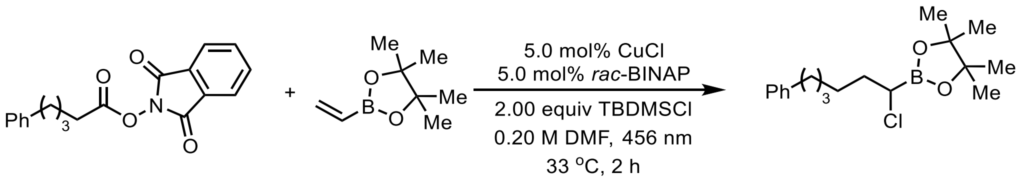

1 **S33** *rac*-**33**, 42%

Under an ambient atmosphere, to a 4-mL borosilicate vial equipped with a Teflon-coated magnetic stir bar were added 1,3-dioxoisoindolin-2-yl 5-phenylpentanoate **1** (129 mg, 0.400 mmol, 2.00 equiv), copper chloride (CuCl) (1.0 mg, 10 µmol, 5.0 mol%), *rac*-2,2'-bis-(diphenylphosphino)-1,1'-binaphthyl (*rac*-BINAP) (6.2 mg, 10 µmol, 5.0 mol%), and *tert*-butyldimethylchlorosilane (TBDMSCl) (60.3 mg, 0.400 mmol, 2.00 equiv) were dissolved in *N*,*N*-dimethylformamide (DMF) (1.0 mL, c = 0.20 M) and then 4,4,5,5-tetramethyl-2-vinyl-1,3,2-dioxaborolane **S33** (30.8 mg, 0.200 mmol, 1.00 equiv) was added. The vial was evacuated and the solvent was bubbled with inert gas using a Schlenk line under 0.50 mbar pressure for 30 seconds. The reaction mixture was irradiated with blue LEDs (456 nm, 2 × 40 W) at 33 °C for 2 h. Following irradiation, ethyl acetate (30 mL) and brine (30 mL) were added. The mixture was extracted once with ethyl acetate (30 mL), and the organic layer was washed with brine (30 mL), dried over Na_2SO_4 and concentrated under reduced pressure. The resulting residue was purified by flash column chromatography on silica gel (EtOAc /MeOH 4:1) to afford *rac*-**33** as colorless solid (20.0 mg, 43%).

R*f* = 0.27 (EtOAc /MeOH 4:1).

NMR Spectroscopy:

^{1}H NMR (500 MHz, $CDCl_3$, 23 °C, δ): 7.31 – 7.21 (m, 2H), 7.21 – 7.11 (m, 3H), 3.42 (dd, *J* = 8.3, 6.4 Hz, 1H), 2.61 (t, 2H), 1.98 – 1.75 (m, 2H), 1.71 – 1.59 (m, 2H), 1.55 – 1.50 (m, 1H), 1.48 – 1.32 (m, 3H), 1.29 (s, 12H).

^{13}C NMR (125 MHz, $CDCl_3$, 23 °C, δ): 142.7, 128.5, 128.3, 125.7, 84.5, 35.9, 34.0, 31.4, 28.8, 27.2, 24.7, 24.7.

HRMS-ESIpos(m/z) calc'd for $C_{16}H_{28}ClBO_2Na$ $[M+Na]^+$, 345.1763; found, 345.1765; deviation: +0.6 ppm.

rac-Dimethyl 2-chloro-3-(4-phenylbutyl)succinate 34

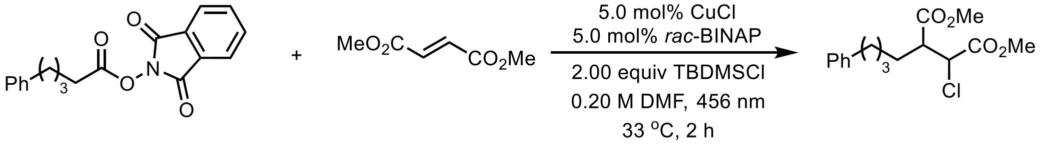

1 **S34** *rac*-**34**, 84%, 1:1 dr

Under an ambient atmosphere, to a 4-mL borosilicate vial equipped with a Teflon-coated magnetic stir bar were added 1,3-dioxoisoindolin-2-yl 5-phenylpentanoate **1** (129 mg, 0.400 mmol, 2.00 equiv), copper chloride (CuCl) (1.0 mg, 10 µmol, 5.0 mol%), *rac*-2,2'-bis-(diphenylphosphino)-1,1'-binaphthyl (*rac*-BINAP) (6.2 mg, 10 µmol, 5.0 mol%), and *tert*-butyldimethylchlorosilane (TBDMSCl) (60.3 mg, 0.400 mmol, 2.00 equiv) were dissolved in *N*,*N*-dimethylformamide (DMF) (1.0 mL, c = 0.20 M) and then dimethyl fumarate **S34** (28.8 mg, 0.200 mmol, 1.00 equiv) was added. The vial was evacuated and the solvent was bubbled with inert gas using a Schlenk line under 0.50 mbar pressure for 30 seconds. The reaction mixture was irradiated with blue LEDs (456 nm, 2 × 40 W) at 33 °C for 2 h. Following irradiation, ethyl acetate (30 mL) and brine (30 mL) were added. The mixture was extracted once with ethyl acetate (30 mL), and the organic layer was washed with brine (30 mL), dried over Na_2SO_4 and concentrated under reduced pressure. The diastereomeric ratio of the crude product was determined using ^{1}H NMR spectroscopy. The resulting residue was purified by flash

column chromatography on silica gel (Pentane/Et_2O 10:1) to afford *rac*-**34** as colorless oil (52.4 mg, 84%).

R*f* = 0.23 (Pentane/Et_2O 10:1).

NMR Spectroscopy:

^{1}H NMR (500 MHz, $CDCl_3$, 23 °C, δ): 7.28 (t, *J* = 7.5 Hz, 2H), 7.21 – 7.13 (m, 3H), 4.48 (dd, *J* = 30.0, 9.1 Hz, 1H), 3.79 (d, *J* = 8.9 Hz, 3H), 3.70 (d, *J* = 17.4 Hz, 3H), 3.19 – 2.97 (m, 1H), 2.61 (ddt, *J* = 14.8, 8.4, 5.4 Hz, 2H), 1.93 – 1.79 (m, 1H), 1.78 – 1.47 (m, 3H), 1.48 – 1.21 (m, 2H).

^{13}C NMR (125 MHz, $CDCl_3$, 23 °C, δ): 172.9, 172.1, 169.3, 168.7, 142.2, 142.1, 128.4, 128.3, 125.8, 57.0, 56.5, 53.2, 52.2, 50.1, 48.5, 35.5, 31.1, 30.9, 29.4, 28.3, 26.7, 25.7.

HRMS-ESIpos (m/z) calc'd for $C_{16}H_{21}ClO_4Na$ $[M+Na]^+$, 335.1020; found, 335.1019; deviation: –0.2 ppm.

rac-Dimethyl 2-chloro-2-(5-phenylpentyl)succinate 35

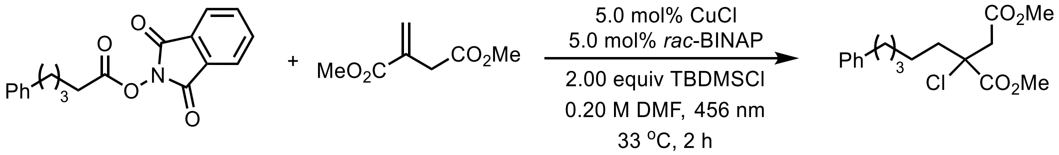

1 **S35** *rac*-**35**, 79%

Under an ambient atmosphere, to a 4-mL borosilicate vial equipped with a Teflon-coated magnetic stir bar were added 1,3-dioxoisoindolin-2-yl 5-phenylpentanoate **1** (129 mg, 0.400 mmol, 2.00 equiv), copper chloride (CuCl) (1.0 mg, 10 µmol, 5.0 mol%), *rac*-2,2'-bis-(diphenylphosphino)-1,1'-binaphthyl (*rac*-BINAP) (6.2 mg, 10 µmol, 5.0 mol%), and *tert*-butyldimethylchlorosilane (TBDMSCl) (60.3 mg, 0.400 mmol, 2.00 equiv) were dissolved in *N*,*N*-dimethylformamide (DMF) (1.0 mL, c = 0.20 M) and then dimethyl 2-methylenesuccinate **S35** (31.6 mg , 28.1 µL, 0.200 mmol, 1.00 equiv) was added. The vial was evacuated and the solvent was bubbled with inert gas using a Schlenk line under 0.50 mbar pressure for 30 seconds. The reaction mixture was irradiated with blue LEDs (456 nm, 2 × 40 W) at 33 °C for 2 h. Following irradiation, ethyl acetate (30 mL) and brine (30 mL) were added. The mixture was extracted once with ethyl acetate (30 mL), and the organic layer was washed with brine (30 mL), dried over Na_2SO_4 and concentrated under reduced pressure. The resulting residue was purified by flash column chromatography on silica gel (Pentane/Et_2O 10:1) to afford *rac*-**35** as colorless oil (51.5 mg, 79%).

R*f* = 0.17 (Pentane/Et_2O 10:1).

NMR Spectroscopy:

^{1}H NMR (500 MHz, $CDCl_3$, 23 °C, δ): 7.32 – 7.25 (m, 2H), 7.20 – 7.14 (m, 3H), 3.81 (s, 3H), 3.69 (s, 3H), 3.27 – 2.93 (m, 2H), 2.60 (t, *J* = 7.7 Hz, 2H), 2.05 (dddd, *J* = 46.7, 14.3, 11.5, 4.2 Hz, 2H), 1.64 (p, *J* = 7.6 Hz, 2H), 1.51 (ddt, *J* = 14.5, 7.4, 3.7 Hz, 1H), 1.43 – 1.31 (m, 3H).

^{13}C NMR (125 MHz, $CDCl_3$, 23 °C, δ): 170.7, 169.5, 142.4, 128.4, 128.3, 125.7, 69.1, 53.4, 52.1, 45.0, 40.3, 35.8, 31.1, 28.8, 24.1.

HRMS-ESIpos (m/z) calc'd for $C_{17}H_{23}ClO_4Na$ [M+Na]$^+$, 349.1177; found, 349.1178; deviation: +0.3 ppm.

rac-2-Chloro-2-methyl-7-phenylheptanal 36

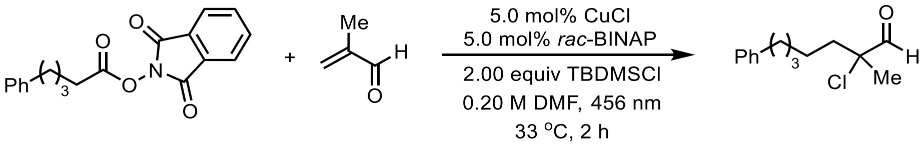

1 S36 *rac*-**36**, 58%

Under an ambient atmosphere, to a 4-mL borosilicate vial equipped with a Teflon-coated magnetic stir bar were added 1,3-dioxoisoindolin-2-yl 5-phenylpentanoate **1** (129 mg, 0.400 mmol, 2.00 equiv), copper chloride (CuCl) (1.0 mg, 10 µmol, 5.0 mol%), *rac*-2,2'-bis-(diphenylphosphino)-1,1'-binaphthyl (*rac*-BINAP) (6.2 mg, 10 µmol, 5.0 mol%), and *tert*-butyldimethylchlorosilane (TBDMSCl) (60.3 mg, 0.400 mmol, 2.00 equiv) were dissolved in *N*,*N*-dimethylformamide (DMF) (1.0 mL, c = 0.20 M) and then methacrylaldehyde **S36** (14.0 mg, 16.5 µL, 0.20 mmol, 1.00 equiv) was added. The vial was evacuated and the solvent was bubbled with inert gas using a Schlenk line under 0.50 mbar pressure for 30 seconds. The reaction mixture was irradiated with blue LEDs (456 nm, 2 × 40 W) at 33 °C for 2 h. Following irradiation, ethyl acetate (30 mL) and brine (30 mL) were added. The mixture was extracted once with ethyl acetate (30 mL), and the organic layer was washed with brine (30 mL), dried over Na_2SO_4 and concentrated under reduced pressure. The resulting residue was purified by flash column chromatography on silica gel (Pentane/EtOAc 10:1) to afford *rac*-**36** as colorless oil (27.7 mg, 58%).

R*f* = 0.66 (Pentane/EtOAc 10:1).

NMR Spectroscopy:

^{1}H NMR (500 MHz, $CDCl_3$, 23 °C, δ): 9.43 (s, 1H), 7.28 (q, *J* = 6.6 Hz, 2H), 7.22 – 7.13 (m, 3H), 2.62 (t, *J* = 7.7 Hz, 2H), 1.87 (dddd, *J* = 58.4, 14.2, 11.0, 5.2 Hz, 2H), 1.65 (p, *J* = 7.5 Hz, 2H), 1.58 (s, 3H), 1.52 – 1.28 (m, 4H).

^{13}C NMR (125 MHz, $CDCl_3$, 23 °C, δ): 195.9, 142.5, 128.5, 128.4, 125.8, 73.6, 38.7, 35.8, 31.2, 29.2, 24.1, 23.7.

HRMS-EI (m/z) calc'd for $C_{14}H_{19}ClO$ [M]$^+$, 238.1118; found, 238.1116; deviation: –0.8 ppm.

rac-Methyl 2-chloro-2-methyl-7-phenylheptanoate 37

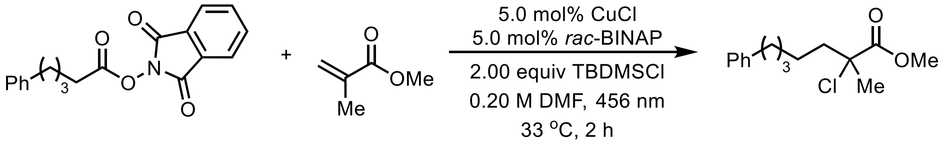

1 S37 *rac*-**37**, 66%

Under an ambient atmosphere, to a 4-mL borosilicate vial equipped with a Teflon-coated magnetic stir bar were added 1,3-dioxoisoindolin-2-yl 5-phenylpentanoate **1** (129 mg, 0.400 mmol, 2.00 equiv), copper chloride (CuCl) (1.0 mg, 10 µmol, 5.0 mol%), *rac*-2,2'-bis-(diphenylphosphino)-1,1'-binaphthyl (*rac*-BINAP) (6.2 mg, 10

µmol, 5.0 mol%), and *tert*-butyldimethylchlorosilane (TBDMSCl) (60.3 mg, 0.400 mmol, 2.00 equiv) were dissolved in *N*,*N*-dimethylformamide (DMF) (1.0 mL, c = 0.20 M) and then methyl methacrylate **S37** (20.0 mg, 21.4 µL, 0.200 mmol, 1.00 equiv) was added. The vial was evacuated and the solvent was bubbled with inert gas using a Schlenk line under 0.50 mbar pressure for 30 seconds. The reaction mixture was irradiated with blue LEDs (456 nm, 2 × 40 W) at 33 °C for 2 h. Following irradiation, ethyl acetate (30 mL) and brine (30 mL) were added. The mixture was extracted once with ethyl acetate (30 mL), and the organic layer was washed with brine (30 mL), dried over Na_2SO_4 and concentrated under reduced pressure. The resulting residue was purified by flash column chromatography on silica gel (hexanes/EtOAc 40:1) to afford *rac*-**37** as yellow oil (35.3 mg, 66%).

R*f* = 0.57 (hexanes/EtOAc 40:1).

NMR Spectroscopy:

^{1}H NMR (500 MHz, $CDCl_3$, 23 °C, δ): 7.29 (t, *J* = 7.6 Hz, 2H), 7.24 – 7.12 (m, 3H), 3.79 (s, 3H), 2.96 – 2.43 (m, 1H), 2.12 – 1.93 (m, 2H), 1.75 (s, 3H), 1.69 – 1.53 (m, 2H), 1.52 – 1.32 (m, 4H).

^{13}C NMR (125 MHz, $CDCl_3$, 23 °C, δ): 172.0, 142.5, 128.5, 128.3, 125.8, 69.1, 53.1, 42.2, 35.8, 31.2, 29.1, 27.8, 24.8.

HRMS-EI (m/z) calc'd for $C_{15}H_{21}ClO_2$ $[M]^+$, 268.1224; found, 268.1225; deviation: +0.2 ppm.

rac-2-(2-Chloro-7-phenylheptanamido)-2-methylpropane-1-sulfonic acid 38

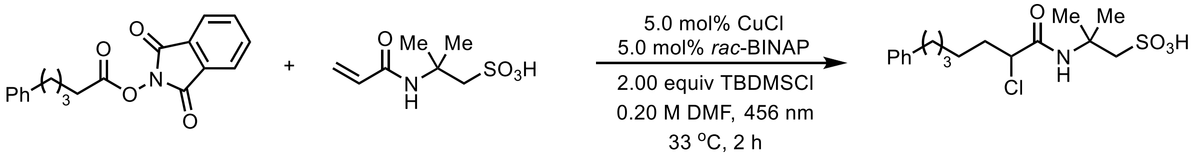

1 **S38** *rac*-**38**, 43%

Under an ambient atmosphere, to a 4-mL borosilicate vial equipped with a Teflon-coated magnetic stir bar were added 1,3-dioxoisoindolin-2-yl 5-phenylpentanoate **1** (129 mg, 0.400 mmol, 2.00 equiv), copper chloride (CuCl) (1.0 mg, 10 µmol, 5.0 mol%), *rac*-2,2'-bis-(diphenylphosphino)-1,1'-binaphthyl (*rac*-BINAP) (6.2 mg, 10 µmol, 5.0 mol%), and *tert*-butyldimethylchlorosilane (TBDMSCl) (60.3 mg, 0.400 mmol, 2.00 equiv) were dissolved in *N*,*N*-dimethylformamide (DMF) (1.0 mL, c = 0.20 M) and then 2-acrylamido-2-methylpropane-1-sulfonic acid **S38** (41.4 mg, 0.200 mmol, 1.00 equiv) was added. The vial was evacuated and the solvent was bubbled with inert gas using a Schlenk line under 0.50 mbar pressure for 30 seconds. The reaction mixture was irradiated with blue LEDs (456 nm, 2 × 40 W) at 33 °C for 2 h. Following irradiation, ethyl acetate (30 mL) and brine (30 mL) were added. The mixture was extracted once with ethyl acetate (30 mL), and the organic layer was washed with brine (30 mL), dried over Na_2SO_4 and concentrated under reduced pressure. The resulting residue was purified by flash column chromatography on silica gel (EtOAc/MeOH 4:1) to afford *rac*-**38** as colorless solid (32.2 mg, 43%).

R*f* = 0.27 (EtOAc/MeOH 4:1).

NMR Spectroscopy:

^{1}H NMR (500 MHz, DMSO, 23 °C, δ): 7.32 – 7.24 (m, 2H), 7.19 (td, *J* = 7.4, 1.5 Hz, 3H), 4.17 (dd, *J* = 8.5, 5.5 Hz, 1H), 2.62 (t, 2H), 2.31 (s, 3H), 2.00 – 1.77 (m, 2H), 1.65 (p, *J* = 6.5 Hz, 2H), 1.58 – 1.48 (m, 1H), 1.47 – 1.31 (m, 3H).

^{13}C NMR (125 MHz, DMSO, 23 °C, δ): 167.2, 142.2, 128.2, 128.2, 125.6, 60.3, 59.8, 52.0, 34.9, 34.7, 30.8, 27.9, 25.9, 25.7, 25.2.

HRMS-ESIneg (m/z) calc'd for $C_{17}H_{25}ClNSO_4$ [M–H]$^-$, 374.1198; found, 374.1201; deviation: +0.8 ppm.

Bicyclopentane-derived tripeptide α-chloronitrile 39

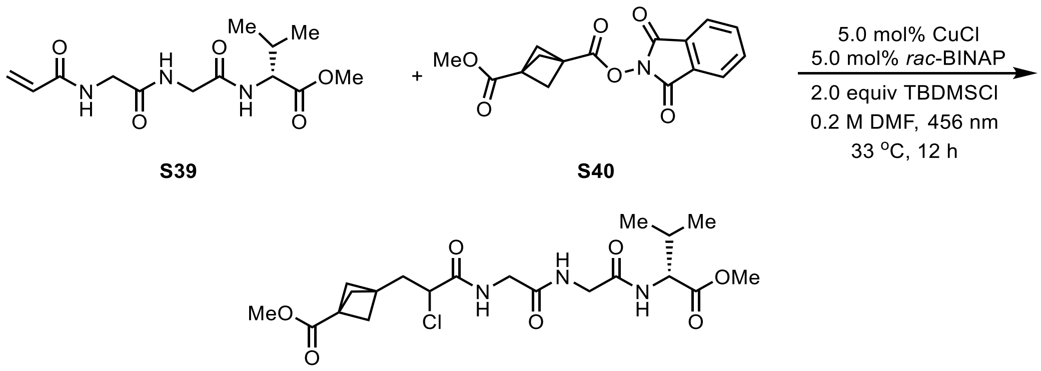

39, 33%

Under an inert atmosphere, to a 4-mL borosilicate vial equipped with a Teflon-coated magnetic stir bar were added methyl acryloylglycylglycylvalinate **S39** (30.0 mg, 0.100 mmol, 1.00 equiv), 1-(1,3-dioxoisoindolin-2-yl) 3-methyl bicyclo[1.1.1]pentane-1,3-dicarboxylate **S40** (126 mg, 0.400 mmol, 4.00 equiv), copper chloride (CuCl) (0.5 mg, 5 µmol, 5.0 mol%), (+-)-2,2'-Bis-(diphenylphosphino)-1,1'-binaphthyl (BINAP) (3.1 mg, 5 µmol, 5.0 mol%) and *tert*.-Butyldimethylchlorsilan (TBDMSCl) (45.2 mg, 0.300 mmol, 3.00 equiv) and then dissolved in *N*,*N*-Dimethylformamid DMF (0.5 mL, c = 0.10 M). The vial was evacuated and the solvent was bubbled with inert gas using a Schlenk line under 0.50 mbar pressure. The reaction mixture was irradiated with blue LEDs (456 nm, 2 × 40 W) for 12 h at 33 °C. Following irradiation, ethyl acetate (30 mL) and brine (30 mL) were added. The mixture was extracted once with ethyl acetate (30 mL), and the organic layer was washed with brine (30 mL), dried over Na_2SO_4 and concentrated under reduced pressure. The resulting residue was purified by flash column chromatography on silica gel (DCM/MeOH 6:1) to afford **39** as colorless solid (15.0 mg, 33%).

R_f = 0.62 (DCM/MeOH 6:1).

NMR Spectroscopy:

^{1}H NMR (500 MHz, $CDCl_3$, 23 °C, δ): 7.48 (q, *J* = 4.6 Hz, 1H), 7.12 – 6.99 (m, 1H), 6.82 (d, *J* = 8.8 Hz, 1H), 4.53 (dd, *J* = 8.7, 5.1 Hz, 1H), 4.34 (dt, *J* = 8.9, 4.5 Hz, 1H), 4.12 – 3.99 (m, 4H), 3.73 (s, 3H), 3.65 (s, 3H), 2.38 (dt, *J* = 15.1, 4.6 Hz, 1H), 2.23 – 2.08 (m, 2H), 2.02 (s, 6H), 0.92 (dd, *J* = 13.6, 6.8 Hz, 6H).

^{13}C NMR (125 MHz, $CDCl_3$, 23 °C, δ): 172.39, 170.2, 169.4, 169.3, 168.7, 168.7, 168.5, 58.0, 57.5, 52.5, 52.43, 51.7, 43.4, 43.1, 38.3, 37.9, 36.6, 31.2, 19.0, 17.9.

HRMS-ESIpos (m/z) calc'd for $C_{20}H_{30}ClN_3O_7Na$ $[M+Na]^+$, 482.1664; found, 482.1663; deviation: –0.3 ppm.

Reaction condition optimization

General procedure for condition optimization

To a 4-mL borosilicate vial containing a Teflon-coated magnetic stirring bar were added redox-active ester **1** (32.3 mg, 0.100 mmol, 1.00 equiv), photocatalyst (5 µmol, 5 mol%), copper chloride (CuCl) (0.5 mg, 5 µmol, 5 mol%), ligand (5 µmol, 5 mol%), and (pseudo)halide source (0.200 mmol, 2.00 equiv) in a solvent (0.5 mL, c = 0.20 M) and then alkene (0.300 mmol, 3.00 equiv) was added. Roughly 100 mL of argon was bubbled through the reaction mixture using a Schlenk line for 30 seconds. The reaction mixture was irradiated with blue LEDs (456 nm, 2 × 40 W) for 12 h at 33 °C. Following irradiation, internal standard CH_2Br_2 (50 µL, c = 0.20 M) was added, and from the mixture 20 µL was taken and dissolved with $CDCl_3$ then analyzed by ^{1}H NMR spectroscopy.

Table S1. Condition optimization

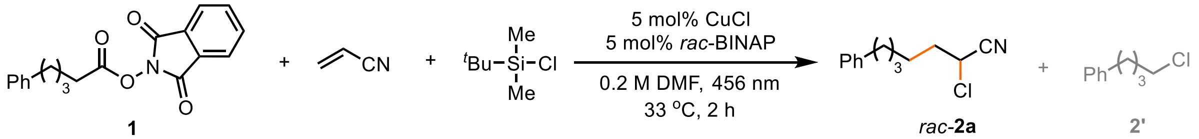

1 *rac*-**2a** **2'**

entry	variations	yield of *rac*-2a, 2' (%)	entry	variations	yield of *rac*-2a, 2' (%)
**1**	none	84, 10	**12**	*N*-isopropylacrylamide	30, 27
**2**	**1** was generated in one pot	71, 18		**solvents**	
**3**	in air	78, 14	**13**	MeCN	10, 6
**4**	2.0 equiv 1 and 1.0 equiv acrylonitrile	93, 72	**14**	THF	18, 10
	**(psuedo)halide sources**		**15**	DCM	5, 2
**5**	Tetrabutylammonium chloride	3, 5	**16**	DMSO	<2, <2
**6**	Pyridine hydrochloride	61, 22	**17**	1,4-Dioxane	26, 20
**7**	TMSCl	69, 15		**ligands**	
**8**	TMSBr	41, 54	**18**[b]	$Cu(dpp)_2PF_6$	<2, <2
**9**[a]	TMSBr	85, 97	**19**	Xantphos	5, 3
**10**[a]	$TMSN_3$	6, <2	**20**	(*R*)-Segphos	50, 4
	**Michael acceptors**		**21**	(*R*)-DTBM-segphos	48, <2
**11**	Methyl acrylate	44, 22	**22**	DPPF	<2, <2

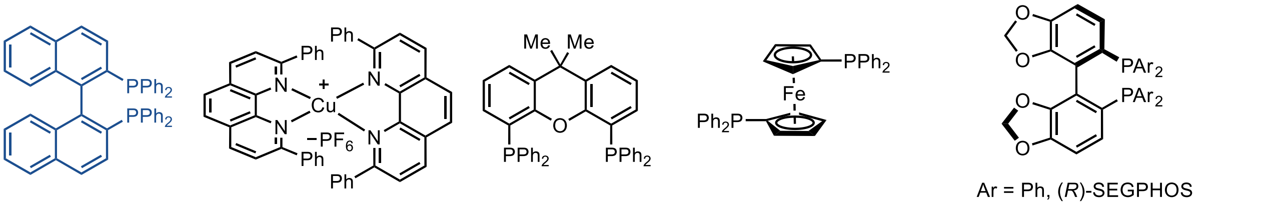

rac-BINAP $Cu(dpp)_2PF_6$ Xantphos DPPF Ar = Ph, (*R*)-SEGPHOS
Ar = 3,5-di-tert-butyl-4-methoxyphenyl, (*R*)-DTBM-segphos

[a]2.0 equiv **1** and 1.0 equiv acrylonitrile were used; [b]without CuCl.

Gram-scale synthesis

Evans auxiliary-derived α-chloro amide 41

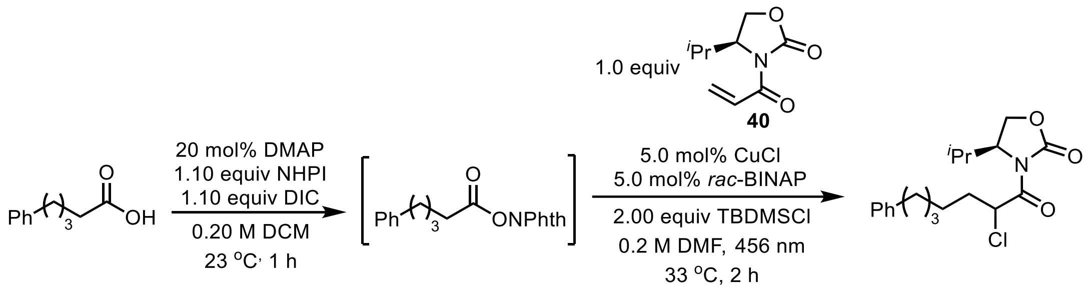

S1 **1** **41**, 61% 1.2:1 dr

Under an ambient atmosphere, to a 50-mL borosilicate flask equipped with a Teflon-coated magnetic stir bar were added 5-phenylpentanoic acid **S1** (1.42 g, 8.00 mmol, 2.00 equiv), *N*-Hydroxyphthalimid (NHPI) (1.43 g, 8.80 mmol, 2.20 equiv), 4-(Dimethylamino)-pyridin (DMAP) (0.19 g, 1.60 mmol, 0.4 equiv), *N*,*N*'-Diisopropylcarbodiimid (DIC) (1.36 mL, 8.80 mmol, 2.20 equiv) in Dichloromethane (DCM) (40 mL, c = 0.20 M). After stirring the mixture at 23 °C for 1 h the solvent was removed under reduced pressure. Without purification of the crude redox-active ester **1**, copper chloride (CuCl) (20.0 mg, 0.2 mmol, 0.05 equiv), (+-)-2,2'-Bis-(diphenylphosphino)-1,1'-binaphthyl (BINAP) (0.124 g, 0.2 mmol, 0.05 equiv) and *tert*.-Butyldimethylchlorsilan (TBDMSCl) (1.20 g, 8.0 mmol, 2.00 equiv) were dissolved in *N*,*N*-Dimethylformamid DMF (20 mL, c = 0.20 M) and then (S)-3-acryloyl-4-isopropyloxazolidin-2-one **40** (0.73 g, 4.0 mmol, 1.00 equiv) was added. The vial was evacuated and the solvent was bubbled with inert gas using a Schlenk line under 0.50 mbar pressure. The reaction mixture was irradiated with blue LEDs (456 nm, 2 × 40 W) at 33 °C for 2 h. After reaction completion brine (2 × 30 mL) was added and extracted by ethyl acetate (30 mL). The organic layer was washed by Na_2CO_3 (5 × 20 mL), dried over Na_2SO_4, and concentrated under reduced pressure. The diastereomeric ratio of the crude product was determined using ^{1}H NMR spectroscopy. The resulting residue was purified by flash column chromatography on silica gel (Pentane/Et_2O 5:1) to afford **41** as colorless oil (0.856 g, 61%).

R_f = 0.23 (Pentane/Et_2O 5:1).

NMR Spectroscopy:

^{1}H NMR (500 MHz, $CDCl_3$, 23 °C, δ): 7.32 – 7.25 (m, 2H), 7.18 (dd, *J* = 5.5, 3.5 Hz, 3H), 5.63 (ddd, *J* = 21.1, 8.1, 5.6 Hz, 1H), 4.47 (dt, *J* = 7.6, 3.6 Hz, 1H), 4.32 (dt, *J* = 13.5, 8.8 Hz, 1H), 4.25 (dt, *J* = 9.1, 3.1 Hz, 1H), 2.62 (t, *J* = 7.7 Hz, 2H), 2.38 (ddqd, *J* = 27.9, 13.9, 7.0, 3.9 Hz, 1H), 2.14 – 2.00 (m, 1H), 1.98 – 1.85 (m, 1H), 1.71 – 1.61 (m, 2H), 1.58 – 1.27 (m, 4H), 0.99 – 0.86 (m, 6H).

^{13}C NMR (125 MHz, $CDCl_3$, 23 °C, δ): 169.2, 169.1, 153.4, 153.1, 142.5, 142.5, 128.4, 128.3, 125.7, 63.9, 63.5, 59.0, 58.5, 55.7, 54.9, 35.8, 34.6, 33.6, 31.2, 31.2, 28.6, 28.6, 28.5, 28.0, 26.0, 26.0, 17.9, 17.8, 14.8, 14.6.

HRMS-ESIpos (m/z) calc'd for $C_{19}H_{26}ClNO_3Na$ $[M+Na]^+$, 374.1493; found, 374.1492; deviation: –0.3 ppm.

Transformations of the alkyl halide product

5-Phenylpentanoyl-derived α- amide 42

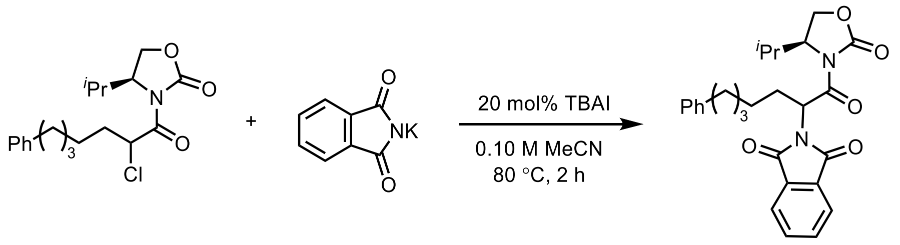

41 1.5 equiv **42**, 60% 2.3:1 dr

Under an ambient atmosphere, to a 4-mL borosilicate flask equipped with a Teflon-coated magnetic stir bar were added 3-(2-chloro-5-phenylpentanoyl)-4-isopropyloxazolidin-2-one **41** (35.2 mg, 0.100 mmol, 1.00 equiv), tetrabutylammonium iodide (TBAI) (7.40 mg, 20 µmol, 20 mol%), potassium phthalimide (27.8 mg, 0.150 mmol, 1.50 equiv) in anhydrous acetonitrile (MeCN) (1 mL, c = 0.10 M). After stirring the mixture at 80 °C for 2 h. After reaction completion, ethyl acetate (30 mL) and brine (30 mL) were added. The organic layer was washed by brine (30 mL), dried over Na_2SO_4 and concentrated under reduced pressure. The diastereomeric ratio of the crude product was determined using ^1H NMR spectroscopy. The resulting residue was purified by flash column chromatography on silica gel (Pentane/Et_2O 5:1) to afford **42** as colorless oil (43.0 mg, 93%). The sample contains a mixture of two diastereomers. The ratio of major to minor component is 2.3:1. Unfortunately, due to missing NOEs between the benzo-dioxazole and 4-isopropyloxazolidin-2-one we were not able to establish a relative stereochemistry of the individual diastereomers.

R_f = 0.37 (Pentane/Et_2O 5:1).

NMR Spectroscopy:

^{1}H NMR (500 MHz, $CDCl_3$, 23 °C, δ): 7.86 (ddd, *J* = 4.5, 3.2, 1.4 Hz, 2H), 7.73 (dd, *J* = 5.6, 2.9 Hz, 2H), 7.26 – 7.20 (m, 2H), 7.19 – 7.09 (m, 3H), 5.85 (dd, *J* = 11.2, 3.9 Hz, 1H), 4.46 (dq, *J* = 8.2, 3.7 Hz, 1H), 4.32 (q, *J* = 8.9 Hz, 1H), 4.23 (dd, *J* = 9.1, 3.5 Hz, 1H), 2.71 – 2.59 (m, 1H), 2.56 (t, *J* = 7.7 Hz, 2H), 2.39 (ddtd, *J* = 18.0, 14.1, 6.8, 3.7 Hz, 1H), 2.19 – 1.98 (m, 1H), 1.66 – 1.53 (m, 2H), 1.51 – 1.32 (m, 4H), 0.96 – 0.79 (m, 6H).

^{13}C NMR (125 MHz, $CDCl_3$, 23 °C, δ): 169.8, 169.5, 168.3, 168.0, 153.4, 153.3, 142.7, 134.3, 134.2, 132.7, 131.8, 128.4, 128.2, 125.6, 123.6, 123.5, 64.1, 63.7, 59.2, 58.5, 54.8, 54.2, 35.8 (d, *J* = 3.4 Hz), 31.2, 28.8, 28.7, 28.5, 28.4, 28.4, 27.9, 26.7, 26.4, 18.1, 17.8, 14.9, 14.4.

HRMS-ESIpos (m/z) calc'd for $C_{27}H_{30}N_2O_5Na$ $[M+Na]^+$, 485.2046; found, 485.2047; deviation: +0.1 ppm.

5-Phenylpentanoyl-derived α-morpholine amide 43

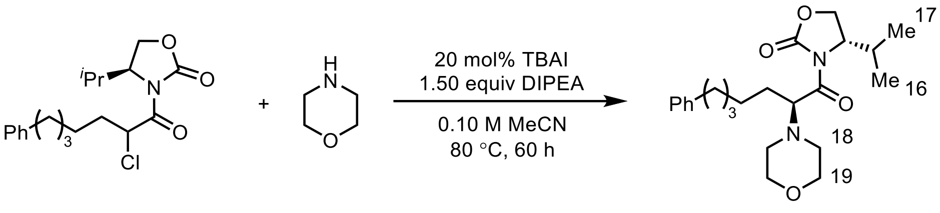

41 1.5 equiv **43**, 60% >20:1 dr

Under an ambient atmosphere, to a 4-mL borosilicate flask equipped with a Teflon-coated magnetic stir bar were added 3-(2-chloro-5-phenylpentanoyl)-4-isopropyloxazolidin-2-one **41** (35.2 mg, 0.100 mmol, 1.00 equiv), tetrabutylammonium iodide (TBAI) (7.40 mg, 20 μmol, 20 mol%), *N*-ethyldiisopropylamine (DIPEA) (26.1 μL, 0.150 mmol, 1.50 equiv), morpholine (13 μL, 0.150 mmol, 1.50 equiv) in anhydrous acetonitrile (MeCN) (1 mL, c = 0.10 M). After stirring the mixture at 80 °C for 60 h. After reaction completion, ethyl acetate (30 mL) and brine (30 mL) were added. The organic layer was washed by brine (30 mL), dried over Na_2SO_4 and concentrated under reduced pressure. The diastereomeric ratio of the crude product was determined using HPLC spectroscopy. The resulting residue was purified by flash column chromatography on silica gel (Pentane/Et_2O 5:1) to afford **43** as colorless oil (24.2 mg, 60%). Only one diastereomer is present in the sample. The relative stereochemistry was proposed based on an observed long range NOE from H16/17 to H18/H19.

R_f = 0.25 (Pentane/Et_2O 5:1).

NMR Spectroscopy:

^{1}H NMR (500 MHz, $CDCl_3$, 23 °C, δ): 7.30 – 7.23 (m, 2H), 7.20 – 7.13 (m, 3H), 4.71 (dd, *J* = 8.8, 5.8 Hz, 1H), 4.48 (dt, *J* = 8.3, 3.4 Hz, 1H), 4.26 (t, *J* = 8.7 Hz, 1H), 4.21 (dd, *J* = 9.1, 3.2 Hz, 1H), 3.65 (t, *J* = 4.6 Hz, 4H), 2.69 (dd, *J* = 5.8, 3.5 Hz, 4H), 2.60 (t, *J* = 7.7 Hz, 2H), 2.35 (heptd, *J* = 7.0, 3.7 Hz, 1H), 1.81 – 1.70 (m, 1H), 1.67 – 1.56 (m, 3H), 1.43 – 1.20 (m, 4H), 0.93 (dd, *J* = 6.9, 1.6 Hz, 6H).

^{13}C NMR (125 MHz, $CDCl_3$, 23 °C, δ): 172.3, 153.8, 142.7, 128.5, 128.3, 125.7, 67.7, 63.2, 63.1, 58.5, 49.6, 35.9, 31.4, 29.3, 28.5, 26.3, 26.1, 18.1, 14.7.

HRMS-ESIpos (m/z) calc'd for $C_{23}H_{35}N_2O_4$ $[M+H]^+$, 403.2591; found, 403.2591; deviation: +0.1 ppm.

5-Phenylpentanoyl-derived α-phenylester amide 44

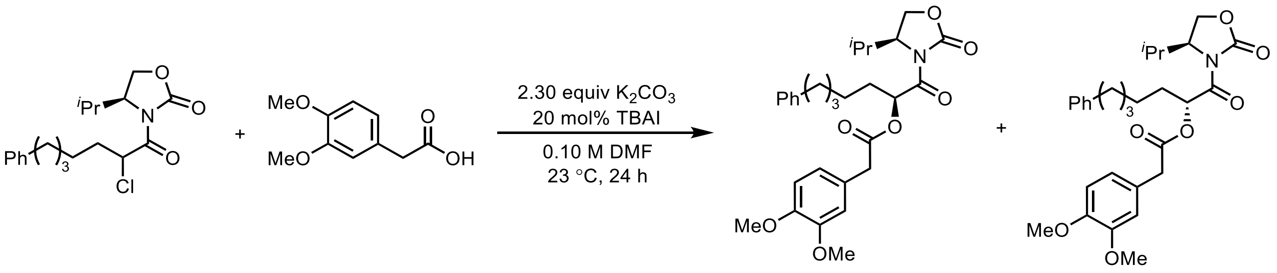

41 2.2 equiv **44+44'**, 82%, 1.2:1 dr

Under an ambient atmosphere, to a 4-mL borosilicate flask equipped with a Teflon-coated magnetic stir bar were added 3,4-Dimethoxyphenylacetic acid (86.3 mg, 0.440 mmol, 2.20 equiv) and potassium carbonate (K_2CO_3) (63.6 mg, 0.460 mmol, 2.30 equiv) in *N*,*N*-Dimethylformamid DMF (2 mL, c = 0.10 M) and stirred at 23 °C for 30 min. To the reaction mixture 3-(2-chloro-5-phenylpentanoyl)-4-isopropyloxazolidin-2-one **41** (70.3 mg, 0.200 mmol, 1.00 equiv) and tetrabutylammonium iodide (TBAI) (15 mg, 40 µmol, 20 mol%) were added and the mixture was stirred at 23 °C for 24 h. After reaction completion, ethyl acetate (30 mL) and brine (30 mL) were added. The organic layer was washed by brine (30 mL), dried over Na_2SO_4 and concentrated under reduced pressure. The diastereomeric ratio of the crude product was determined using ^{1}H NMR spectroscopy. The resulting residue was purified by flash column chromatography on silica gel (Pentane/Et_2O 10:1) to afford **44** (46.0 mg, 45%) and **44'** (38.0 mg, 37%) as colorless oil. Unfortunately, due to missing NOEs between the dimethoxyphenyl and 4-isopropyloxazolidin-2-one we were not able to establish a relative stereochemistry of the individual diastereomers.

Isomer 1: R*f* = 0.25 (Pentane/Et_2O 5:1). **Isomer 2: R*f*** = 0.22 (Pentane/Et_2O 5:1).

NMR Spectroscopy:

^{1}H NMR (500 MHz, $CDCl_3$, 23 °C, δ): **Isomer 1:** 7.27 (t, *J* = 7.6 Hz, 2H), 7.16 (d, *J* = 8.2 Hz, 3H), 6.83 (dd, *J* = 16.3, 2.7 Hz, 3H), 5.88 (dd, *J* = 9.0, 3.2 Hz, 1H), 4.39 (dd, *J* = 8.2, 3.4 Hz, 1H), 4.33 – 4.20 (m, 2H), 3.87 (s, 3H), 3.84 (s, 3H), 3.65 (s, 2H), 2.57 (t, *J* = 7.8 Hz, 2H), 2.44 – 2.34 (m, 1H), 1.87 – 1.69 (m, 2H), 1.66 – 1.53 (m, 2H), 1.47 (p, *J* = 7.6 Hz, 2H), 1.40 – 1.29 (m, 2H), 0.93 – 0.87 (m, 6H). **Isomer 2:** 7.31 – 7.24 (m, 2H), 7.21 – 7.14 (m, 3H), 6.83 (dd, *J* = 12.1, 2.0 Hz, 3H), 5.89 (dd, *J* = 8.9, 3.2 Hz, 1H), 4.44 (ddd, *J* = 8.4, 4.1, 2.8 Hz, 1H), 4.34 (t, *J* = 8.7 Hz, 1H), 4.22 (dd, *J* = 9.1, 2.9 Hz, 1H), 3.88 (s, 3H), 3.84 (s, 3H), 3.66 (d, *J* = 1.8 Hz, 2H), 2.59 (t, *J* = 7.7 Hz, 2H), 2.29 (pd, *J* = 7.0, 4.0 Hz, 1H), 1.95 – 1.77 (m, 2H), 1.68 – 1.55 (m, 2H), 1.50 (qt, *J* = 7.6, 4.6 Hz, 2H), 1.41 – 1.28 (m, 2H), 0.89 (dd, *J* = 18.1, 6.9 Hz, 6H).

^{13}C NMR (125 MHz, $CDCl_3$, 23 °C, δ): **Isomer 1:** 171.5, 170.6, 153.6, 149.0, 148.2, 142.7, 128.5, 128.4, 126.2, 125.8, 121.5, 112.5, 111.2, 73.3, 63.9, 58.7, 55.9, 55.9, 40.5, 35.9, 31.3, 30.7, 28.8, 28.0, 25.4, 17.9, 14.4. **Isomer 2:** 171.8, 170.4, 153.6, 149.0, 148.3, 142.7, 128.5, 128.4, 126.2, 125.8, 121.6, 112.6, 111.3, 73.0, 64.4, 58.4, 56.0, 56.0, 40.4, 35.9, 31.4, 31.1, 28.8, 28.8, 25.4, 18.0, 15.1.

HRMS-ESIpos (m/z) Isomer 1: calc'd for $C_{29}H_{37}NO_7Na$ $[M+Na]^+$, 534.2462; found, 534.2467; deviation: +0.9 ppm. **Isomer 2:** calc'd for $C_{29}H_{37}NO_7Na$ $[M+Na]^+$, 534.2462; found, 534.2464; deviation: +0.5 ppm.

5-Phenylpentanoyl-derived α-oxophthalimid 45

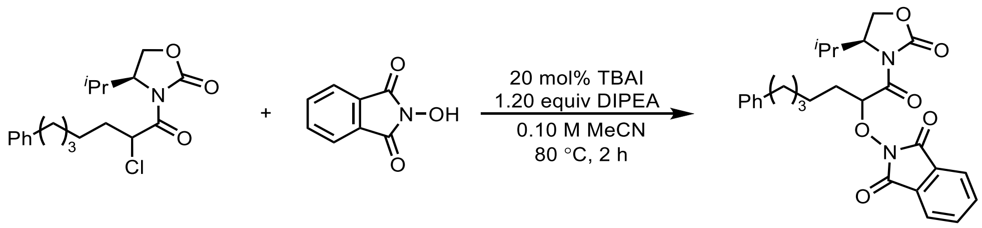

41 1.1 equiv **45**, 63% 7:1 dr

Under an ambient atmosphere, to a 4-mL borosilicate flask equipped with a Teflon-coated magnetic stir bar were added 3-(2-chloro-5-phenylpentanoyl)-4-isopropyloxazolidin-2-one **41** (35.2 mg, 0.100 mmol, 1.00 equiv), tetrabutylammonium iodide (TBAI) (7.40 mg, 20 µmol, 20 mol%), *N*-ethyldiisopropylamine (DIPEA) (21.0 µL, 0.120 mmol, 1.20 equiv) and *N*-Hydroxyphthalimid (NHPI) (18.0 g, 11.0 mmol, 1.10 equiv) in anhydrous acetonitrile (MeCN) (1 mL, c = 0.10 M). After stirring the mixture at 80 °C for 2 h. After reaction completion, ethyl acetate (30 mL) and brine (30 mL) were added. The organic layer was washed by brine (30 mL), dried over Na_2SO_4 and concentrated under reduced pressure. The diastereomeric ratio of the crude product was determined using ^{1}H NMR spectroscopy. The resulting residue was purified by flash column chromatography on silica gel (Pentane/Et_2O 5:1) to afford **45** as colorless oil (43.0 mg, 93%). The sample contains a mixture of two diastereomers. The ratio of major to minor component is 7:1. Unfortunately, due to missing NOEs between the benzo-dioxazole and 4-isopropyloxazolidin-2-one we were not able to establish a relative stereochemistry of the individual diastereomers.

R_f = 0.35 (Pentane/Et_2O 5:1).

NMR Spectroscopy:

^{1}H NMR (500 MHz, $CDCl_3$, 23 °C, δ): 7.82 (tt, *J* = 5.0, 2.3 Hz, 2H), 7.75 (dd, *J* = 5.5, 3.1 Hz, 2H), 7.31 – 7.24 (m, 2H), 7.22 – 7.13 (m, 3H), 5.95 (dd, *J* = 7.7, 4.8 Hz, 1H), 4.56 (ddd, *J* = 8.5, 4.1, 3.0 Hz, 1H), 4.39 (t, *J* = 8.7 Hz, 1H), 4.24 (dd, *J* = 9.1, 3.0 Hz, 1H), 2.64 (dd, *J* = 8.7, 6.7 Hz, 2H), 2.39 (heptd, *J* = 7.0, 4.2 Hz, 1H), 2.12 – 1.93 (m, 2H), 1.90 – 1.61 (m, 4H), 1.48 (qdt, *J* = 13.7, 9.0, 6.4 Hz, 2H), 0.91 (dd, *J* = 23.9, 6.9 Hz, 6H).

^{13}C NMR (125 MHz, $CDCl_3$, 23 °C, δ): 169.7, 163.6, 154.0, 142.8, 134.7, 134.7, 128.9, 128.5, 128.3, 125.7, 123.8, 84.0, 64.4, 58.7, 35.9, 31.5, 31.3, 29.0, 28.6, 25.0, 18.0, 15.0.

HRMS-ESIpos (m/z) calc'd for $C_{27}H_{30}N_2O_6Na$ $[M+Na]^+$, 501.1996; found, 501.1998; deviation: +0.5.

5-Phenylpentanoyl-derived α-phenylthio amide 46

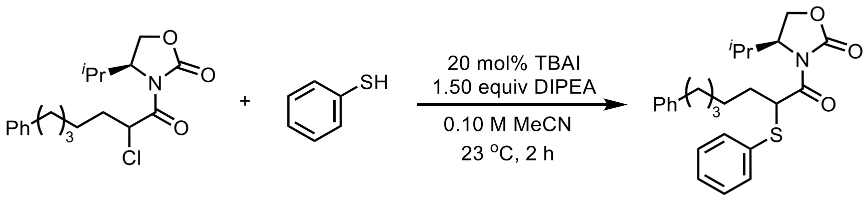

41 1.5 equiv **46**, 60% 1.3:1 dr

Under an ambient atmosphere, to a 4-mL borosilicate flask equipped with a Teflon-coated magnetic stir bar were added 3-(2-chloro-5-phenylpentanoyl)-4-isopropyloxazolidin-2-one 4**1** (35.2 mg, 0.100 mmol, 1.00 equiv), tetrabutylammonium iodide (TBAI) (7.40 mg, 20 µmol, 20 mol%), *N*-ethyldiisopropylamine (DIPEA) (26.1 µL, 0.150 mmol, 1.50 equiv), thiophenole (15.3 µL, 0.150 mmol, 1.50 equiv) in anhydrous acetonitrile (MeCN) (1 mL, c = 0.10 M). After stirring the mixture at room temperature for 2 h. After reaction completion, ethyl acetate (30 mL) and brine (30 mL) were added. The organic layer was washed by brine (30 mL), dried over Na_2SO_4 and concentrated under reduced pressure. The diastereomeric ratio of the crude product was

determined using ^{1}H NMR spectroscopy. The resulting residue was purified by flash column chromatography on silica gel (Pentane/ Et_2O 5:1) to afford **46** as colorless oil (37.7 mg, 89%). The sample contains a mixture of two diastereomers. The ratio of major to minor component is 1.3:1. Unfortunately, due to missing NOEs between the phenylthio and 4-isopropyloxazolidin-2-one we were not able to establish a relative stereochemistry of the individual diastereomers.

R_f = 0.25 (Pentane/Et_2O 5:1).

NMR Spectroscopy:

^{1}H NMR (500 MHz, $CDCl_3$, 23 °C, δ): 7.58 – 7.42 (m, 4H), 7.35 – 7.26 (m, 10H), 7.22 – 7.14 (m, 6H), 5.24 (dd, *J* = 8.3, 6.2 Hz, 1H), 5.17 (dd, *J* = 7.8, 6.7 Hz, 1H), 4.49 (dt, *J* = 8.3, 3.5 Hz, 1H), 4.32 (ddd, *J* = 8.2, 4.0, 2.5 Hz, 1H), 4.27 (t, *J* = 8.8 Hz, 1H), 4.23 (dd, *J* = 9.1, 3.3 Hz, 1H), 4.15 (dd, *J* = 9.0, 2.6 Hz, 1H), 4.12 – 4.03 (m, 1H), 2.59 (td, *J* = 7.8, 3.0 Hz, 4H), 2.37 (ttq, *J* = 10.2, 7.0, 3.3 Hz, 2H), 1.96 (dddd, *J* = 13.7, 10.2, 8.2, 5.0 Hz, 1H), 1.92 – 1.83 (m, 1H), 1.82 – 1.67 (m, 2H), 1.66 – 1.56 (m, 4H), 1.55 – 1.47 (m, 2H), 1.44 – 1.30 (m, 6H), 0.96 – 0.83 (m, 12H).

^{13}C NMR (125 MHz, $CDCl_3$, 23 °C, δ): 171.8, 171.7, 153.7, 142.6, 134.6, 134.1, 131.9, 128.9, 128.9, 128.6, 128.4, 128.4, 128.3, 128.3, 125.7, 125.7, 63.4, 63.0, 59.1, 58.5, 47.3, 47.0, 35.8, 35.8, 31.4, 31.3, 31.2, 30.9, 30.4, 28.9, 28.9, 28.7, 28.2, 27.0, 27.0, 18.0, 18.0, 14.8, 14.5.

HRMS- ESIpos (m/z) calc'd for $C_{25}H_{31}N_1O_3S$ $[M+Na]^+$, 448.1916; found, 448.1918; deviation: +0.3 ppm.

Mechanistic study

UV-vis absorption spectroscopy studies

All UV-Vis spectrum measurements were recorded on a Shimadzu UV-Vis spectrophotometer UV-2600 with temperature controller using a quartz cuvette (10 × 10 mm, 3.5 mL) in *N*,*N*-dimethylformamide (DMF).

UV-Vis spectra of the reaction mixture were measured with the following concentrations:

rac-BINAP (2.5 mg, 4 µmol, c = 0.2 mM) in DMF (20 mL);

rac-BINAPCuCl (2.9 mg, 4 µmol, c = 0.2 mM) in DMF (20 mL);

rac-BINAP (2.5 mg, 4 µmol, c = 0.2 mM) in DMF (20 mL) and CuCl (4.0 mg, 4 µmol, c = 0.04 mM) in DMF (100 mL);

rac-BINAP (3.1 mg, 5 µmol, c = 10 mM), CuCl (0.5 mg, 5 µmol, c = 10 mM), TBDMSCl (30.1 mg, 0.200 mmol, c = 0.4 M) and **1** (32.3 mg, 0.100 mmol, c = 0.2 M) in DMF (0.50 mL). From the mixture, 30 µL was taken and diluted with 3 mL of DMF;

rac-BINAP (3.1 mg, 5 µmol, c = 10 mM), CuCl (0.5 mg, 5 µmol, c = 10 mM) and TBDMSCl (30.1 mg, 0.200 mmol, c = 0.4 M) in DMF (0.50 mL). From the mixture, 30 µL was taken and diluted with 3 mL of DMF;

rac-BINAP (3.1 mg, 5 µmol, c = 10 mM), CuCl (0.5 mg, 5 µmol, c = 10 mM), TBDMSCl (30.1 mg, 0.200 mmol, c = 0.4 M), acrylonitrile (20.1 µL, 16.0 mg, 0.300 mmol, c = 0.6 M) and **1** (32.3 mg, 0.100 mmol, c = 0.2

M) in DMF (0.50 mL). From the mixture, 30 µL was taken and diluted with 3 mL of DMF;

rac-BINAP (3.1 mg, 5 µmol, c = 10 mM), CuCl (0.5 mg, 5 µmol, c = 10 mM), acrylonitrile (20.1 µL, 16.0 mg, 0.300 mmol, c = 0.6 M) and **1** (32.3 mg, 0.100 mmol, c = 0.2 M) in DMF (0.50 mL). From the mixture, 30 µL was taken and diluted with 3 mL of DMF.

1 (32.3 mg, 0.100 mmol, c = 0.2 M) in DMF (0.50 mL). From the mixture, 30 µL was taken and diluted with 3 mL of DMF.

TBDMSCl (30.1 mg, 0.200 mmol, c = 0.4 M) in DMF (0.50 mL). From the mixture, 30 µL was taken and diluted with 3 mL of DMF.

1 (32.3 mg, 0.100 mmol, c = 0.2 M) and TBDMSCl (30.1 mg, 0.200 mmol, c = 0.4 M) in DMF (0.50 mL). From the mixture, 30 µL was taken and diluted with 3 mL of DMF.

Segphos (2.4 mg, 4 µmol, c = 10 mM) in DMF (0.50 mL). From the mixture, 30 µL was taken and diluted with 3 mL of DMF.

Segphos (2.4 mg, 4 µmol, c = 10 mM) and CuCl (0.5 mg, 5 µmol, c = 10 mM) in DMF (0.50 mL). From the mixture, 30 µL was taken and diluted with 3 mL of DMF.

Xantphos (2.3 mg, 4 µmol, c = 10 mM) in DMF (0.50 mL). From the mixture, 30 µL was taken and diluted with 3 mL of DMF.

Xantphos (2.3 mg, 4 µmol, c = 10 mM) and CuCl (0.5 mg, 5 µmol, c = 10 mM) in DMF (0.50 mL). From the mixture, 30 µL was taken and diluted with 3 mL of DMF.

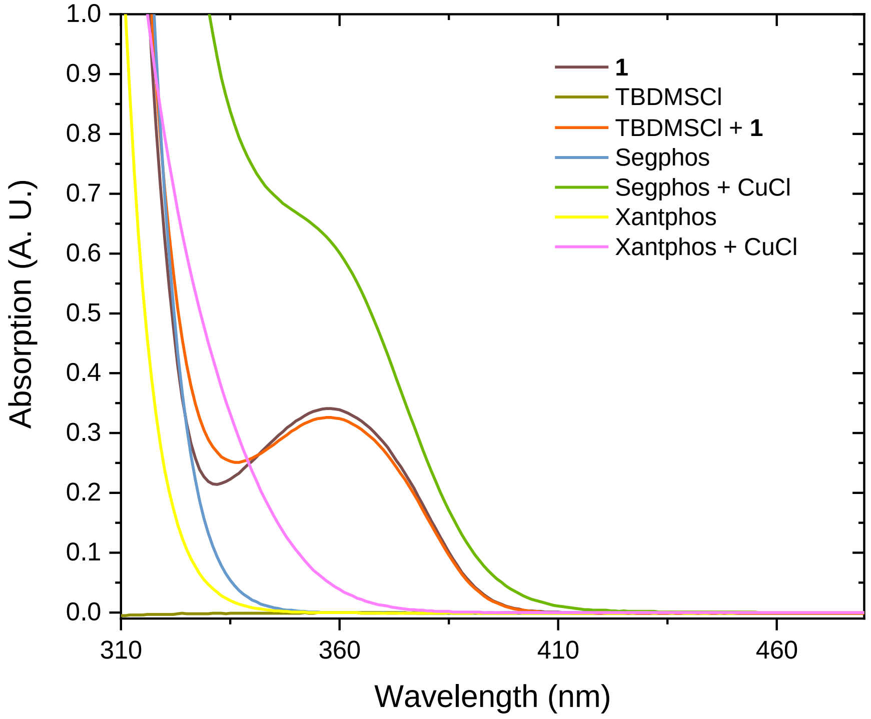

Figure S1. UV-Vis absorption spectra of **1** (brown), TBDMSCl (olive), TBDMSCl + **1** (orange), Segphos (dark blue), Segphos + CuCl (light green), ×antphos (yellow) and ×antphos + CuCl (pink) in DMF.

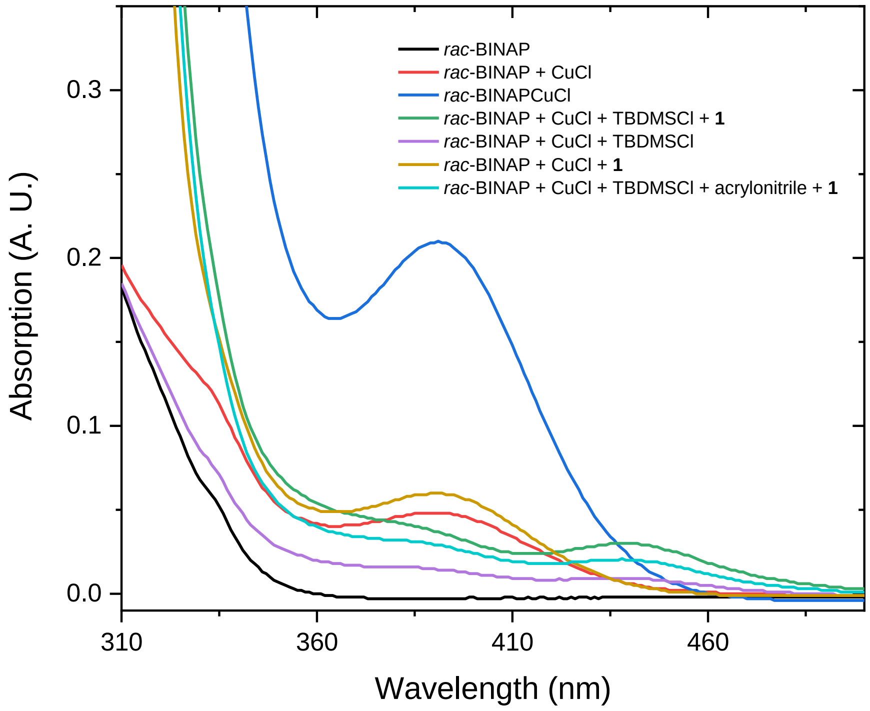

Figure S2. UV-Vis absorption spectra of *rac*-BINAP (black), *rac*-BINAP + CuCl (red), *rac*-BINAPCuCl (blue), *rac*-BINAP + CuCl + TBDMSCl + **1** (dark green), *rac*-BINAP + CuCl + TBDMSCl (purple), *rac*-BINAP + CuCl + **1** (gold) and *rac*-BINAP + CuCl + TBDMSCl + acrylonitrile in DMF.

Cyclic voltammograms

Cyclic voltammograms were recorded using an Autolab PGSTAT204 potentiostat and a Pt working electrode, an Ag/AgCl reference electrode and a Pt wire auxiliary electrode. The voltammograms were recorded at room temperature in 0.1 M tetrabutylammonium tetrafluoroborate in DMF (3 mL, c = 3.3 mM) containing **1** (9.7 mg, 30 µmol), *rac*-**2a** (6.65 mg, 30 µmol) and *rac*-**2b** (8.00 mg, 30 µmol). The scan rate was 1 $mV \cdot s^{-1}$.

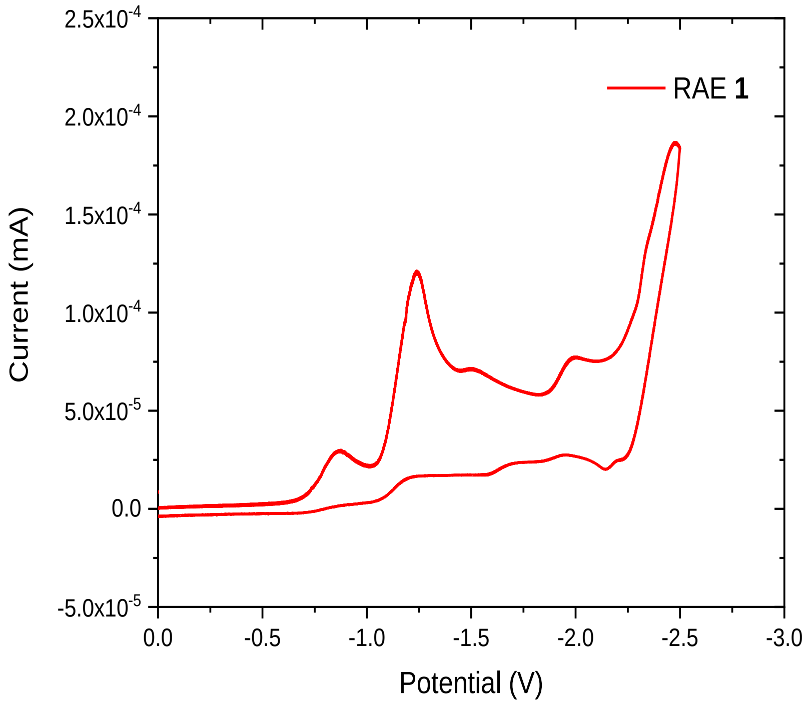

Figure S3. Cyclic voltammetry of RAE **1**. E_p= –1.2 V.

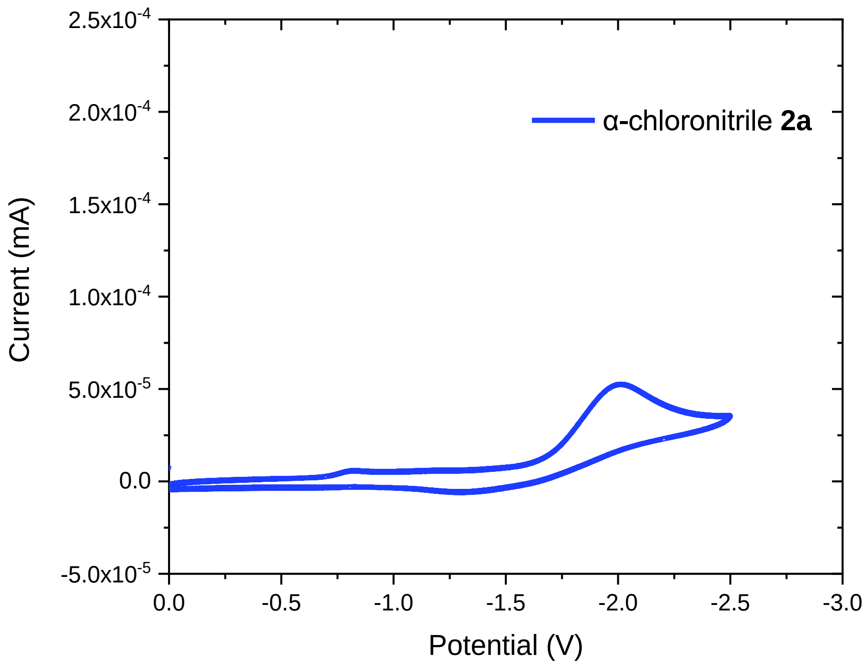

Figure S4. Cyclic voltammetry of *rac*-α-chloronitrile **2a**. E_p= –2.0 V.

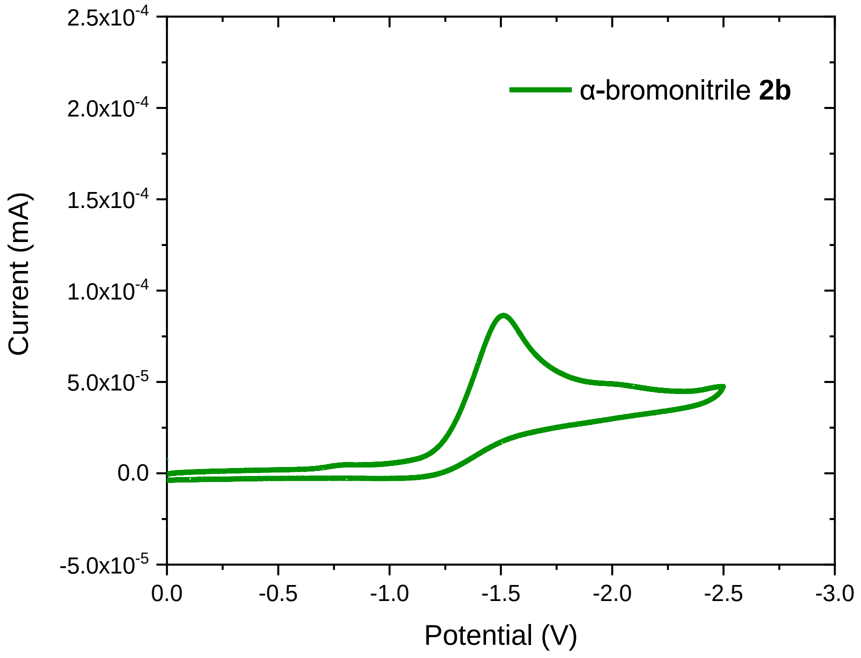

Figure S5. Cyclic voltammetry of *rac*-α-bromonitrile **2b**. E_p = –1.5 V.

Stern-volmer luminescence quenching studies

Visible light luminescence intensities were recorded using an Edinburgh Instruments FS5 spectrofluorometer. All luminescence measurements were recorded using a screw-top quartz cuvette (Hellma fluorescence quartz cuvette, 10 × 10 mm, 3.5 mL). All solutions of *rac*-BINAPCuCl, RAE **1** and TBDMSCl were prepared in DMF. The solutions were transferred to the screw-top cuvette, the cuvette was sealed, and then placed in the Instrument for visible light luminescence measurements. In a typical procedure, RAE **1** (161 mg, 0.50 mmol) was dissolved and diluted to a final volume of 10 mL (c = 50.0 mM) with a stock solution of *rac*-BINAPCuCl in DMF (c = 0.50 mM). Serial dilution of this 50.0 mM RAE **1** solution was carried out by starting with taking out 3 mL of 50.0 mM RAE **1** and replacing it with another 3 mL of the stock solution of *rac*-BINAPCuCl in DMF (c = 0.50 mM) for each measurement. All subsequent solutions were prepared by the same procedure. All solutions were excited at 450 nm and the emission was measured from 470 to 800 nm.

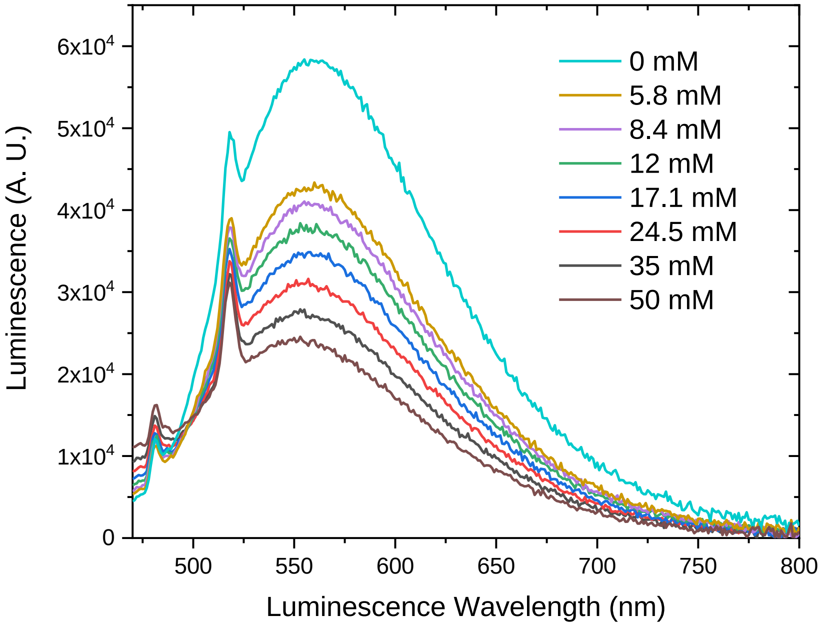

Figure S6. Emission spectra for *rac*-BINAPCuCl luminescence quenching by RAE **1**.

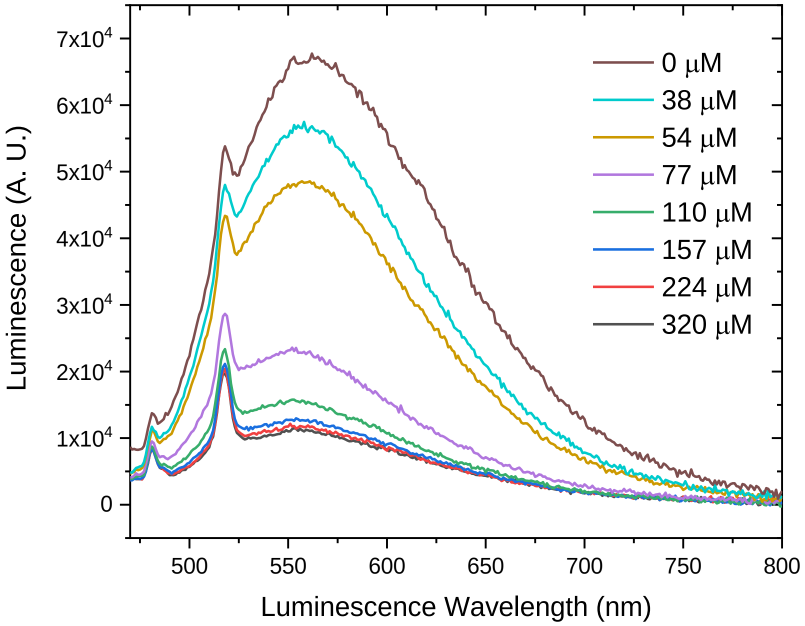

Figure S7. Emission spectra for *rac*-BINAPCuCl luminescence quenching by TBDMSCl + RAE **1**.

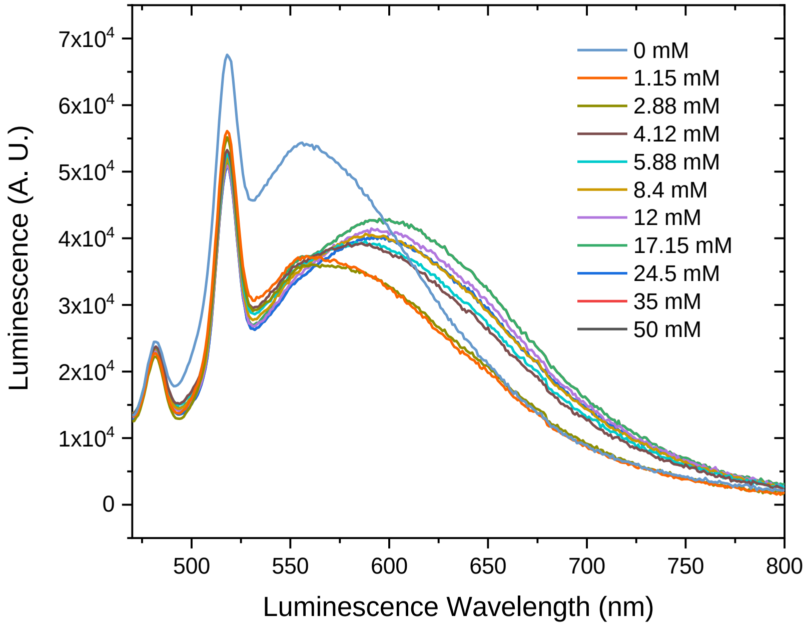

Figure S8. Emission spectra for *rac*-BINAPCuCl luminescence quenching by TBDMSCl.

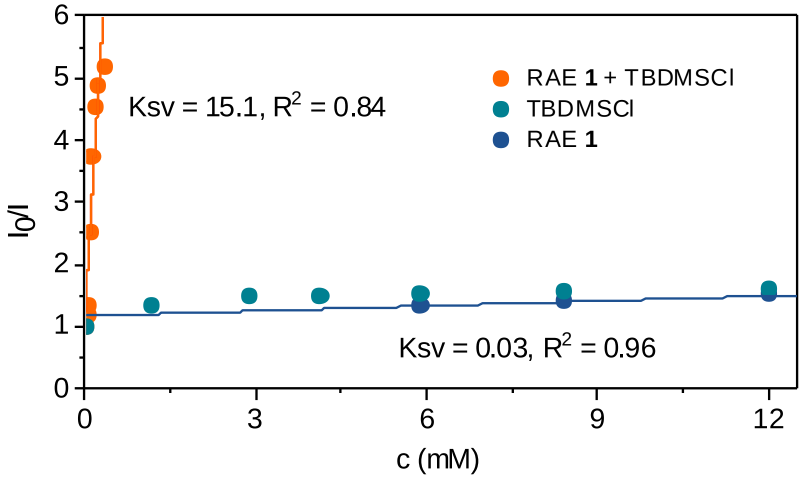

Figure S9. Stern-Volmer plots for the luminescence quenching of *rac*-BINAPCuCl by RAE **1** (with *Ksv*= 0.03 mM^{-1}) and TBDMSCl + RAE **1** (with *Ksv*= 15.1 mM^{-1}).

SPECTROSCOPIC DATA

^{1}H NMR of *rac*-α-chloronitrile 2a

$CDCl_3$, 23 °C

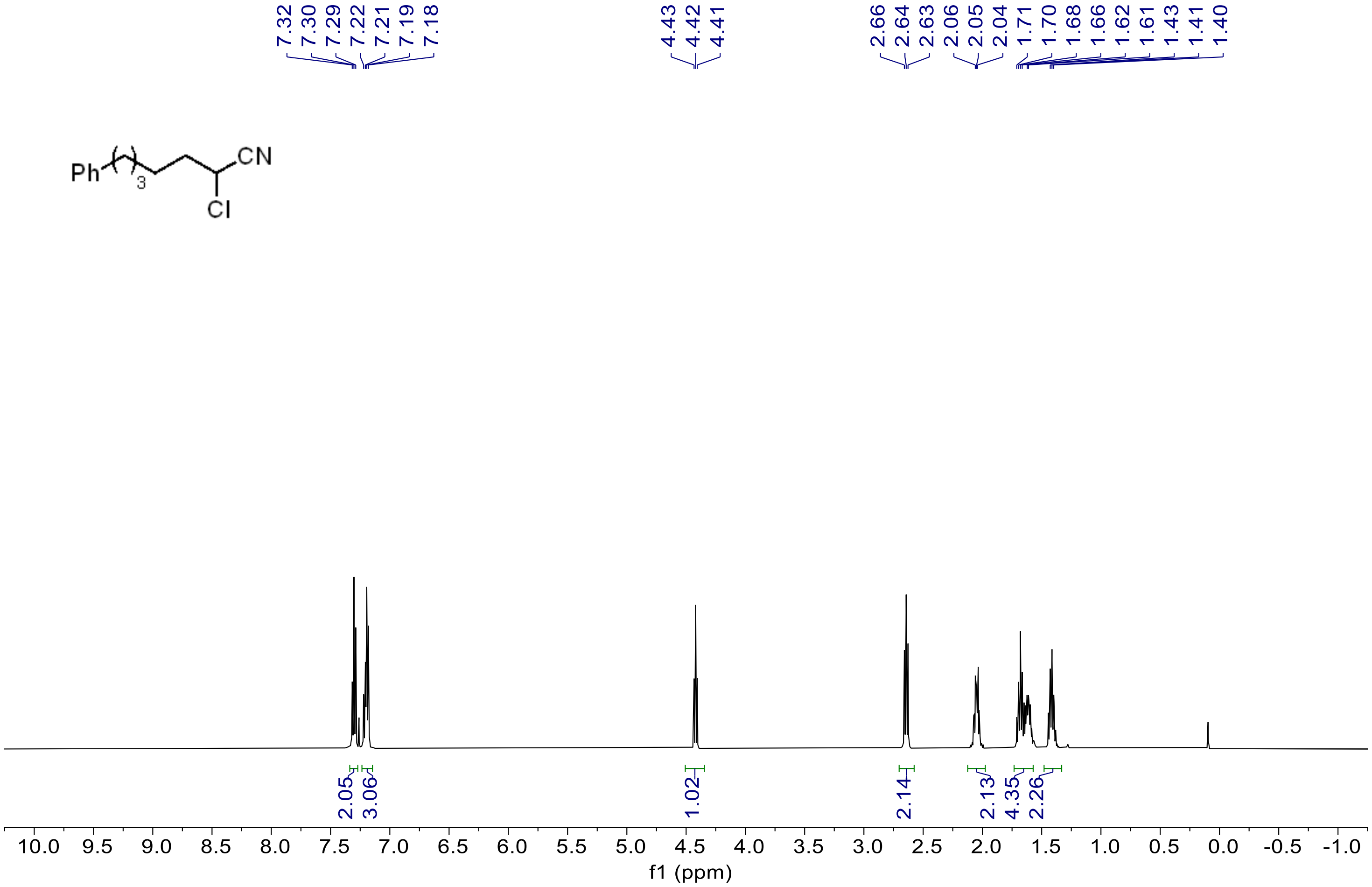

^{13}C NMR of *rac*-α-chloronitrile 2a

$CDCl_3$, 23 °C

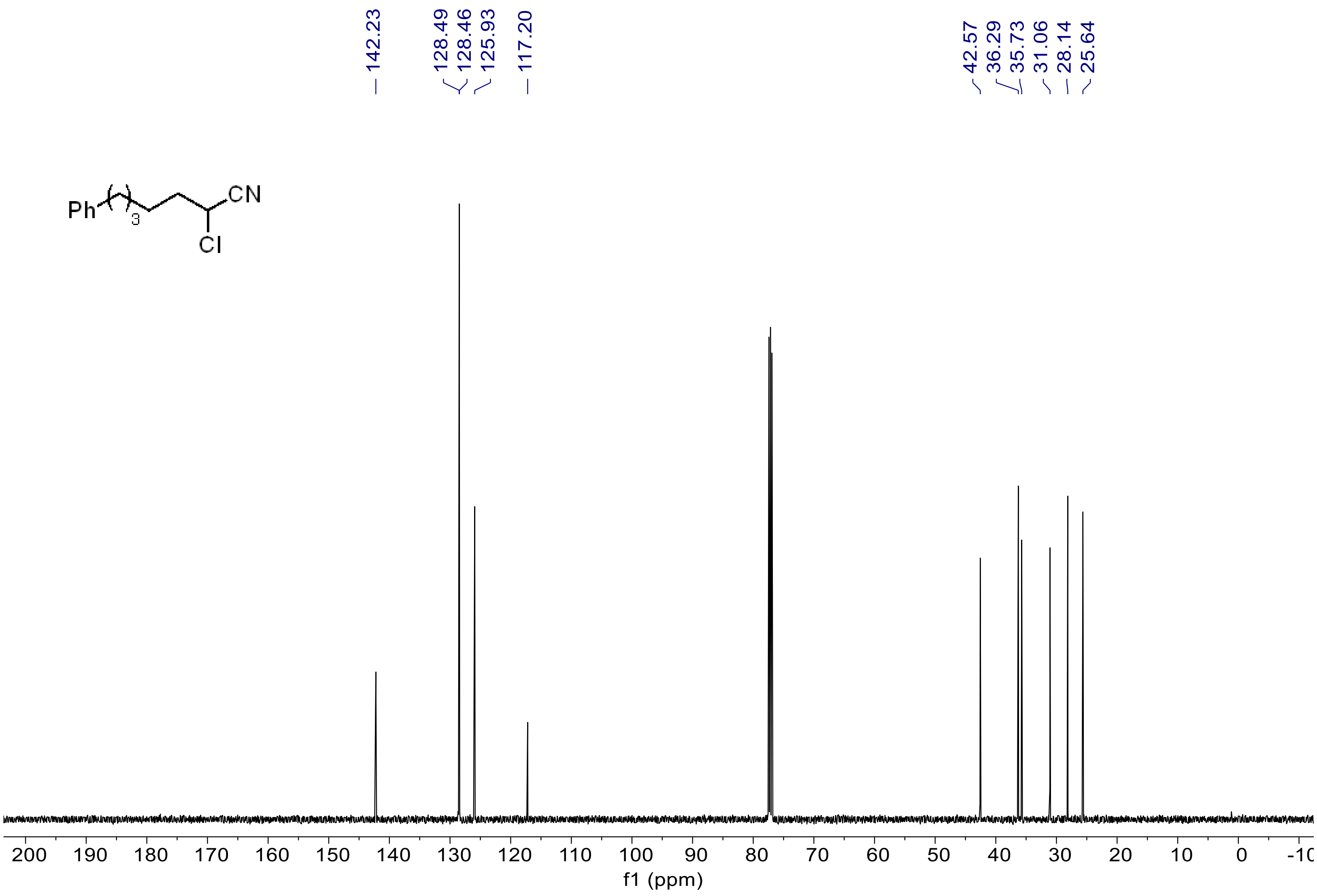

^{1}H NMR of *rac*-α-bromonitrile 2b

$CDCl_3$, 23 °C

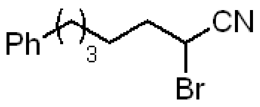

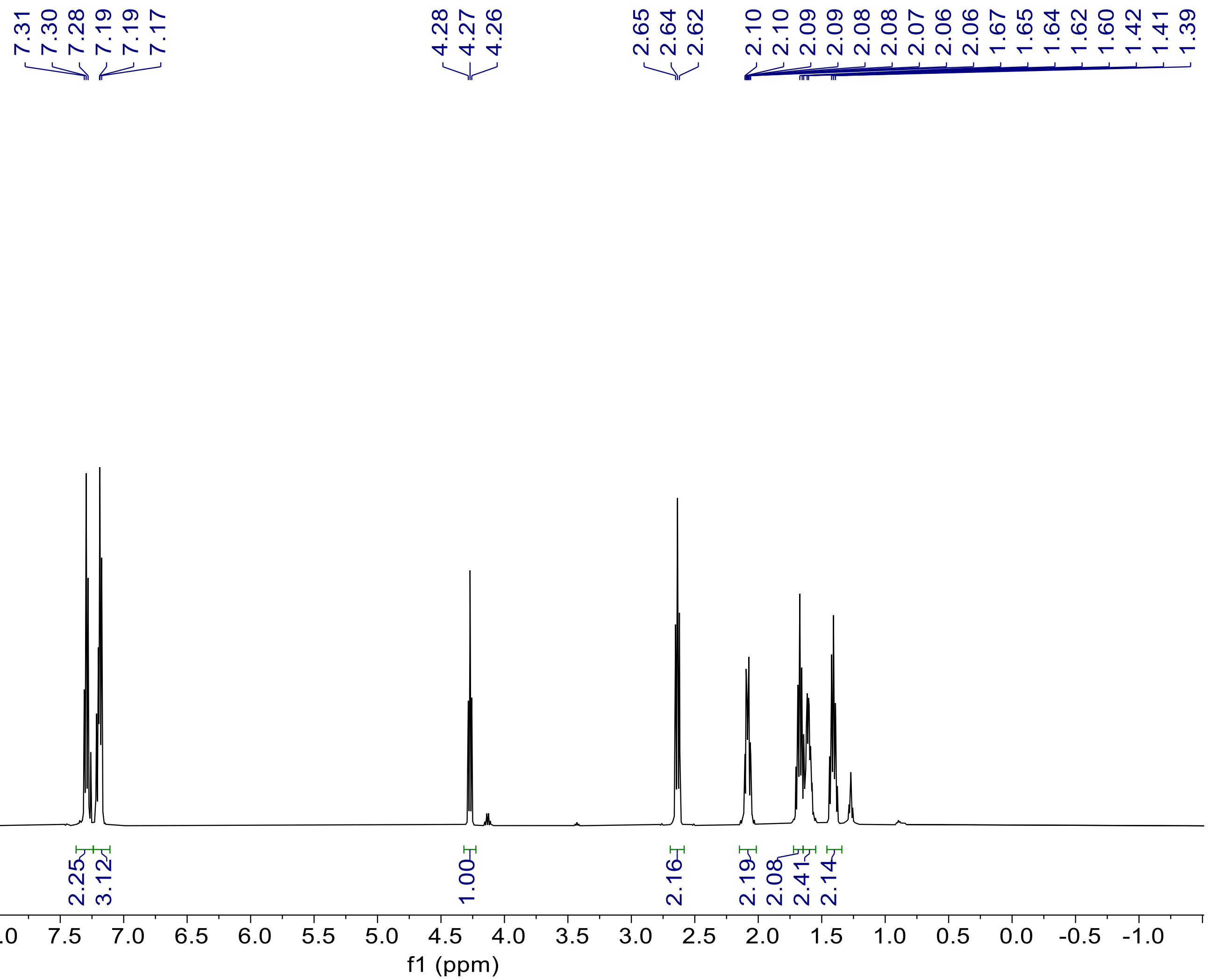

^{13}C NMR of *rac*-α-bromonitrile 2b

$CDCl_3$, 23 °C

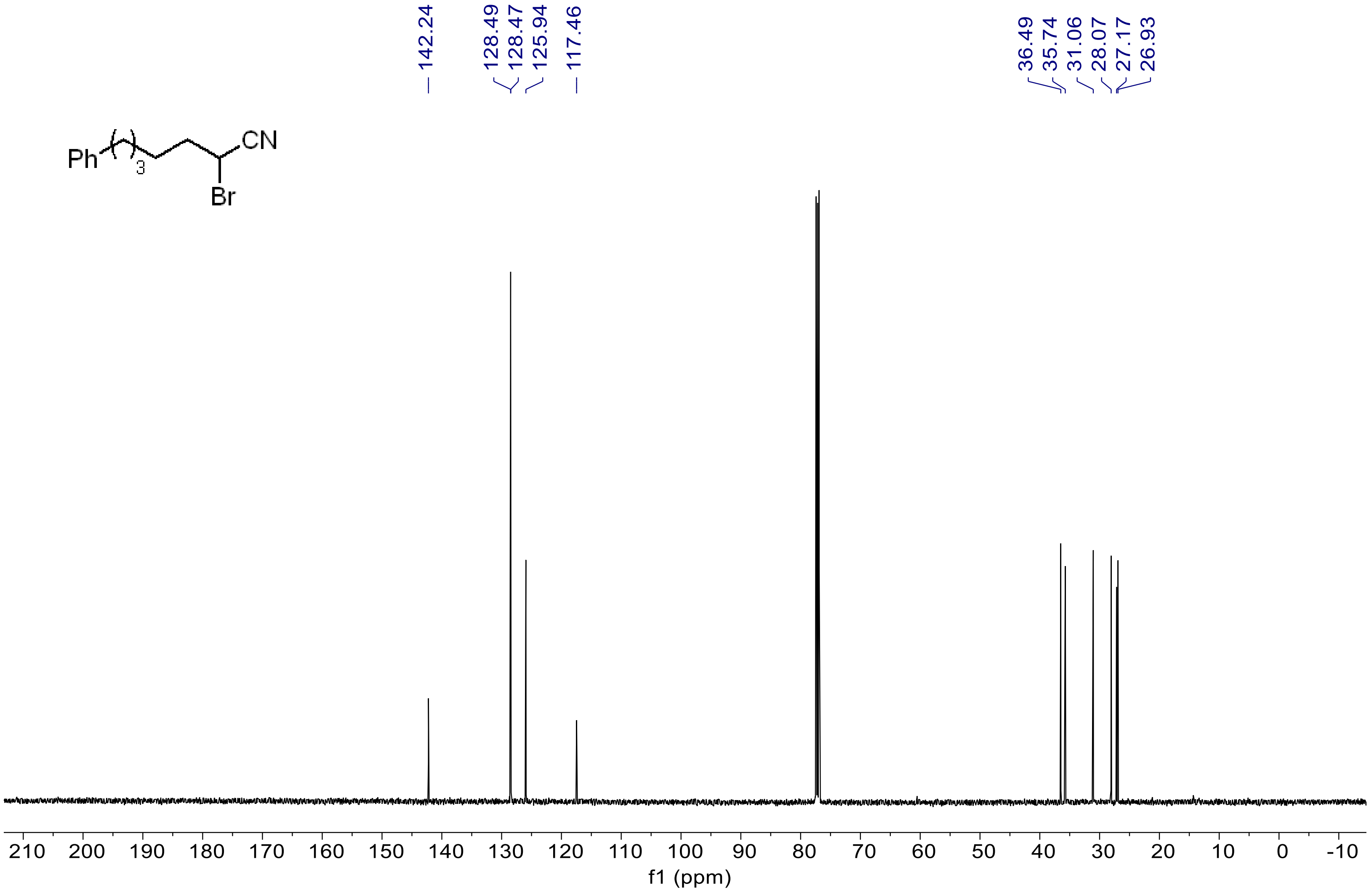

^{1}H NMR of *rac*-bicyclopentane-derived α-chloronitrile 3a

$CDCl_3$, 23 °C

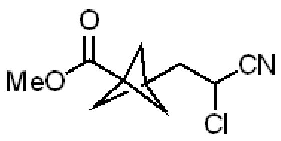

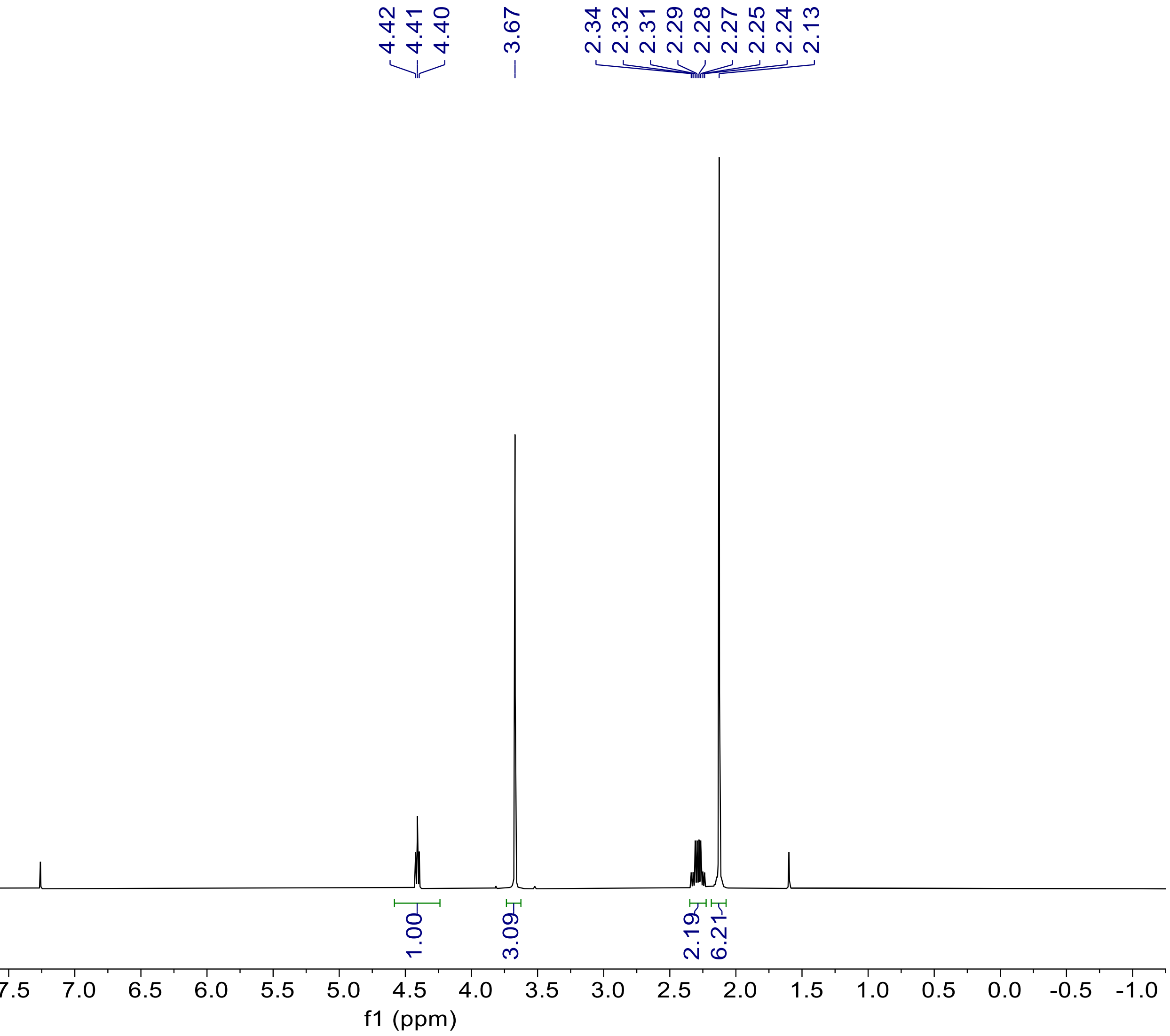

^{13}C NMR of *rac*-bicyclopentane-derived α-chloronitrile 3a

$CDCl_3$, 23 °C

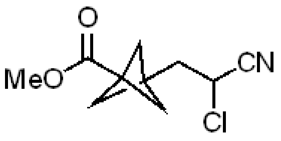

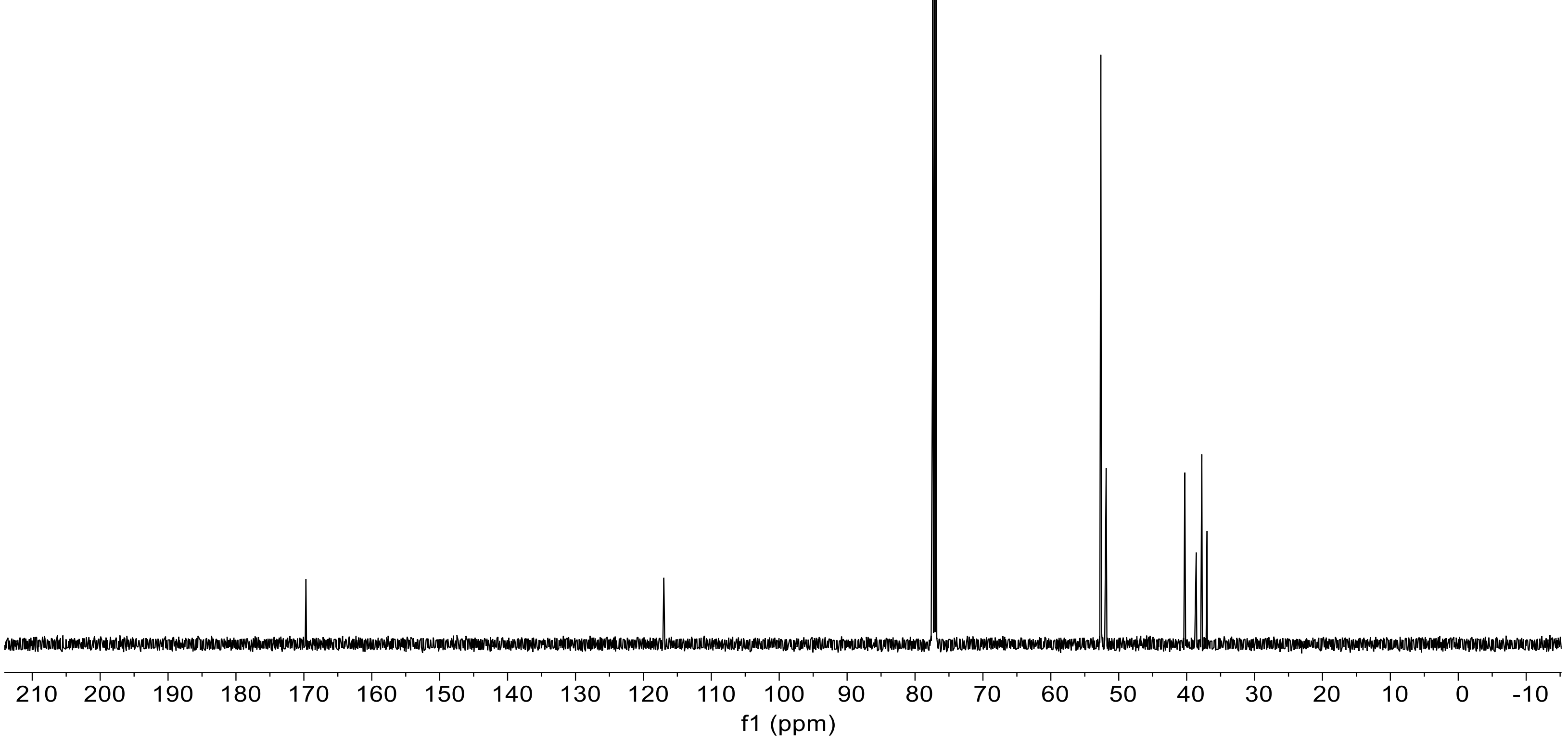

^{1}H NMR of *rac*-bicyclopentane-derived α-bromonitrile 3b

$CDCl_3$, 23 °C

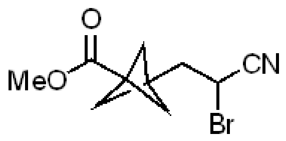

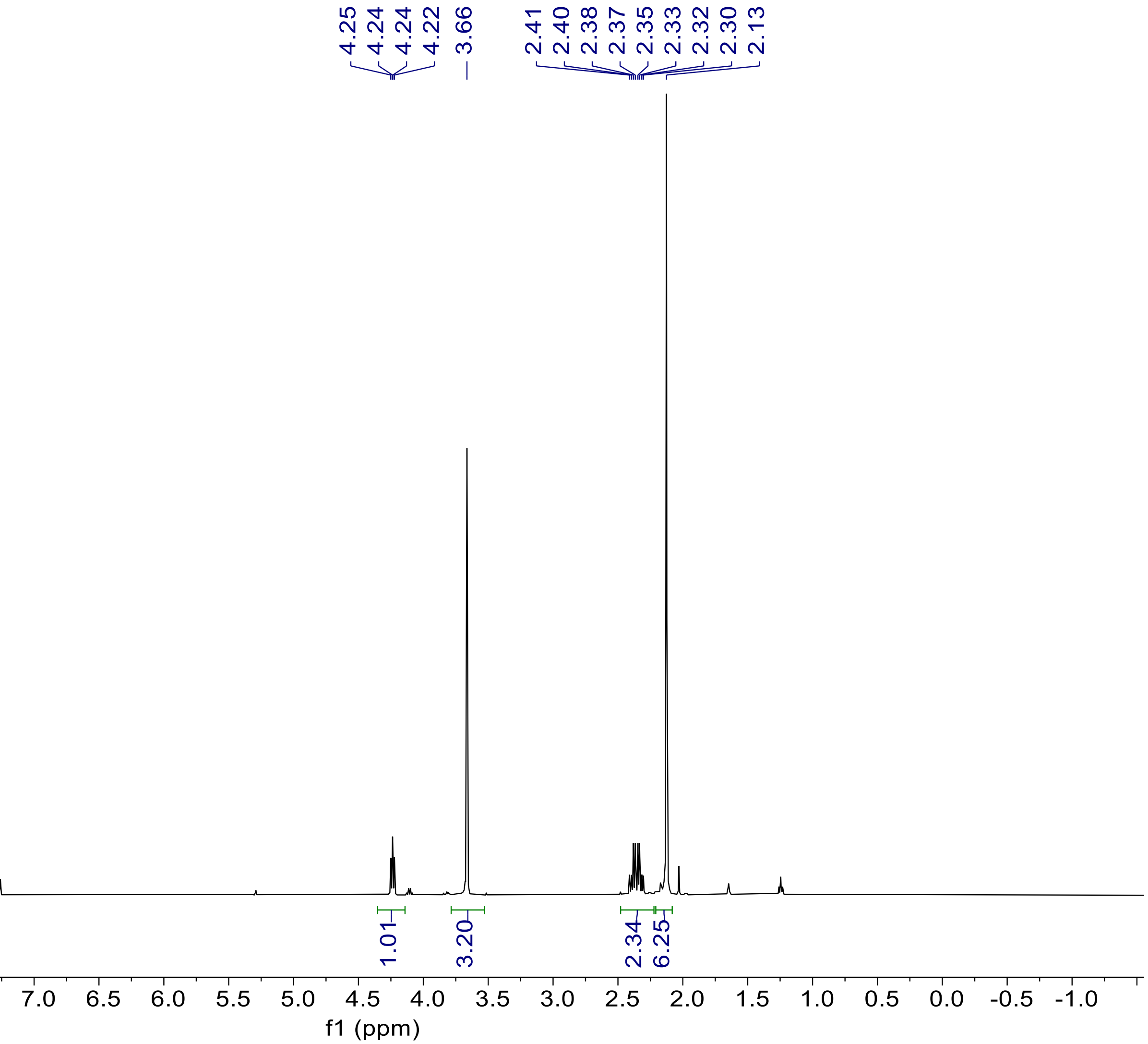

^{13}C NMR of *rac*-bicyclopentane-derived α-bromonitrile 3b

$CDCl_3$, 23 °C

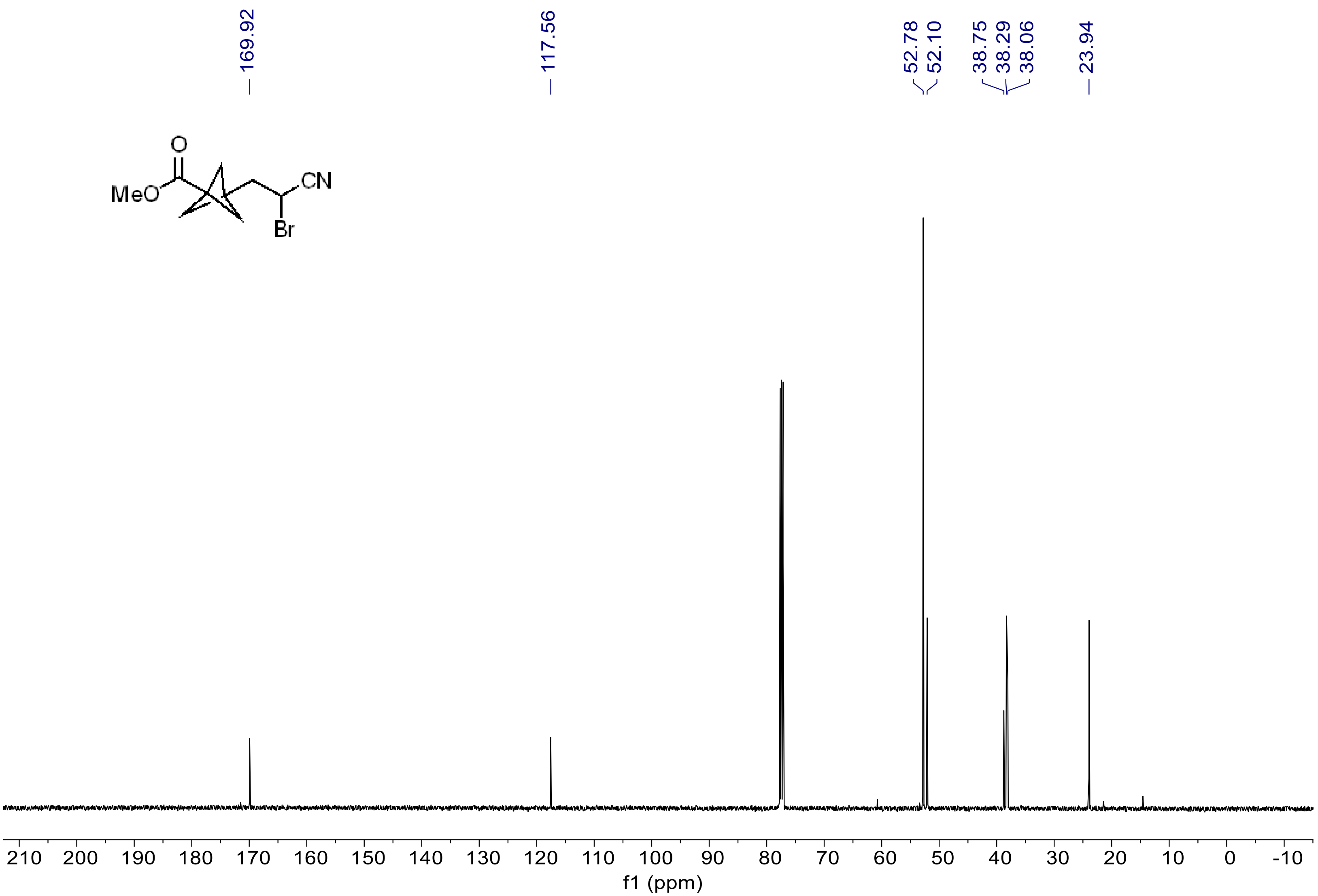

^{1}H NMR of *rac*-cubane-derived α-chloronitrile 4

$CDCl_3$, 23 °C

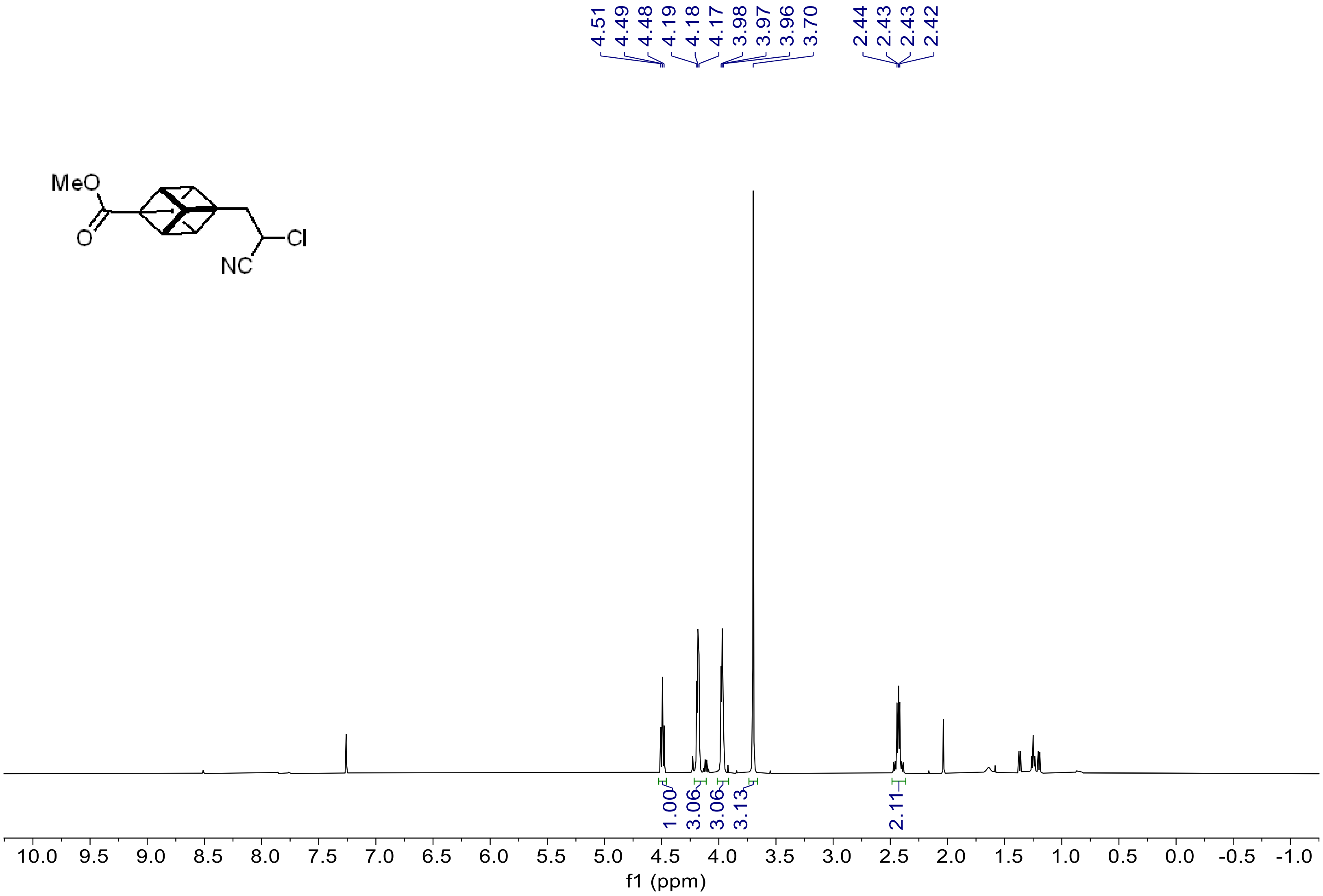

^{13}C NMR of *rac*-cubane-derived α-chloronitrile 4

$CDCl_3$, 23 °C

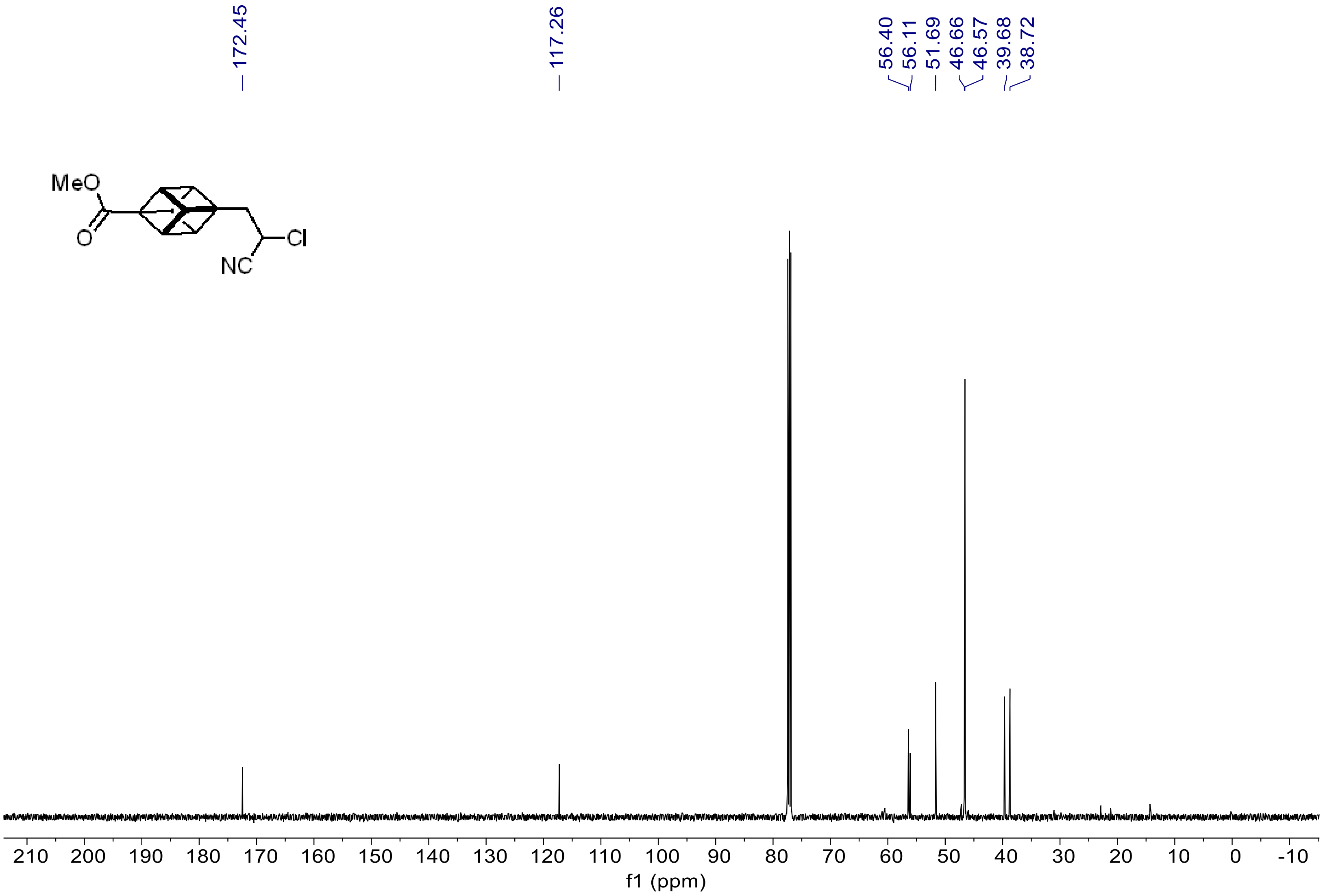

^{1}H NMR of *rac*-cyclopropane-derived α-chloronitrile 5

$CDCl_3$, 23 °C

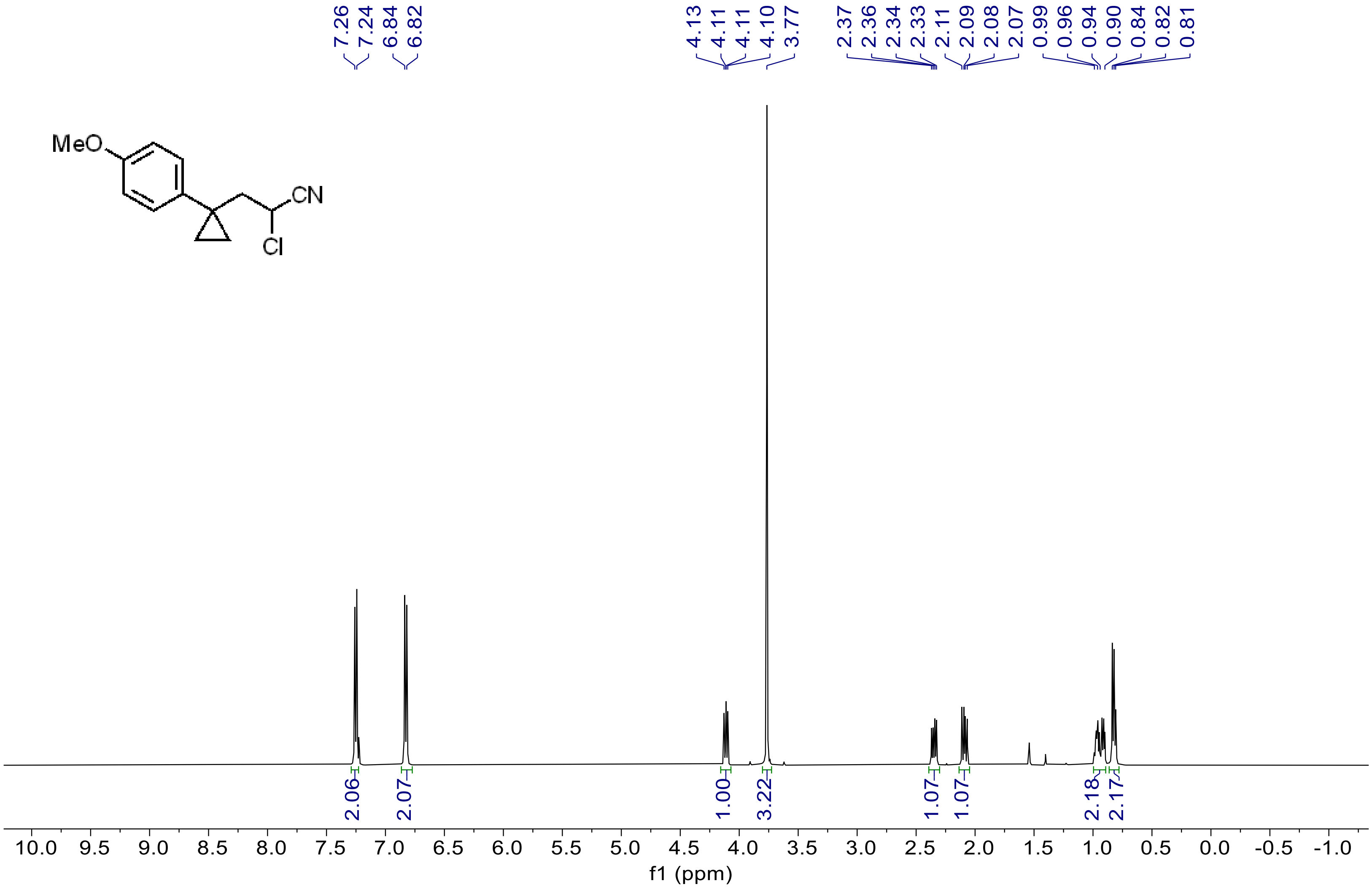

^{13}C NMR of *rac*-cyclopropane-derived α-chloronitrile 5

$CDCl_3$, 23 °C

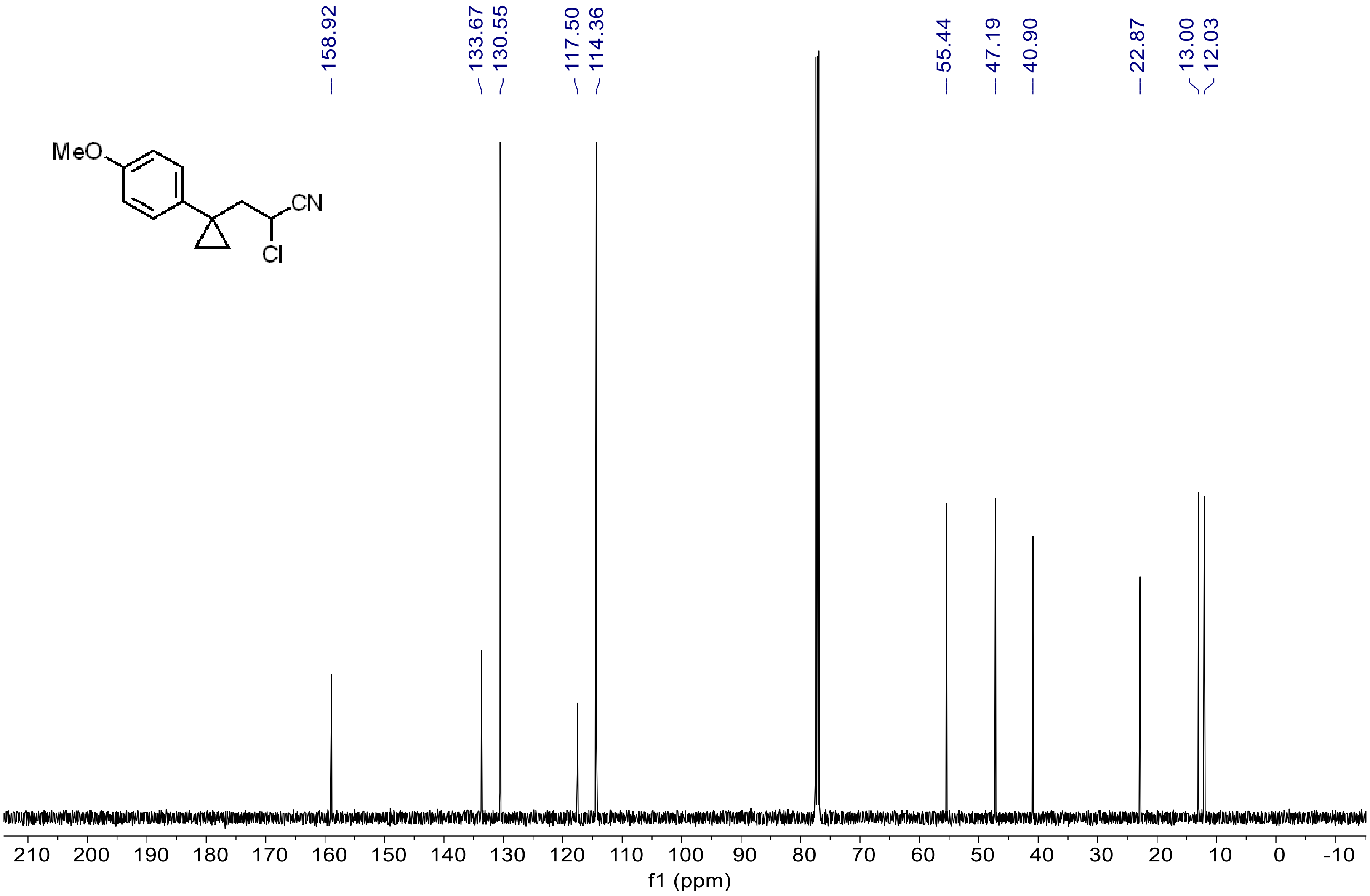

^{1}H NMR of *rac*-multifluoroalkyl-derived α-chloronitrile 6

$CDCl_3$, 23 °C

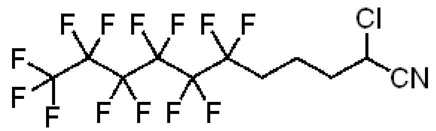

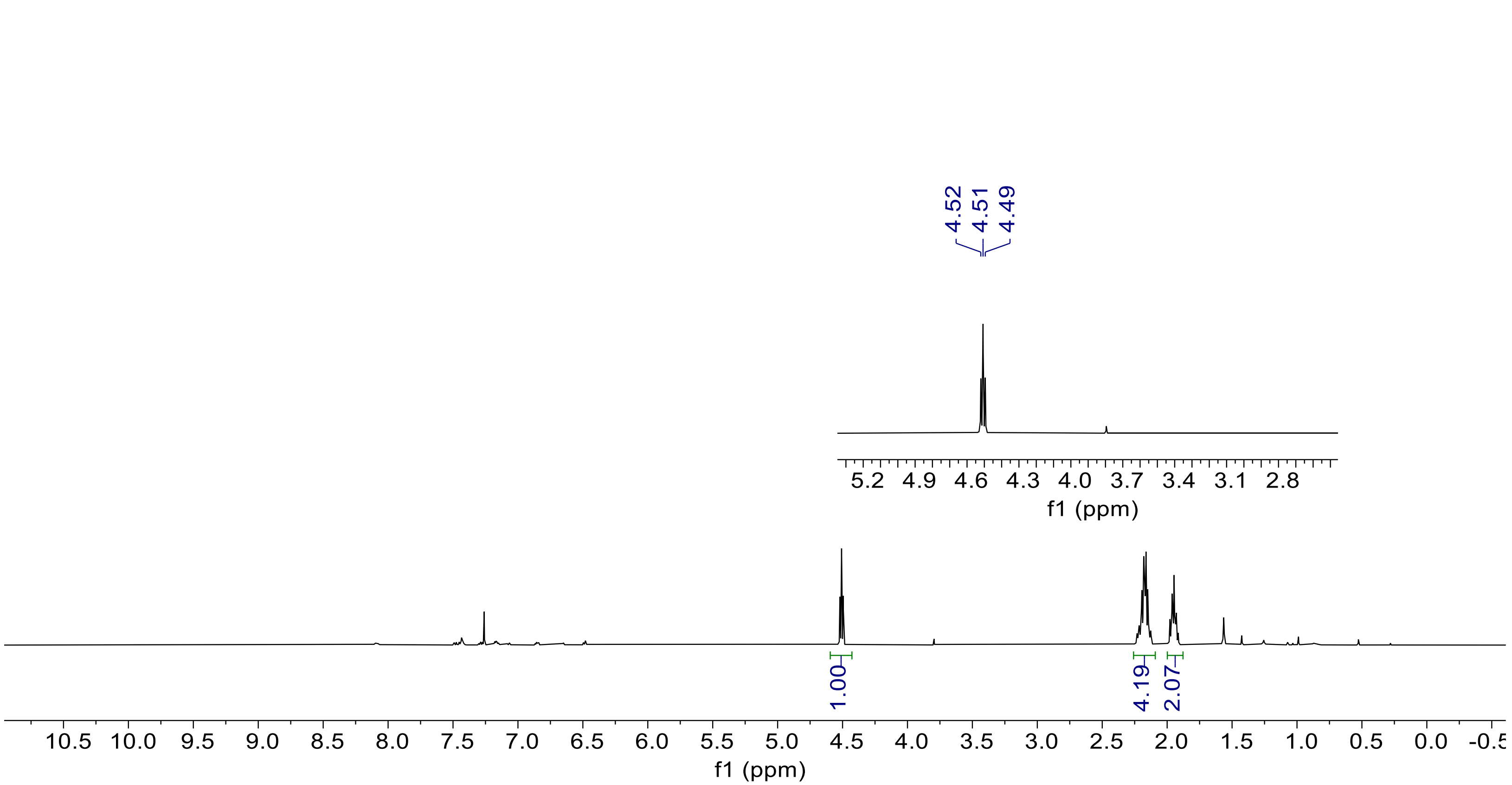

^{19}F NMR of *rac*-multifluoroalkyl-derived α-chloronitrile 6

$CDCl_3$, 23 °C

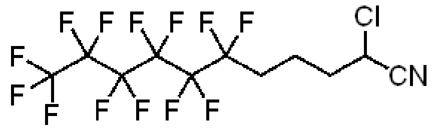

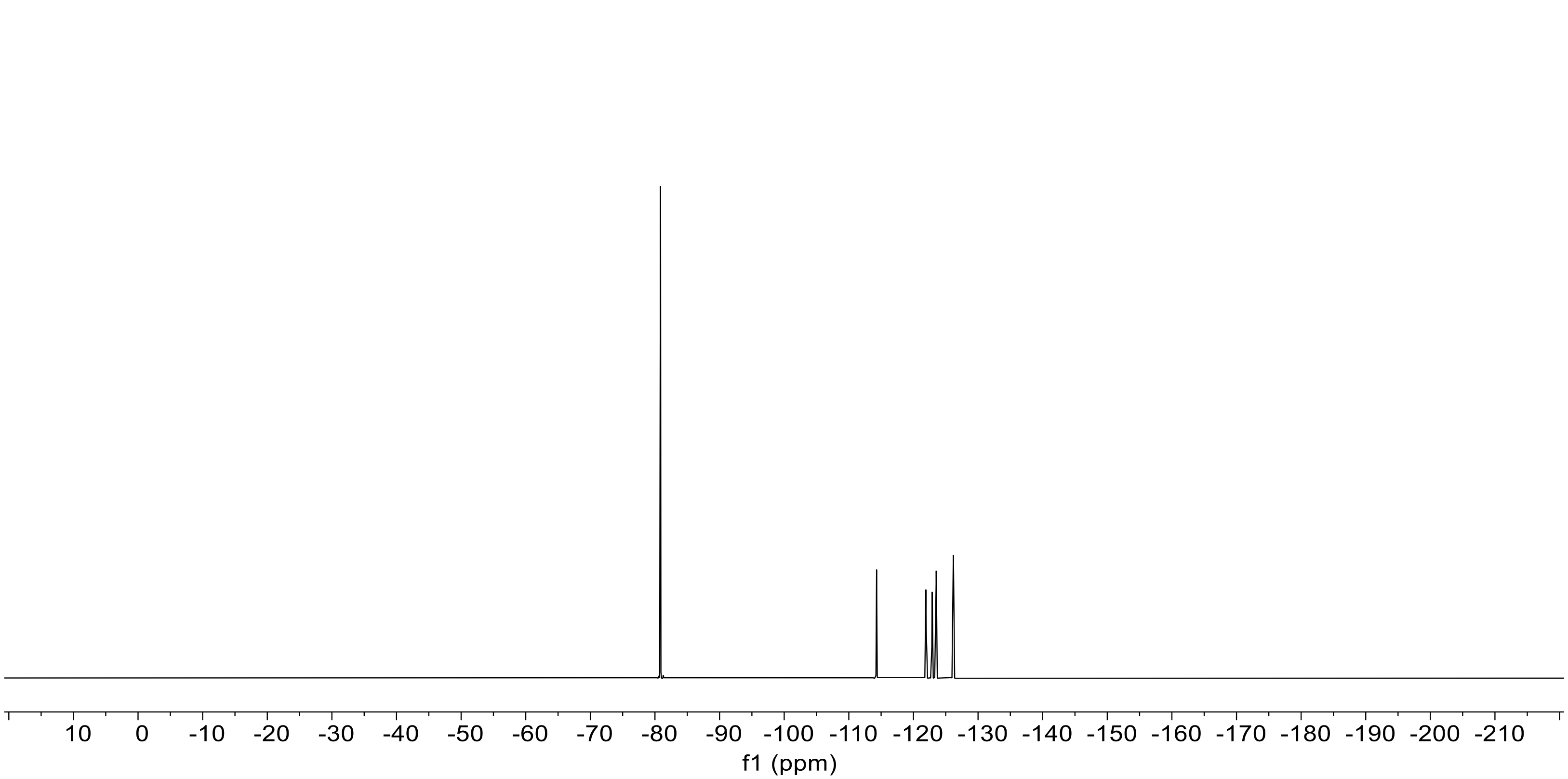

$^{13}C\{^{19}F\}$ NMR of *rac*-multifluoroalkyl-derived α-chloronitrile 6

CDCl₃, 23 °C

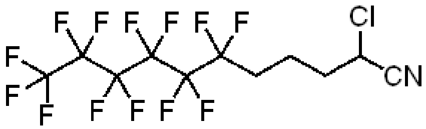

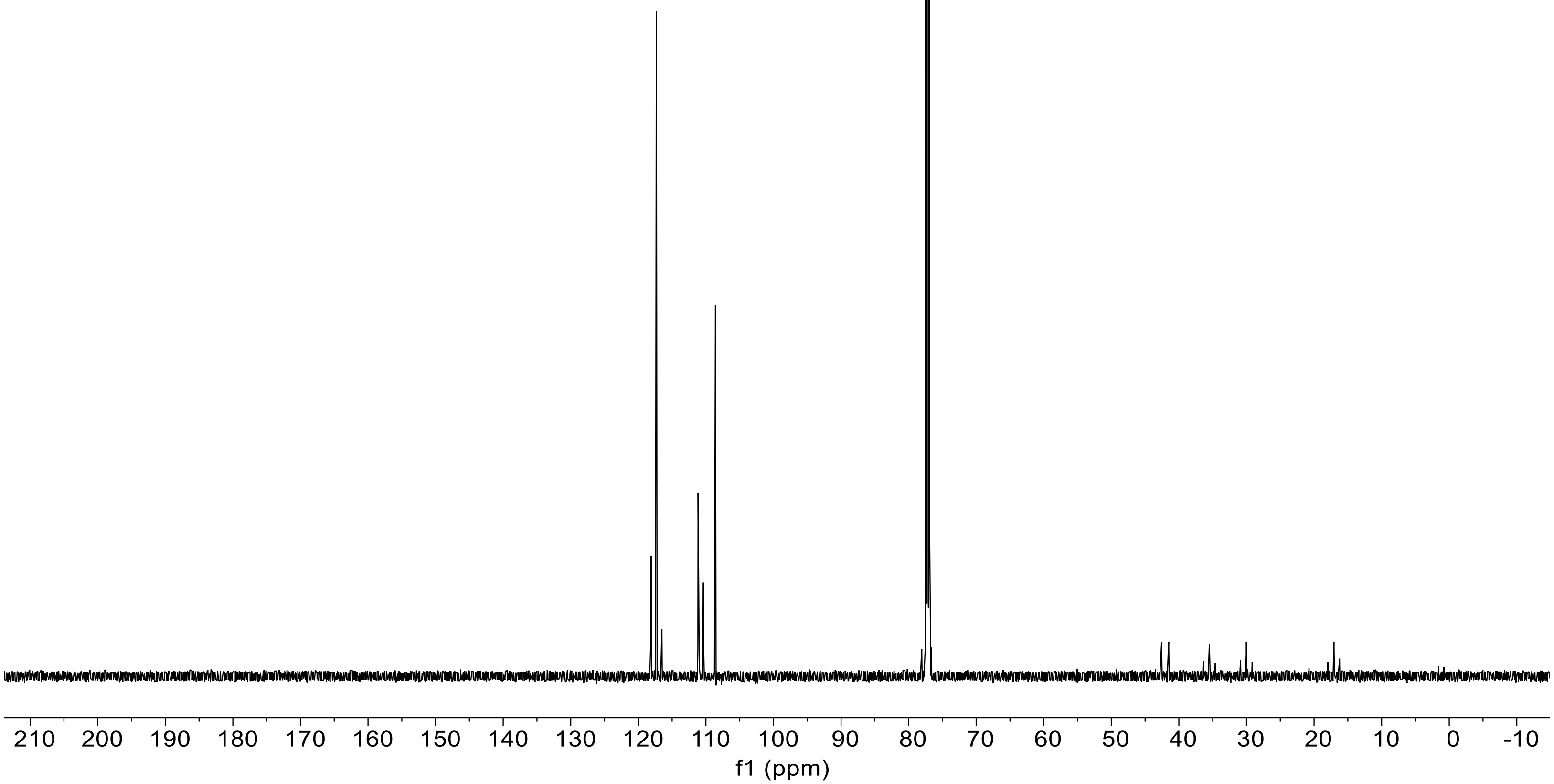

^{1}H NMR of *rac*-azetidine-derived α-chloronitrile 7

$CDCl_3$, 23 °C

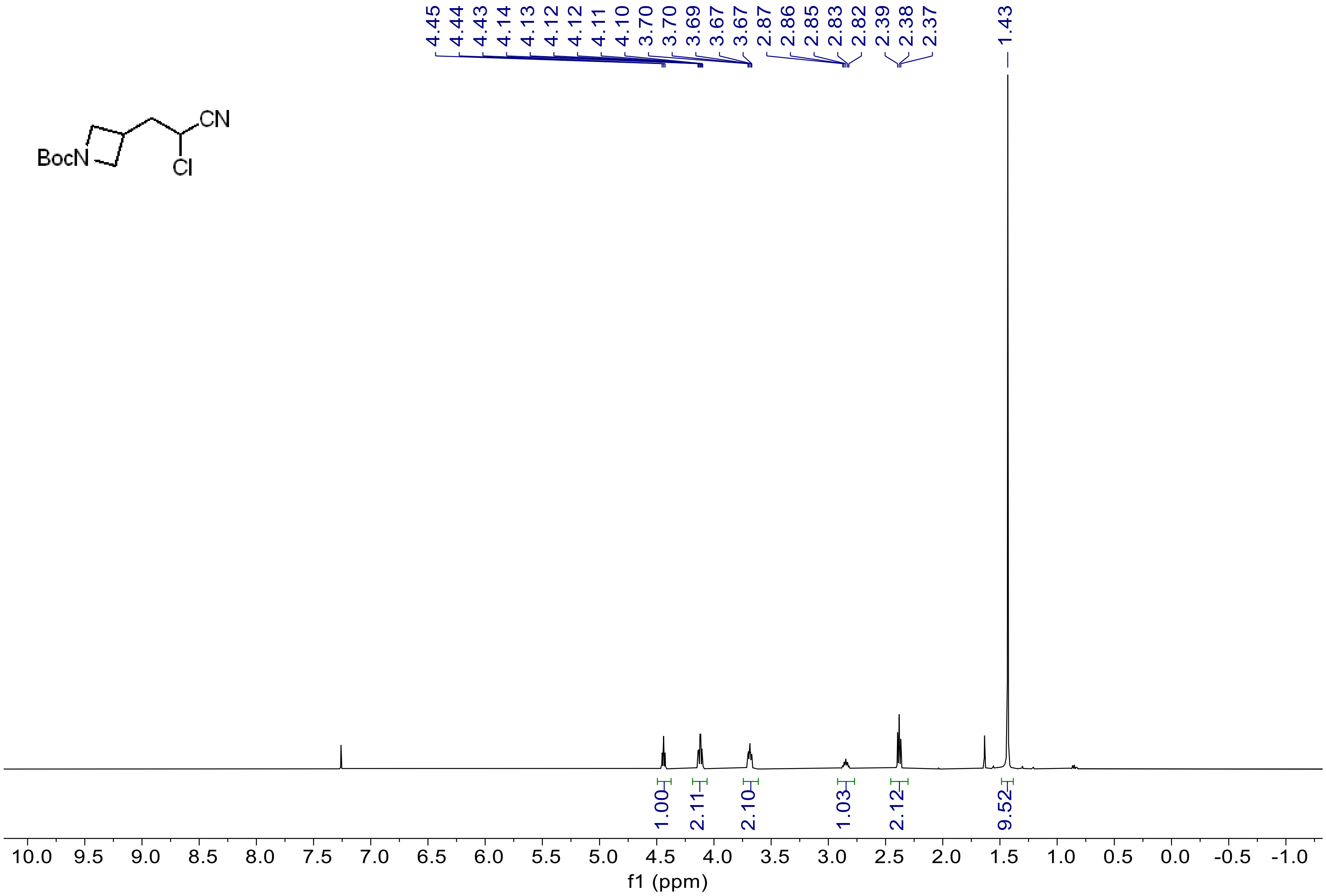

^{13}C NMR of *rac*-azetidine-derived α-chloronitrile 7

$CDCl_3$, 23 °C

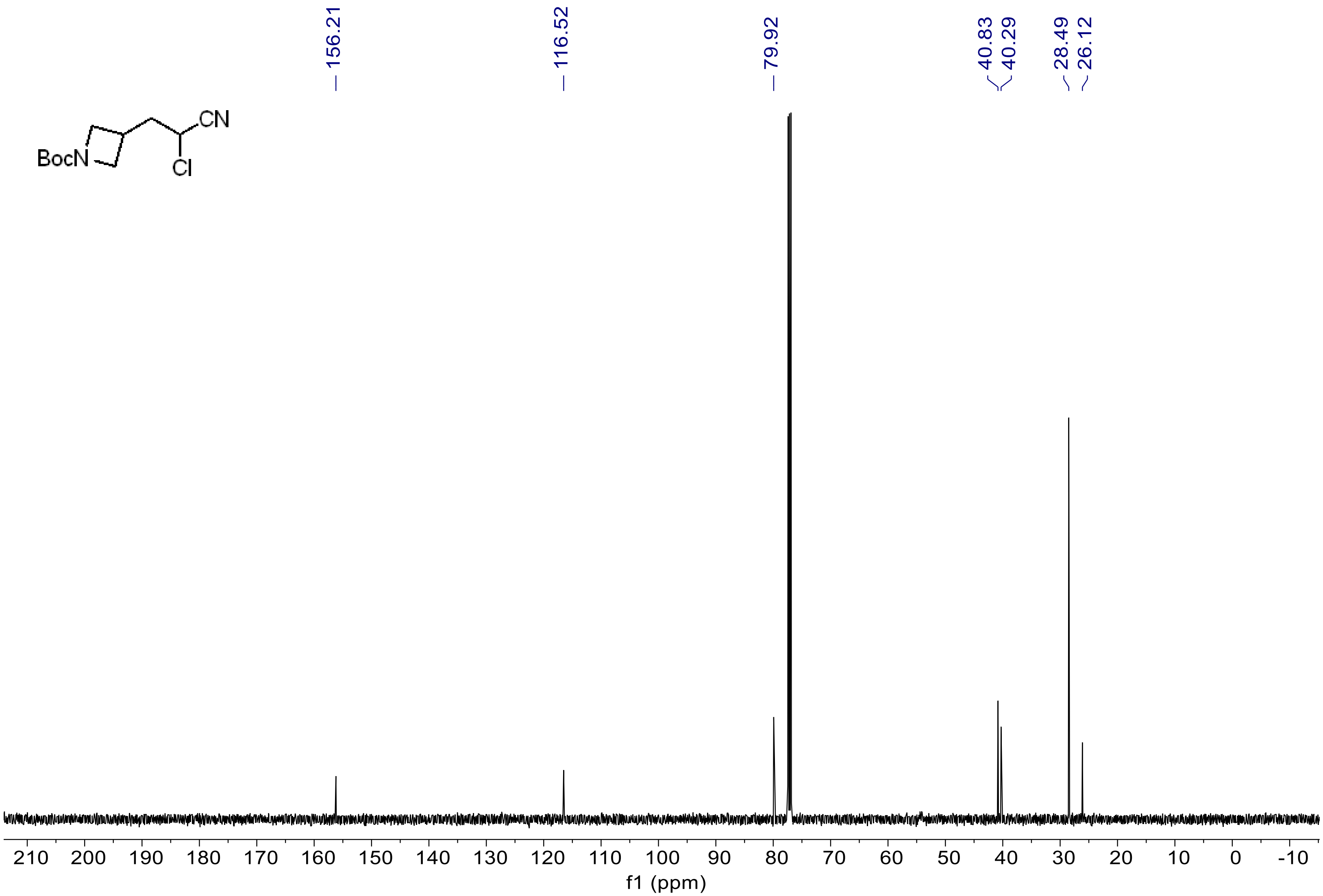

^{1}H NMR of *rac*-fluorochromane-derived α-chloronitrile 8

$CDCl_3$, 23 °C+

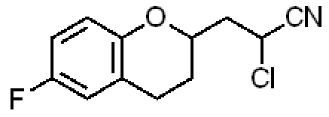

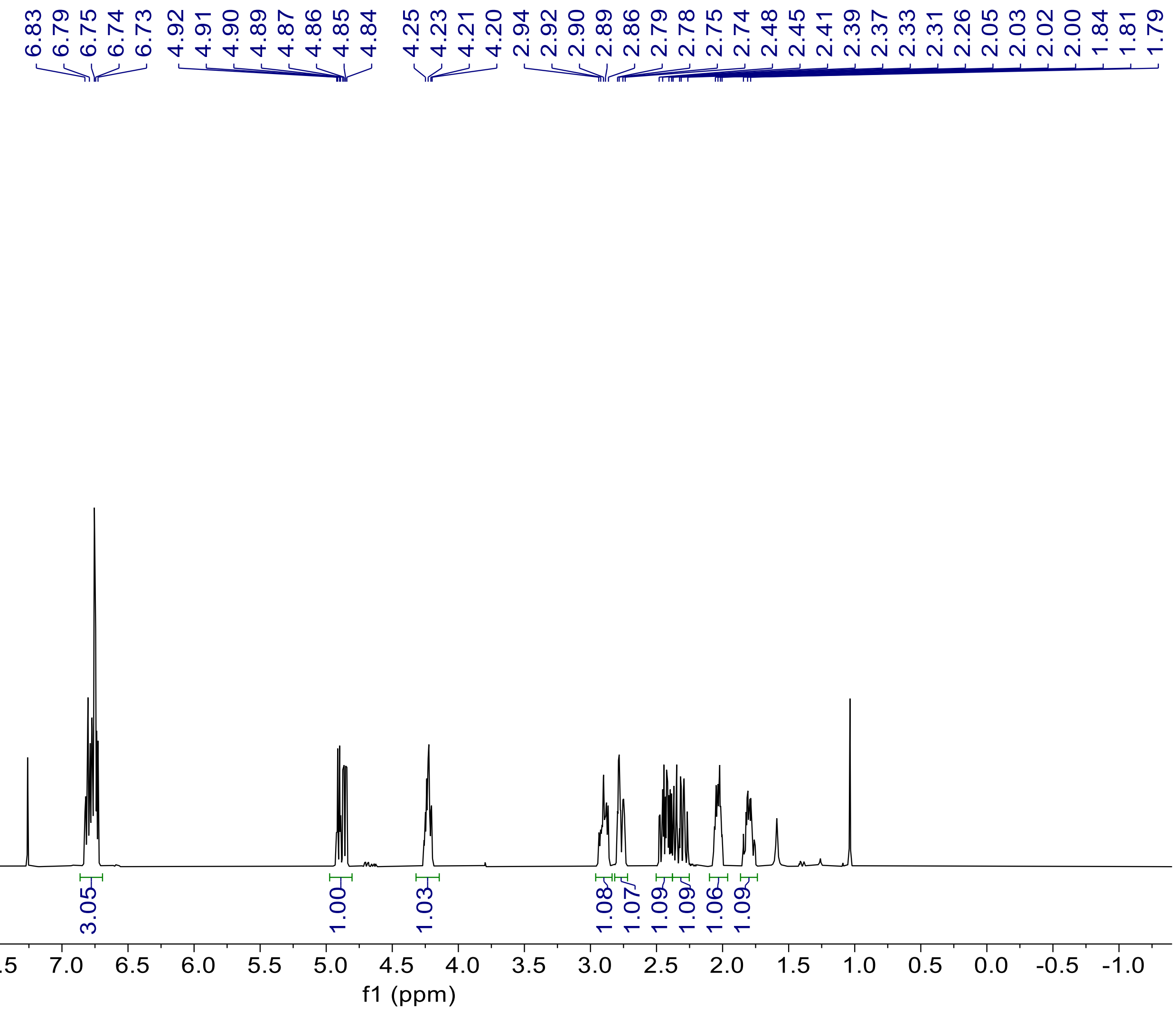

^{19}F NMR of *rac*-fluorochromane-derived α-chloronitrile 8

CDCl$_3$, 23 °C

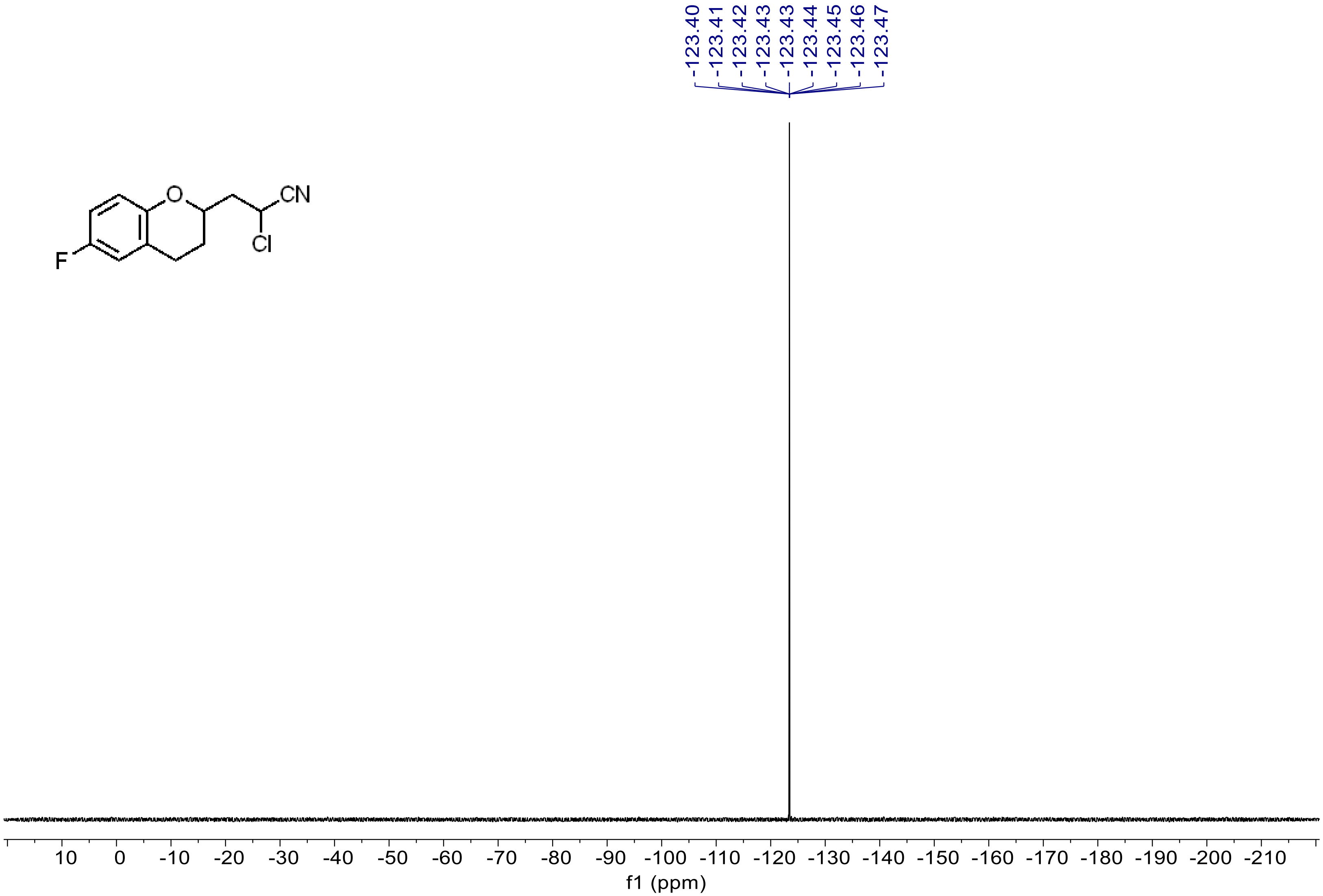

^{13}C NMR of *rac*-fluorochromane-derived α-chloronitrile 8

CDCl₃, 23 °C

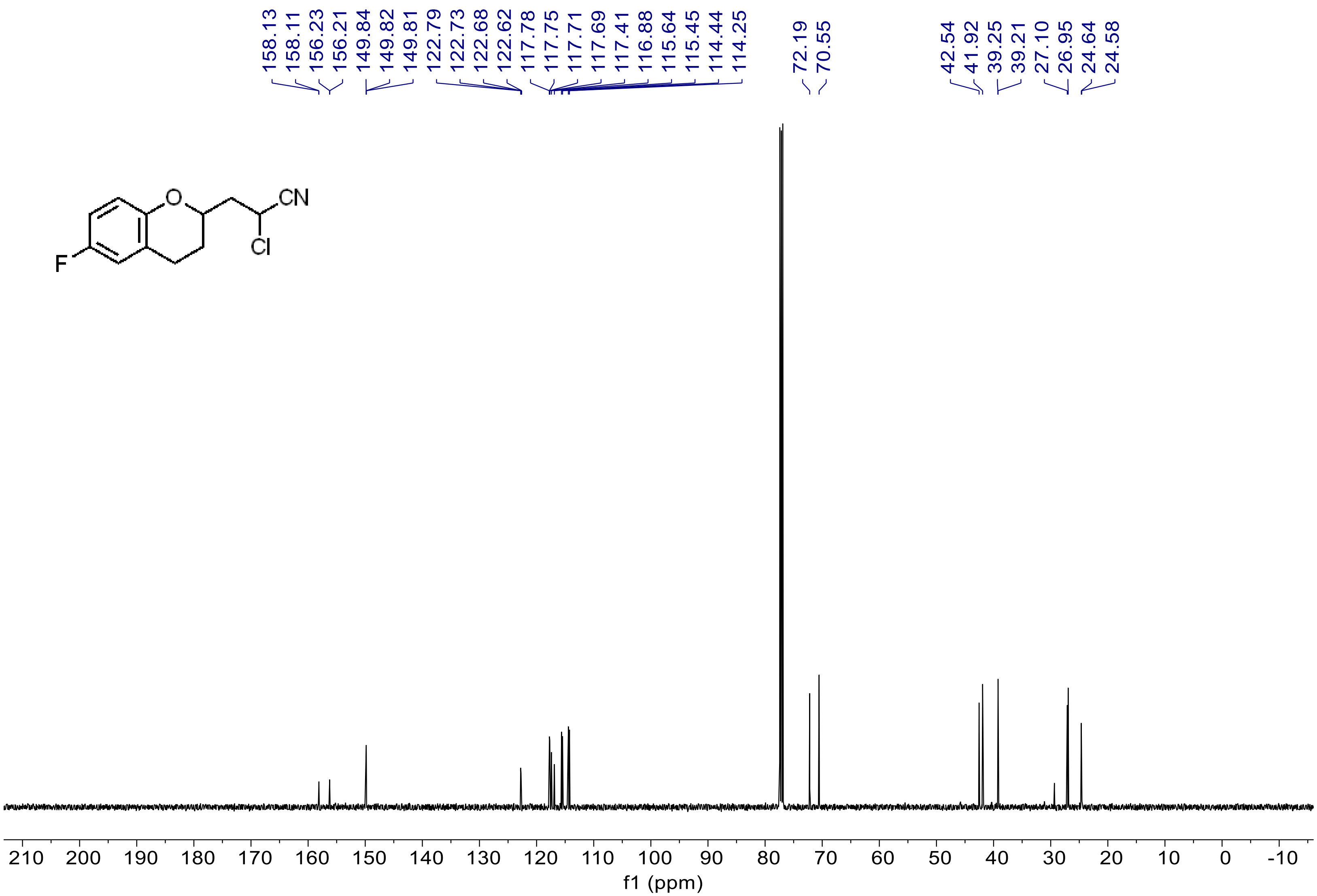

^{1}H NMR of *rac*-benzoylpiperidine-derived α-chloronitrile 9

CDCl$_3$, 23 °C

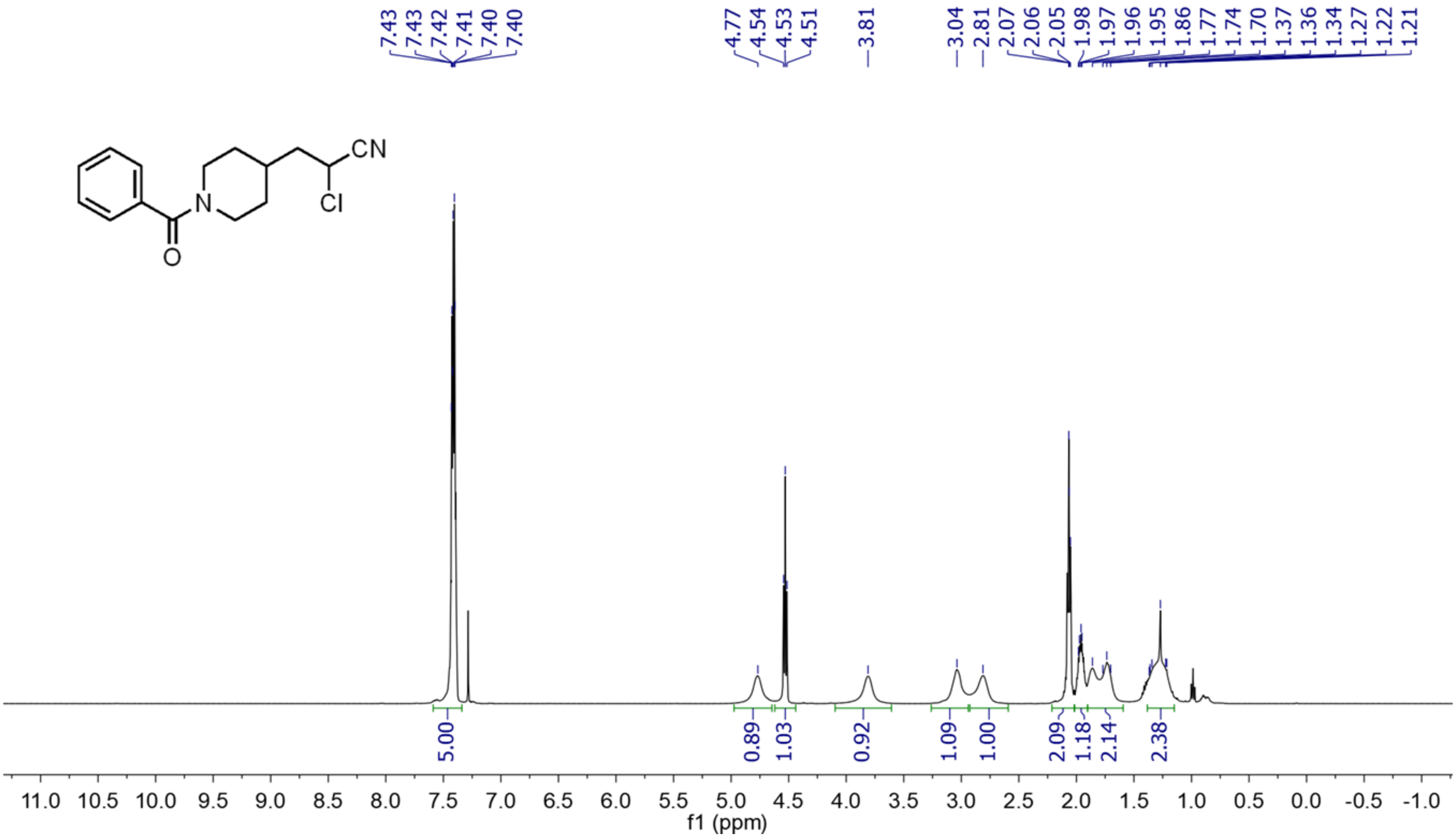

^{13}C NMR of *rac*-benzoylpiperidine-derived α-chloronitrile 9

$CDCl_3$, 23 °C

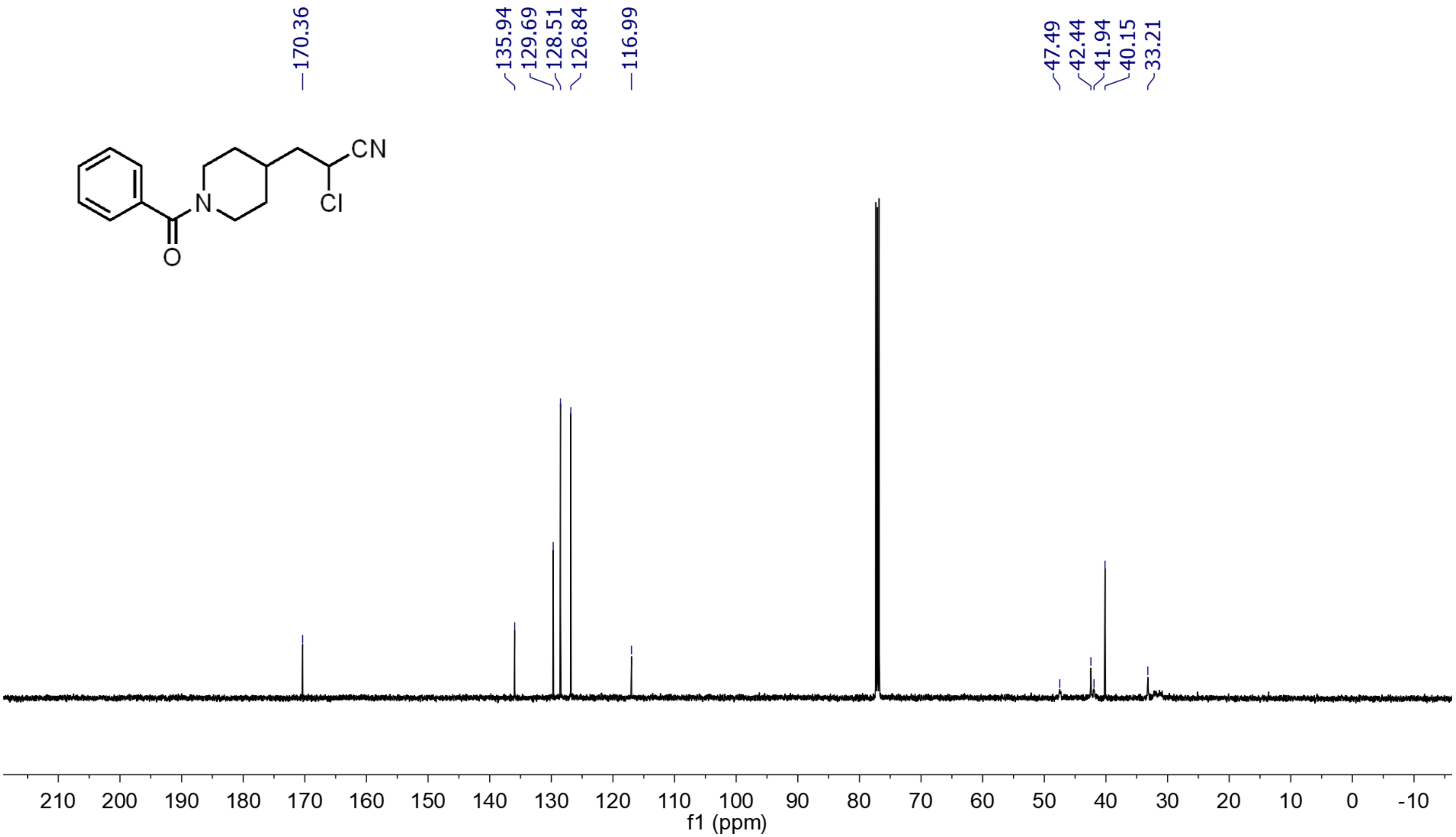

^{1}H NMR of *rac*-gemfibrozil-derived α-chloronitrile 10

$CDCl_3$, 23 °C

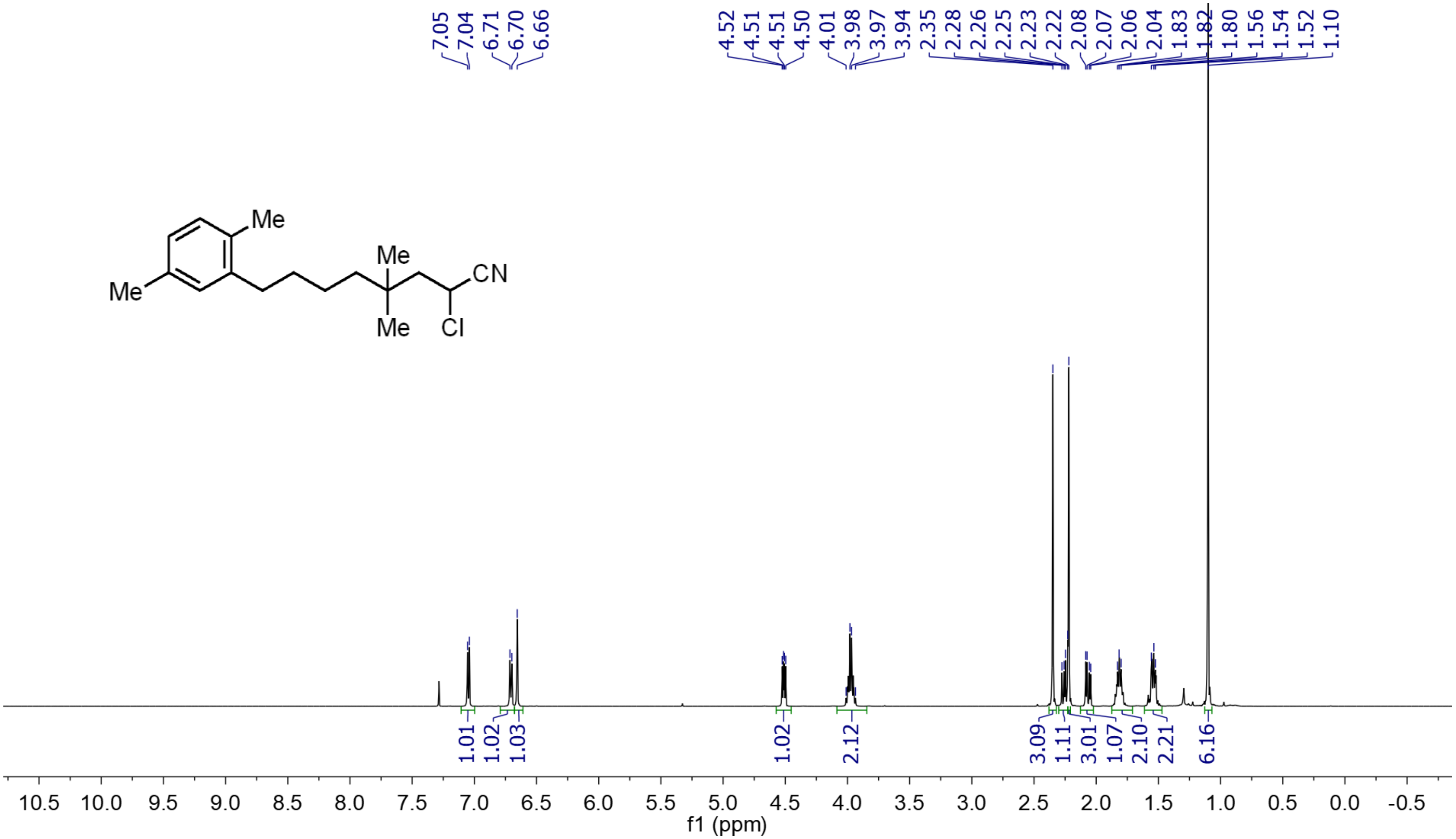

^{13}C NMR of *rac*-gemfibrozil-derived α-chloronitrile 10

$CDCl_3$, 23 °C

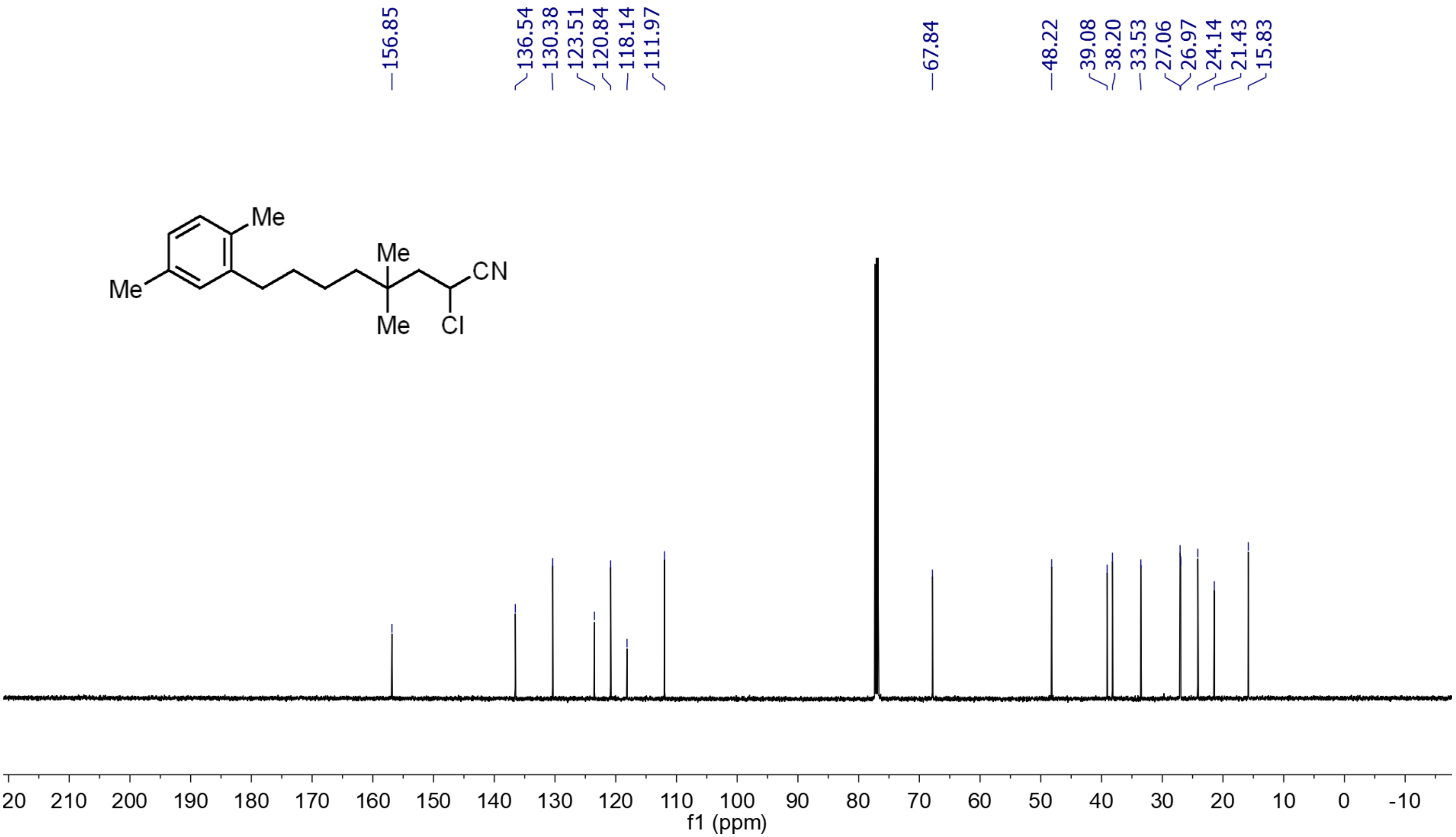

^{1}H NMR of *rac*-cyclopropane-derived α-chloronitrile 11

$CDCl_3$, 23 °C

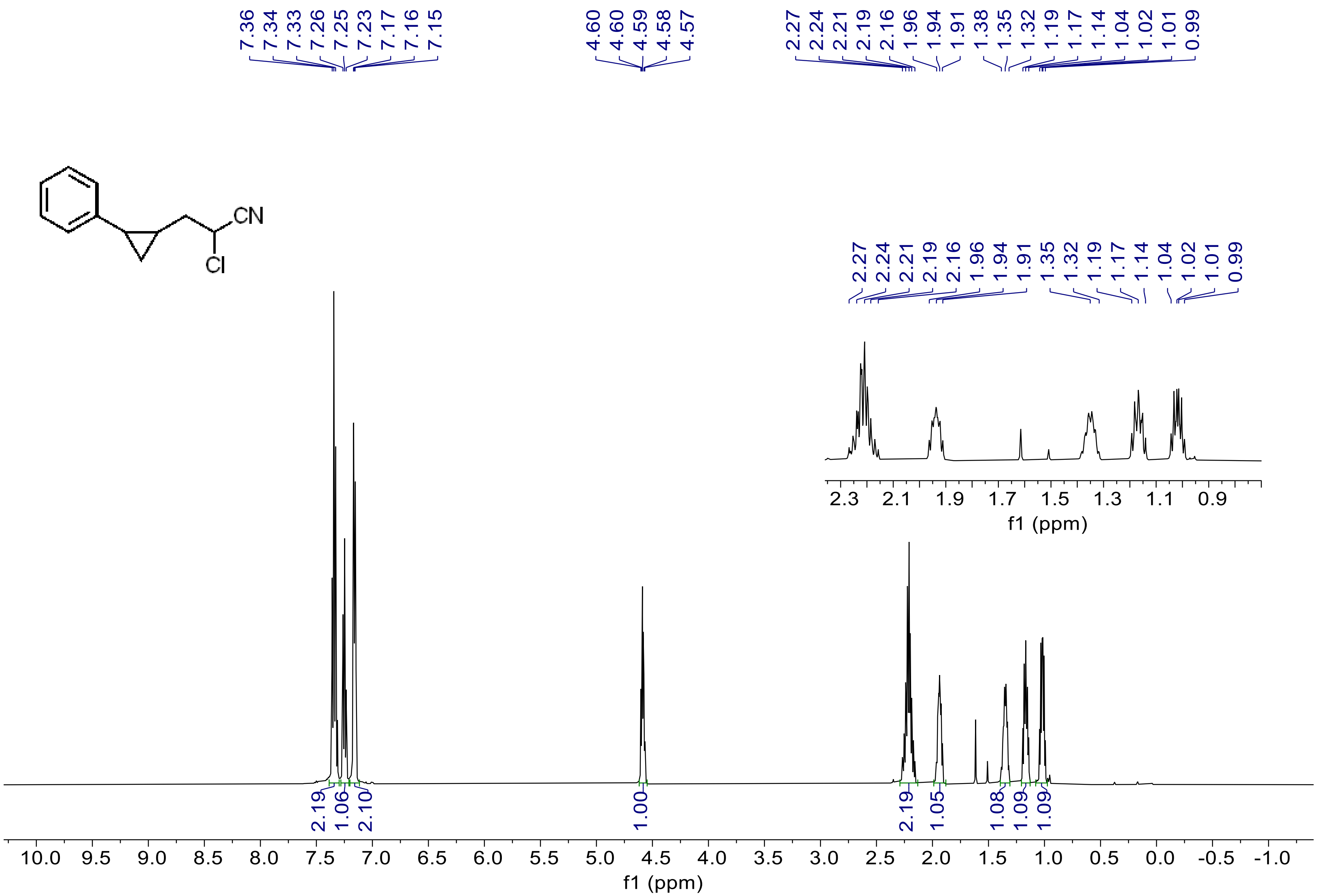

^{13}C NMR of *rac*-cyclopropane-derived α-chloronitrile 11

$CDCl_3$, 23 °C

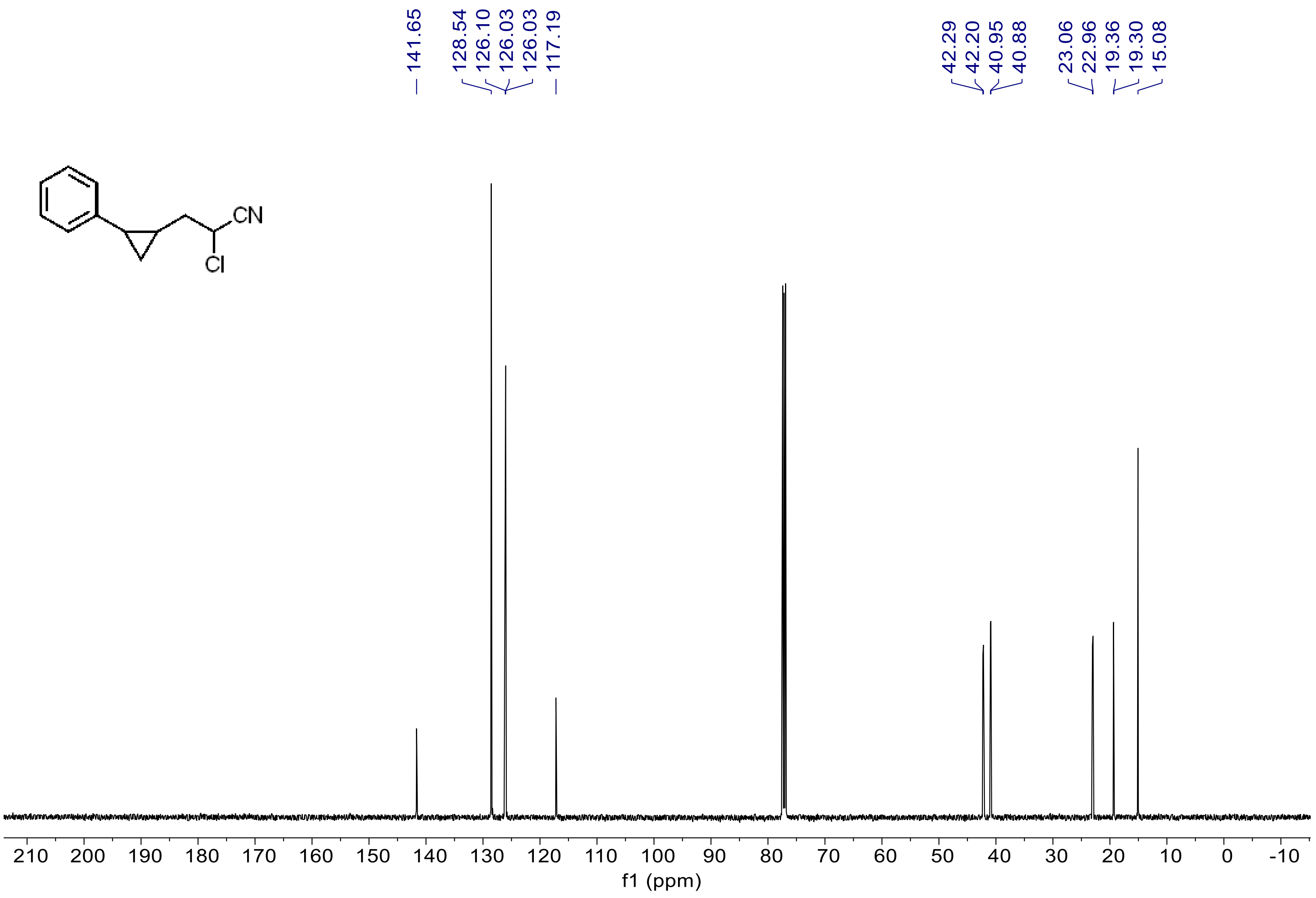

^{1}H NMR of *rac*-adamantane-derived α-chloronitrile 12

$CDCl_3$, 23 °C

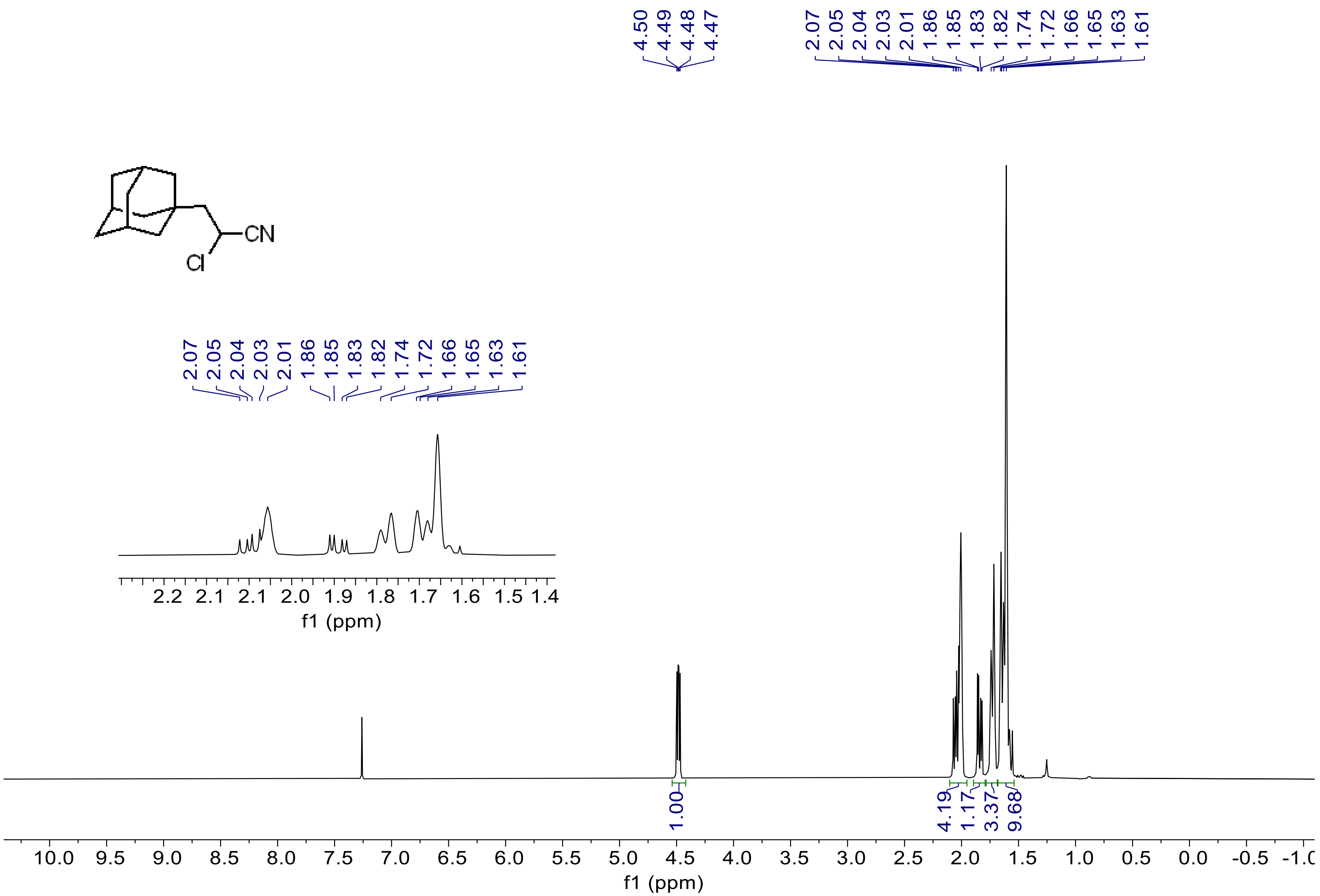

^{13}C NMR of *rac*-adamantane-derived α-chloronitrile 12

$CDCl_3$, 23 °C

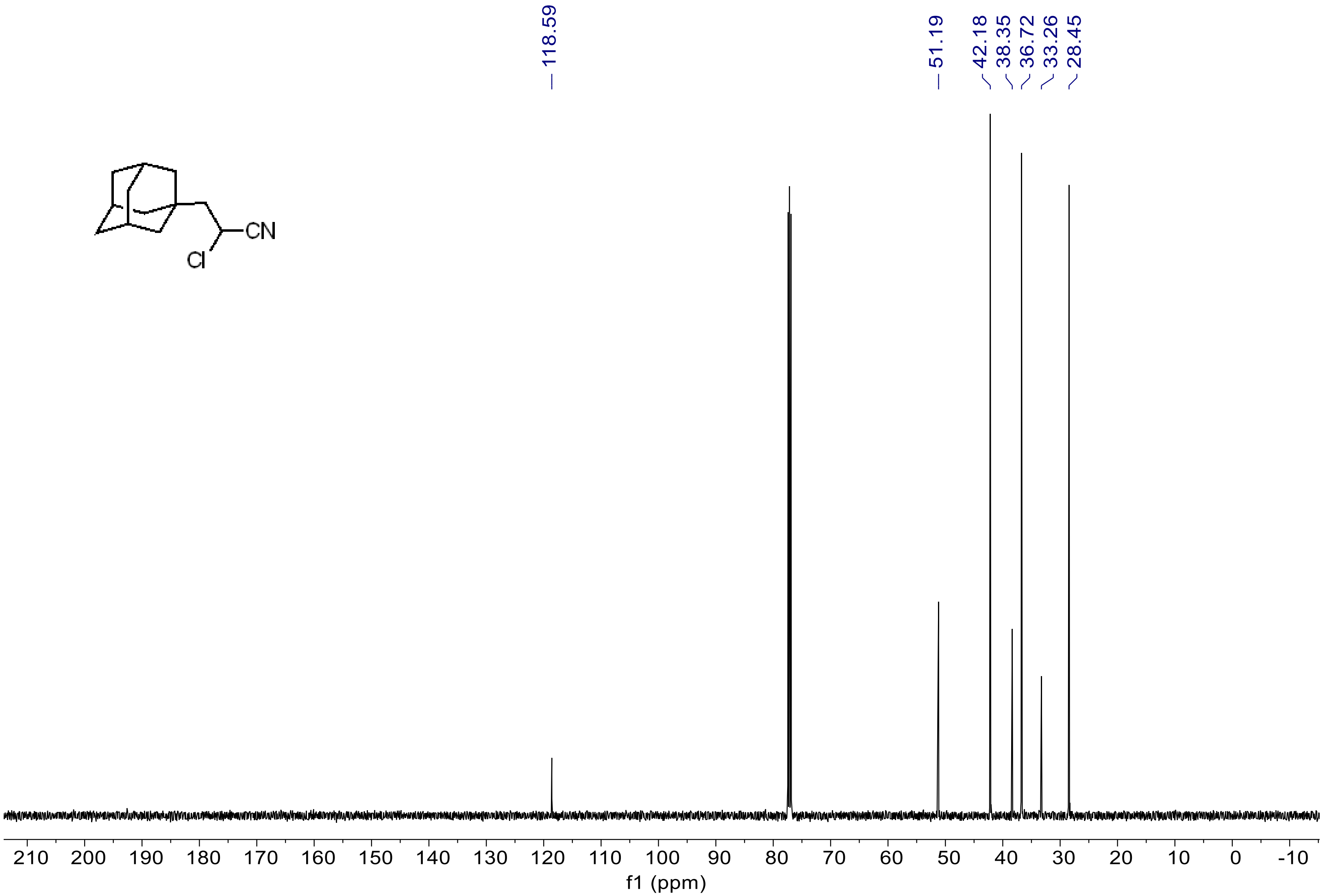

^{1}H NMR of *rac*-glutamic acid-derived α-chloronitrile 13

CD_3CN, 23 °C

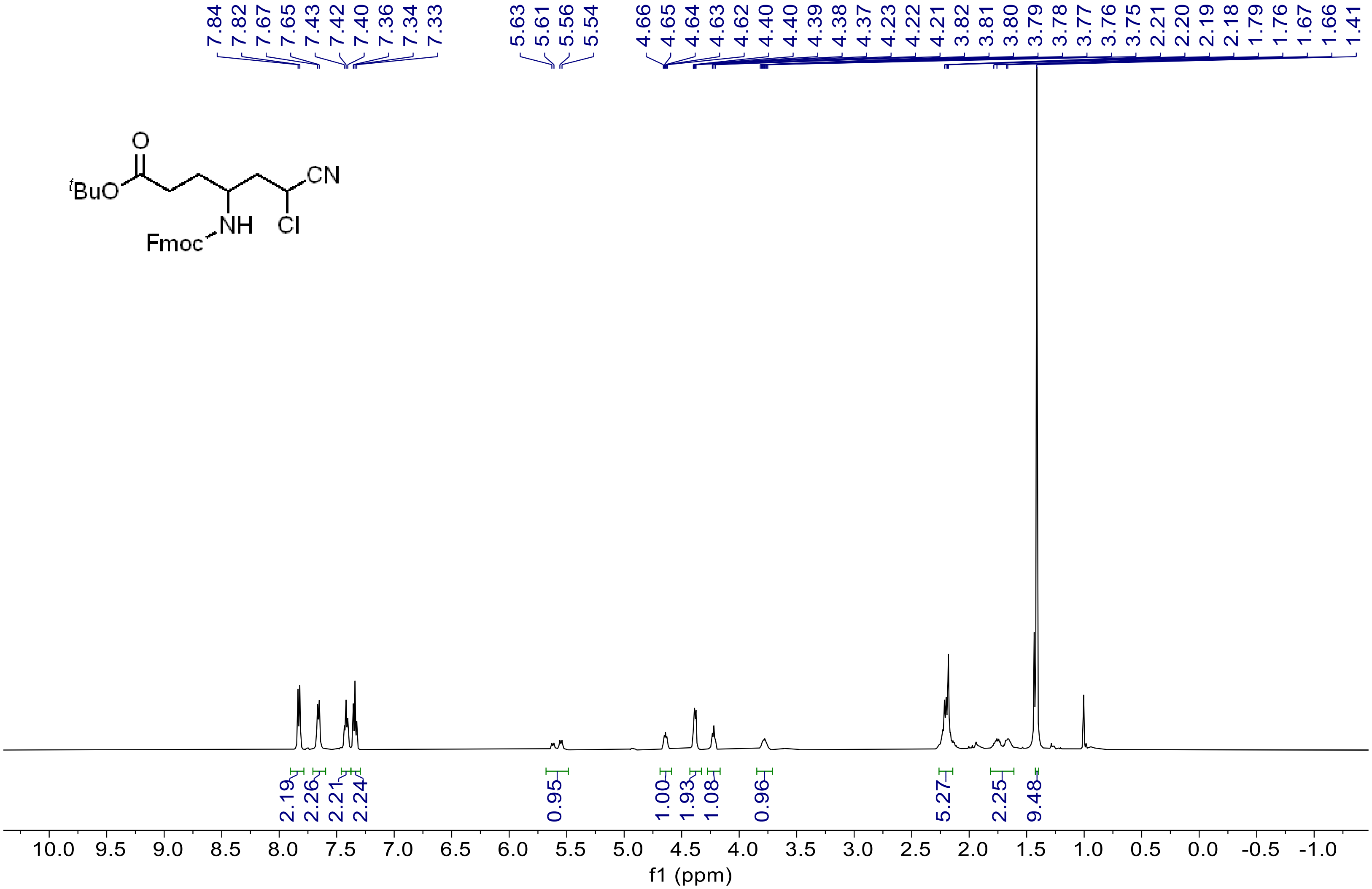

^{13}C NMR of *rac*-glutamic acid-derived α-chloronitrile 13

CD_3CN, 23 °C

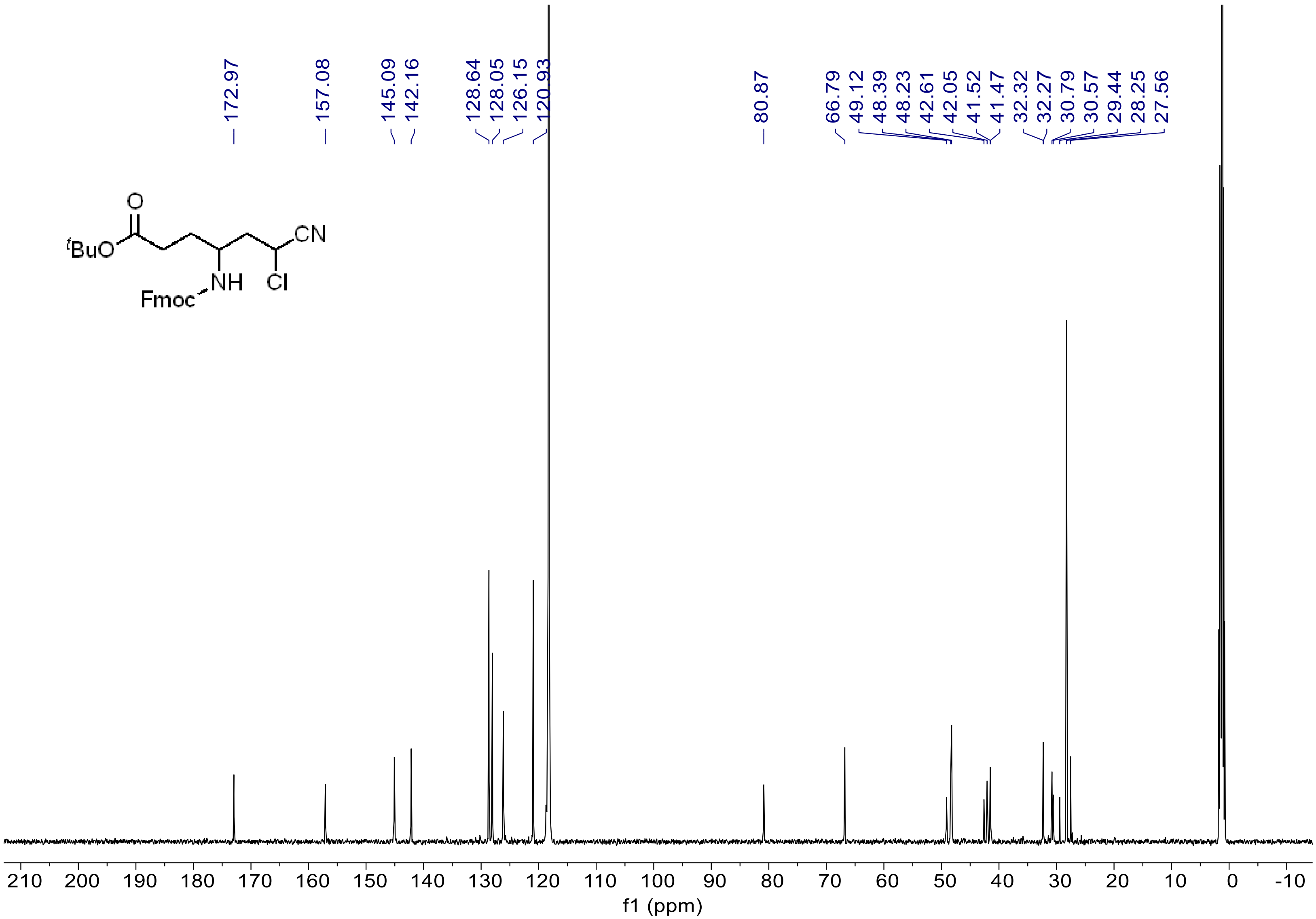

^{1}H NMR of lithocholic-derived α-chloronitrile 14

$CDCl_3$, 23 °C

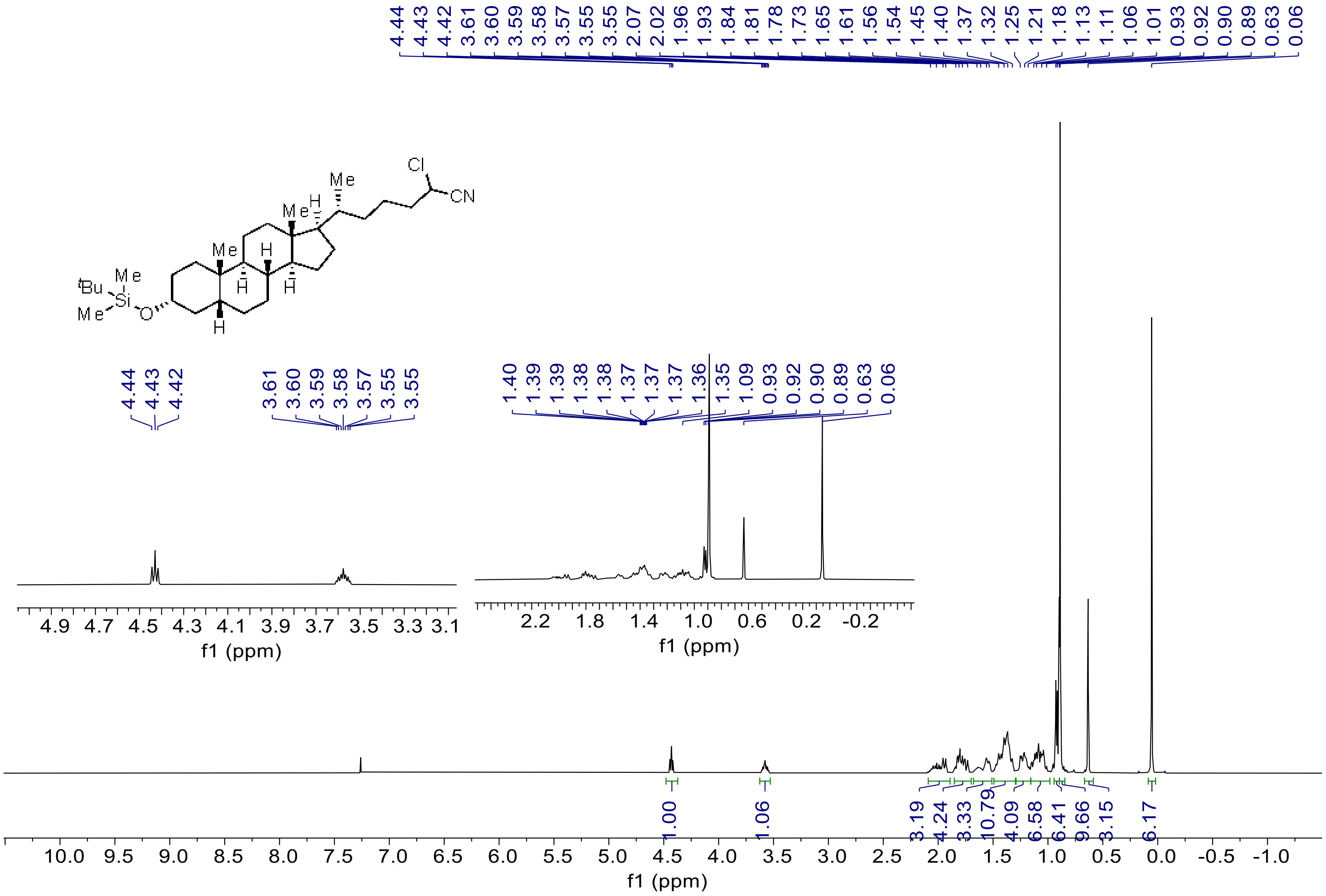

^{13}C NMR of lithocholic-derived α-chloronitrile 14

CDCl$_3$, 23 °C

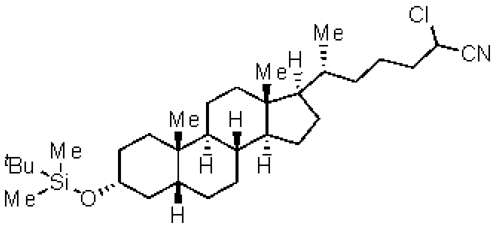

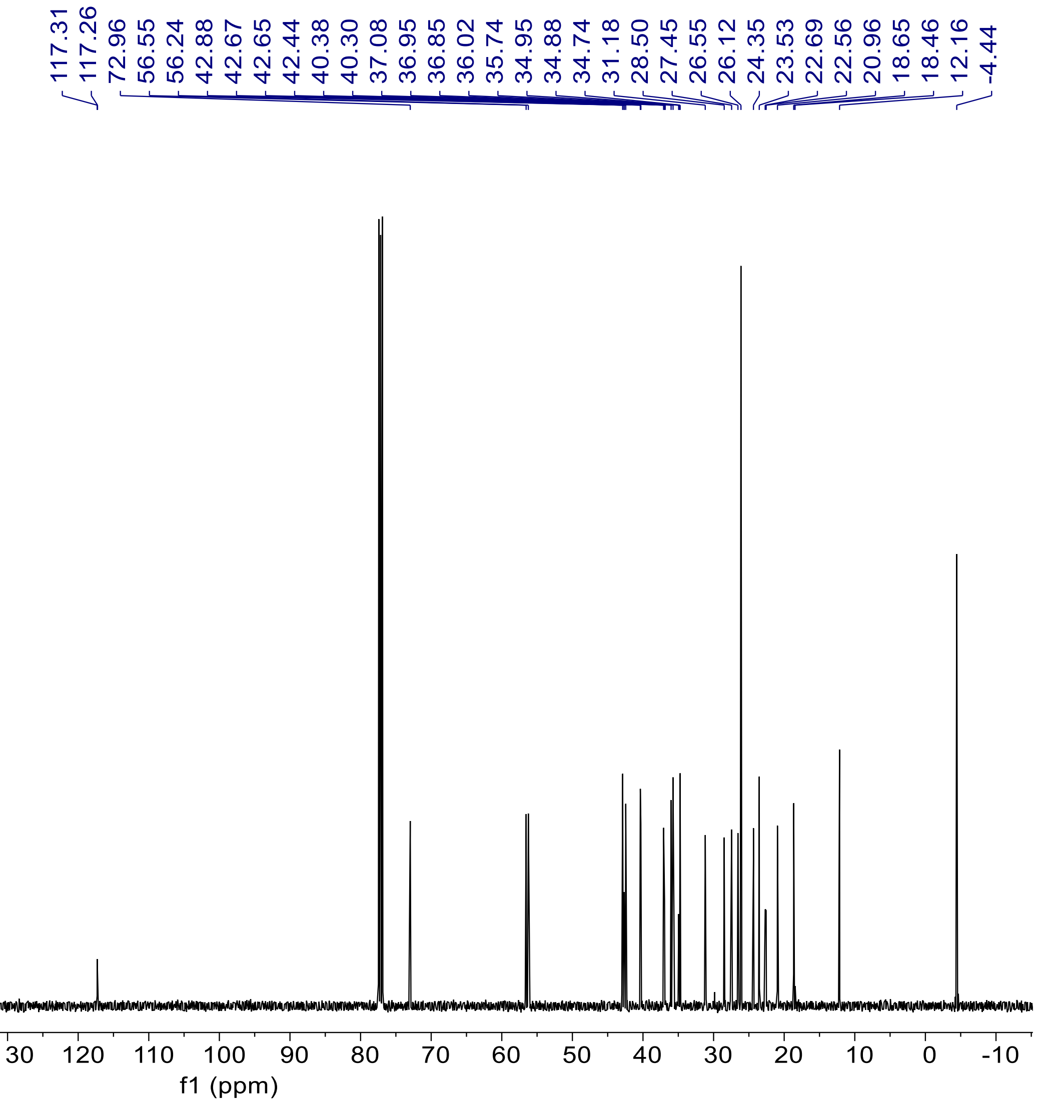

^{1}H NMR of *rac*-difluorocyclobutane-derived α-chloronitrile 15

$CDCl_3$, 23 °C

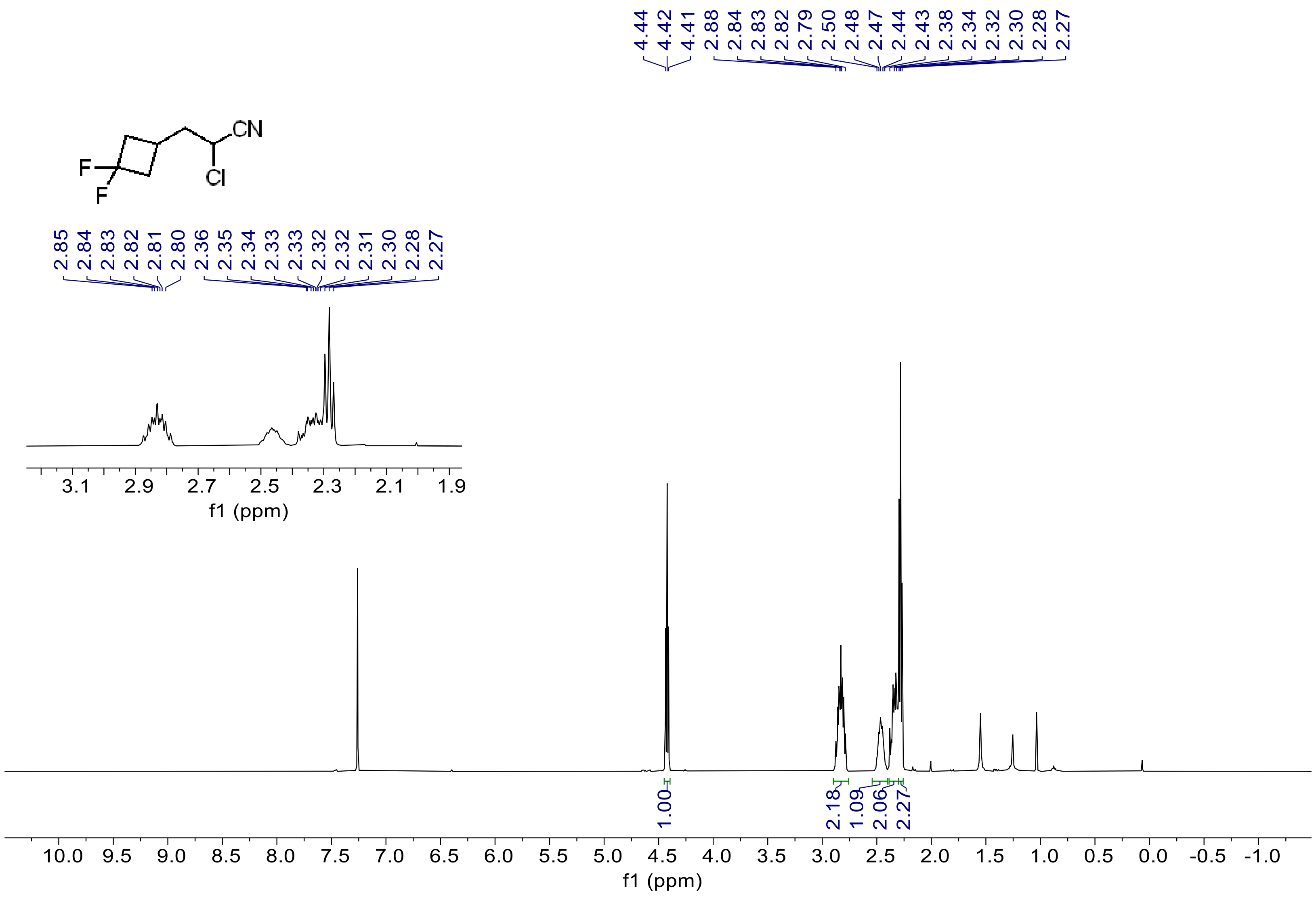

^{19}F NMR of *rac*-difluorocyclobutane-derived α-chloronitrile 15

CDCl$_3$, 23 °C

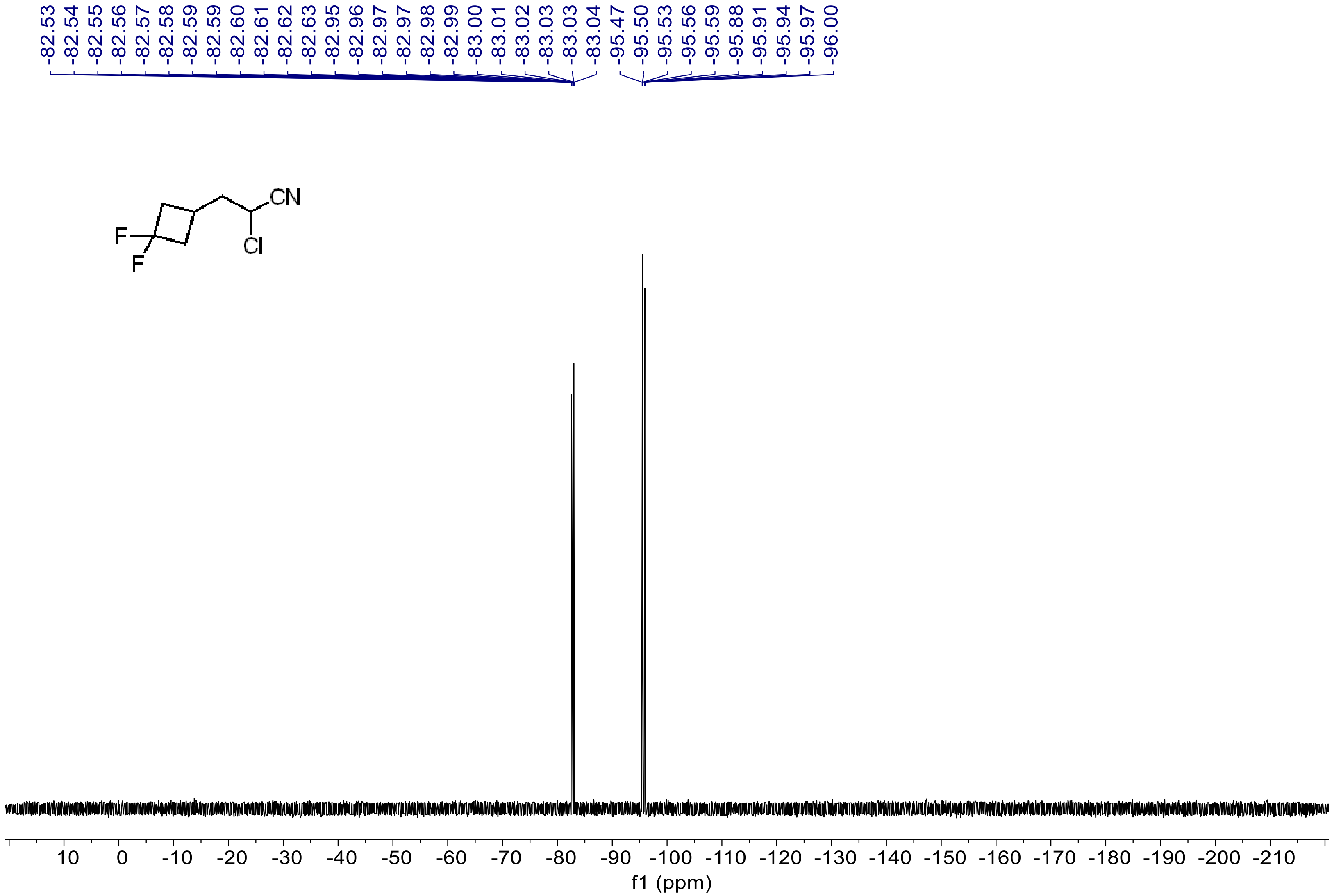

^{13}C NMR of *rac*-difluorocyclobutane-derived α-chloronitrile 15

$CDCl_3$, 23 °C

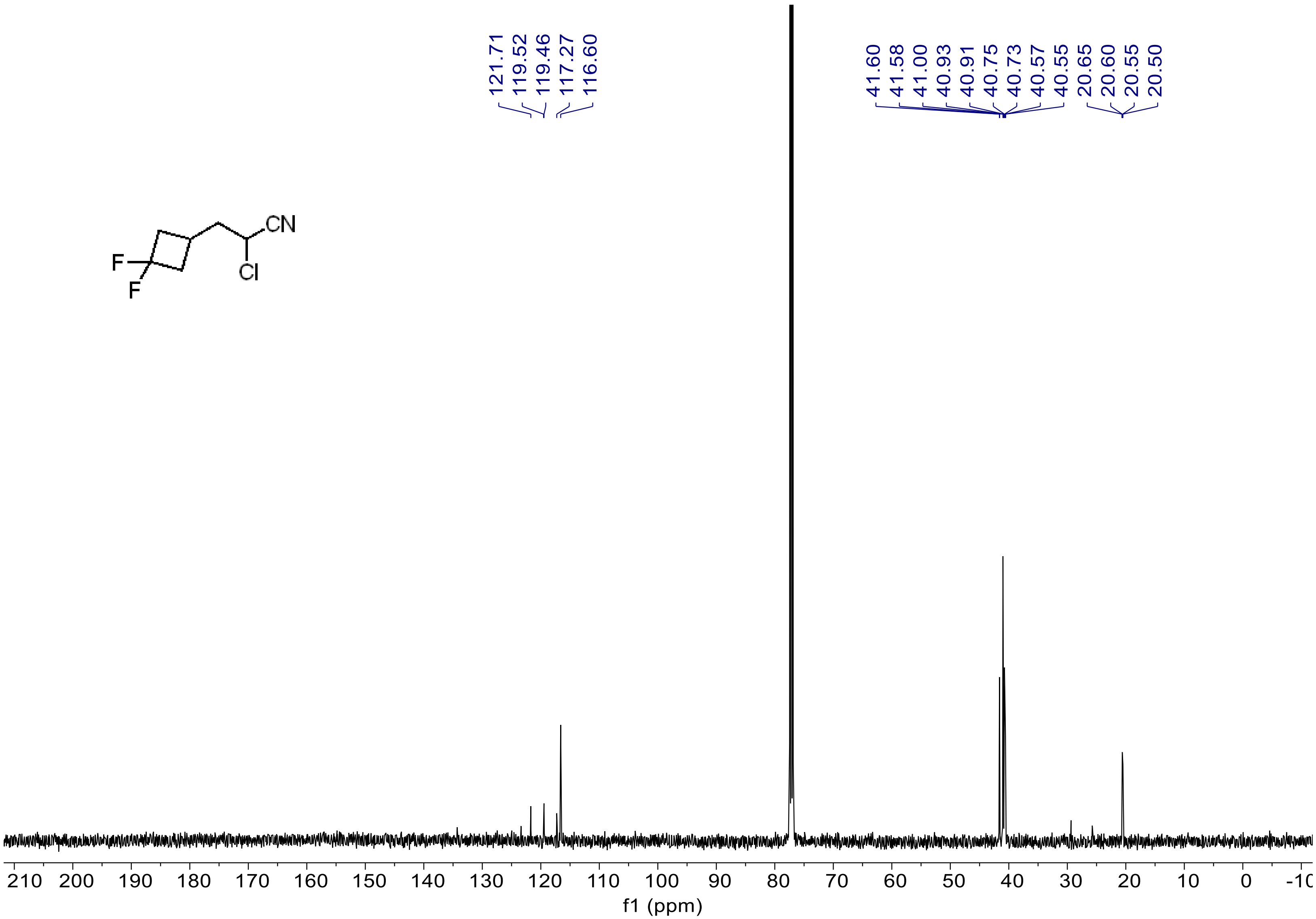

^{1}H NMR of *rac*-dihydrobenzodioxine-derived α-chloronitrile 16

$CDCl_3$, 23 °C

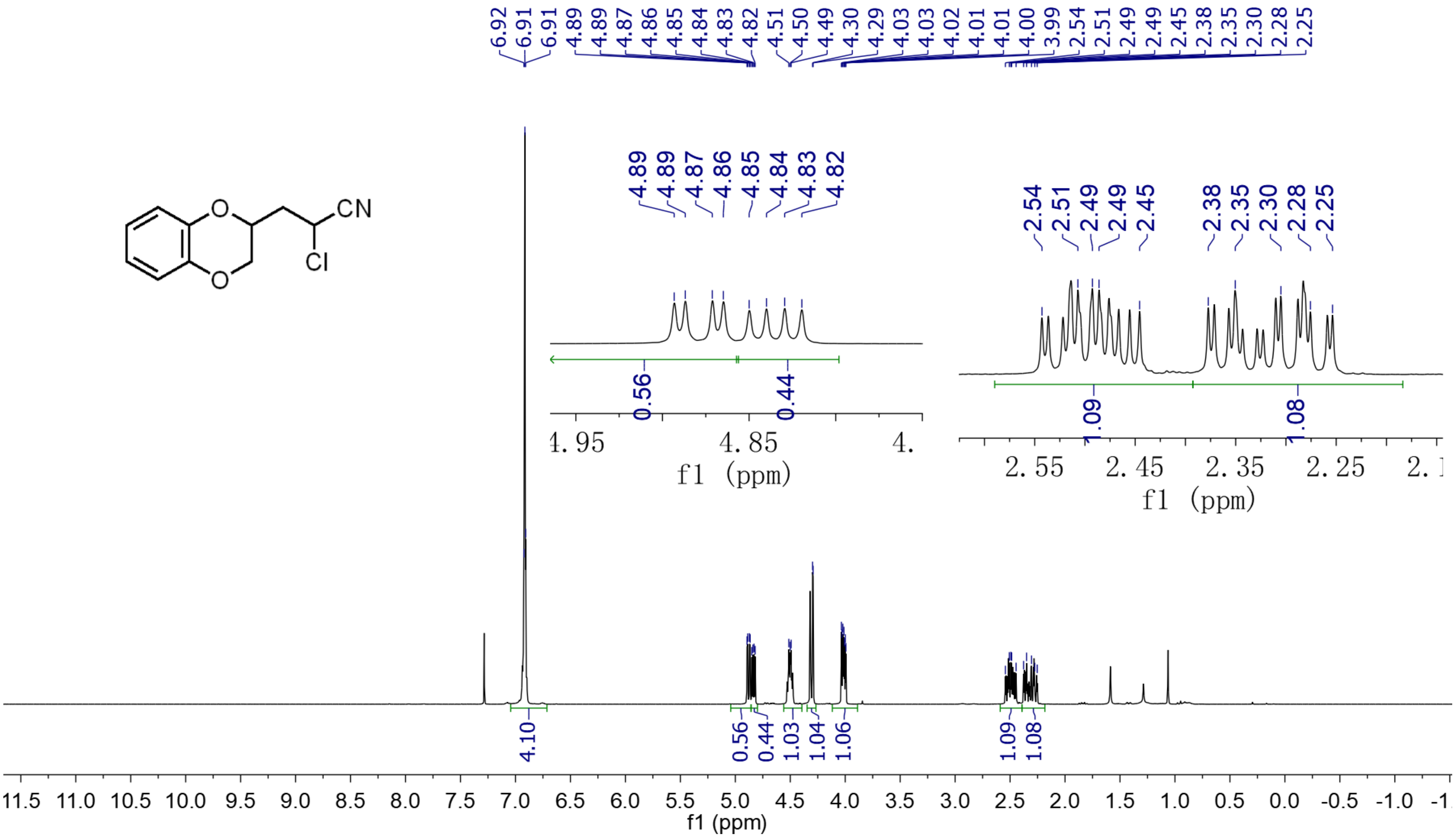

^{13}C NMR of *rac*-dihydrobenzodioxine-derived α-chloronitrile 16

$CDCl_3$, 23 °C

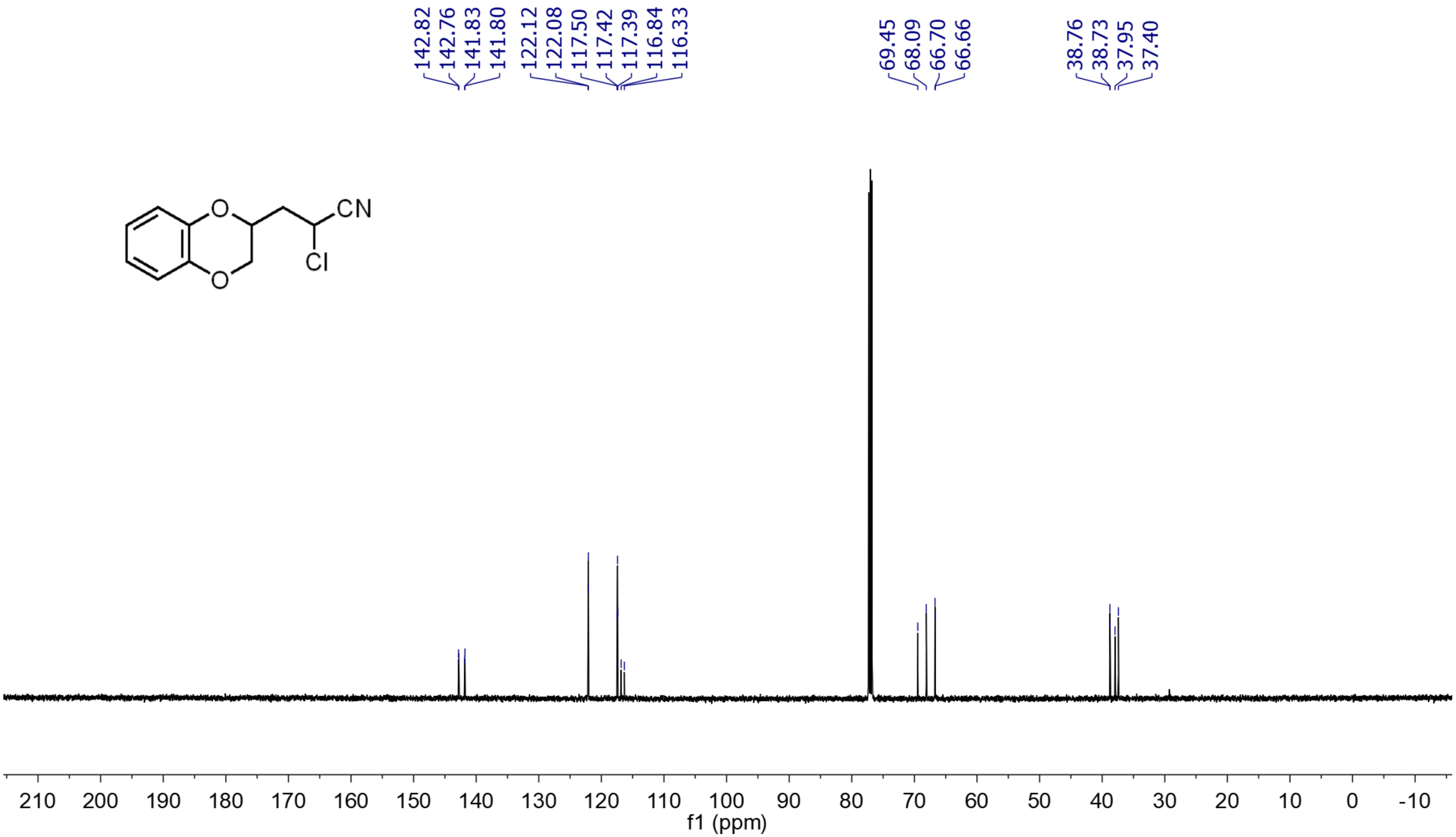

^{1}H NMR of dehydroabietic-derived α-chloronitrile 17

$CDCl_3$, 23 °C

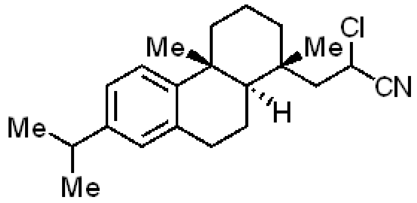

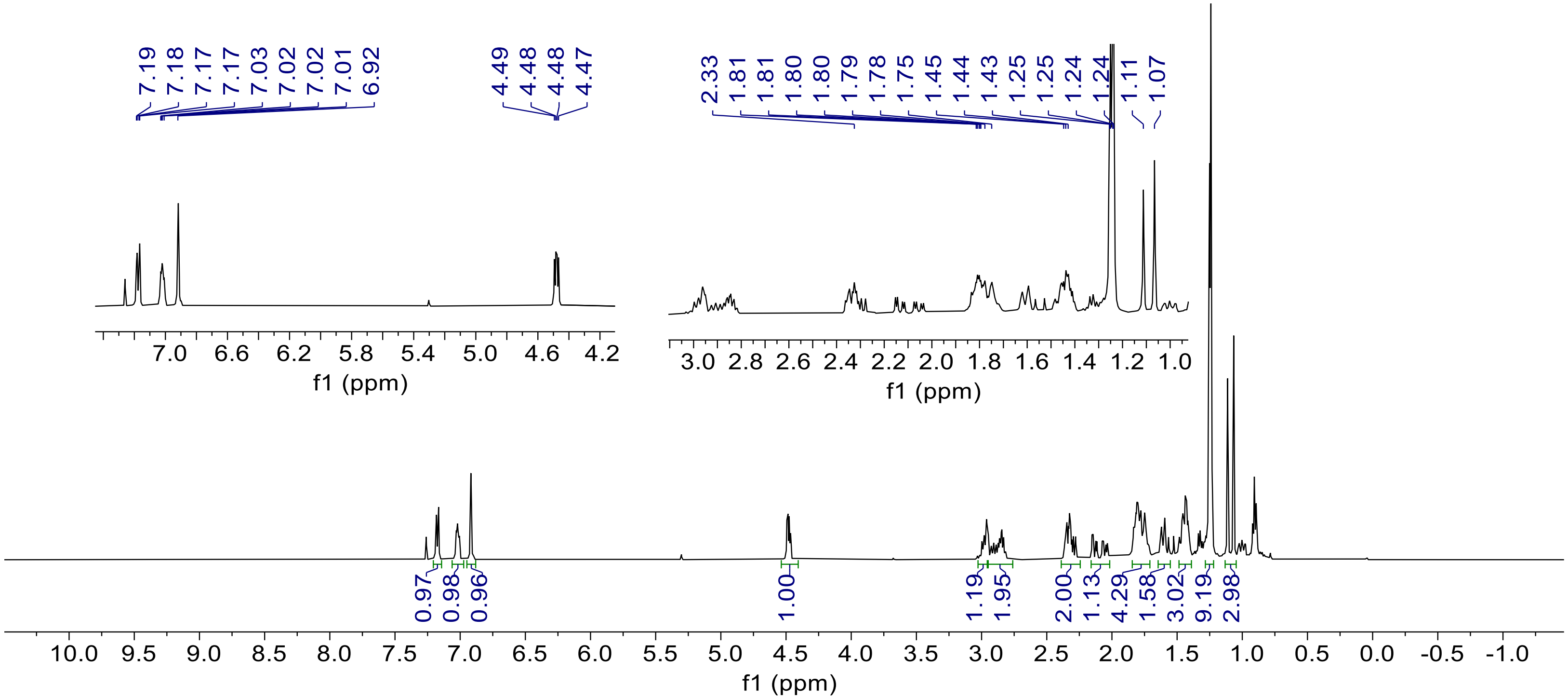

^{13}C NMR of dehydroabietic-derived α-chloronitrile 17

$CDCl_3$, 23 °C

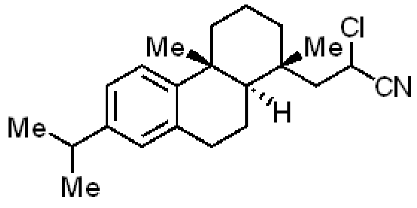

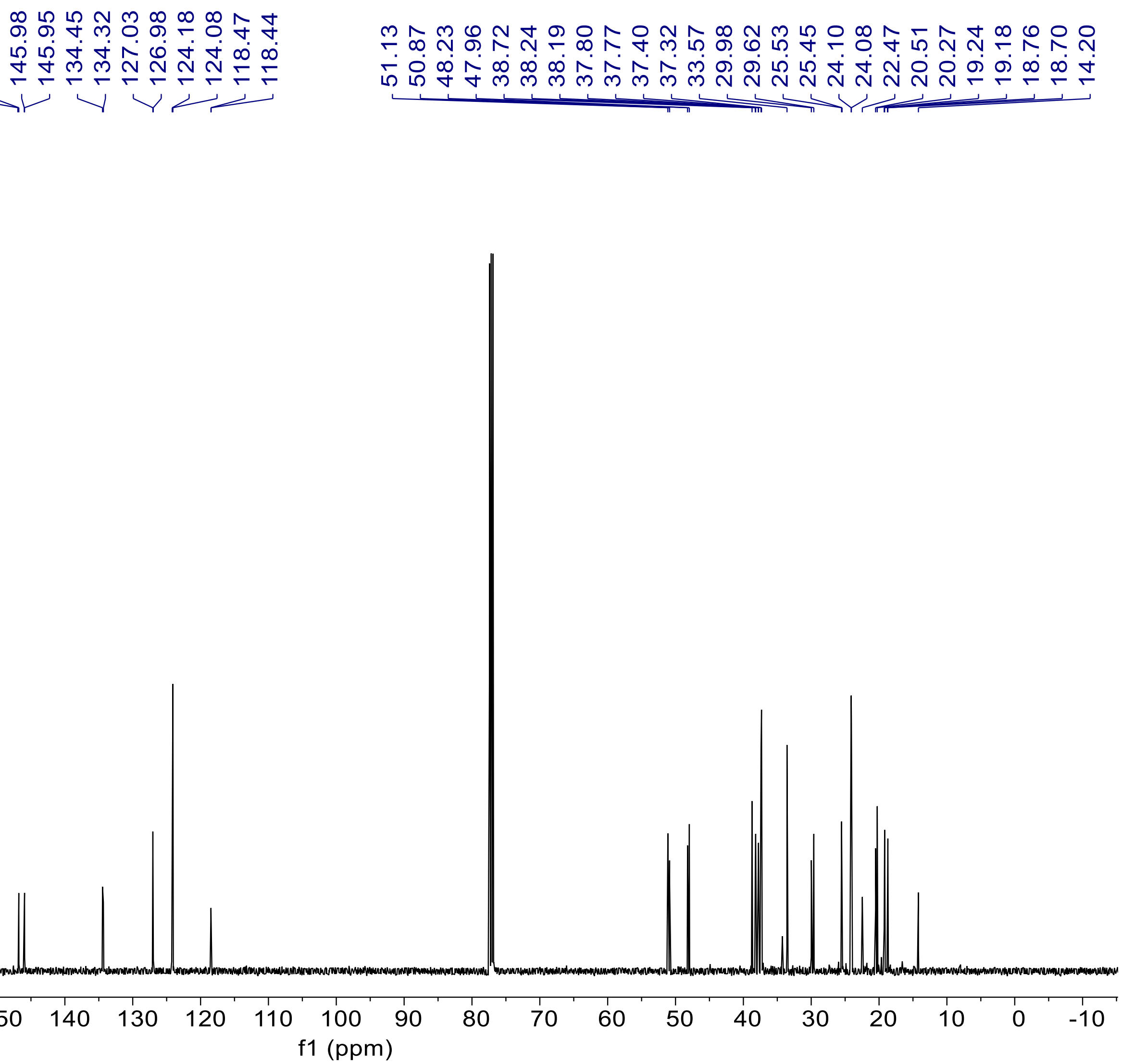

^{1}H NMR of *rac*-bezafibrate-derived α-chloronitrile 18

$CDCl_3$, 23 °C

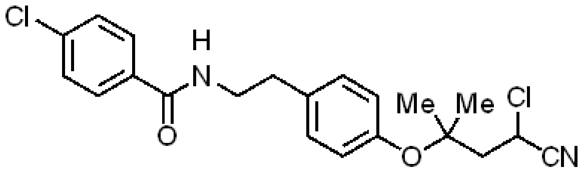

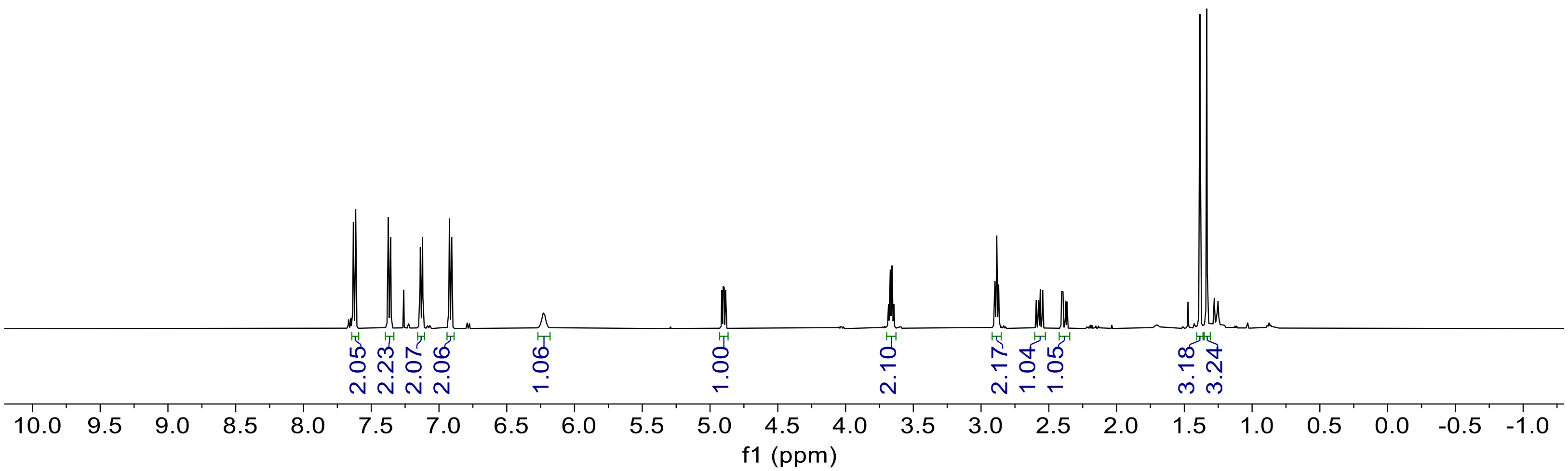

^{13}C NMR of *rac*-bezafibrate-derived α-chloronitrile 18

$CDCl_3$, 23 °C

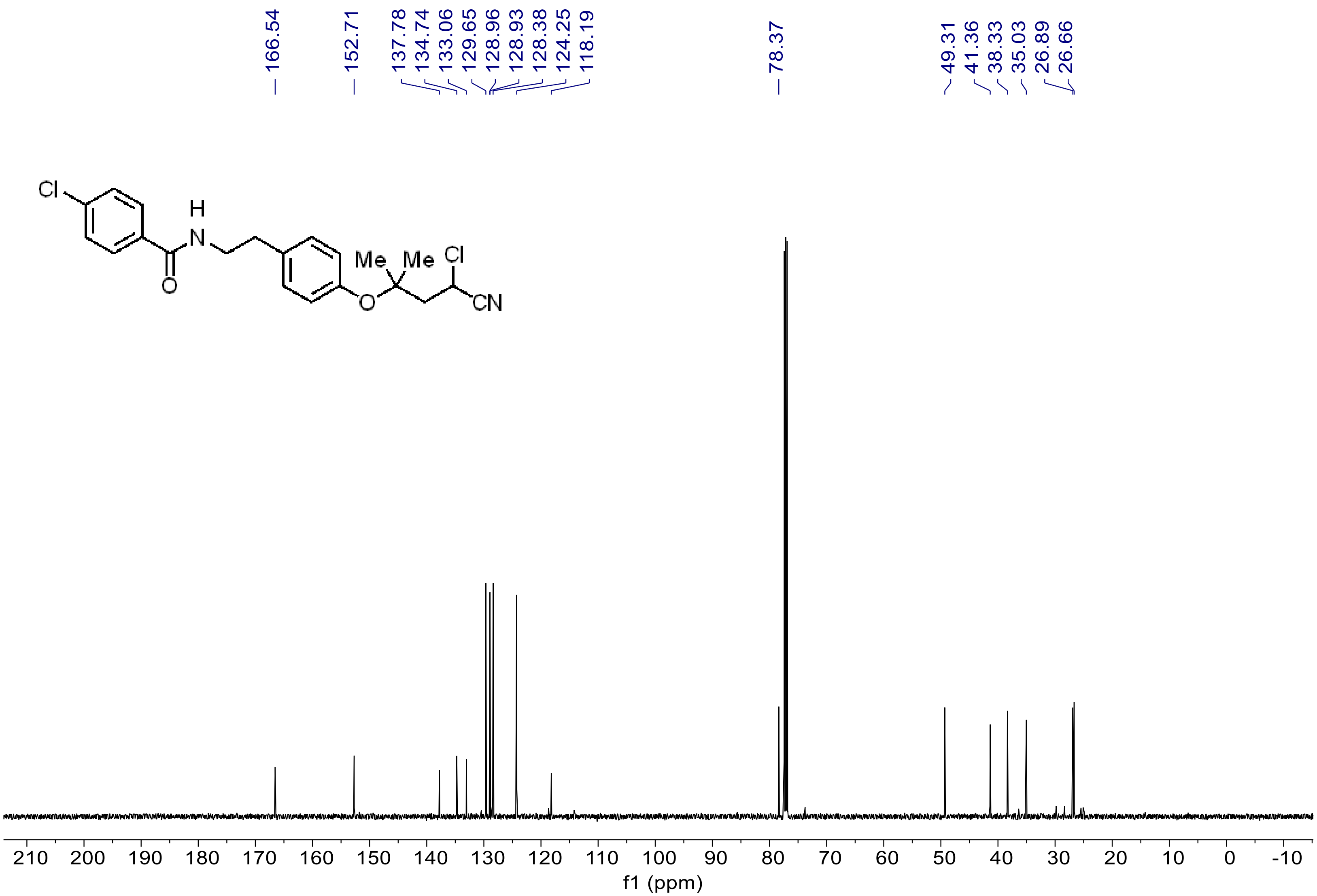

^{1}H NMR of (methyl-d$_3$)-derived α-chloroisopropyloxazolidone 19

$CDCl_3$, 23 °C

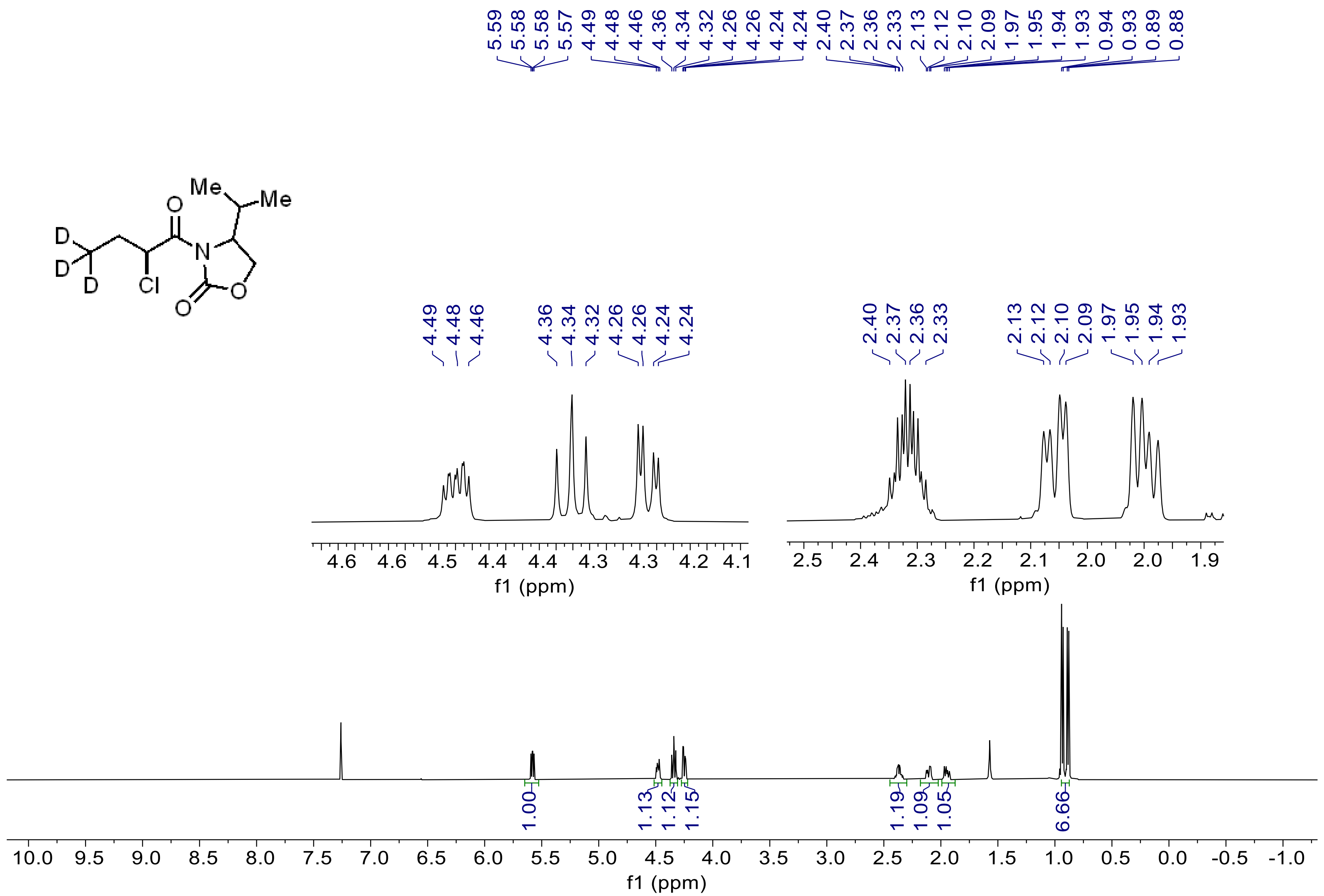

^{13}C NMR of (methyl-d_3)-derived α-chloroisopropyloxazolidone 19

$CDCl_3$, 23 °C

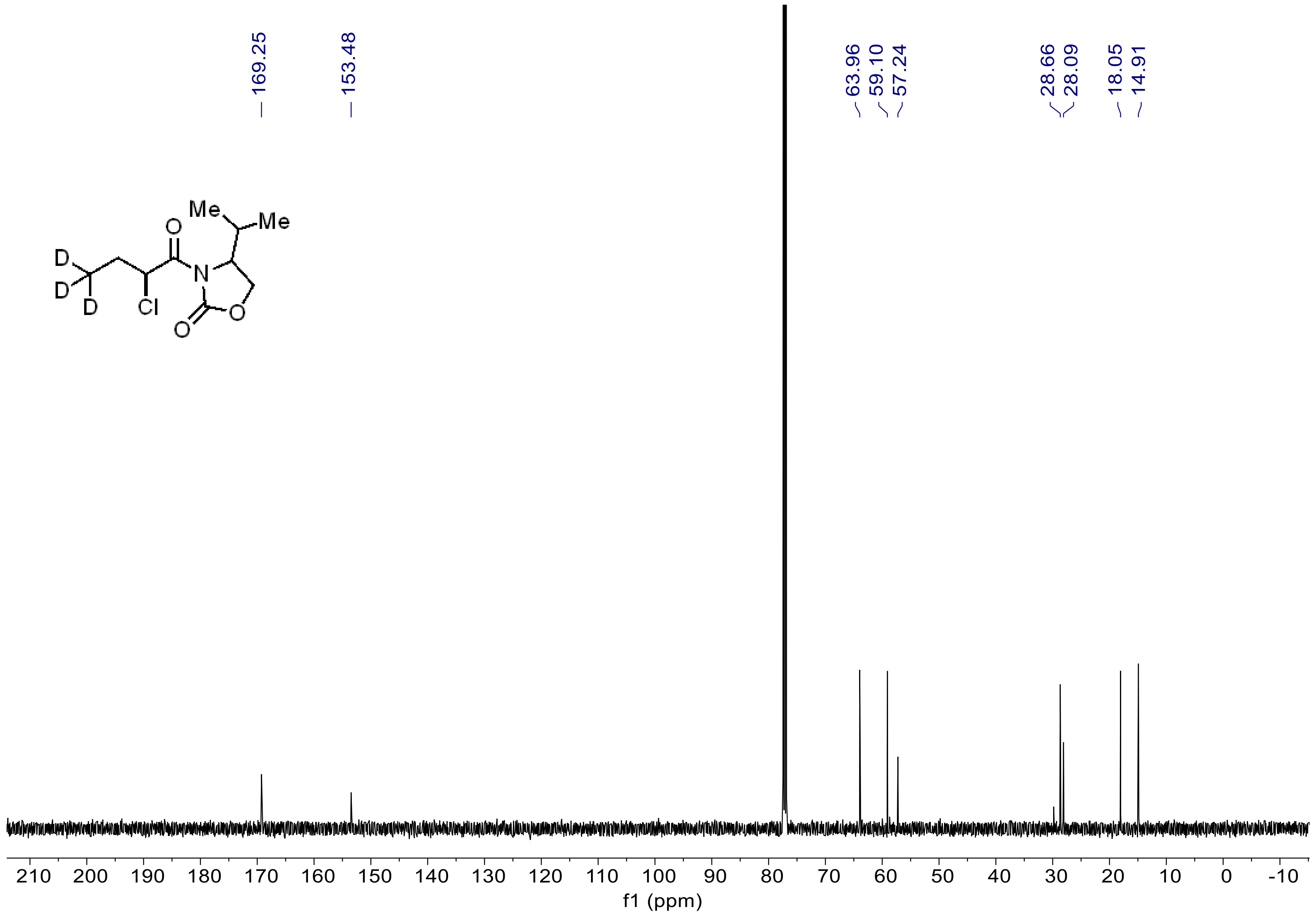

^{1}H NMR of *rac*-oxoacridin-derived α-chloronitrile 20

$CDCl_3$, 23 °C

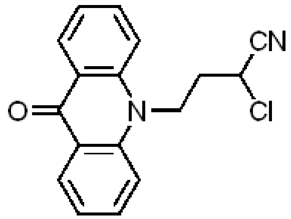

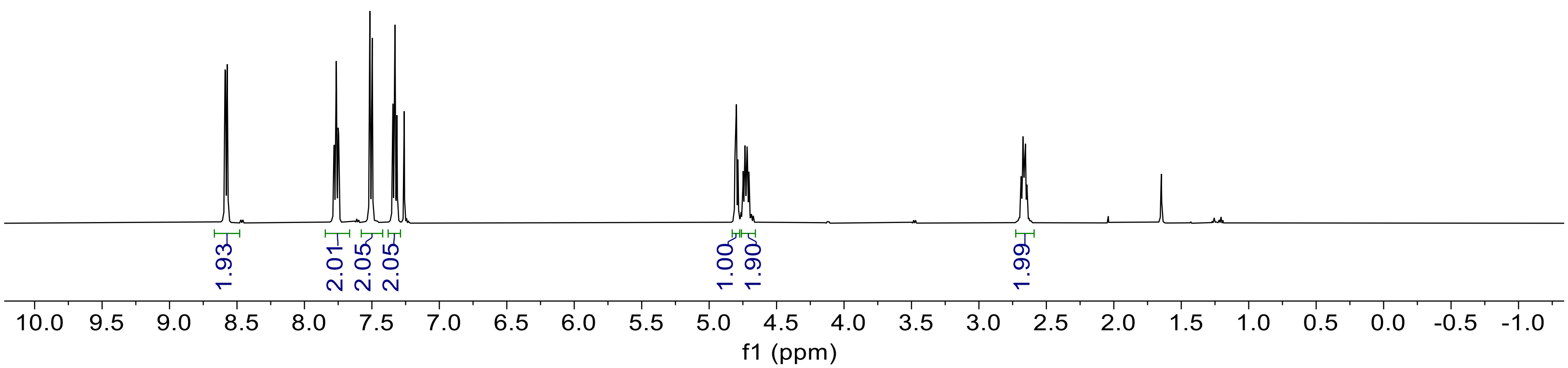

^{13}C NMR of *rac*-oxoacridin-derived α-chloronitrile 20

$CDCl_3$, 23 °C

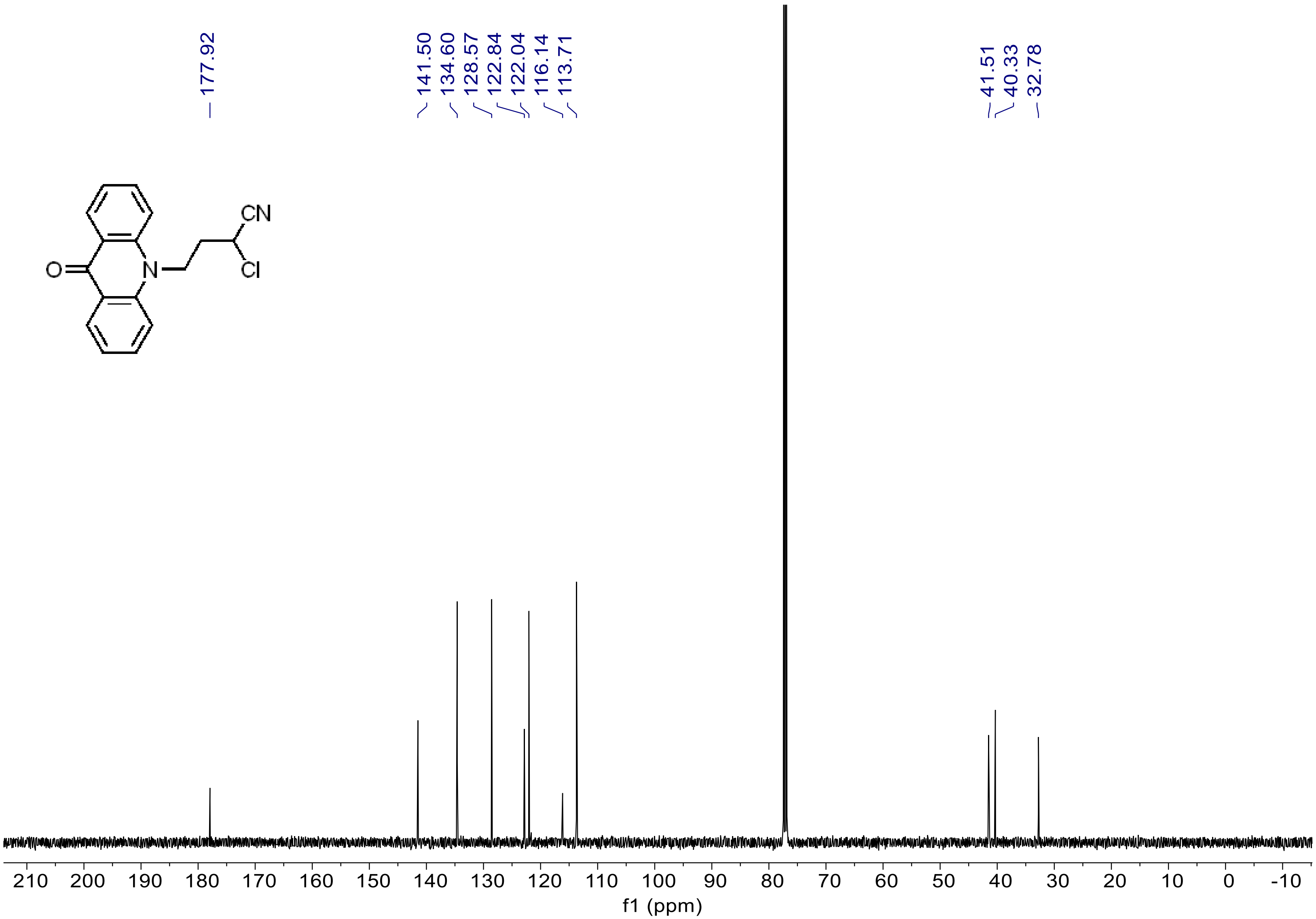

^{1}H NMR of *rac*-tryptophan-derived α-chloronitrile 21

DMSO, 23 °C

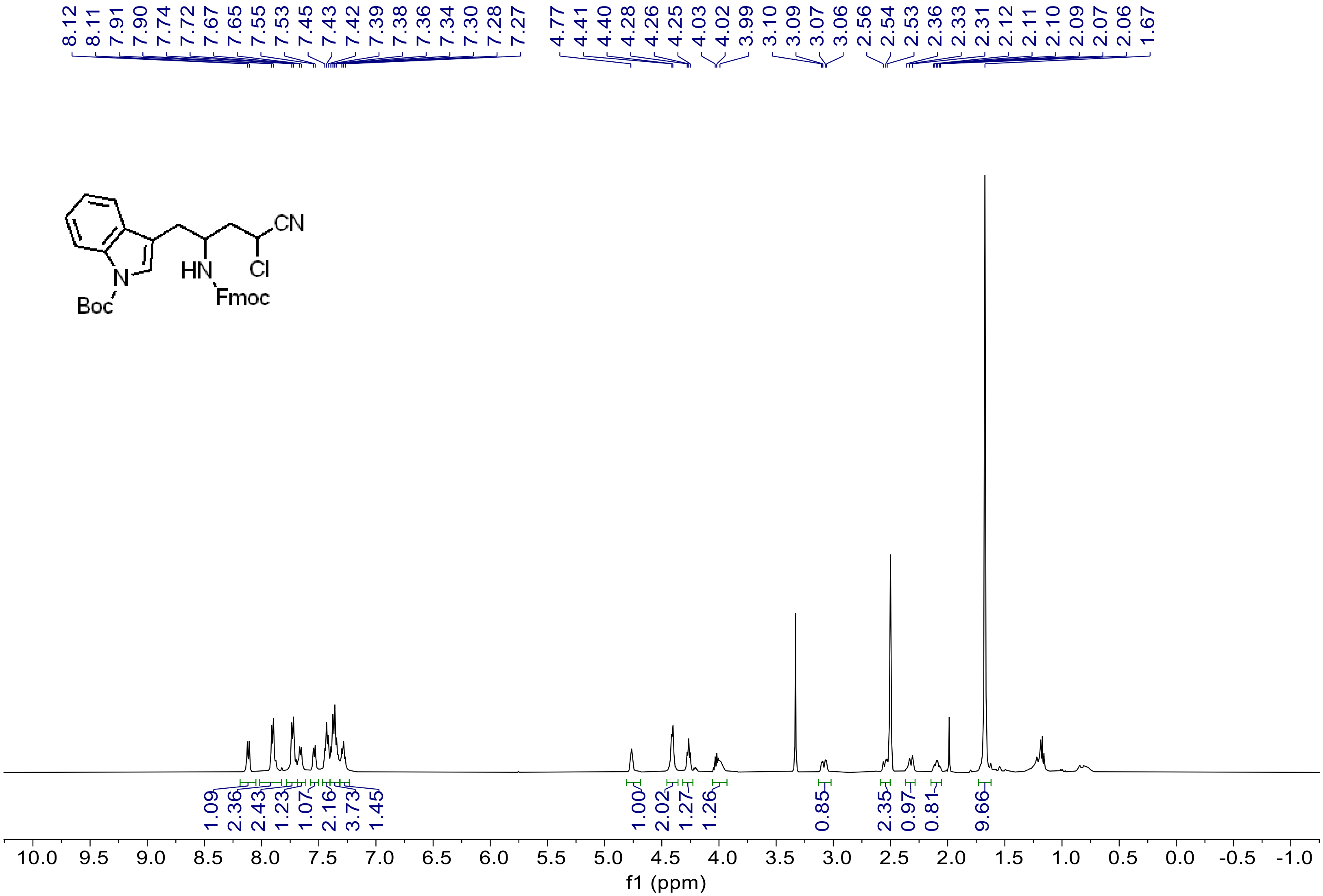

^{13}C NMR of *rac*-tryptophan-derived α-chloronitrile 21

DMSO, 23 °C

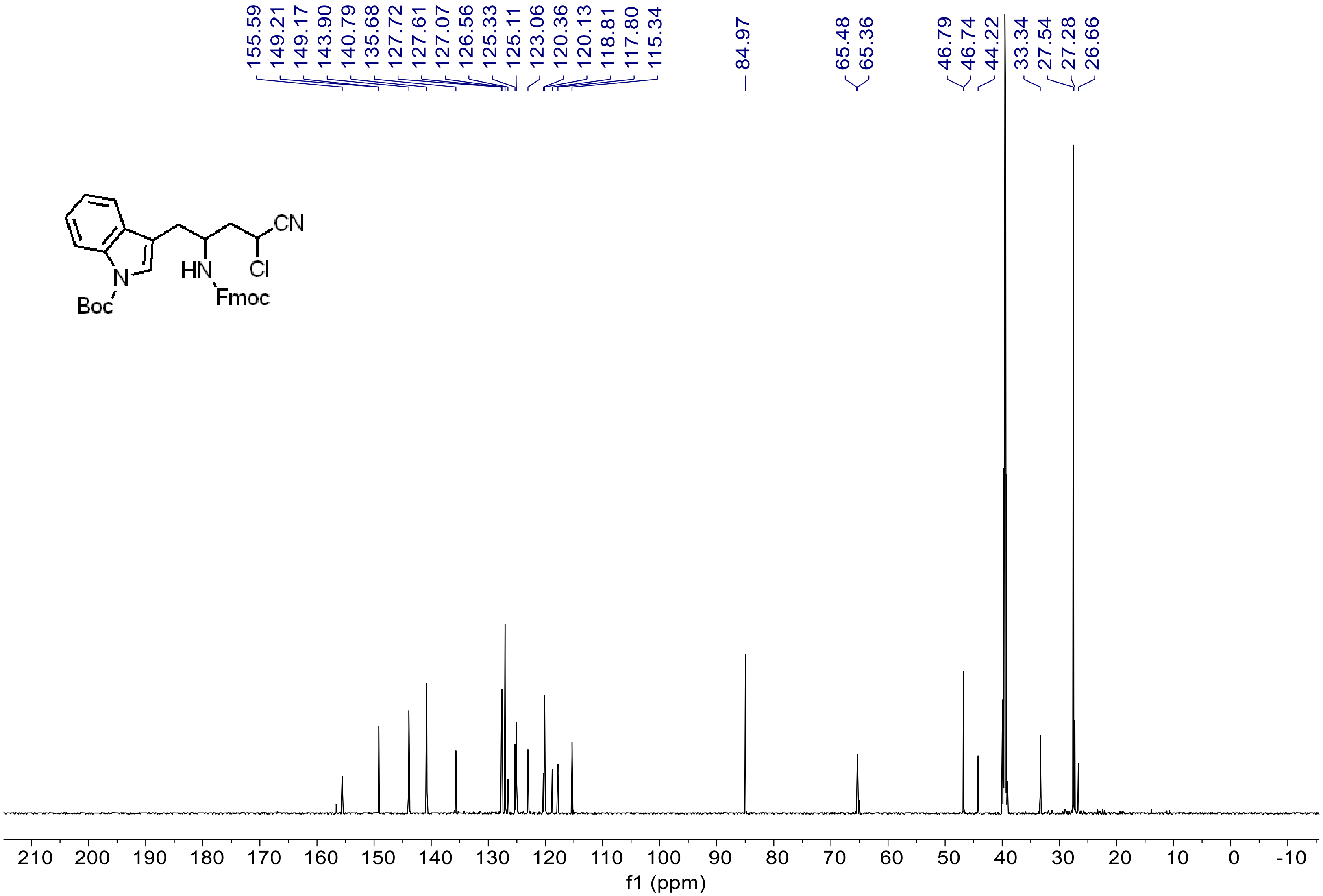

^{1}H NMR of *rac*-dipeptide-derived α-chloronitrile 22

DMSO, 23 °C

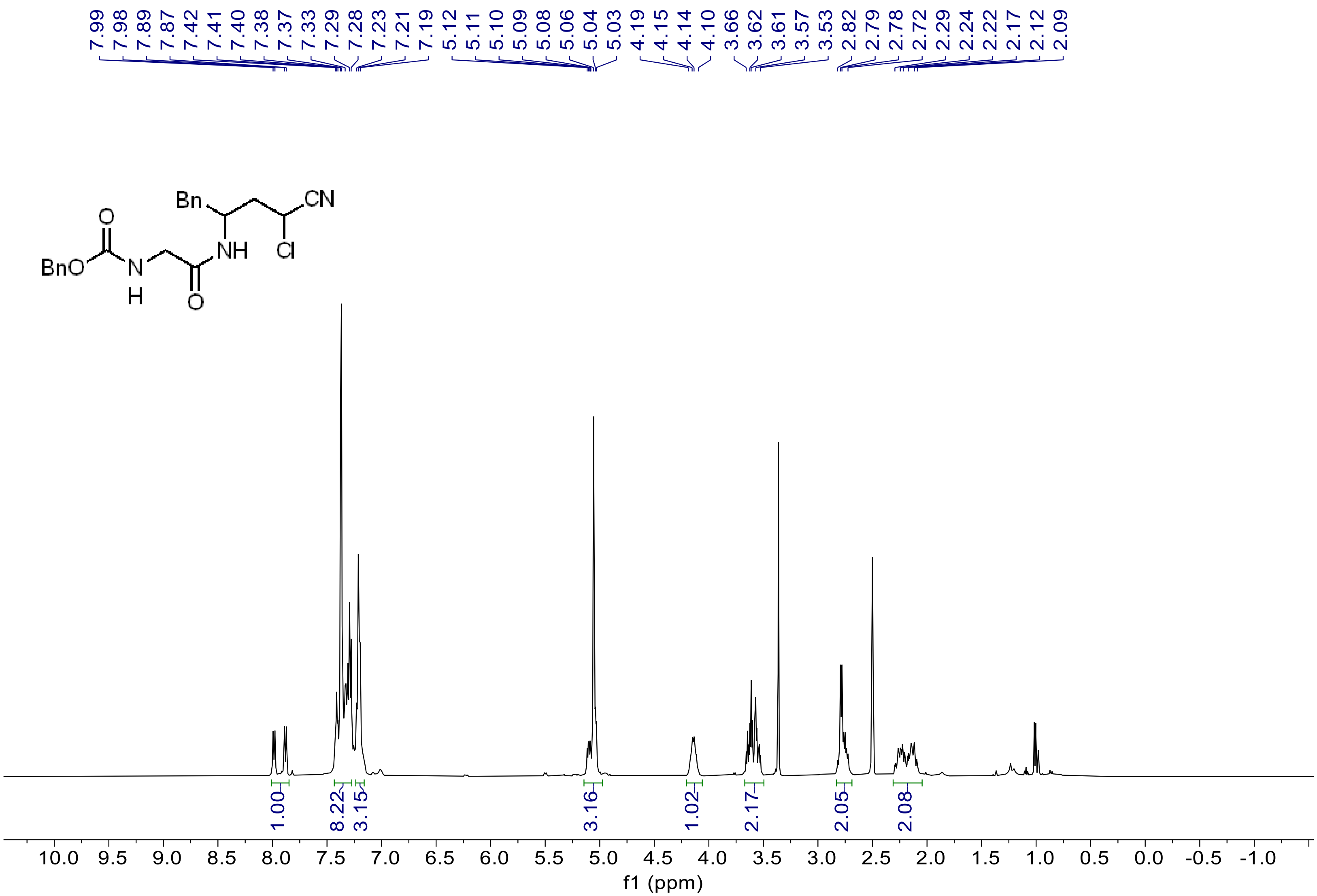

^{13}C NMR of *rac*-dipeptide-derived α-chloronitrile 22

DMSO, 23 °C

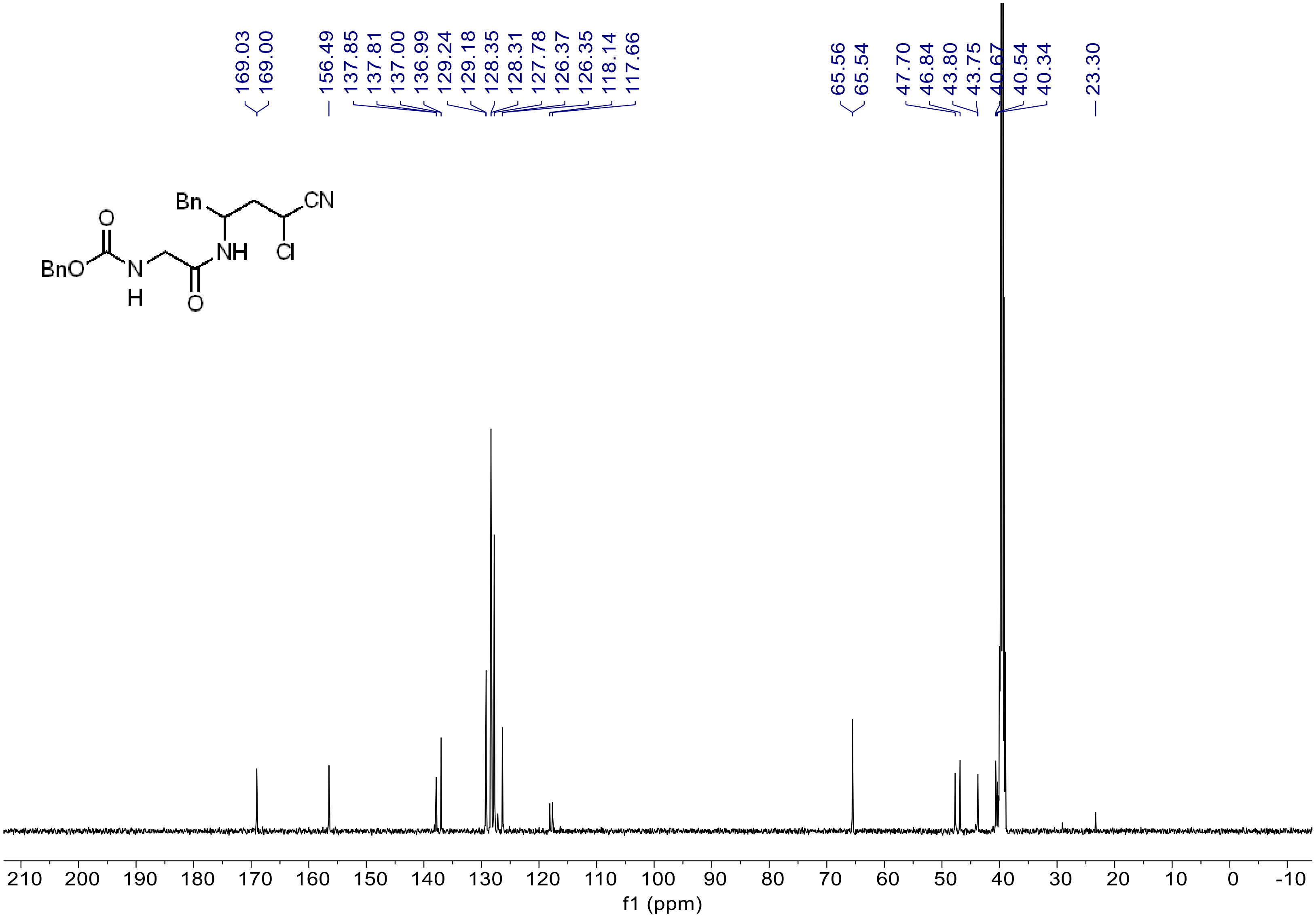

^{1}H NMR of *rac*-linolenic-derived α-chloronitrile 23

$CDCl_3$, 23 °C

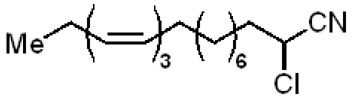

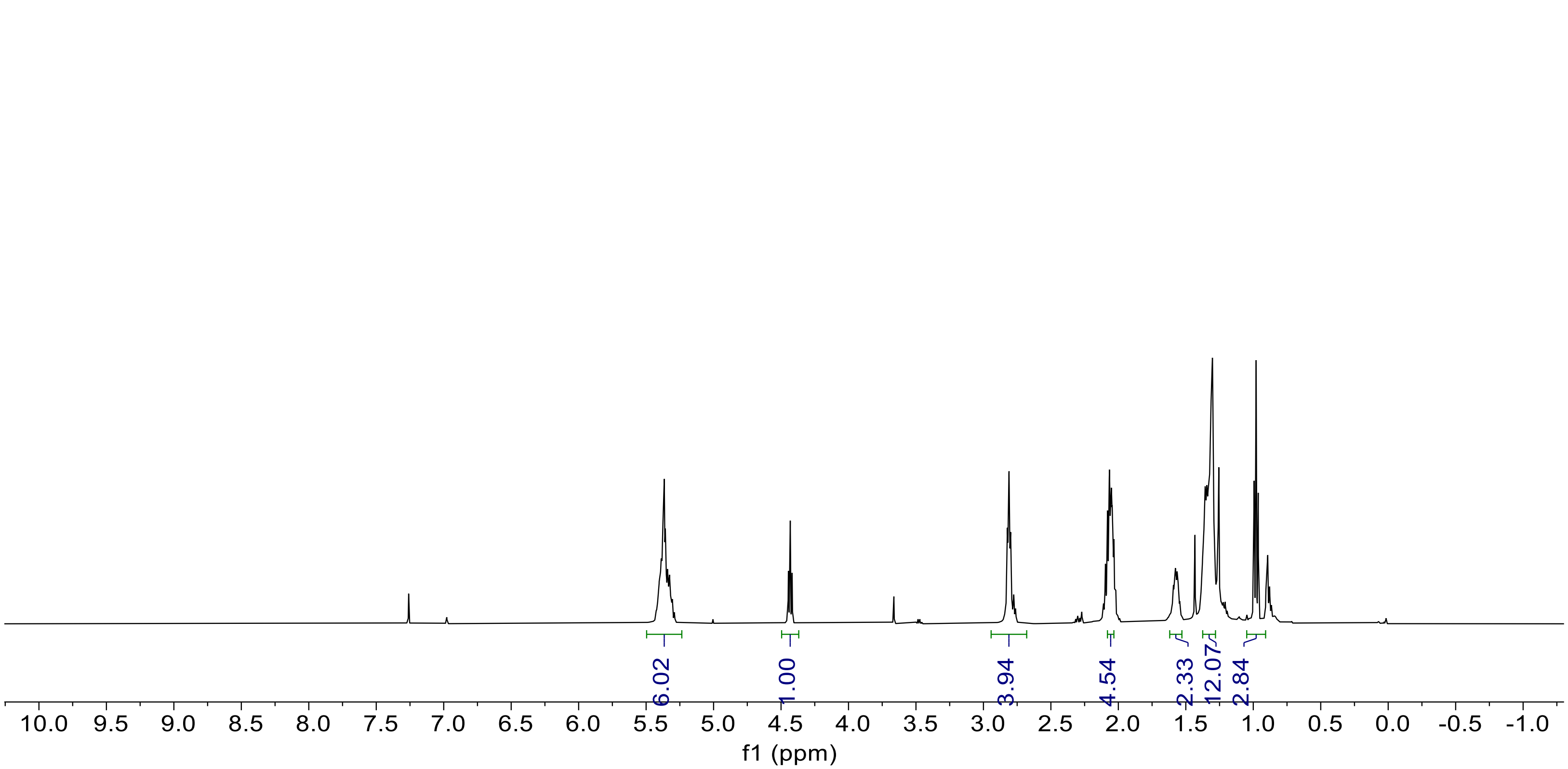

^{13}C NMR of *rac*-linolenic-derived α-chloronitrile 23

CDCl$_3$, 23 °C

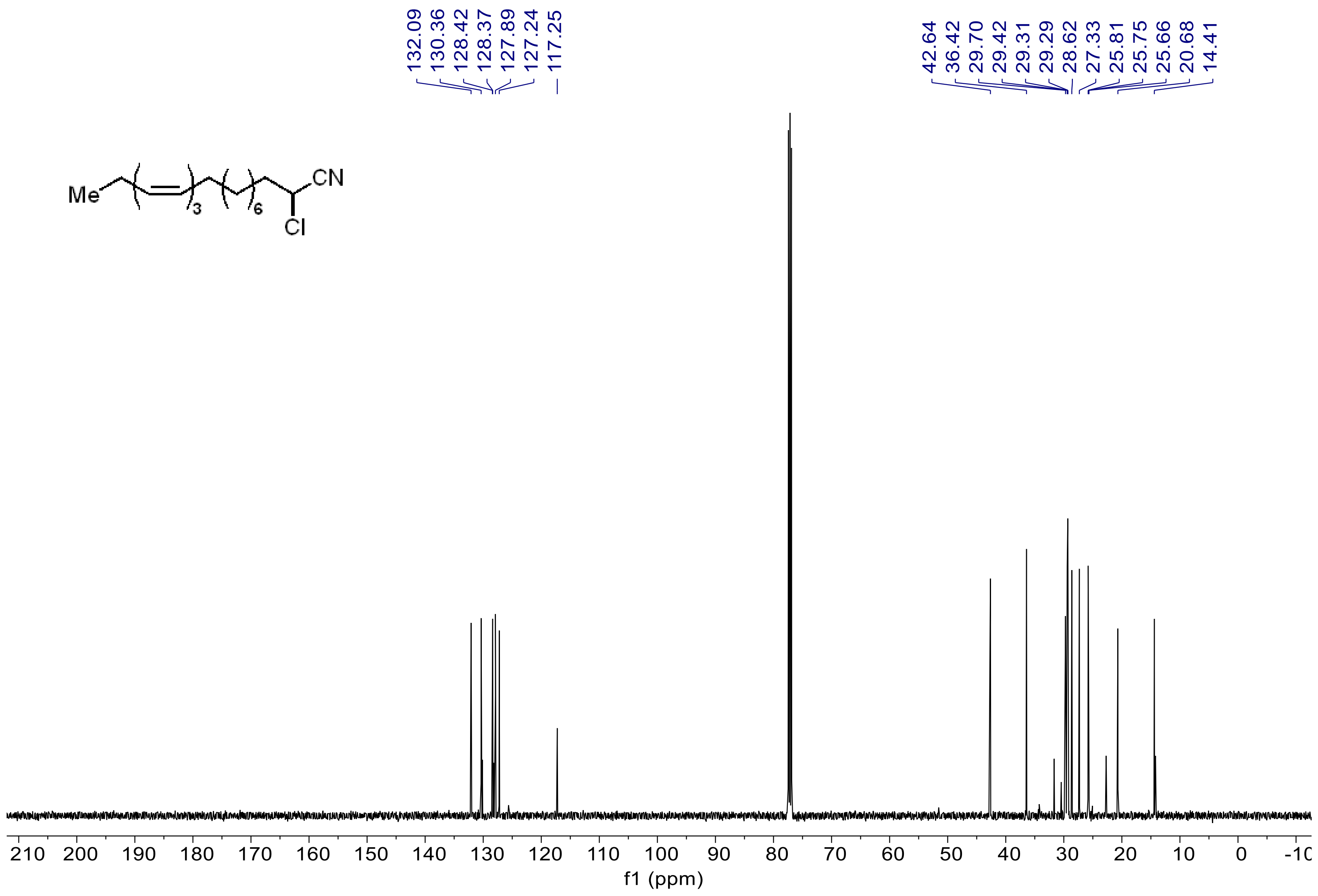

^{1}H NMR of *rac*-fenbufen-derived α-chloronitrile 24

CDCl$_3$, 23 °C

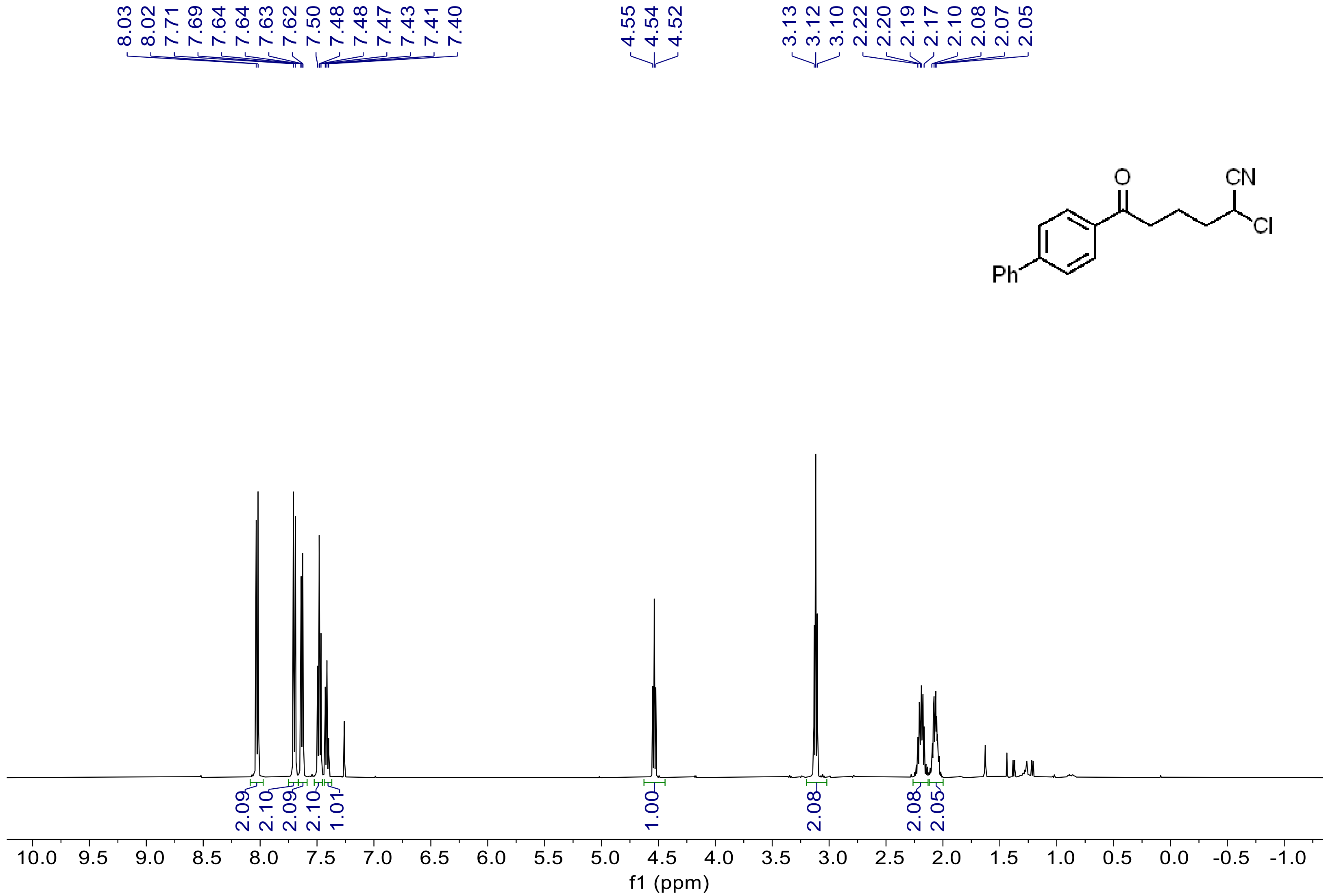

^{13}C NMR of *rac*-fenbufen-derived α-chloronitrile 24

$CDCl_3$, 23 °C

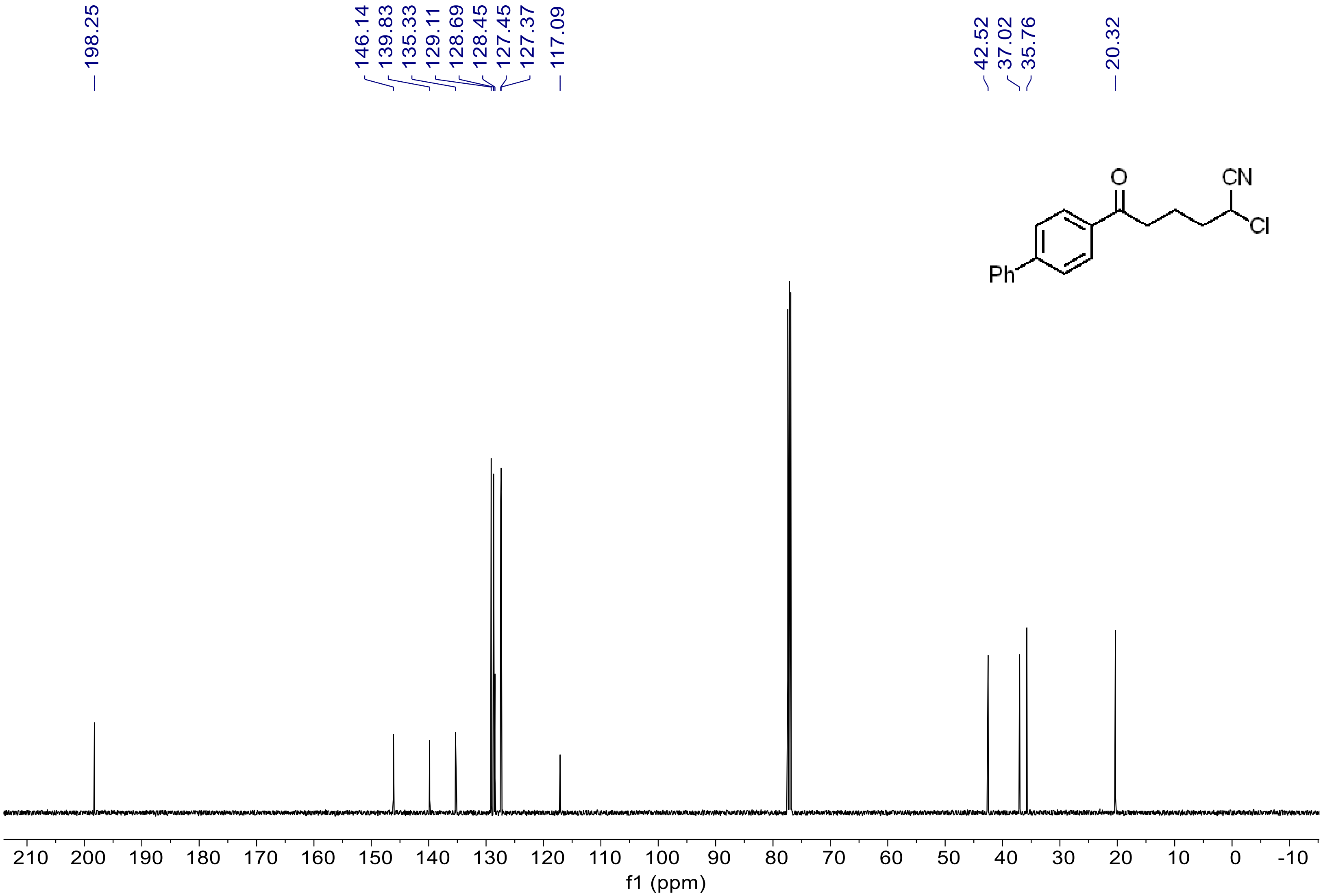

^{1}H NMR of *rac*-fenofibric acid-derived α-chloronitrile 25

$CDCl_3$, 23 °C

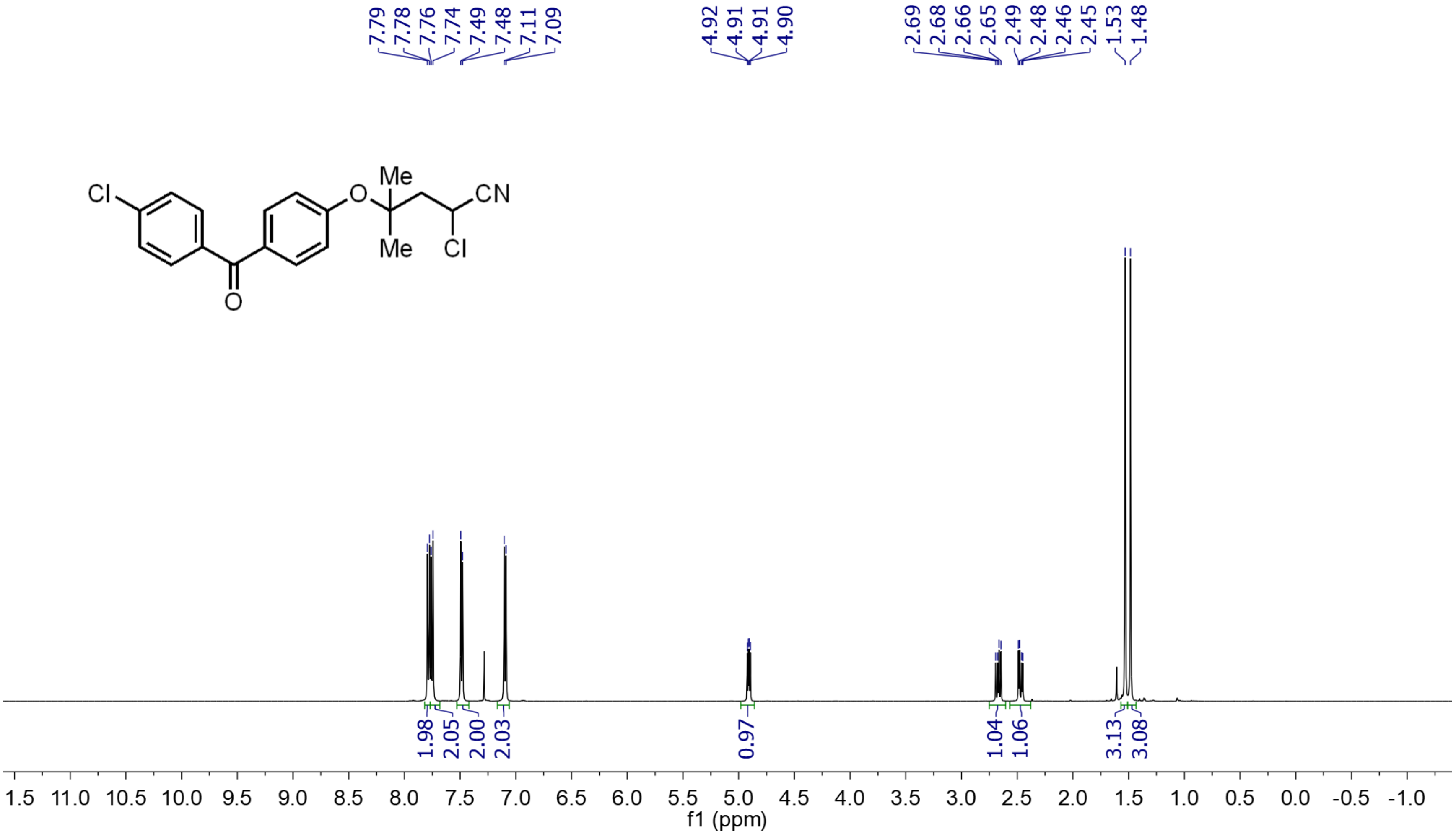

^{13}C NMR of *rac*-fenofibric acid-derived α-chloronitrile 25

$CDCl_3$, 23 °C

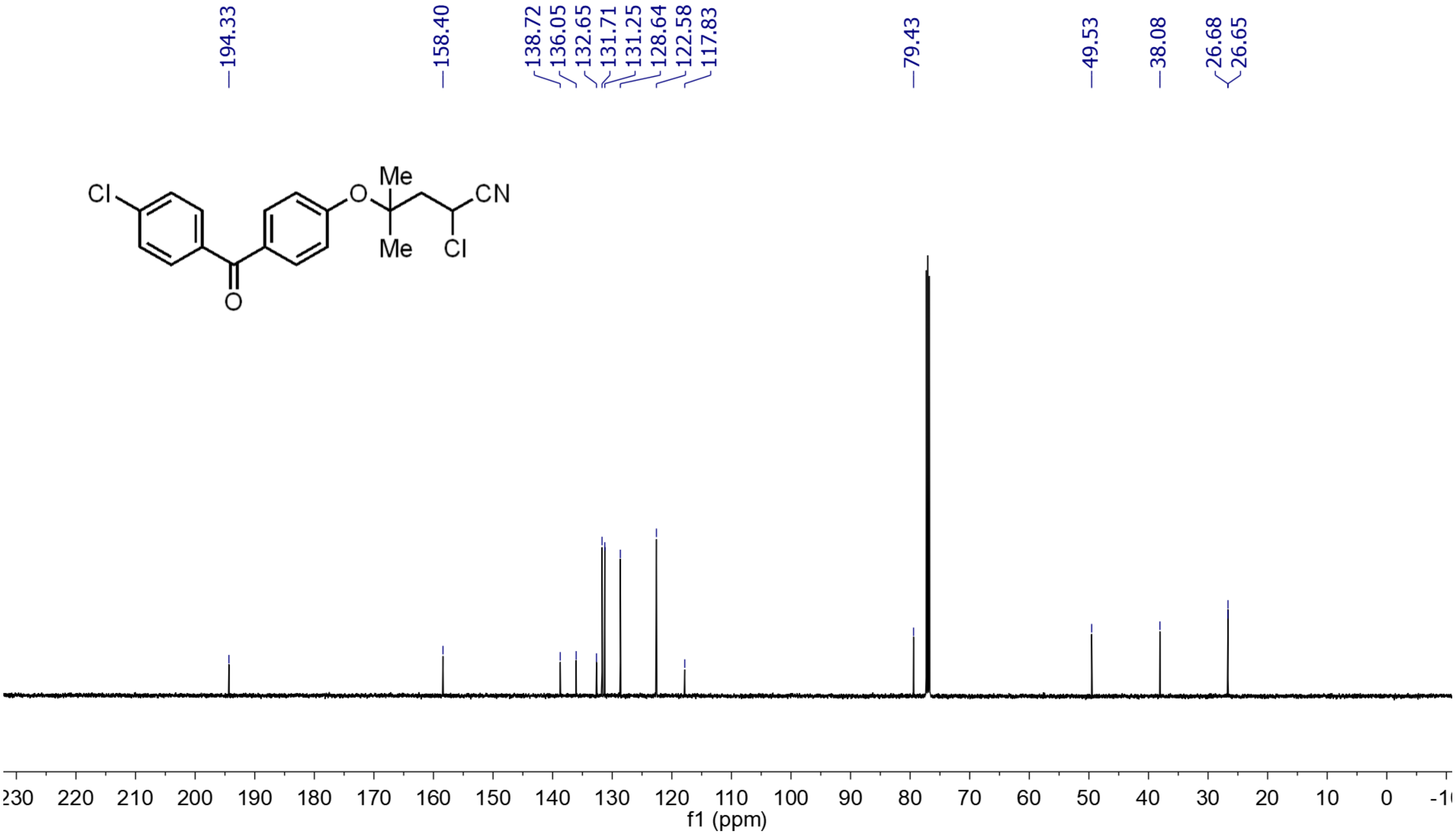

^{1}H NMR of *rac*-2-chloro-7-phenylheptanal 26

$CDCl_3$, 23 °C

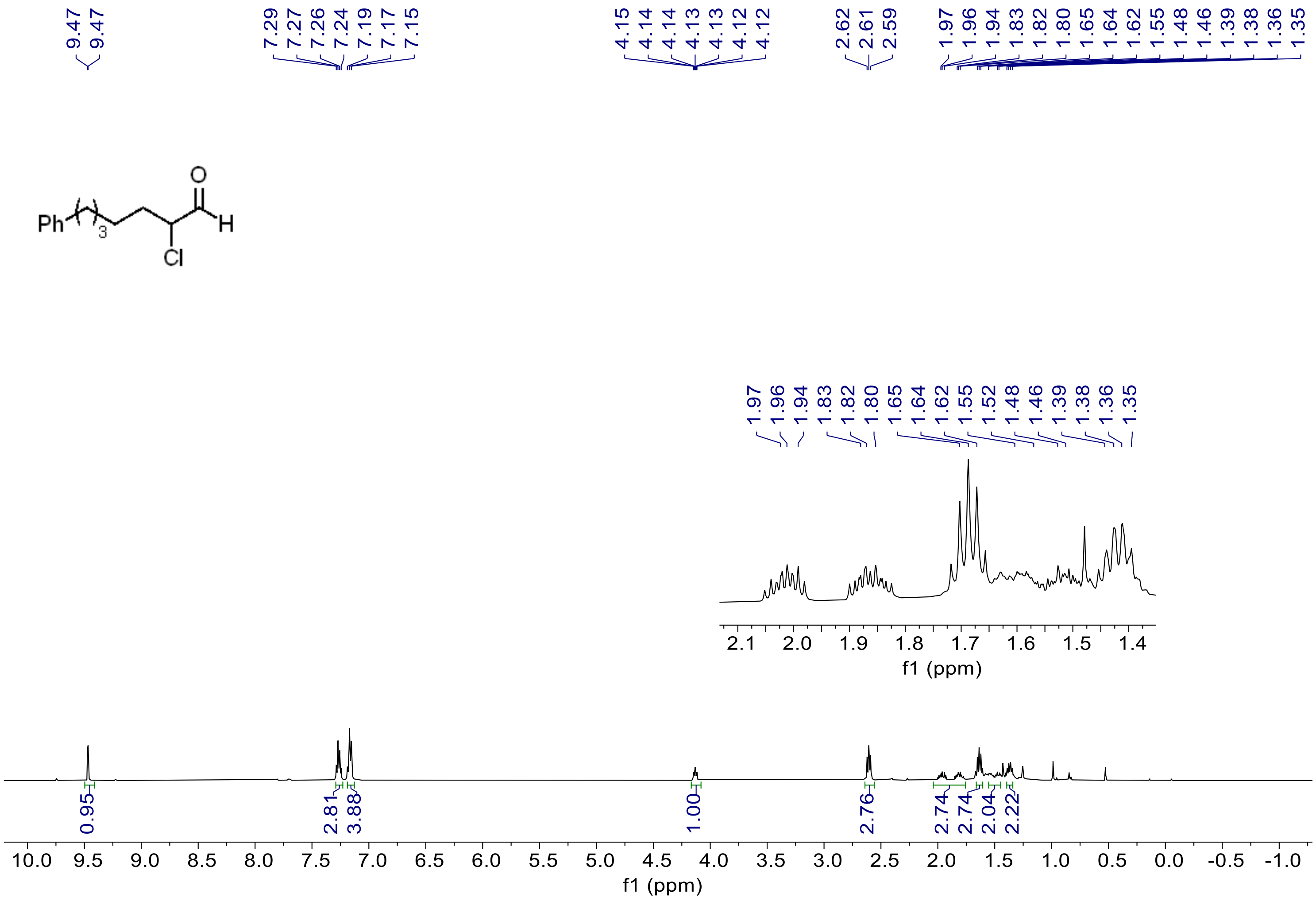

^{13}C NMR of *rac*-2-chloro-7-phenylheptanal 26

CDCl$_3$, 23 °C

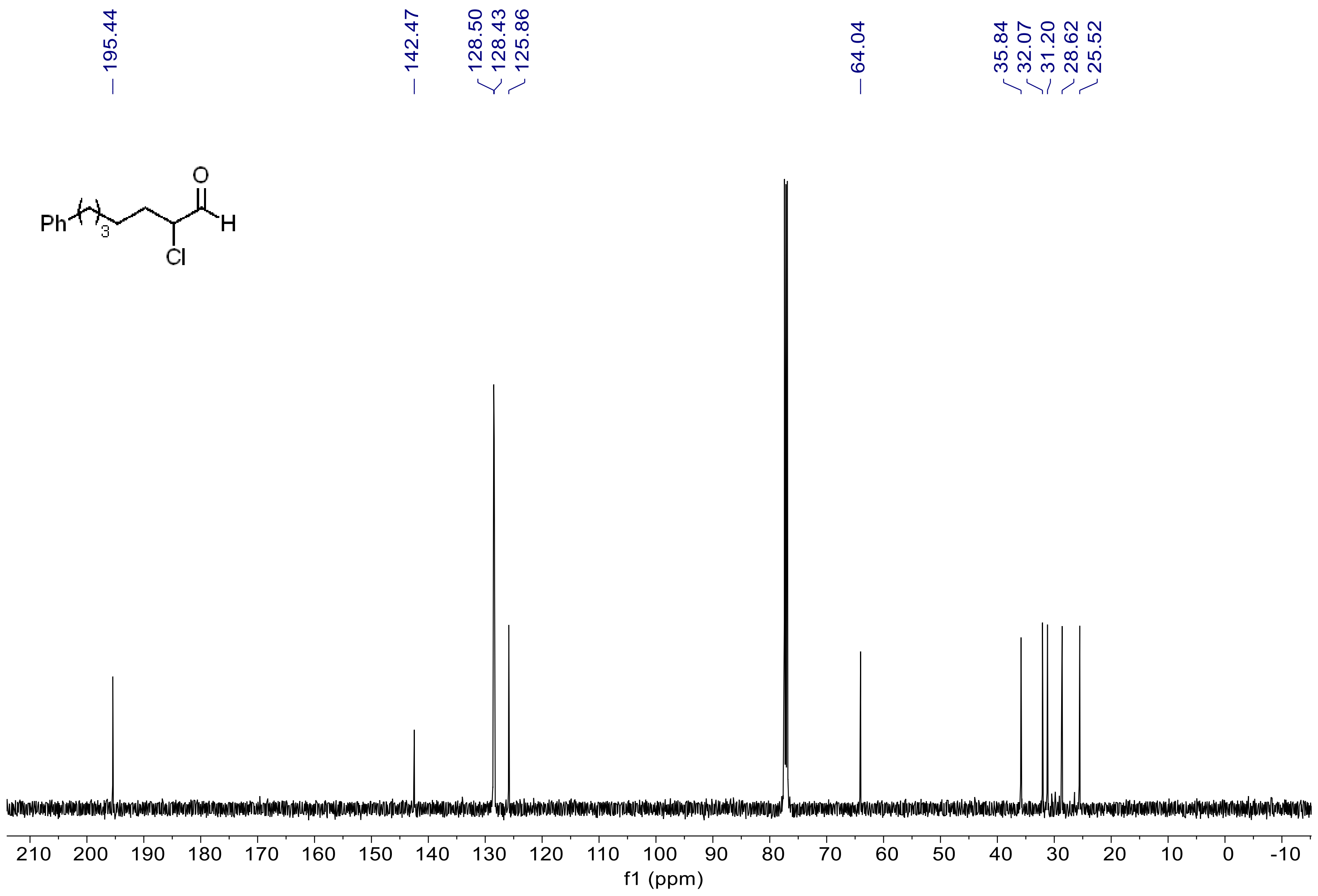

^{1}H NMR of *rac*-4-chloro-8-phenyloctan-2-one 27

$CDCl_3$, 23 °C

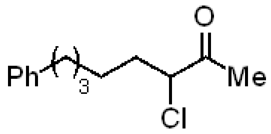

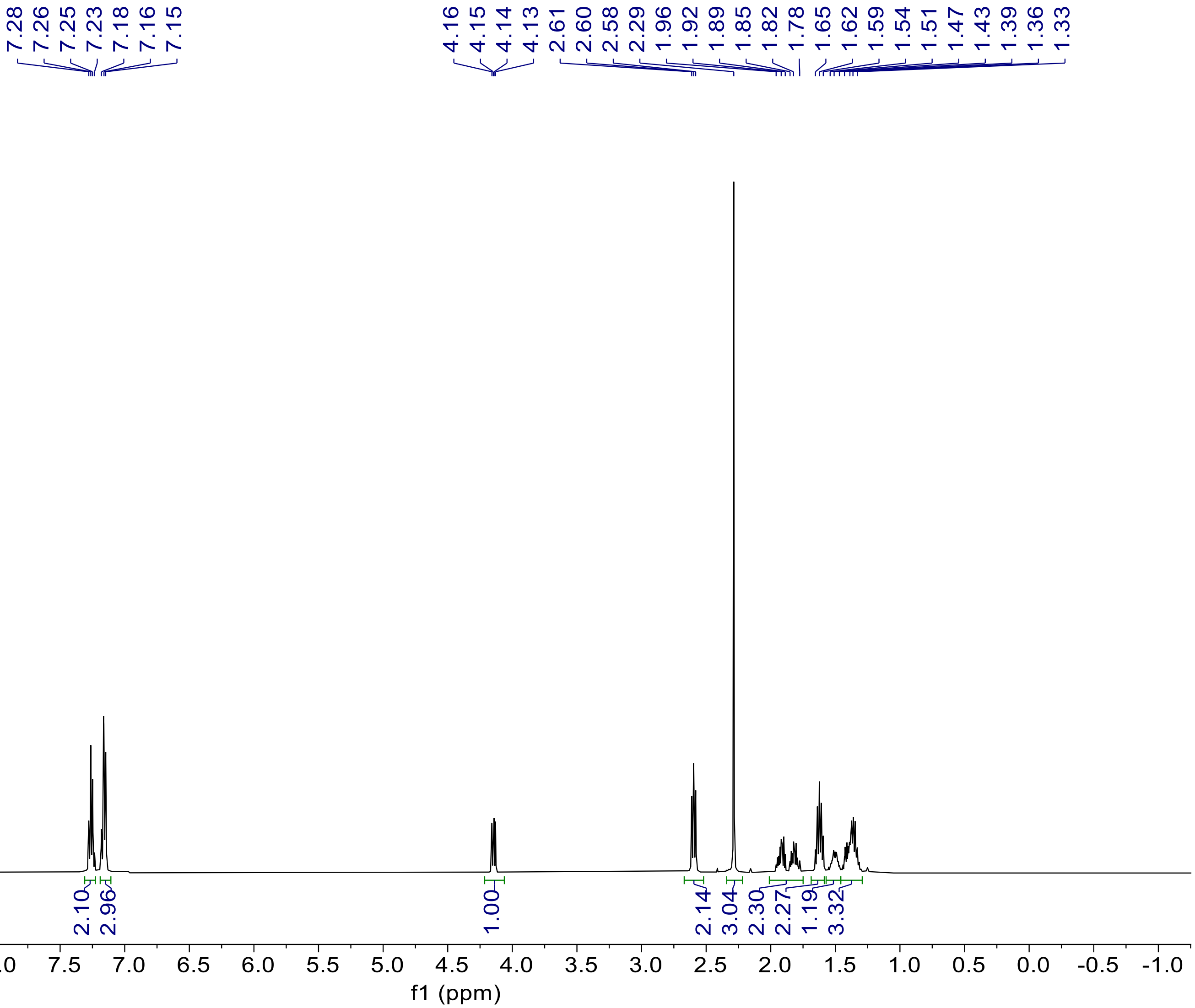

^{13}C NMR of *rac*-4-chloro-8-phenyloctan-2-one 27

CDCl$_3$, 23 °C

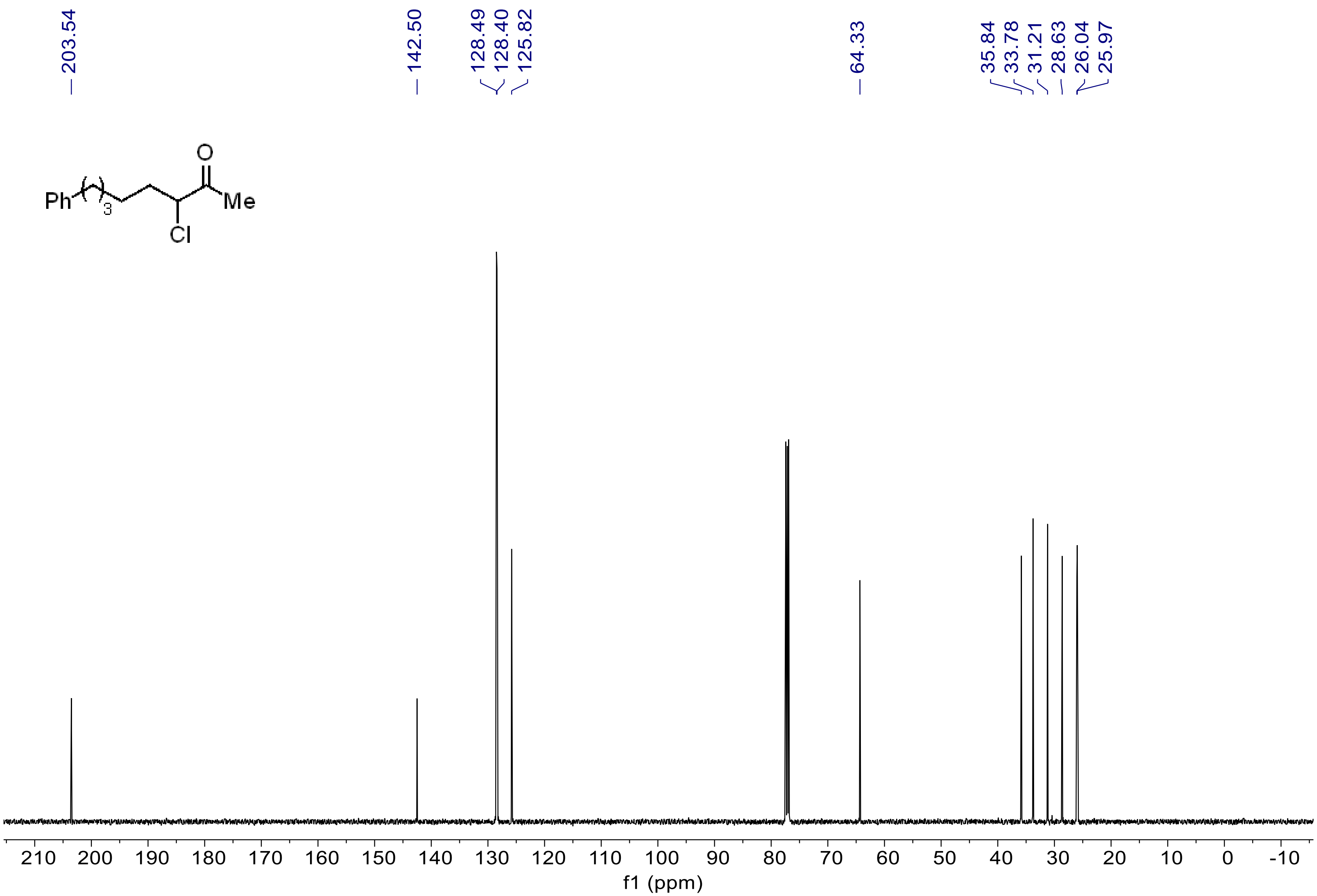

^{1}H NMR of *rac*-*tert*-butyl 2-chloro-7-phenylheptanoate 28

$CDCl_3$, 23 °C

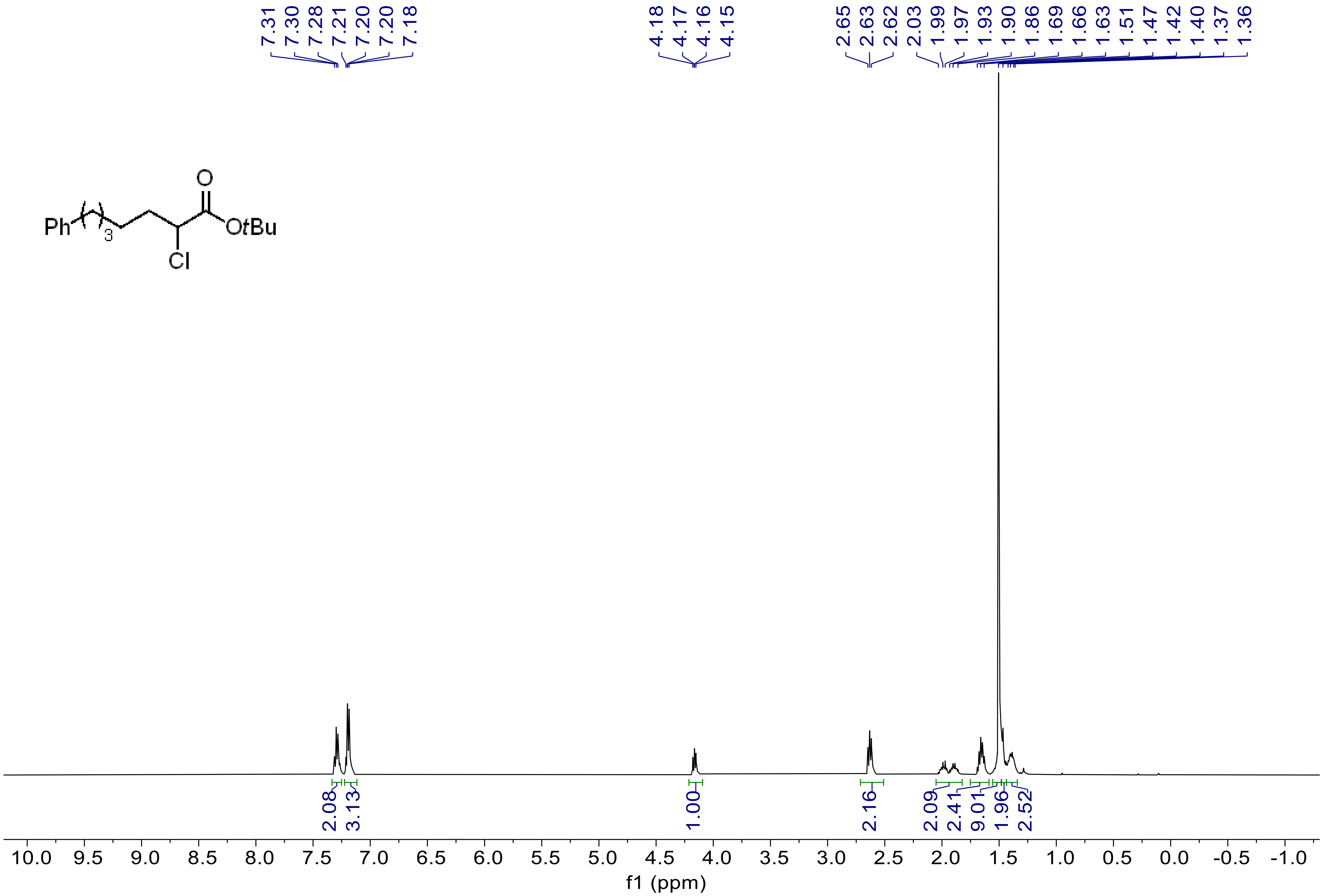

^{13}C NMR of *rac-tert*-butyl 2-chloro-7-phenylheptanoate 28

$CDCl_3$, 23 °C

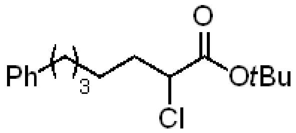

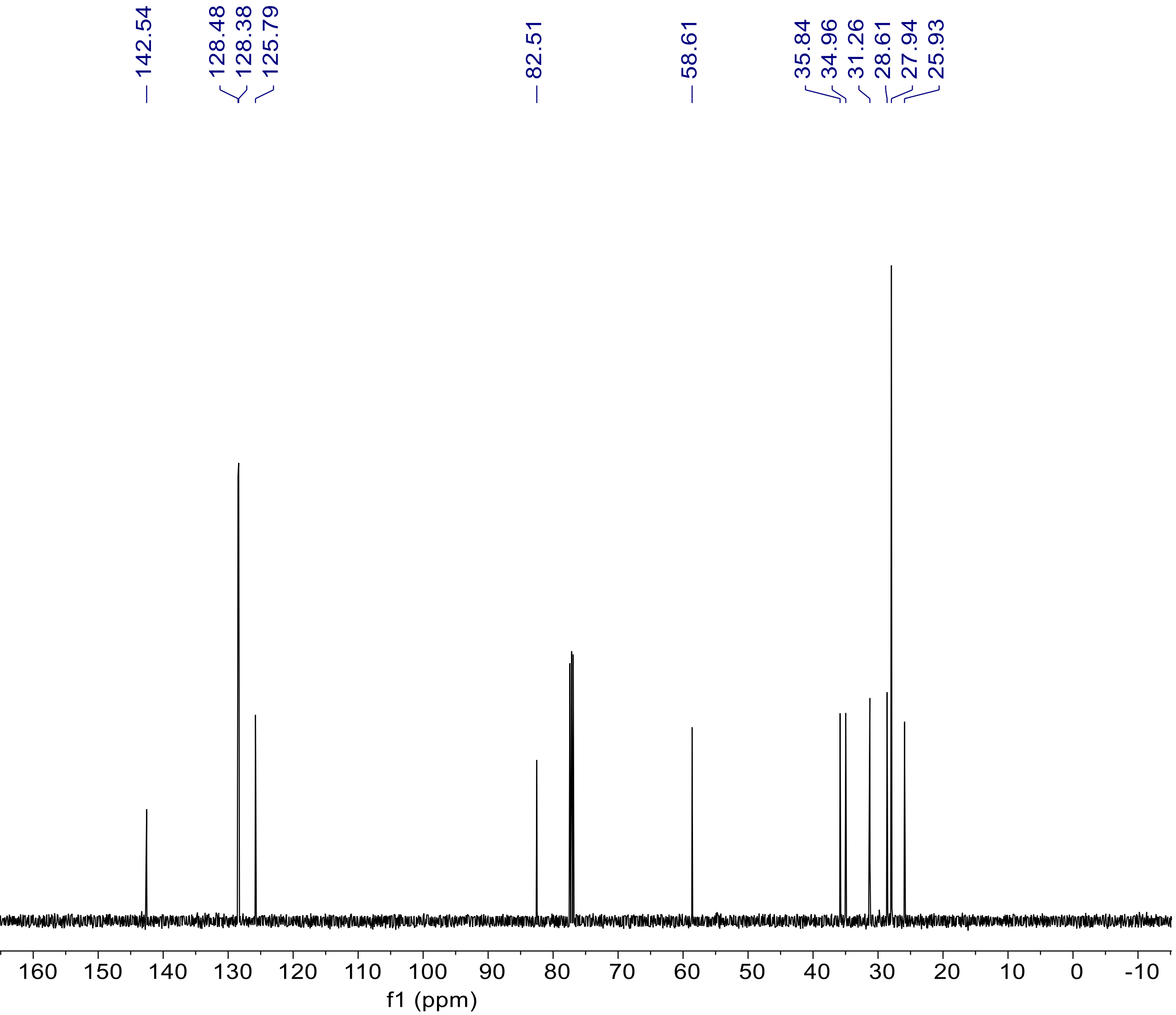

^{1}H NMR of *rac*-2-chloro-*N,N*-dimethyl-7-phenylheptanamide 29

$CDCl_3$, 23 °C

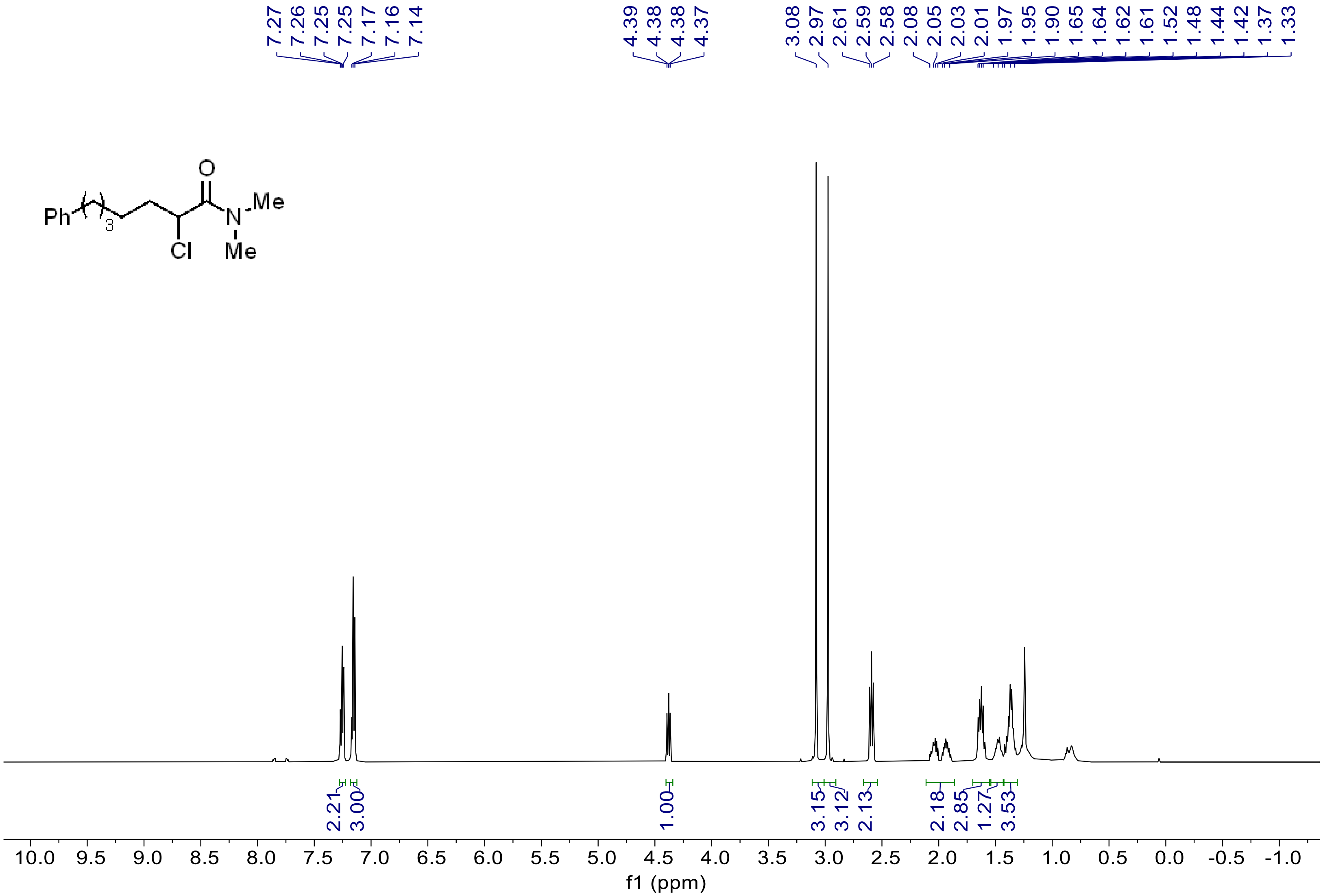

^{13}C NMR of *rac*-2-chloro-*N,N*-dimethyl-7-phenylheptanamide 29

$CDCl_3$, 23 °C

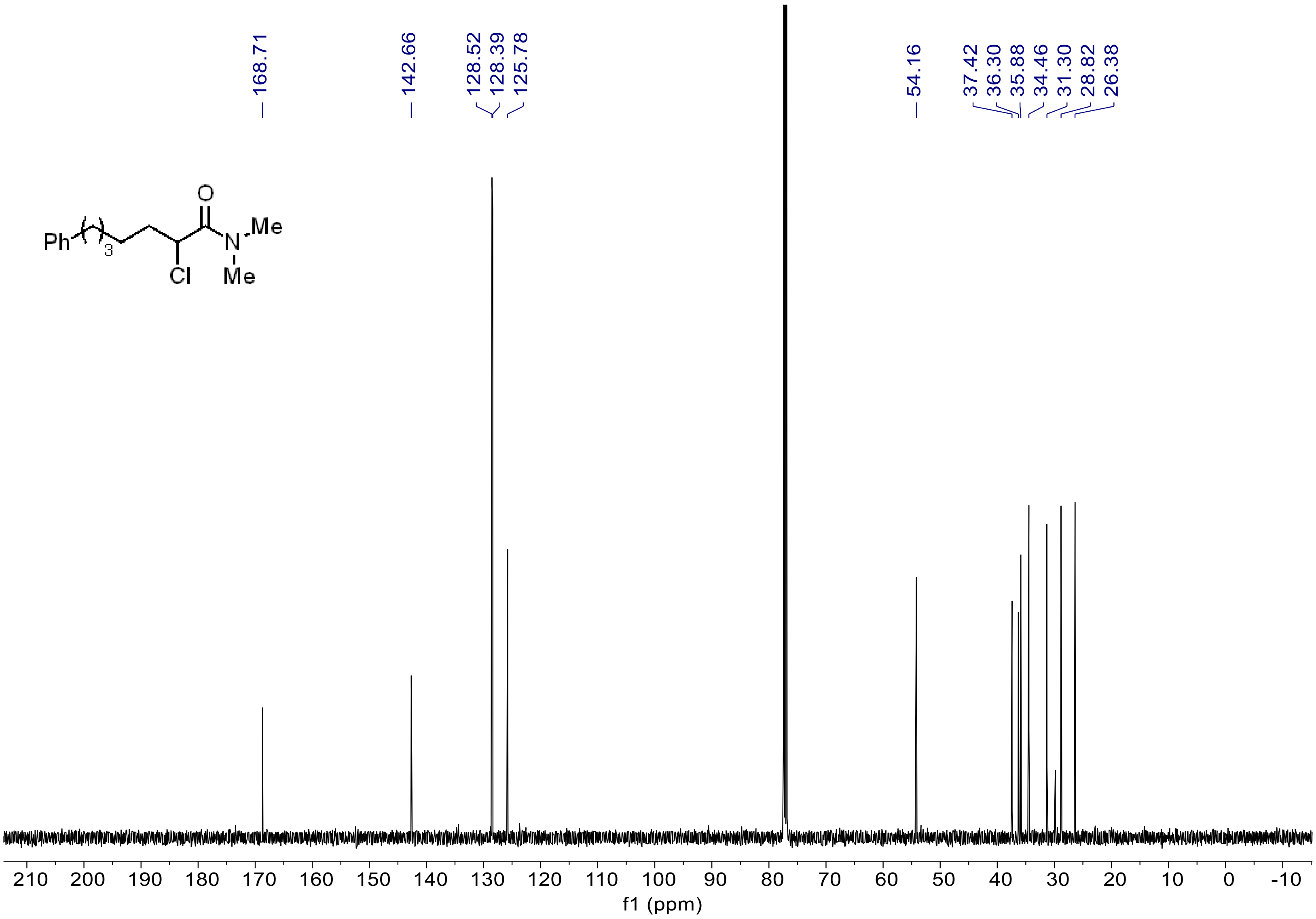

^{1}H NMR of *rac*-(1-chlorohexane-1,6-diyl)dibenzene 30

$CDCl_3$, 23 °C

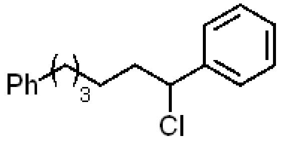

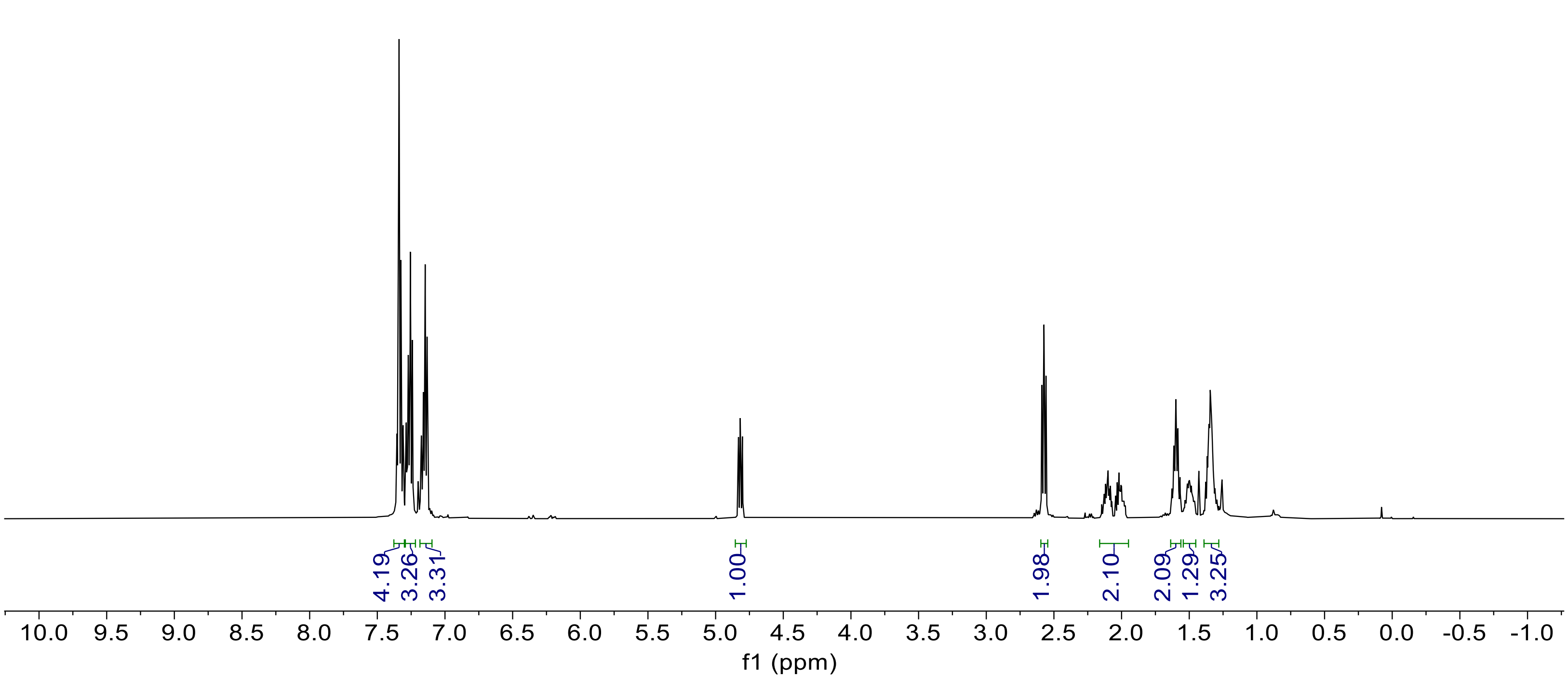

^{13}C NMR of *rac*-(1-chlorohexane-1,6-diyl)dibenzene 30

$CDCl_3$, 23 °C

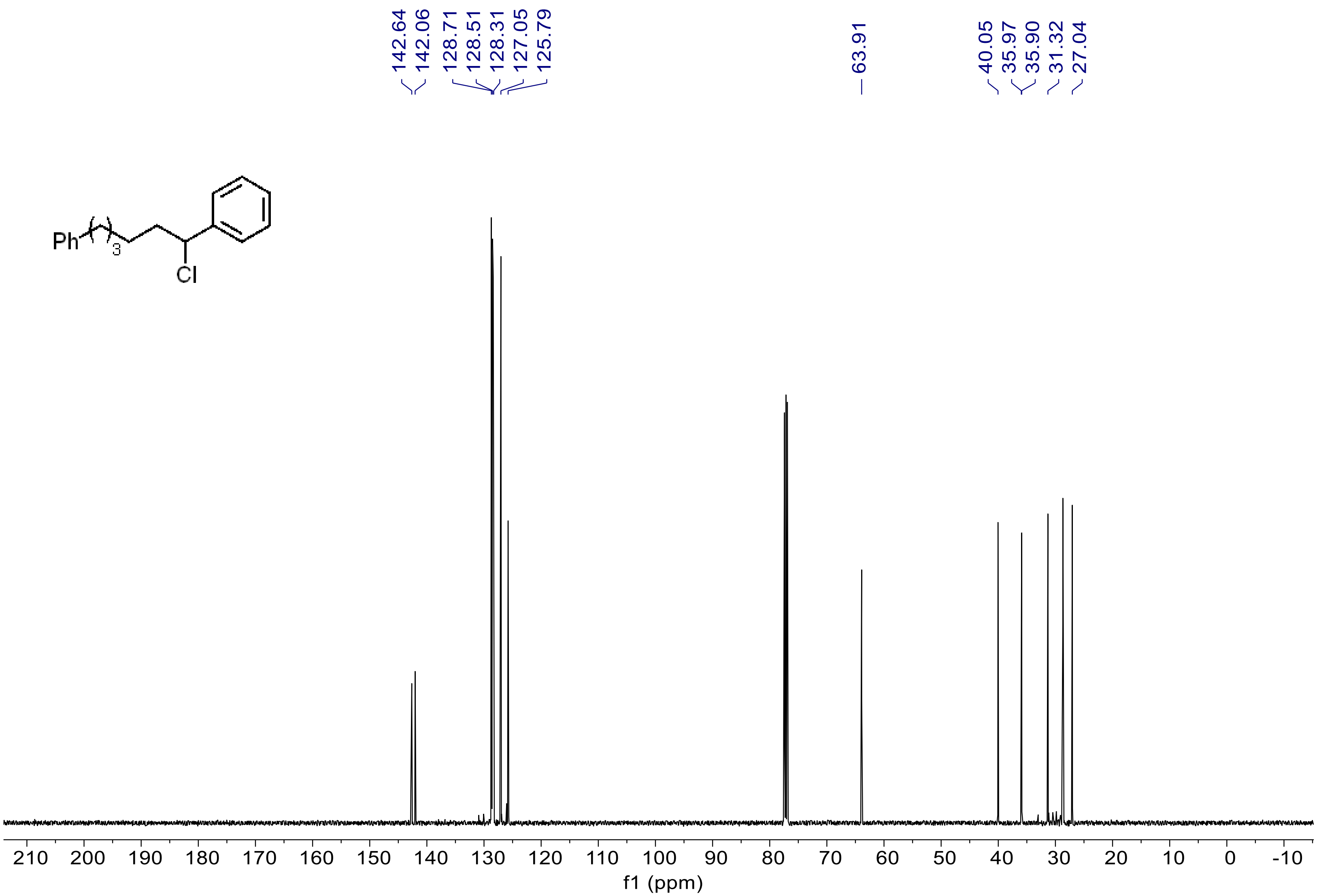

^{1}H NMR of *rac*-1-(1-chloro-6-phenylhexyl)-4-(trifluoromethyl)benzene 31

$CDCl_3$, 23 °C

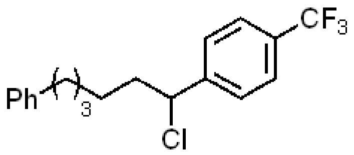

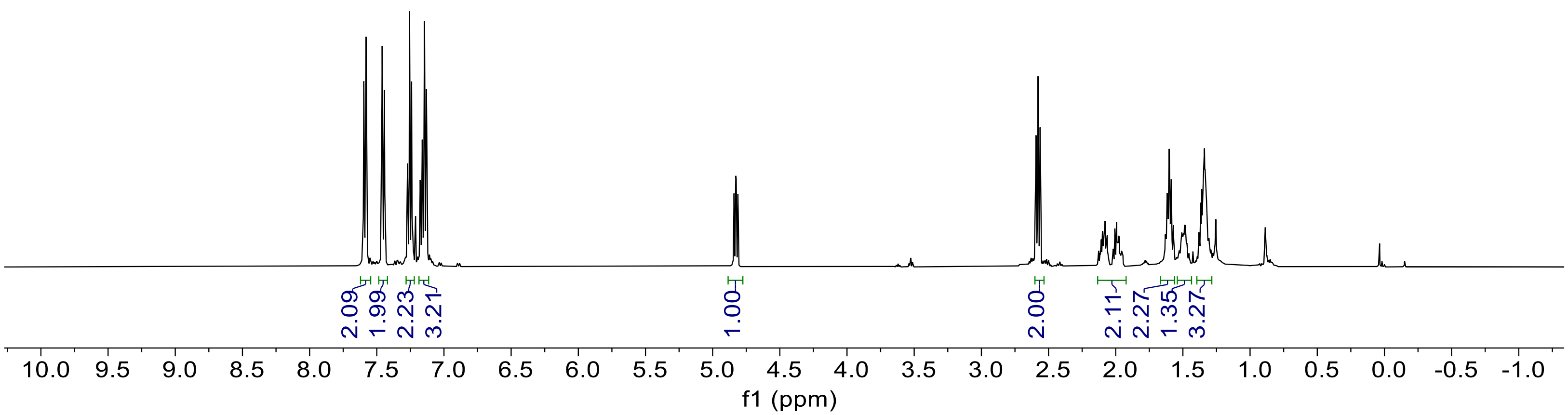

^{19}F NMR of *rac*-1-(1-chloro-6-phenylhexyl)-4-(trifluoromethyl)benzene 31

CDCl$_3$, 23 °C

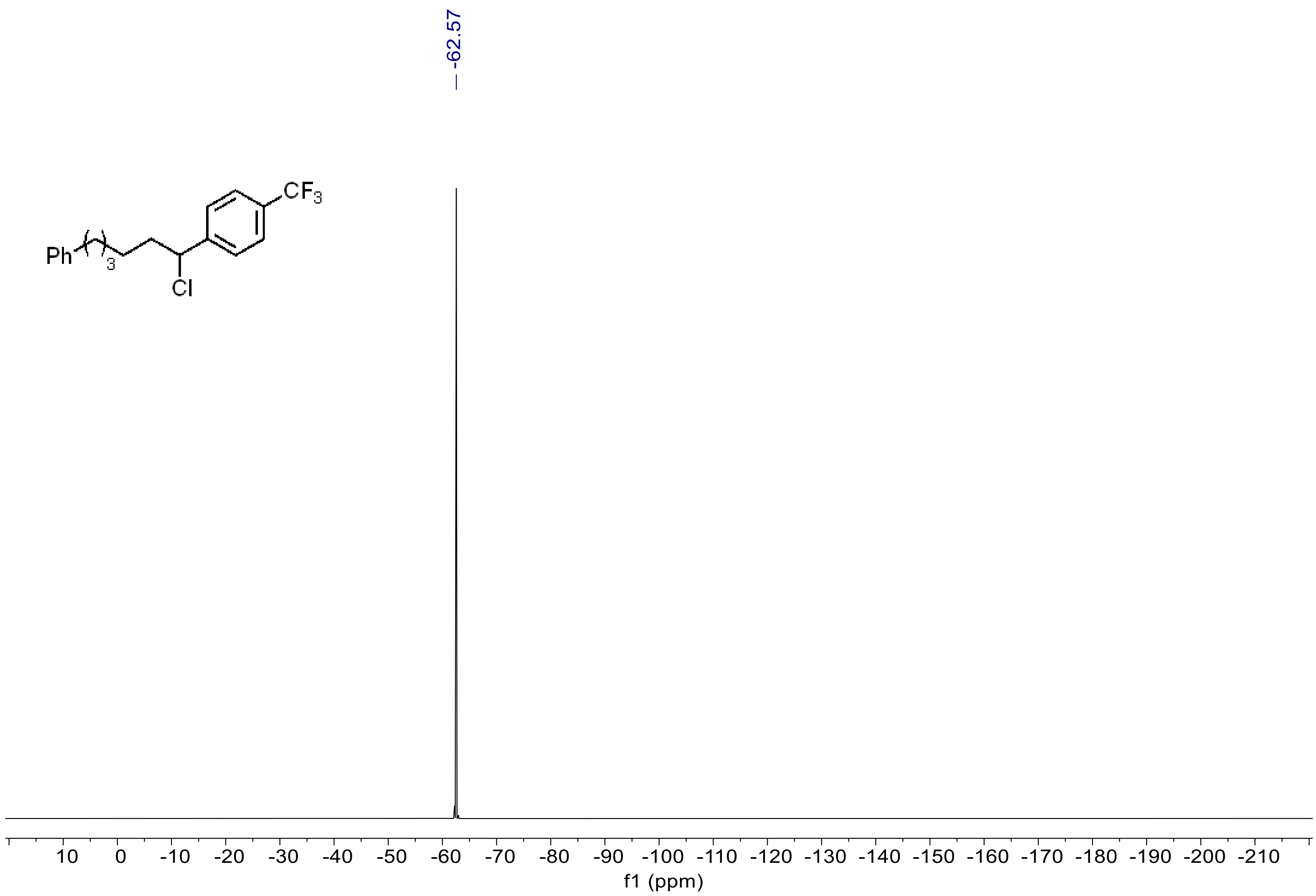

^{13}C NMR of *rac*-1-(1-chloro-6-phenylhexyl)-4-(trifluoromethyl)benzene 31

CDCl3, 23 °C

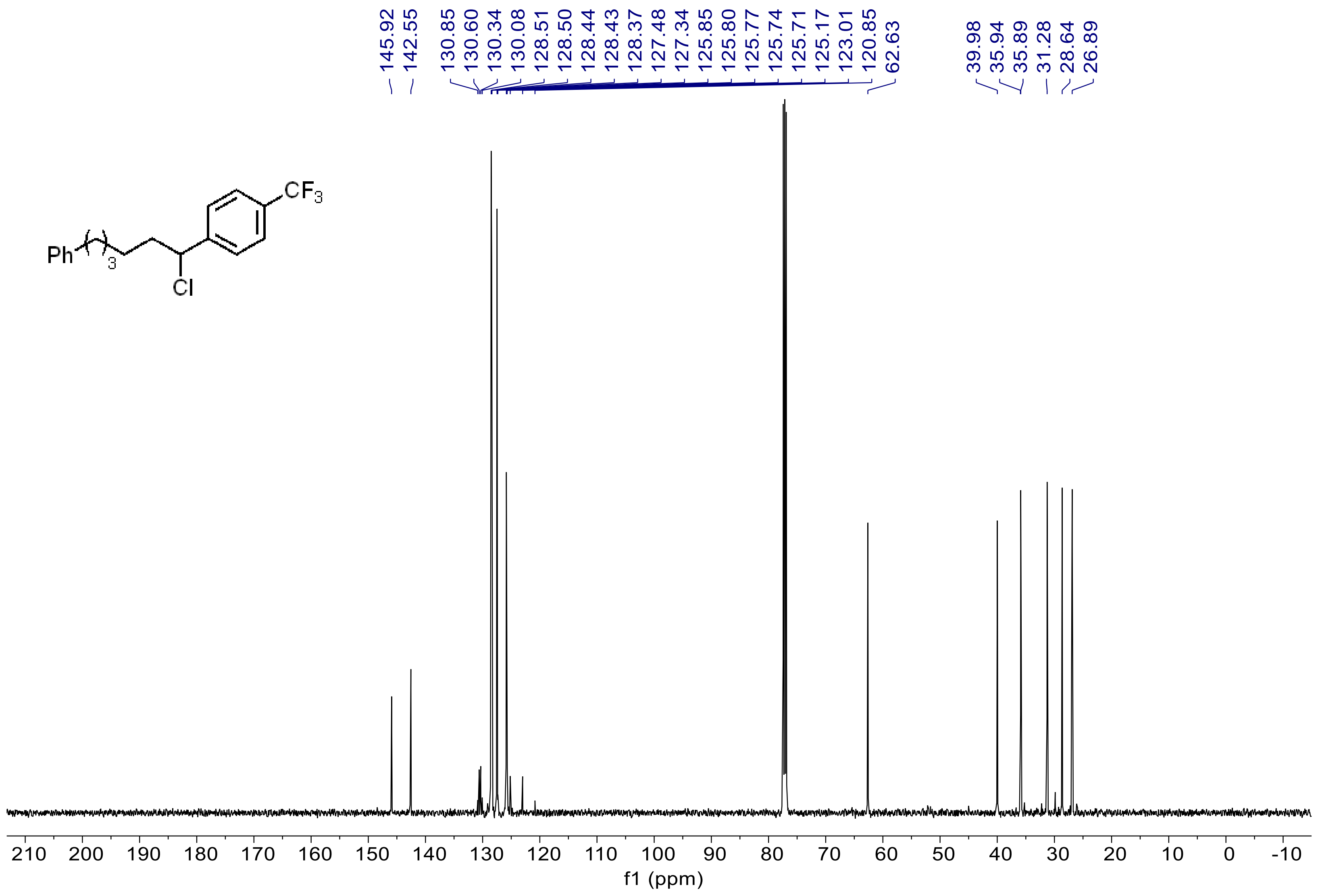

^{1}H NMR of *rac*-3-(2-chloro-7-phenylheptanoyl)oxazolidin-2-one 32

$CDCl_3$, 23 °C

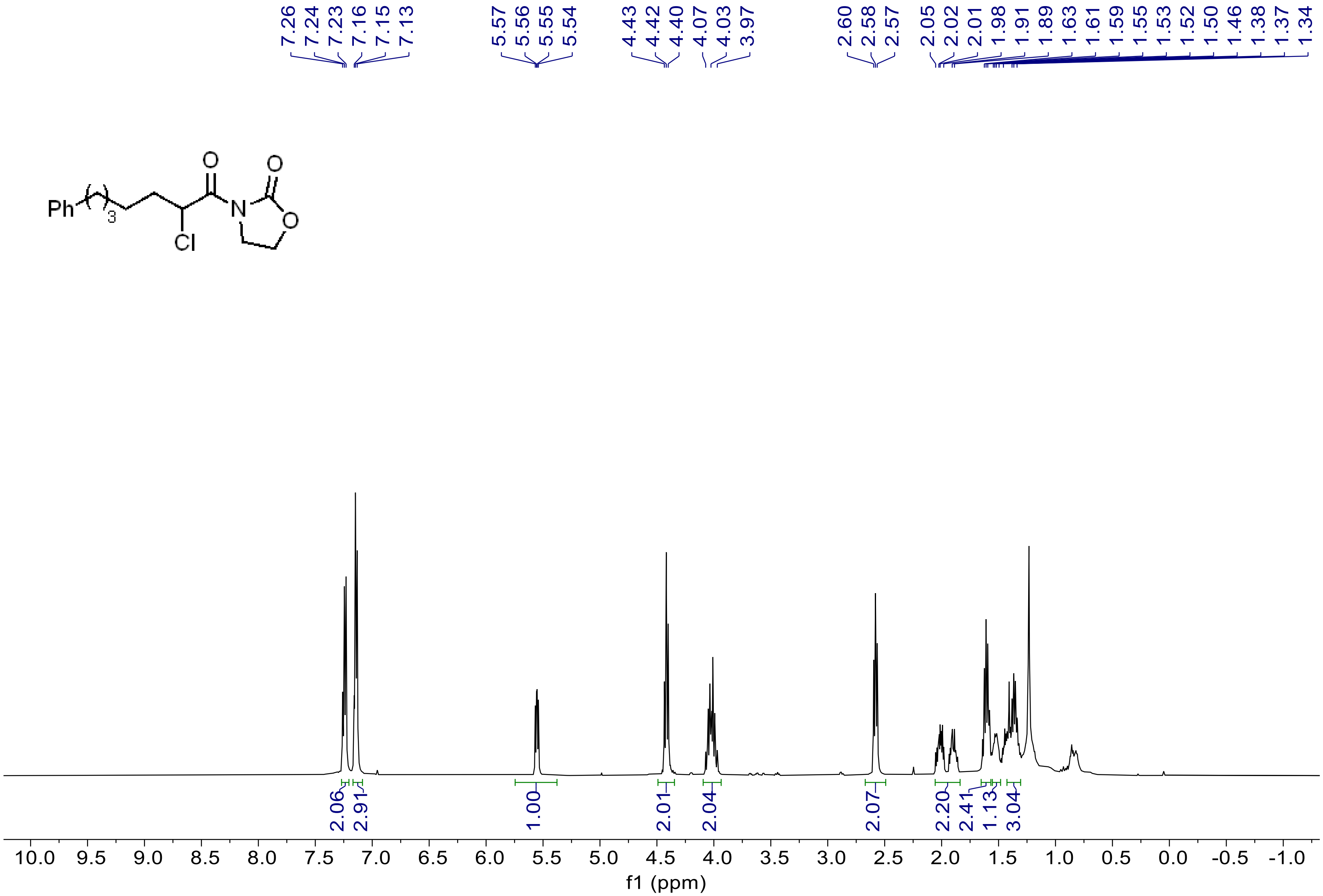

^{13}C NMR of *rac*-3-(2-chloro-7-phenylheptanoyl)oxazolidin-2-one 32

$CDCl_3$, 23 °C

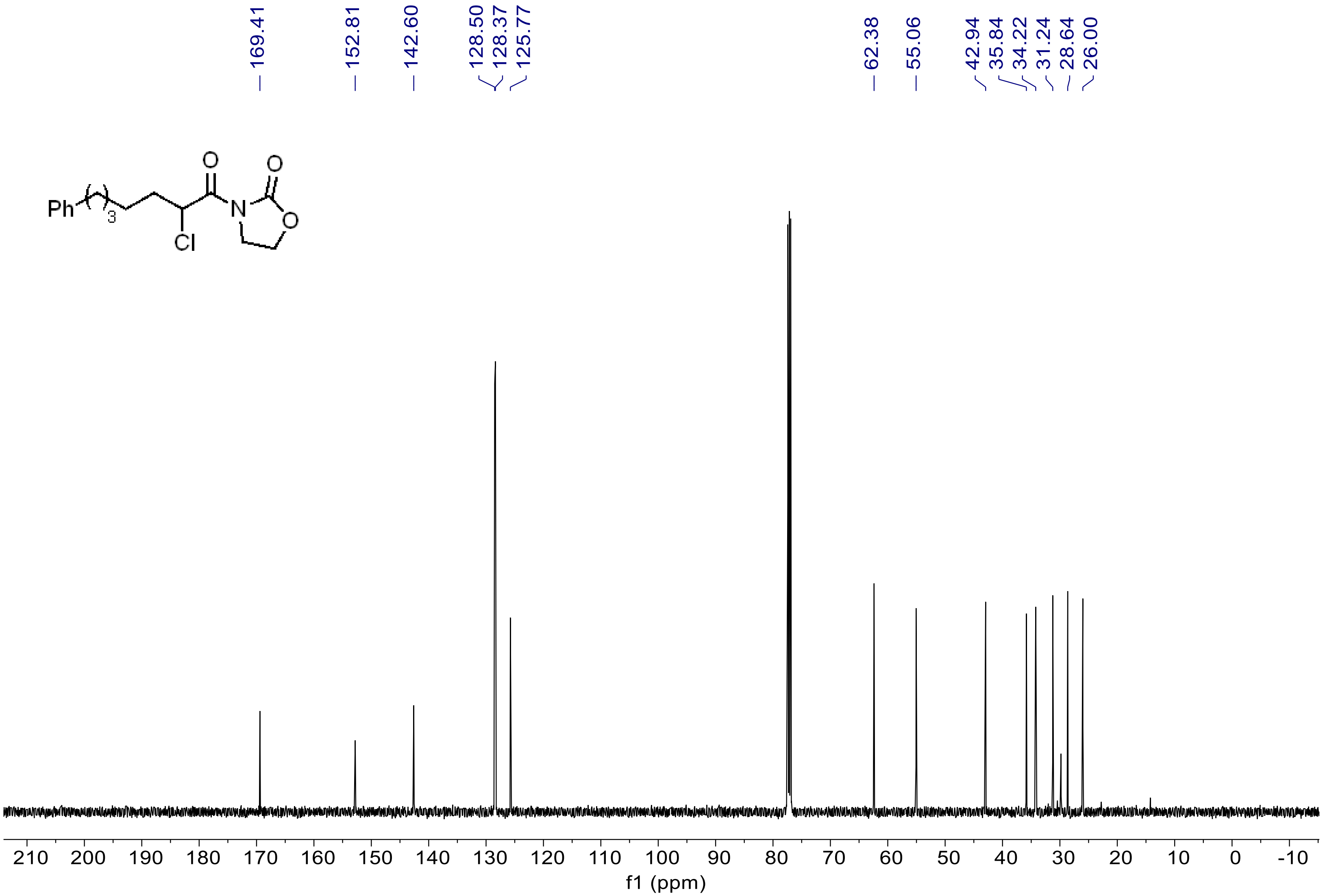

^{1}H NMR of *rac*-2-(1-chloro-6-phenylhexyl)-4,4,5,5-tetramethyl-1,3,2-dioxaborolane 33

$CDCl_3$, 23 °C

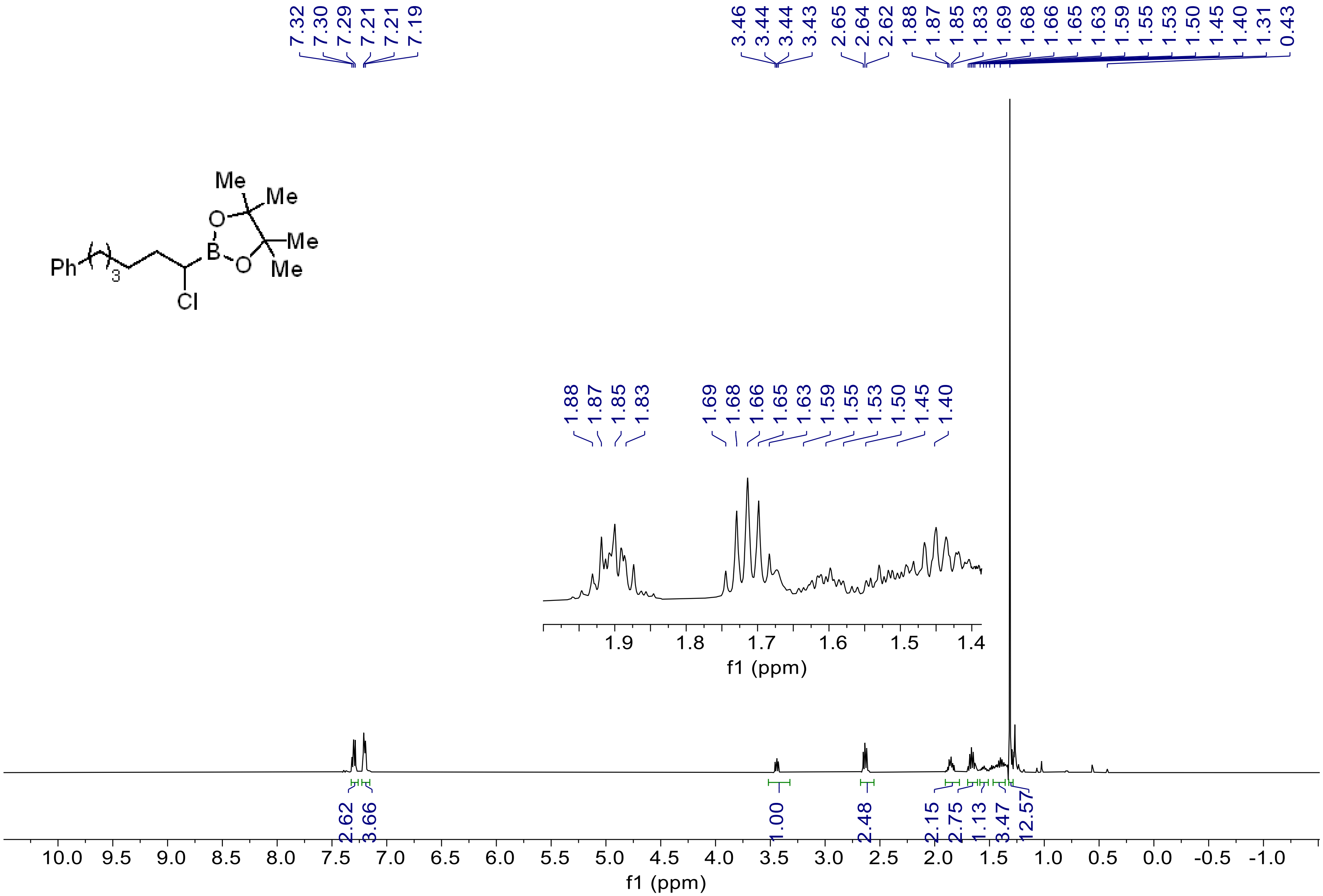

^{13}C NMR of *rac*-2-(1-chloro-6-phenylhexyl)-4,4,5,5-tetramethyl-1,3,2-dioxaborolane 33

CDCl₃, 23 °C

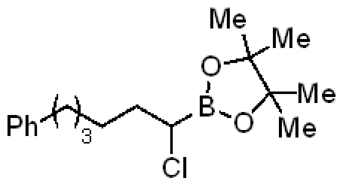

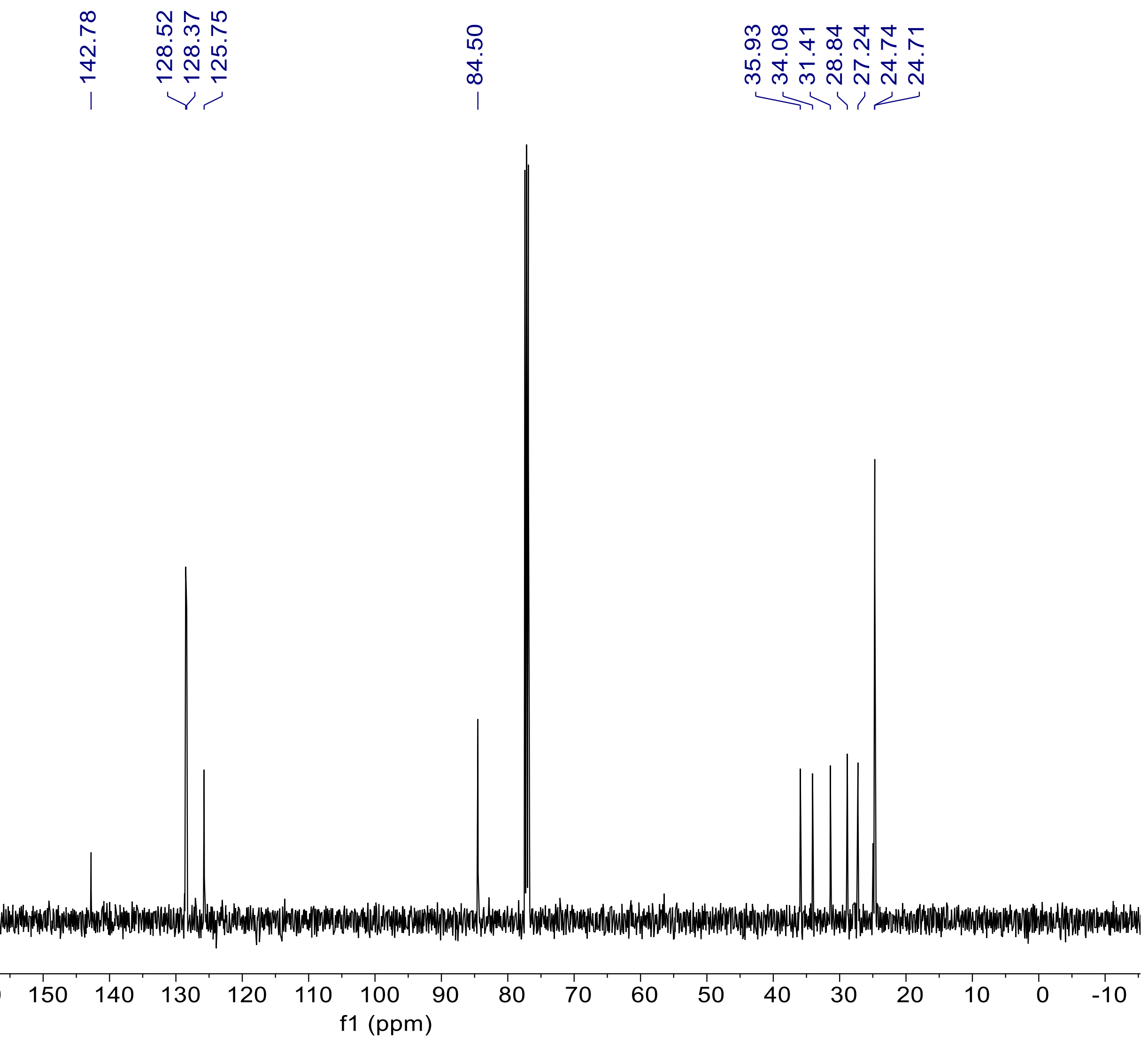

^{1}H NMR of *rac*-dimethyl 2-chloro-3-(4-phenylbutyl)succinate 34

$CDCl_3$, 23 °C

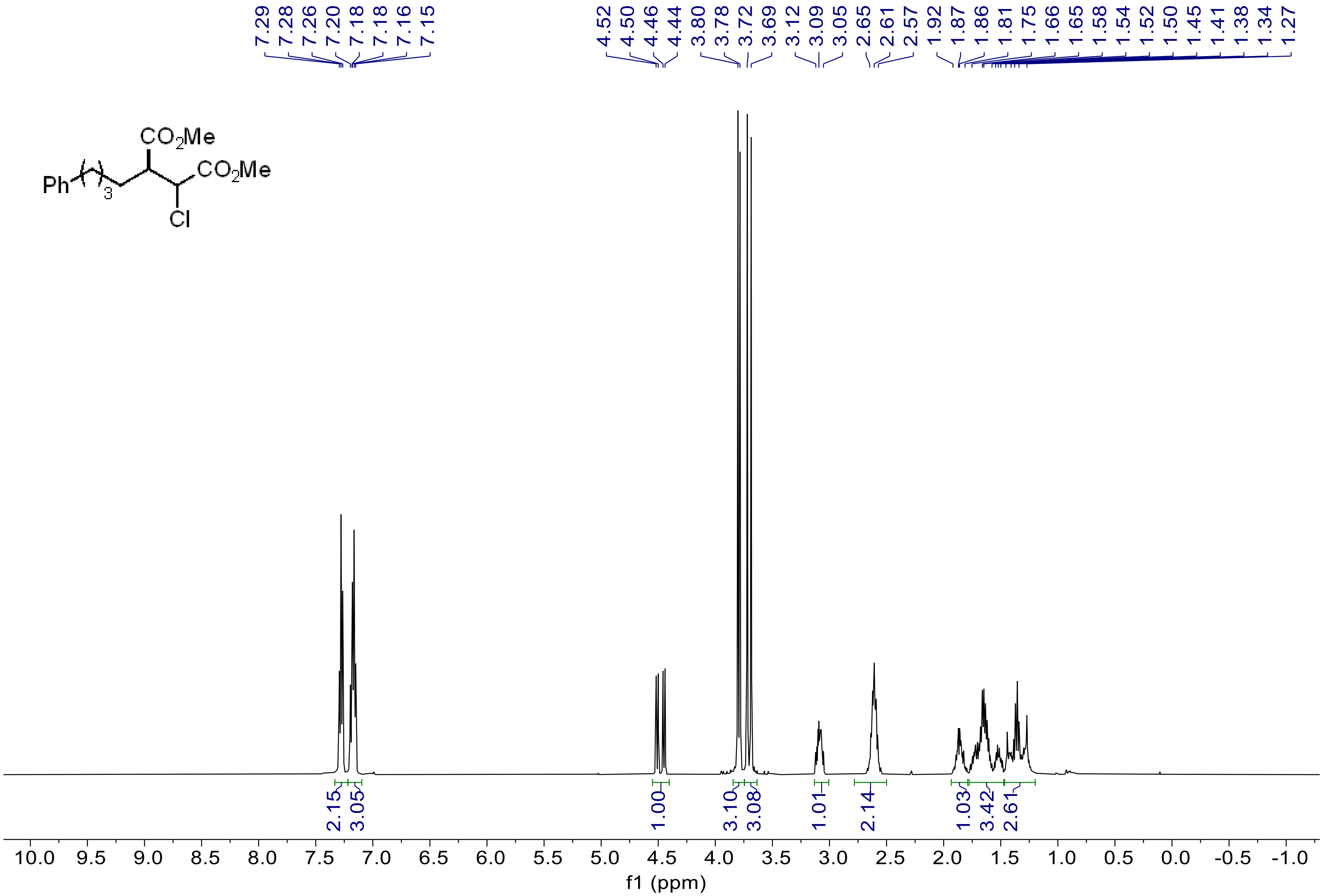

^{13}C NMR of *rac*-dimethyl 2-chloro-3-(4-phenylbutyl)succinate 34

$CDCl_3$, 23 °C

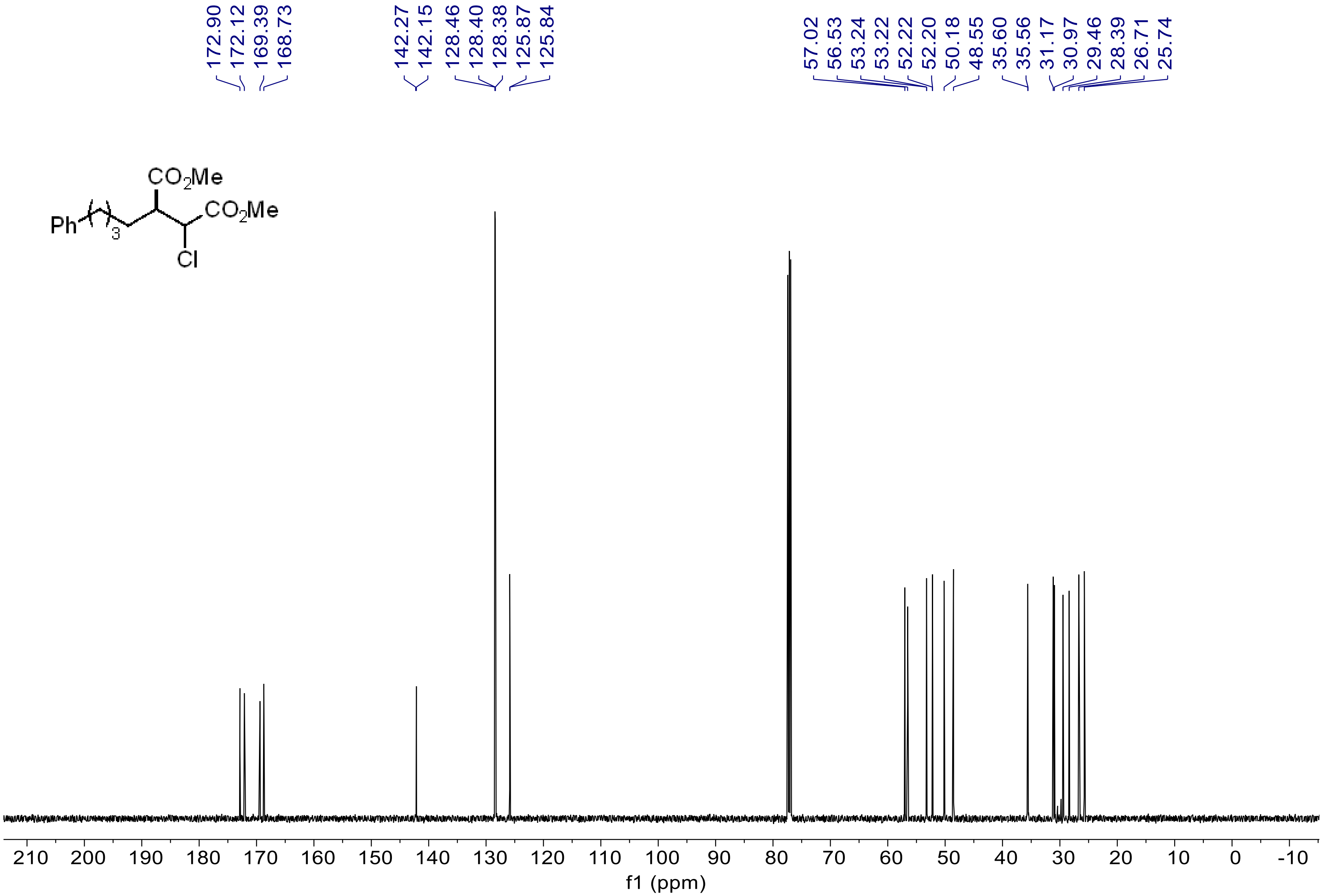

^{1}H NMR of *rac*-dimethyl 2-chloro-2-(5-phenylpentyl)succinate 35

$CDCl_3$, 23 °C

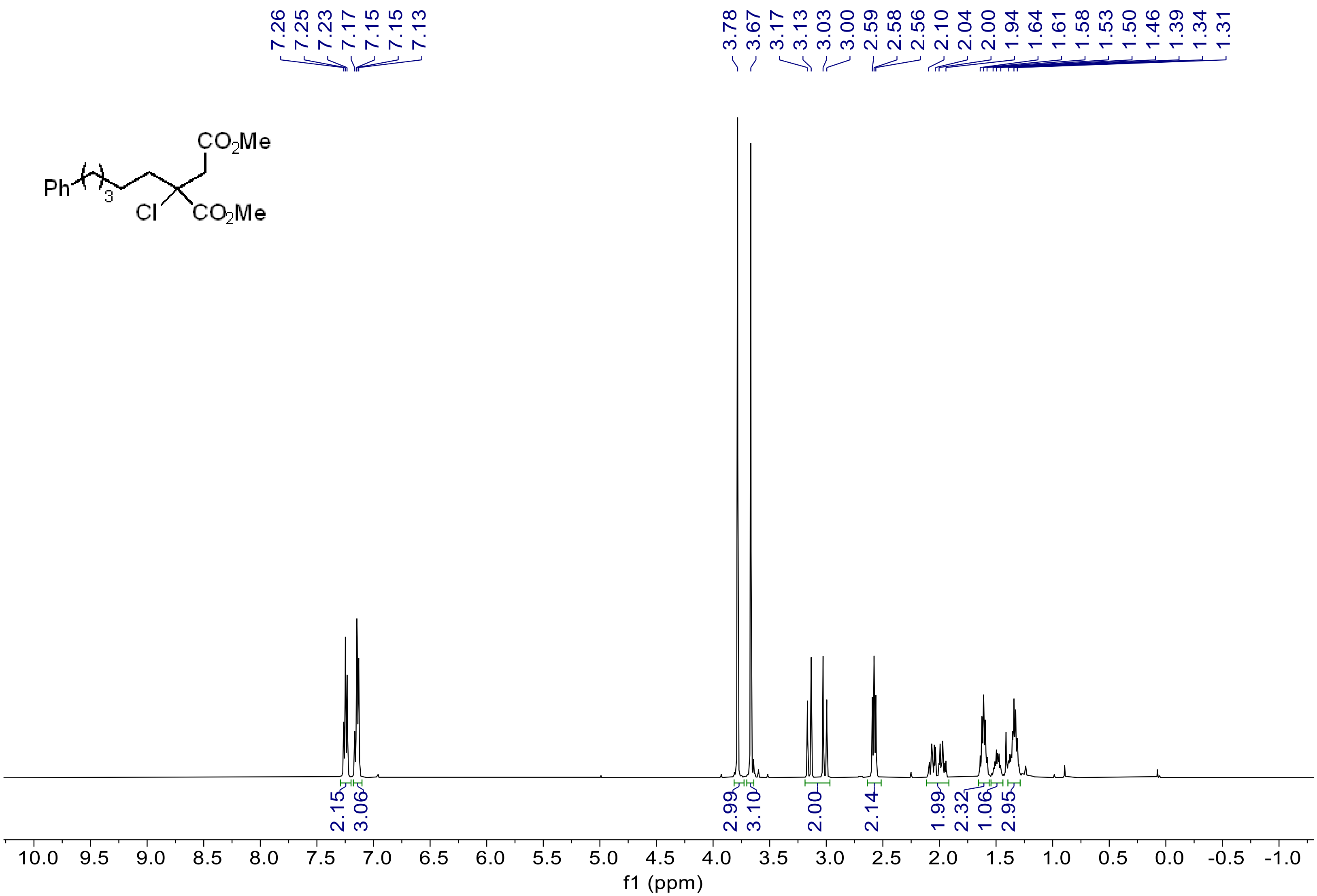

^{13}C NMR of *rac*-dimethyl 2-chloro-2-(5-phenylpentyl)succinate 35

$CDCl_3$, 23 °C

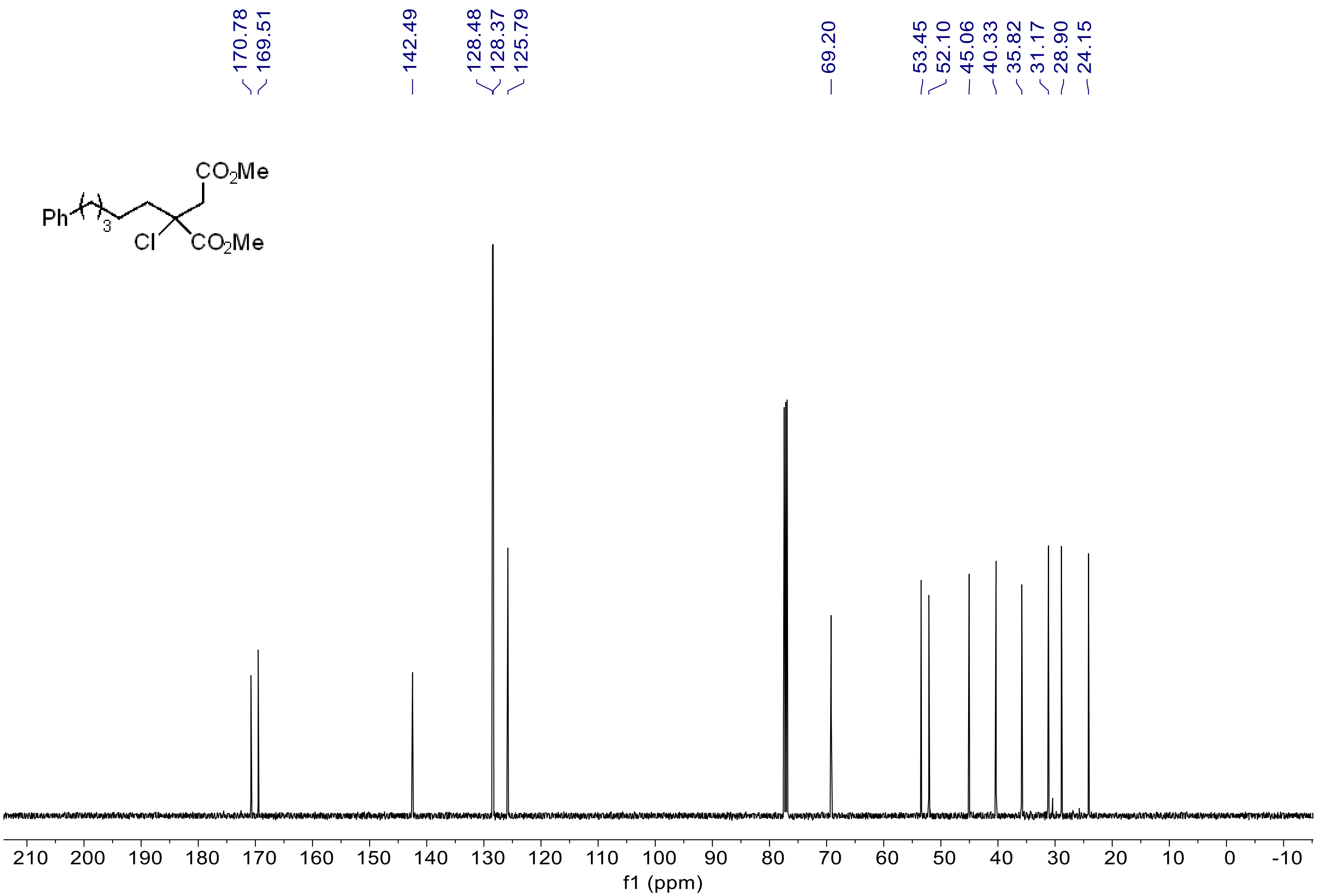

^{1}H NMR of *rac*-2-chloro-2-methyl-7-phenylheptanal 36

$CDCl_3$, 23 °C

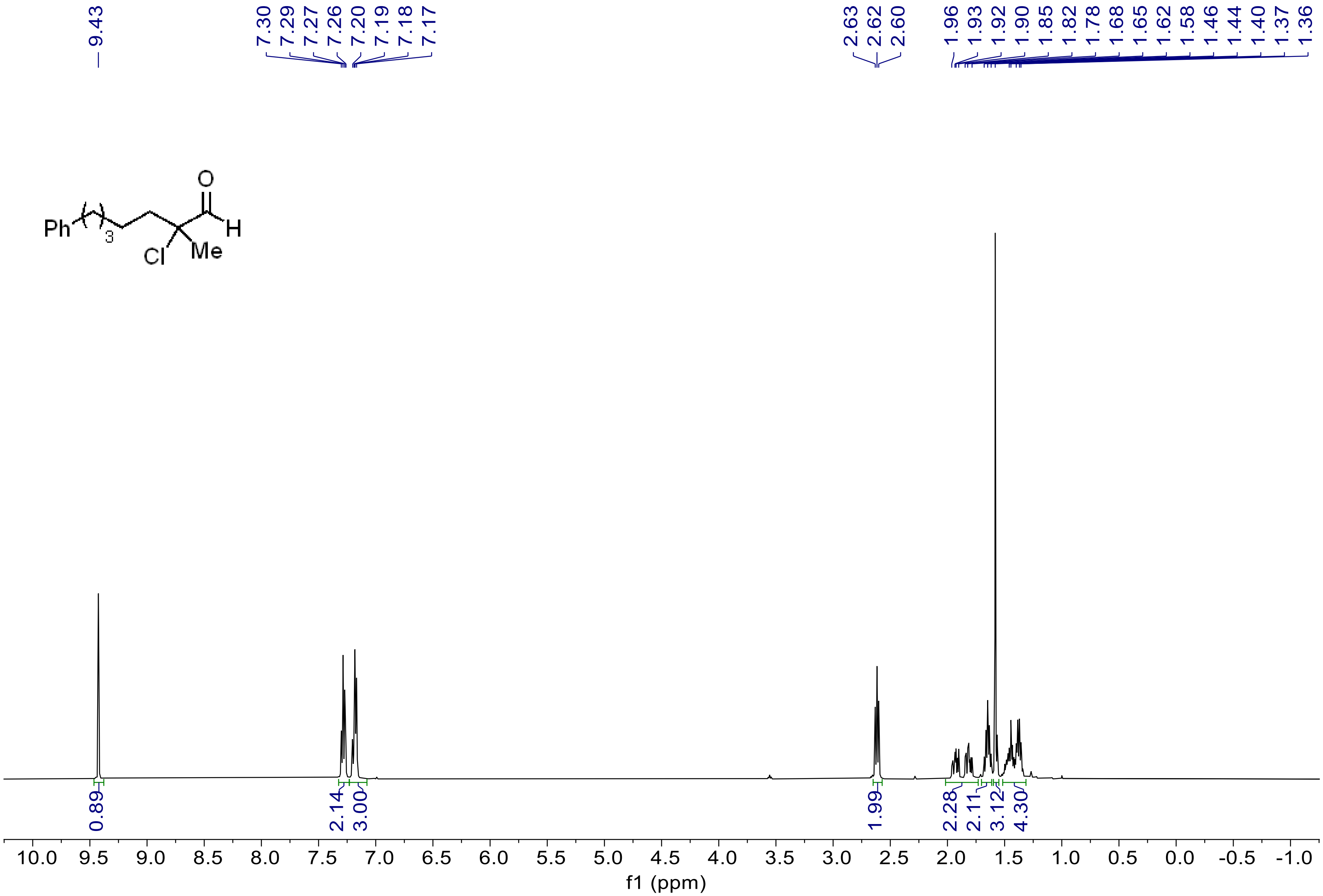

^{13}C NMR of *rac*-2-chloro-2-methyl-7-phenylheptanal 36

$CDCl_3$, 23 °C

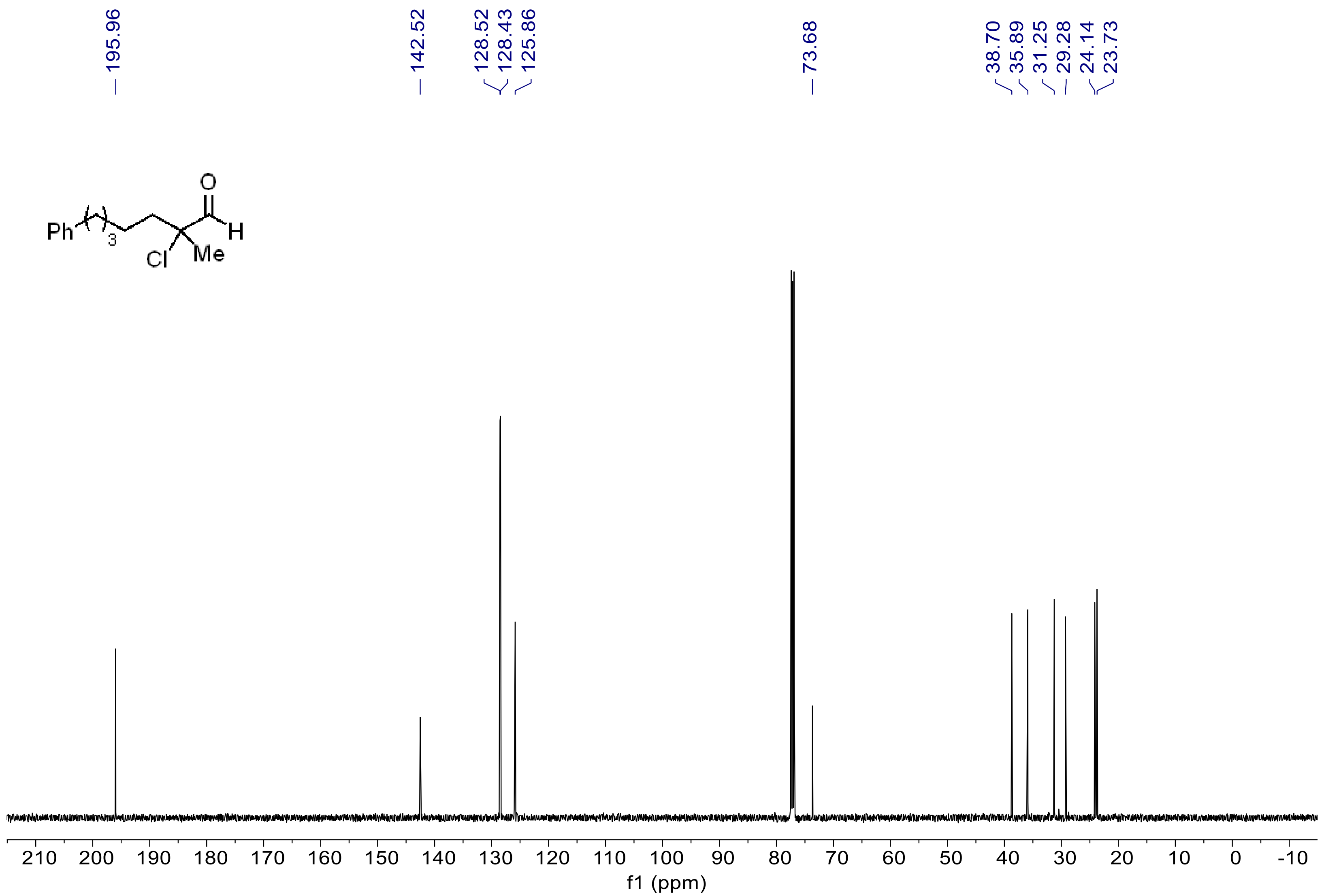

^{1}H NMR of *rac*-methyl 2-chloro-2-methyl-7-phenylheptanoate 37

CDCl$_3$, 23 °C

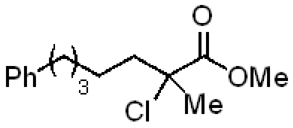

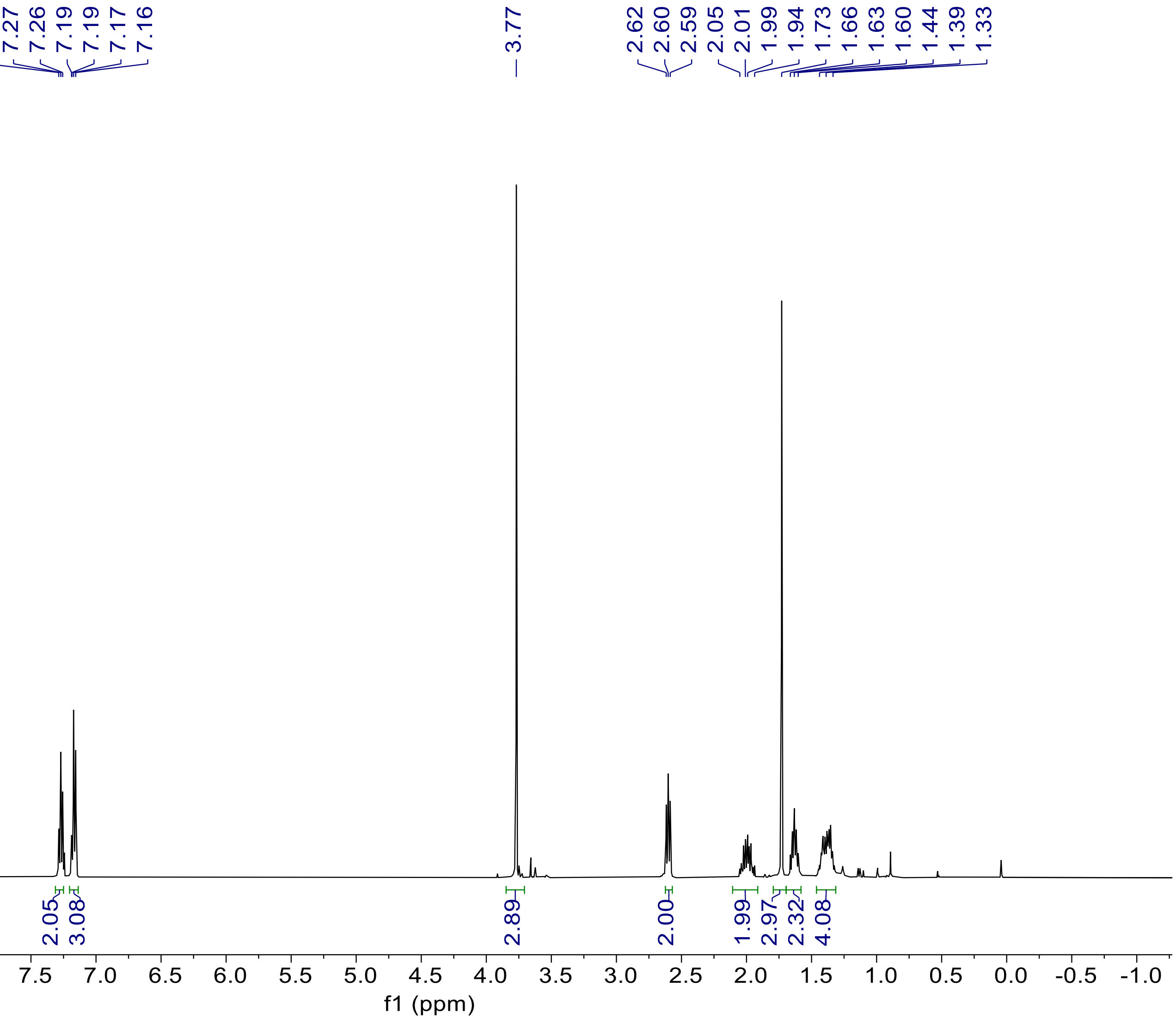

^{13}C NMR of *rac*-methyl 2-chloro-2-methyl-7-phenylheptanoate 37

$CDCl_3$, 23 °C

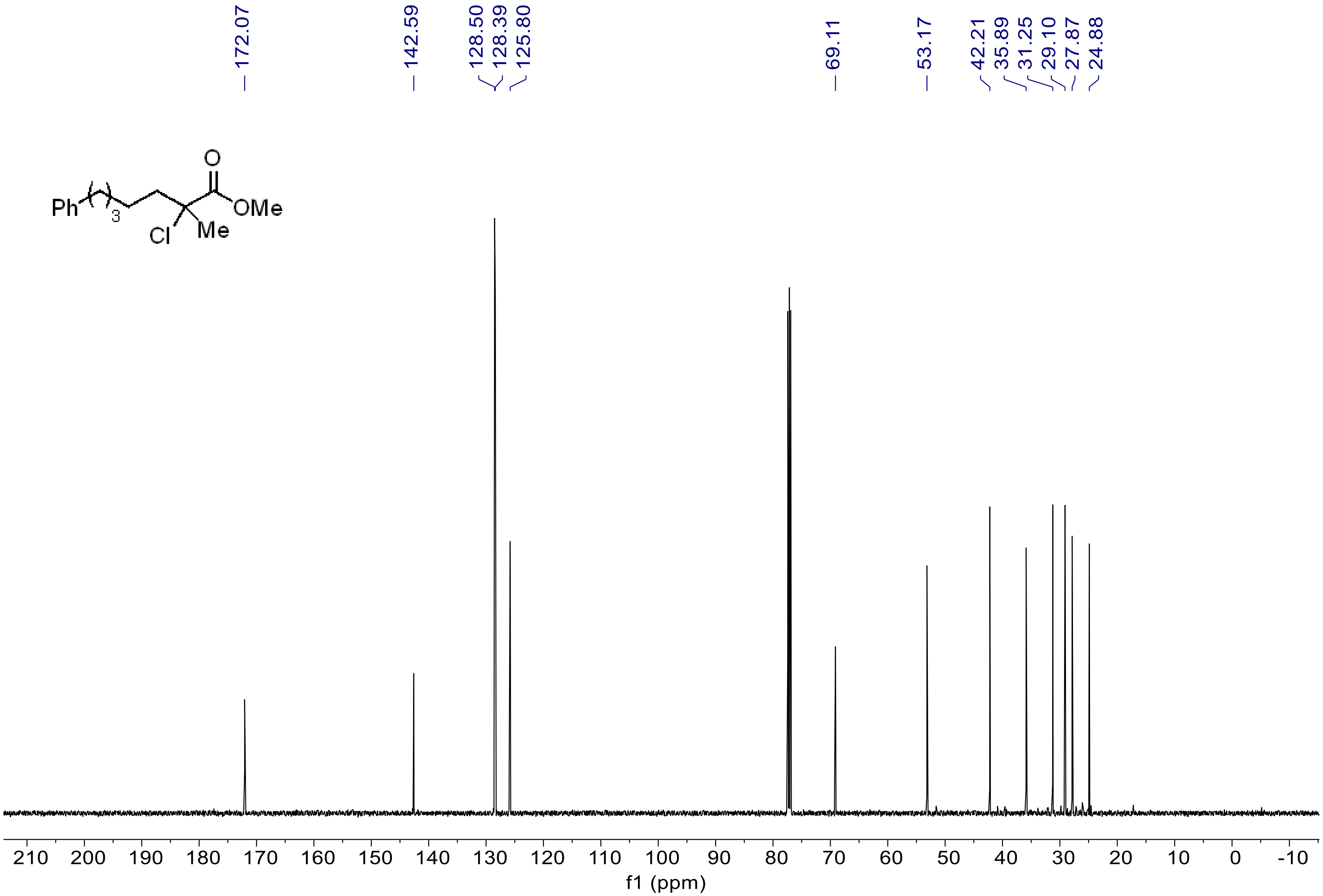

^{1}H NMR of *rac*-2-(2-chloro-7-phenylheptanamido)-2-methylpropane-1-sulfonic acid 38

DMSO, 23 °C

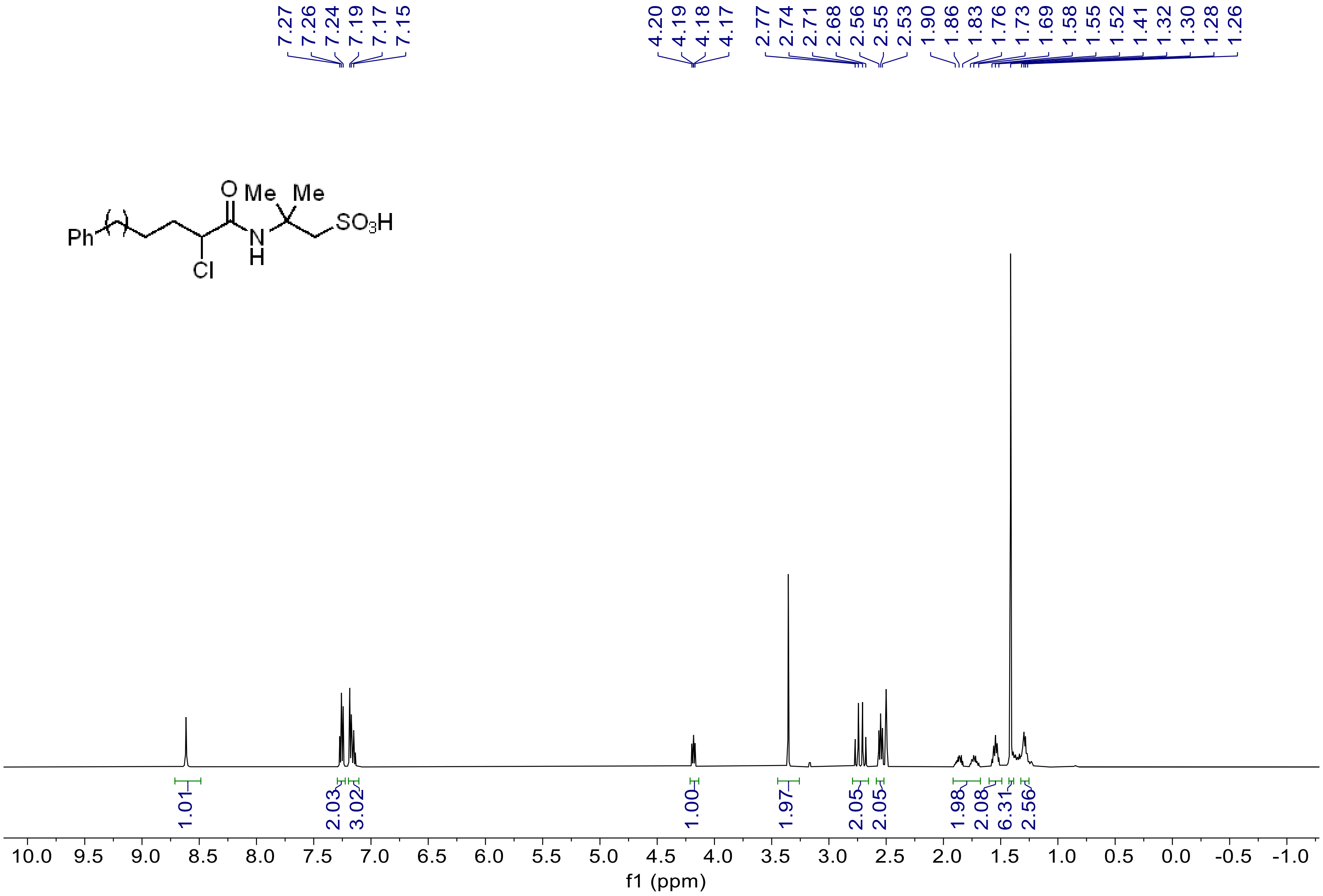

^{13}C NMR of *rac*-2-(2-chloro-7-phenylheptanamido)-2-methylpropane-1-sulfonic acid 38

DMSO, 23 °C

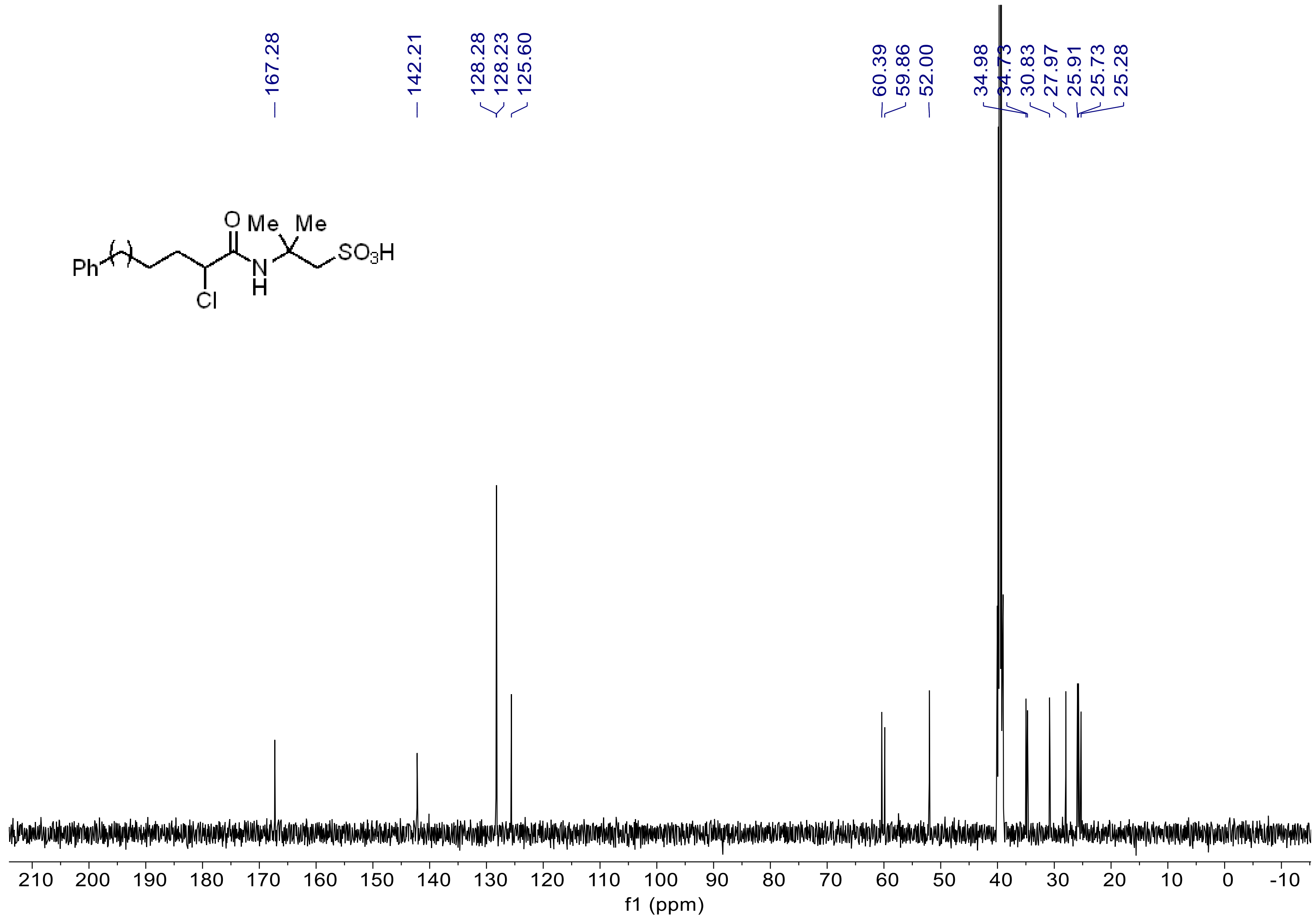

^{1}H NMR of bicyclopentane-derived tripeptide α-chloronitrile 39

$CDCl_3$, 23 °C

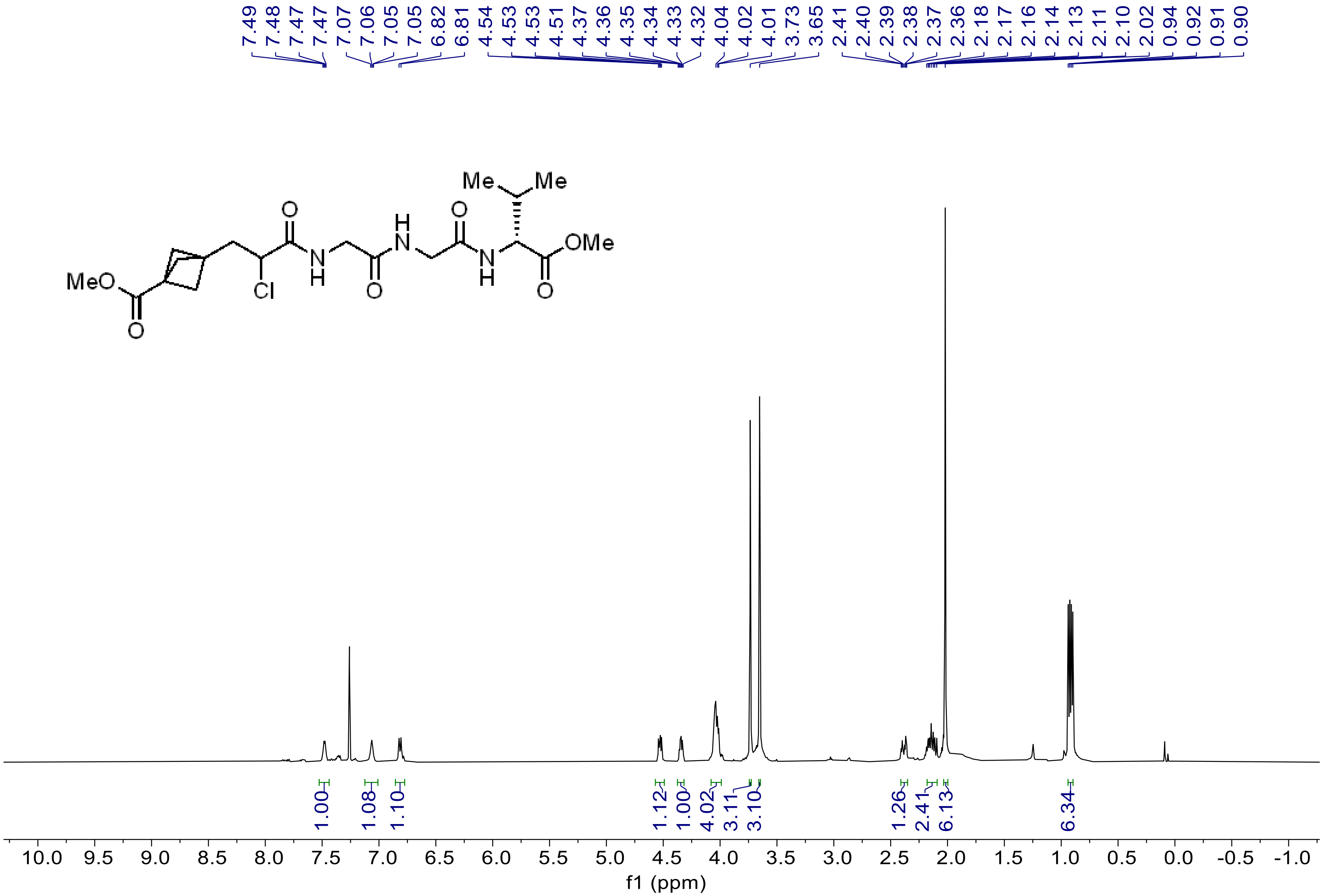

^{13}C NMR of bicyclopentane-derived tripeptide α-chloronitrile 39

$CDCl_3$, 23 °C

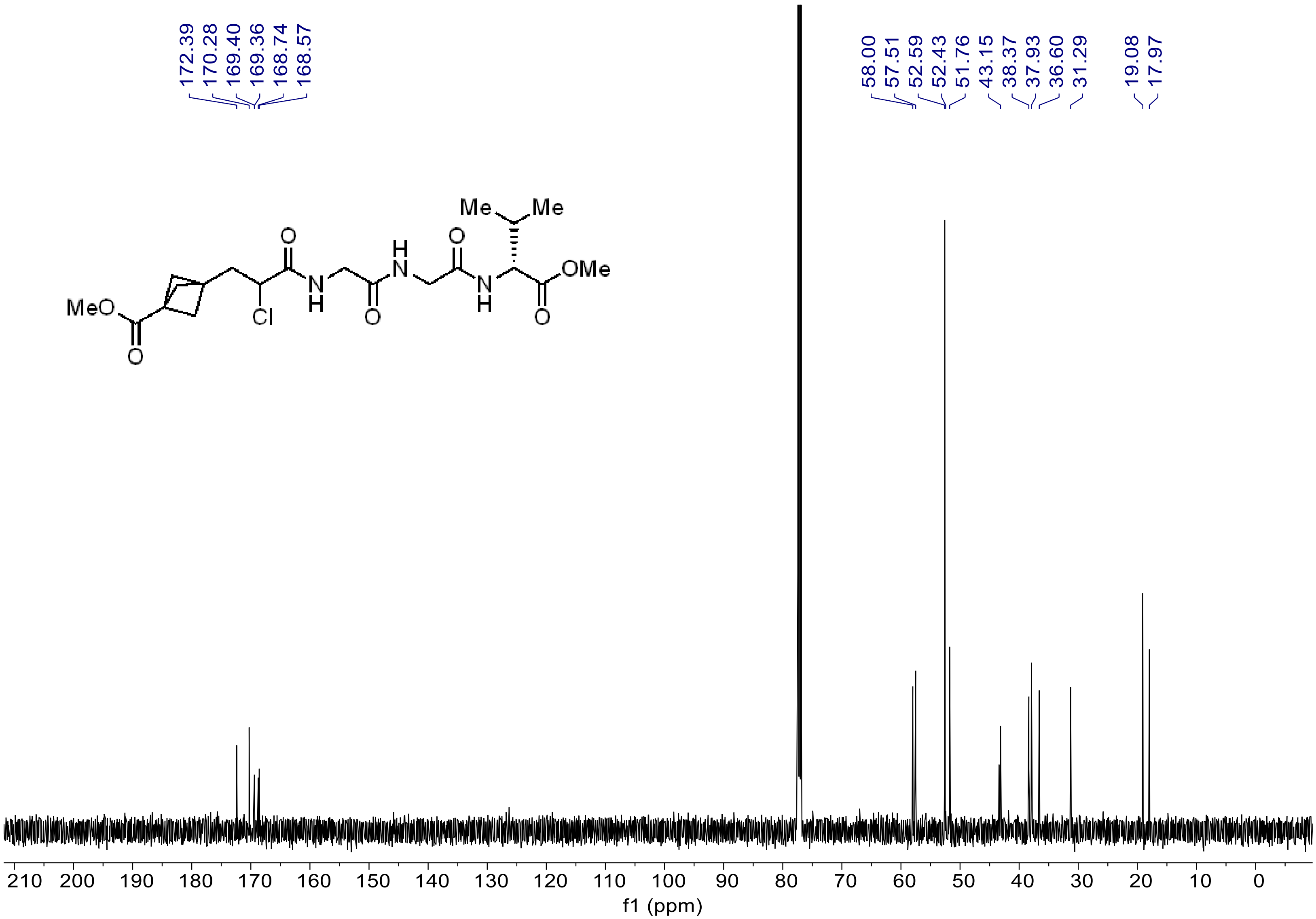

^{1}H NMR of 5-phenylpentanoyl-derived α-chloro amide 41

$CDCl_3$, 23 °C

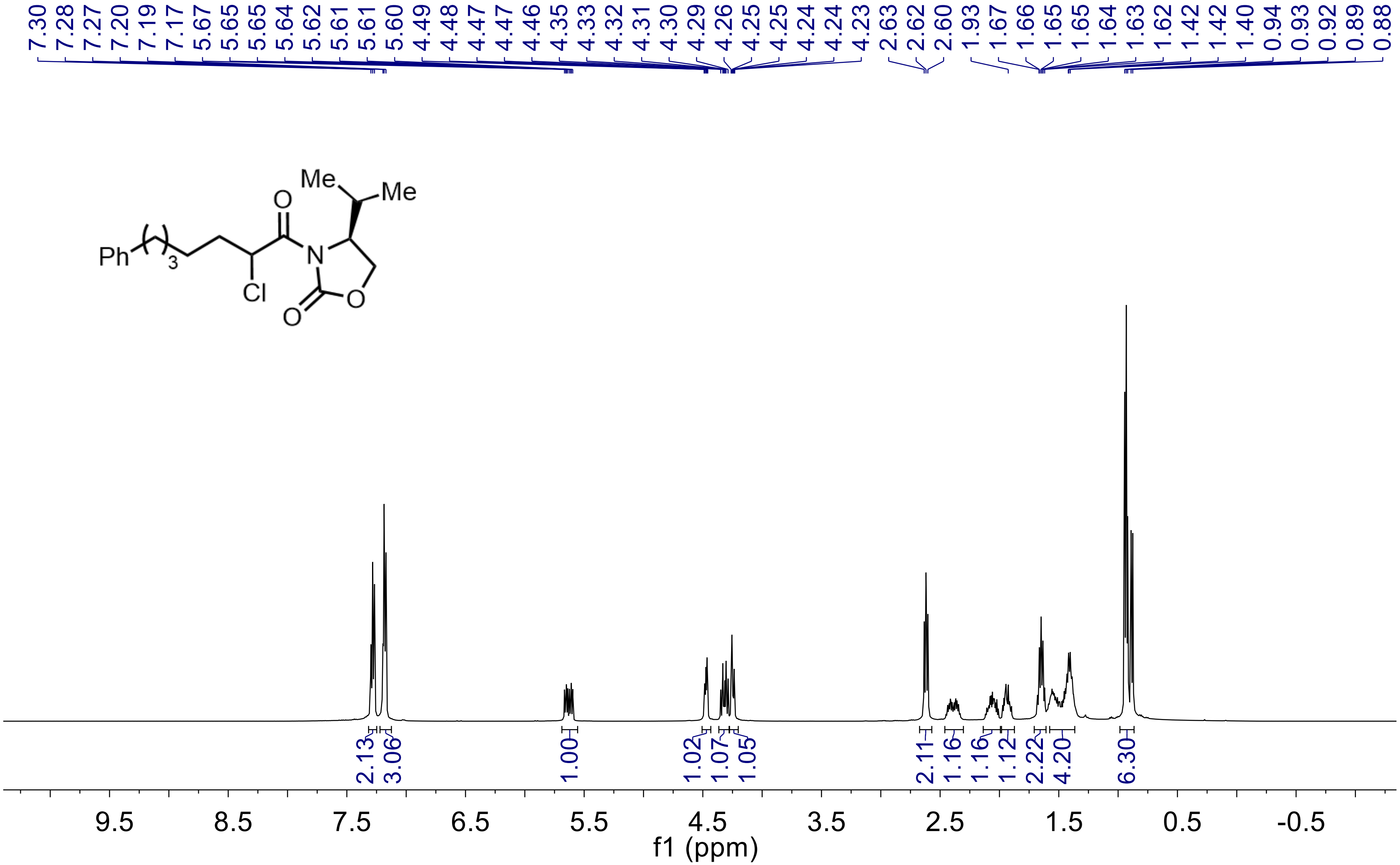

^{13}C NMR of 5-phenylpentanoyl-derived α-chloro amide 41

$CDCl_3$, 23 °C

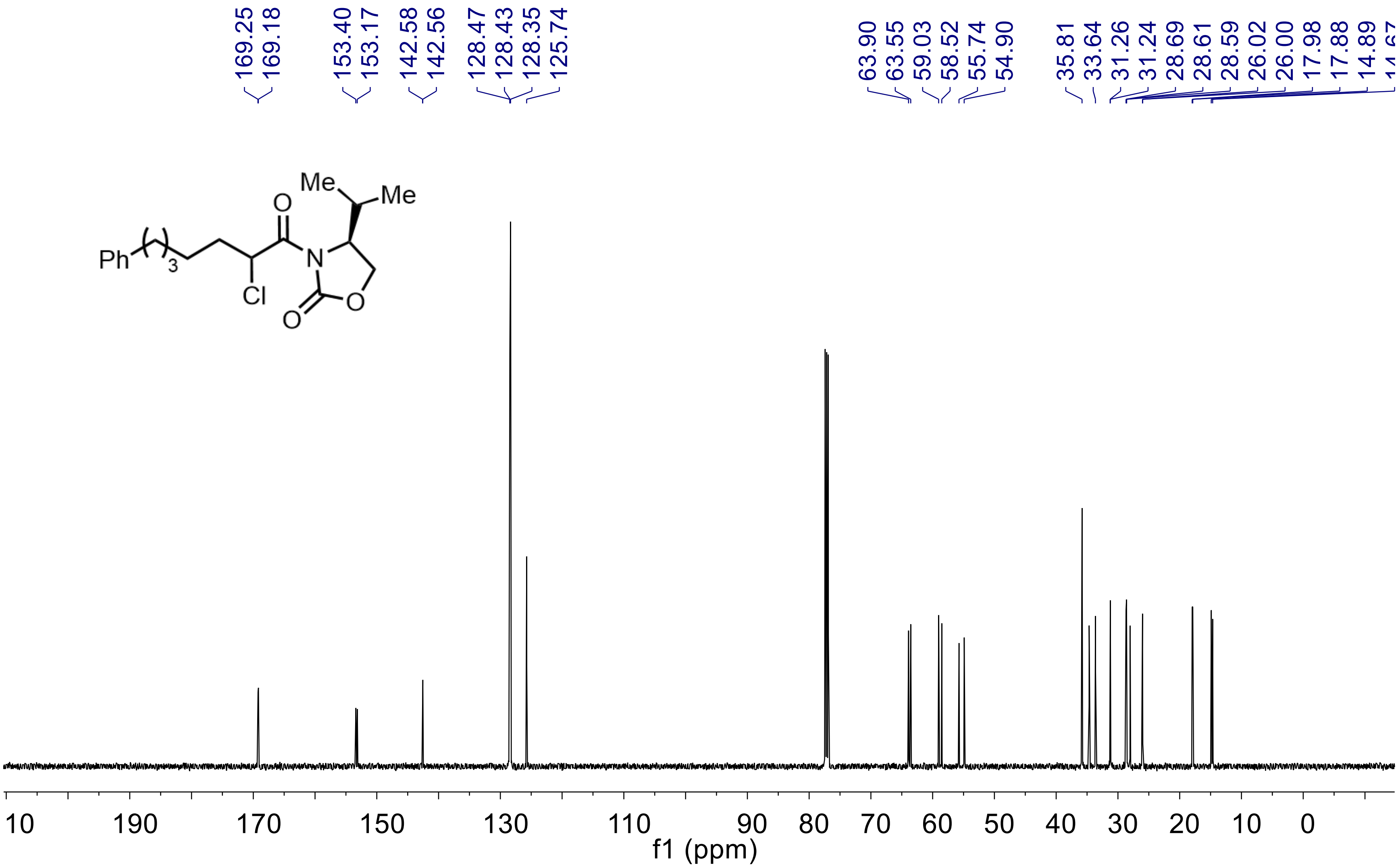

^{1}H NMR of 5-phenylpentanoyl-derived α-amide 42

$CDCl_3$, 23 °C

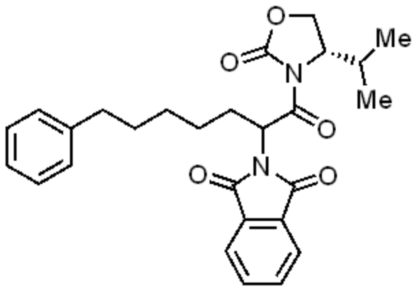

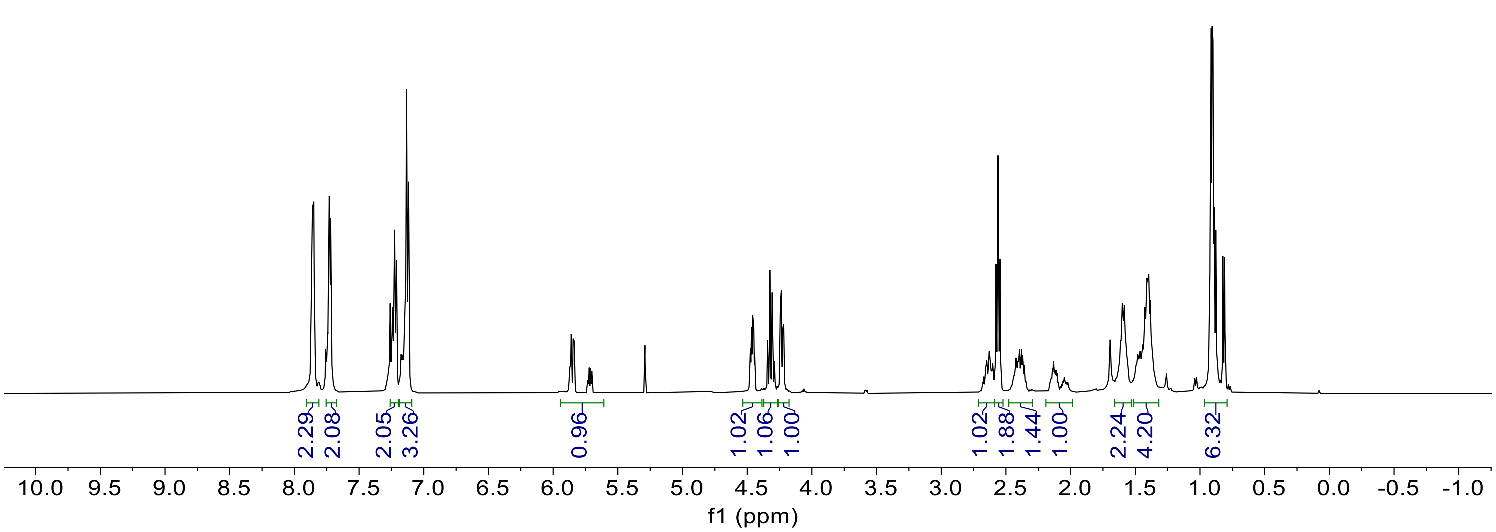

^{13}C NMR of 5-phenylpentanoyl-derived α-amide 42

$CDCl_3$, 23 °C

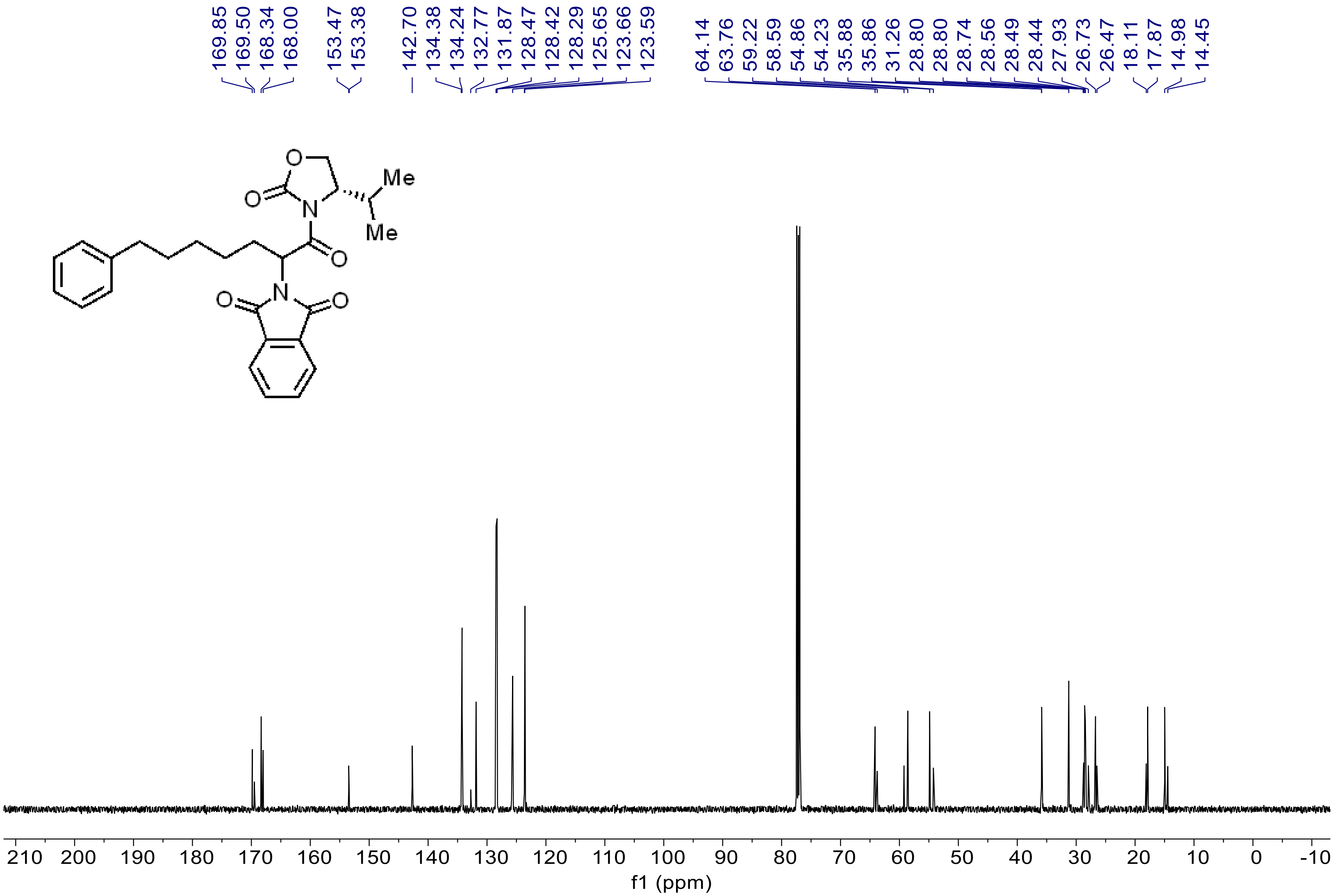

^{1}H NMR of 5-phenylpentanoyl-derived α-morpholine amide 43

$CDCl_3$, 23 °C

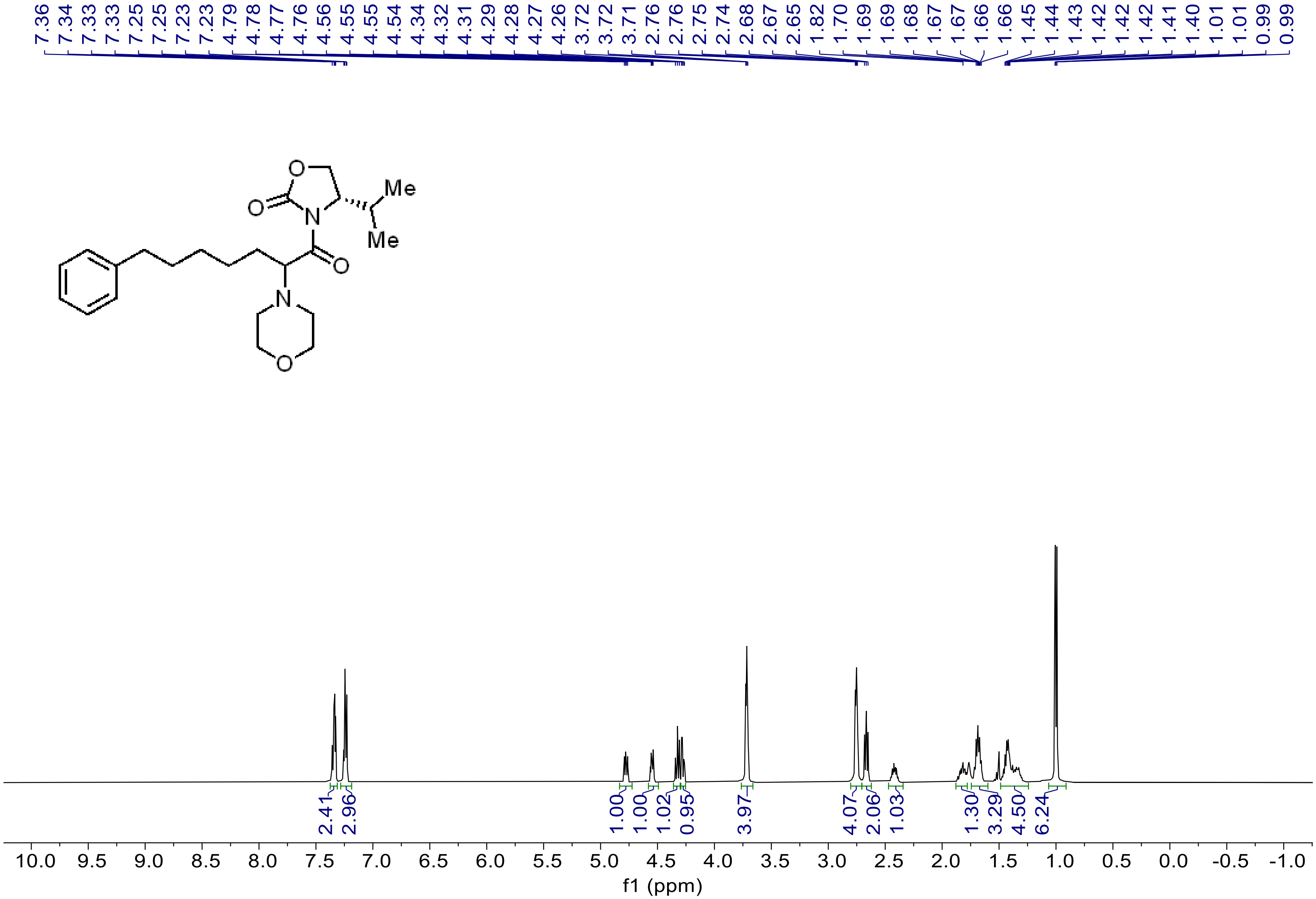

^{13}C NMR of 5-phenylpentanoyl-derived α-morpholine amide 43

$CDCl_3$, 23 °C

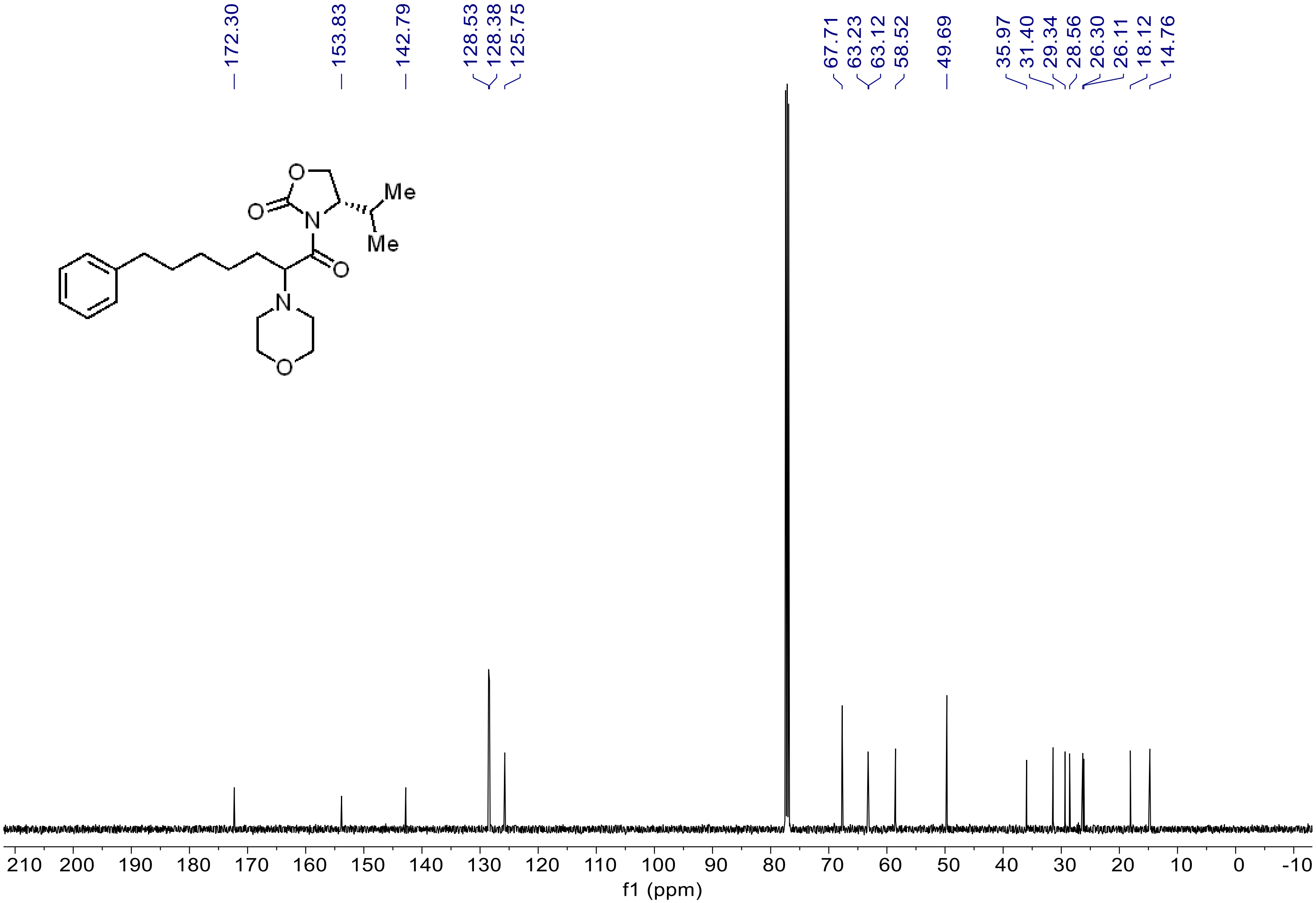

^{1}H NMR of 5-phenylpentanoyl-derived α-phenylester amide 44

CDCl$_3$, 23 °C, Isomer 1

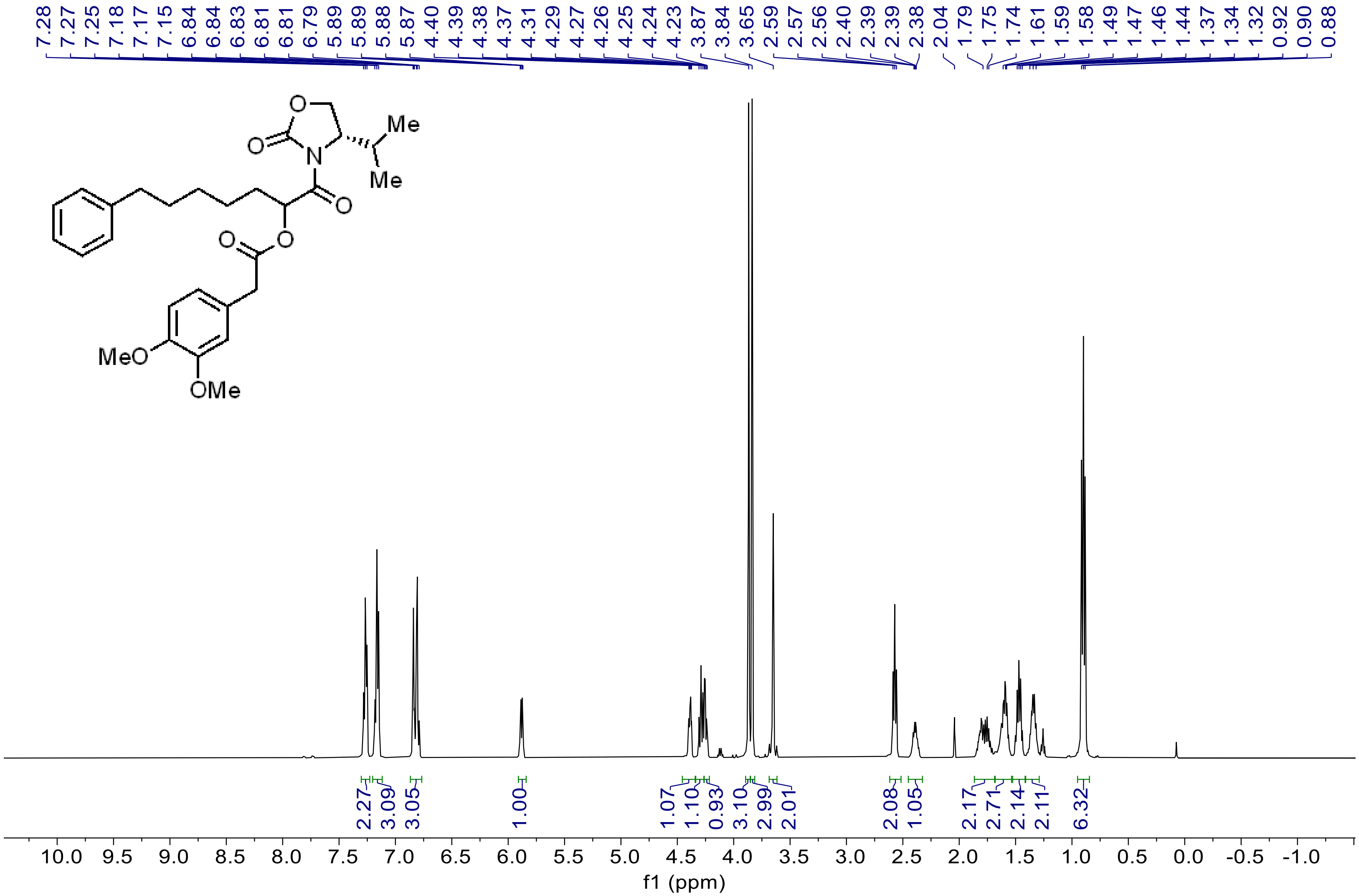

^{13}C NMR of 5-phenylpentanoyl-derived α-phenylester amide 44

$CDCl_3$, 23 °C, Isomer 1

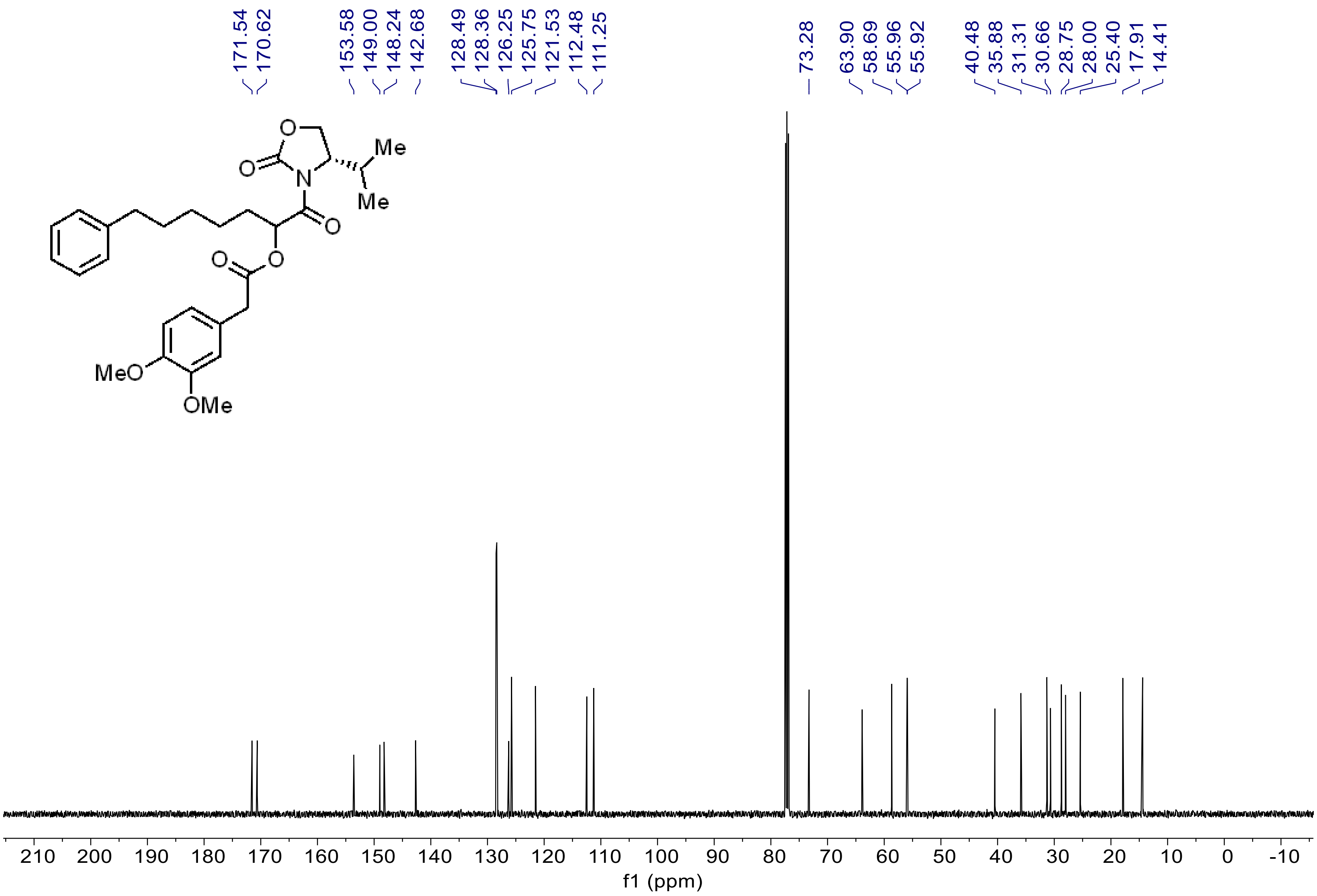

^{1}H NMR of 5-phenylpentanoyl-derived α-phenylester amide 44’

$CDCl_3$, 23 °C, Isomer 2

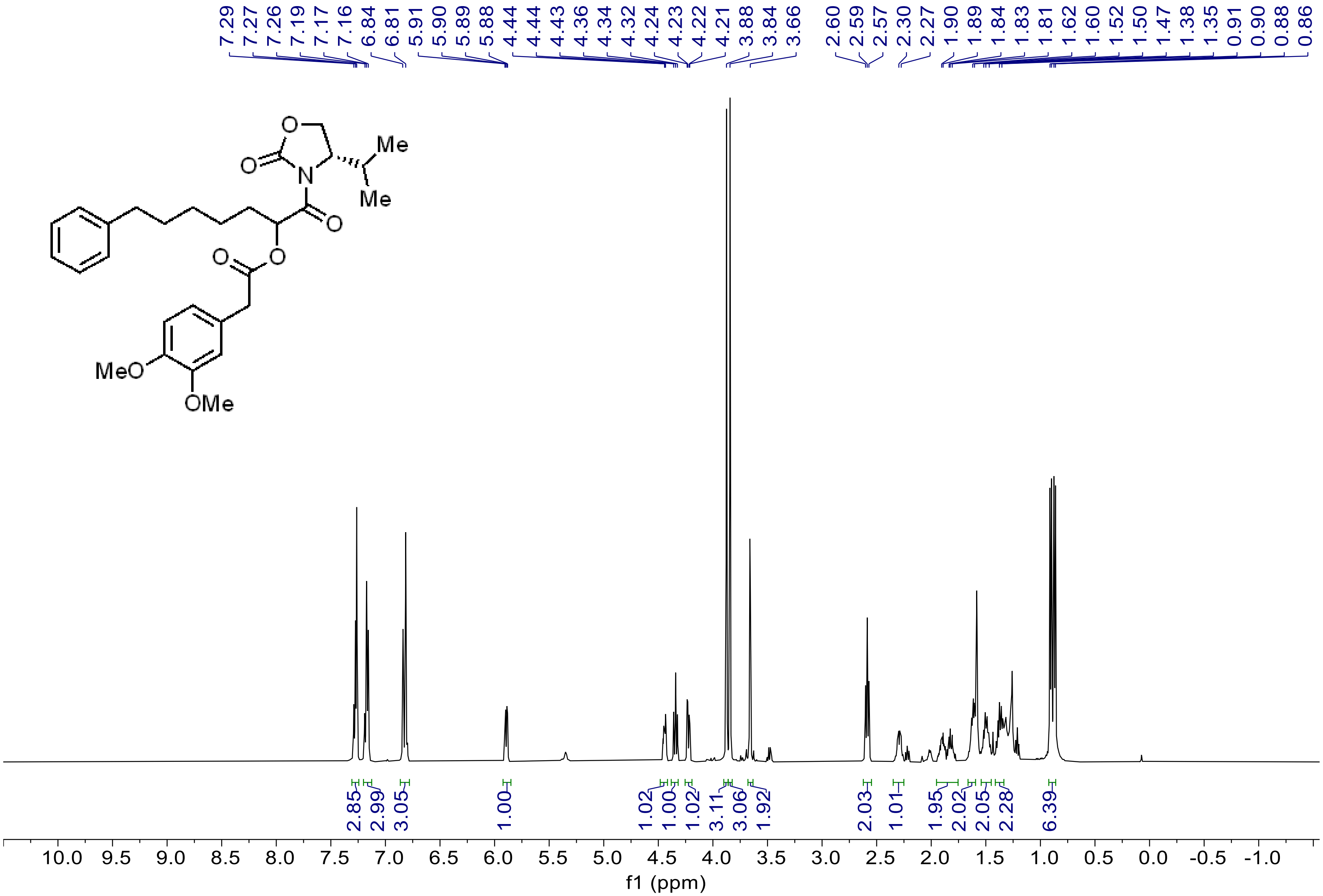

^{13}C NMR of 5-phenylpentanoyl-derived α-phenylester amide 44’

CDCl$_3$, 23 °C, Isomer 2

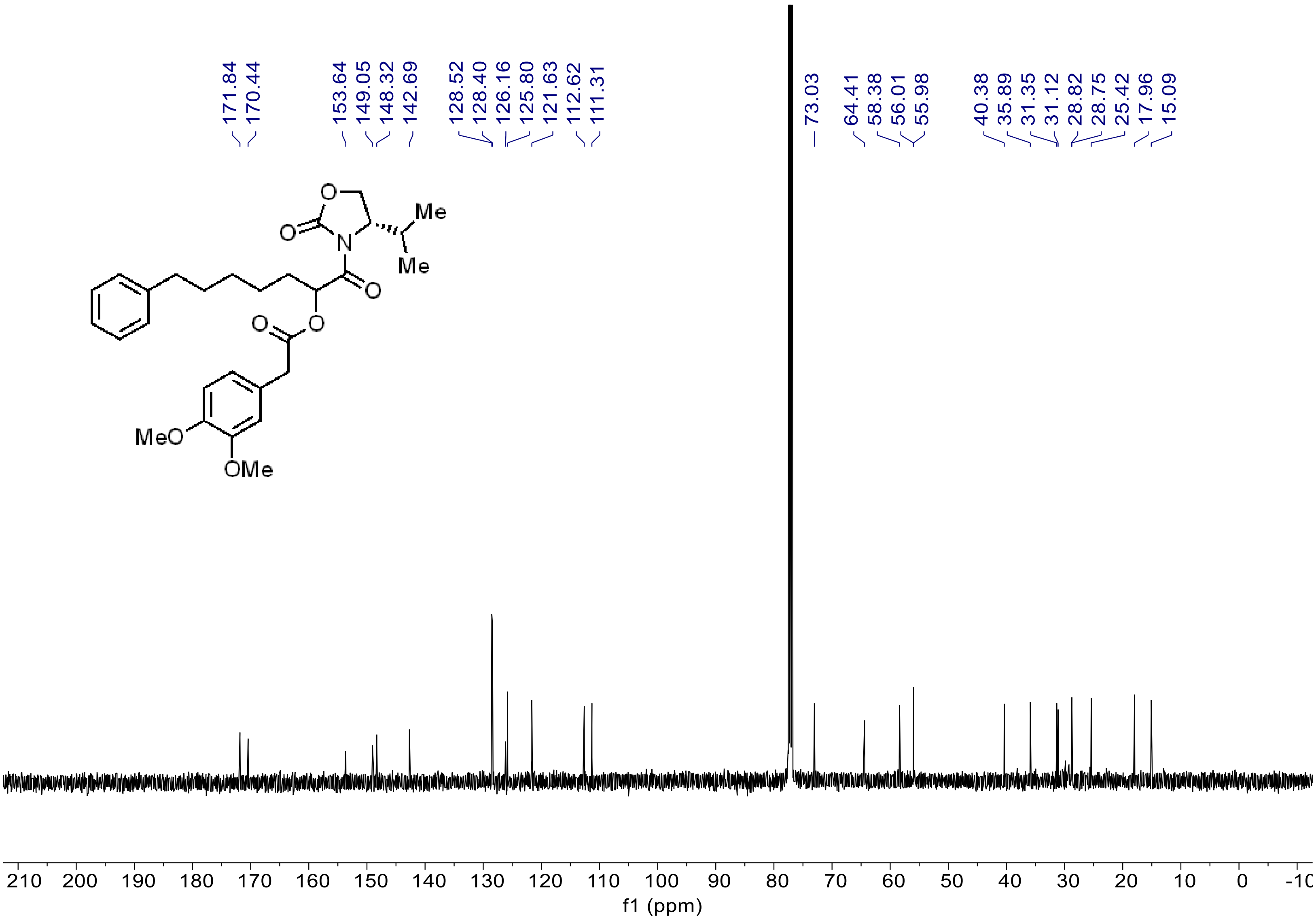

^{1}H NMR of 5-phenylpentanoyl-derived α-oxo amide 45

CDCl$_3$, 23 °C

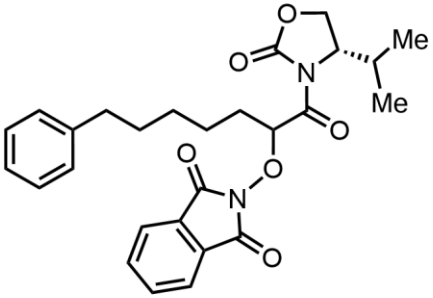

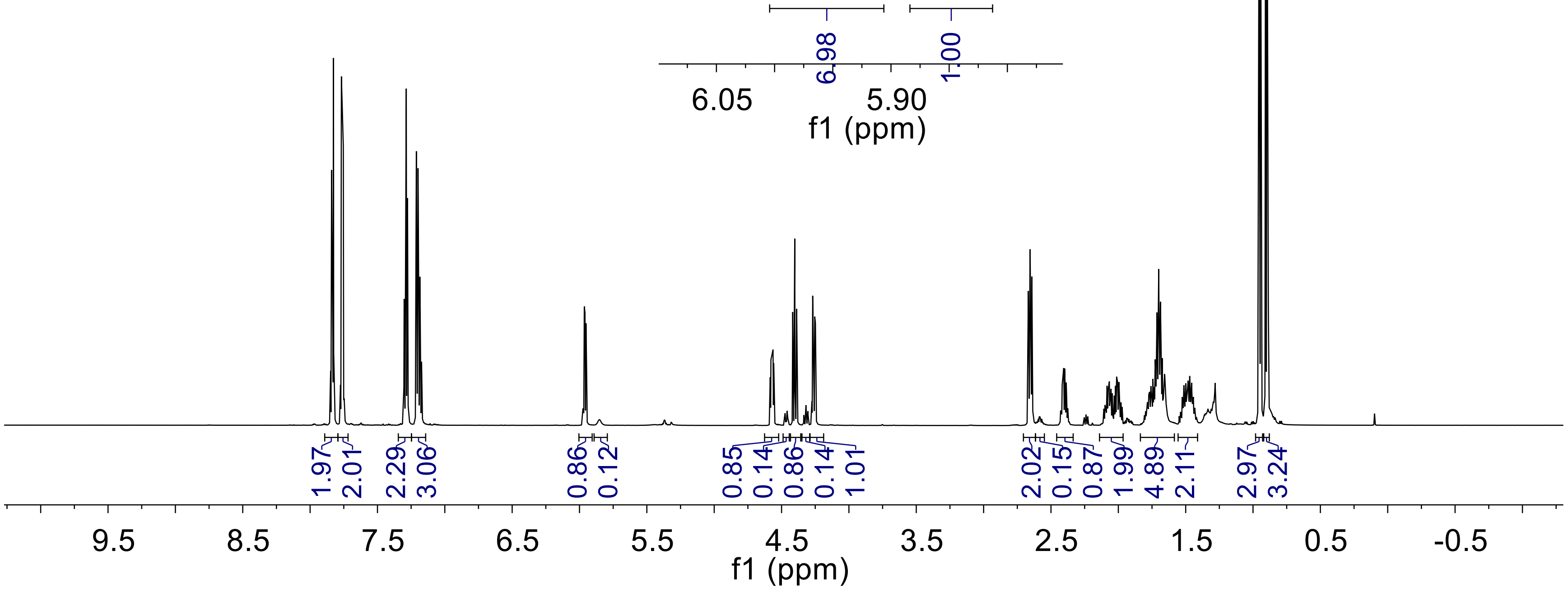

^{13}C NMR of 5-phenylpentanoyl-derived α-oxo amide 45

$CDCl_3$, 23 °C

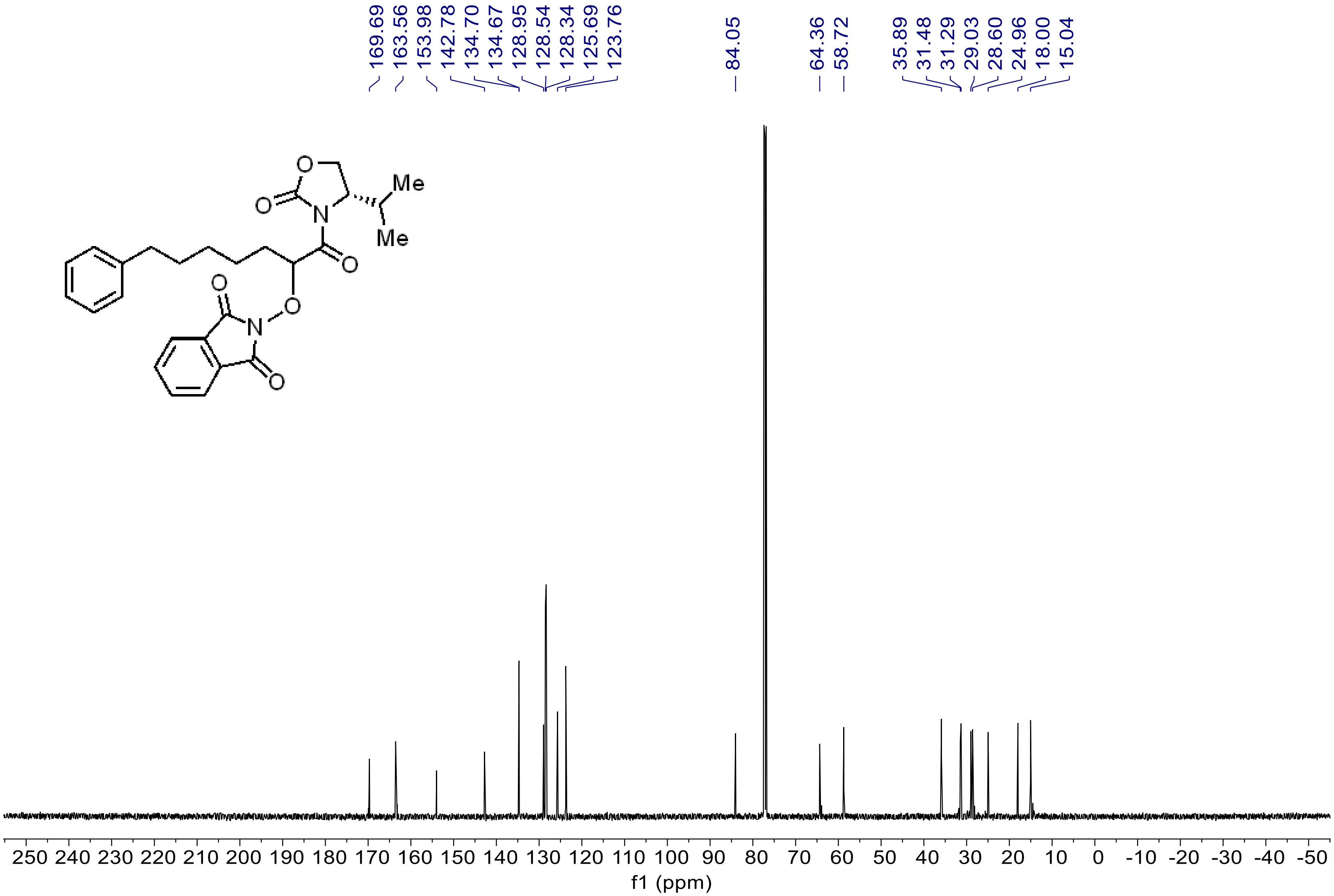

^{1}H NMR of 5-phenylpentanoyl-derived α- phenylthio amide 46

CDCl$_3$, 23 °C

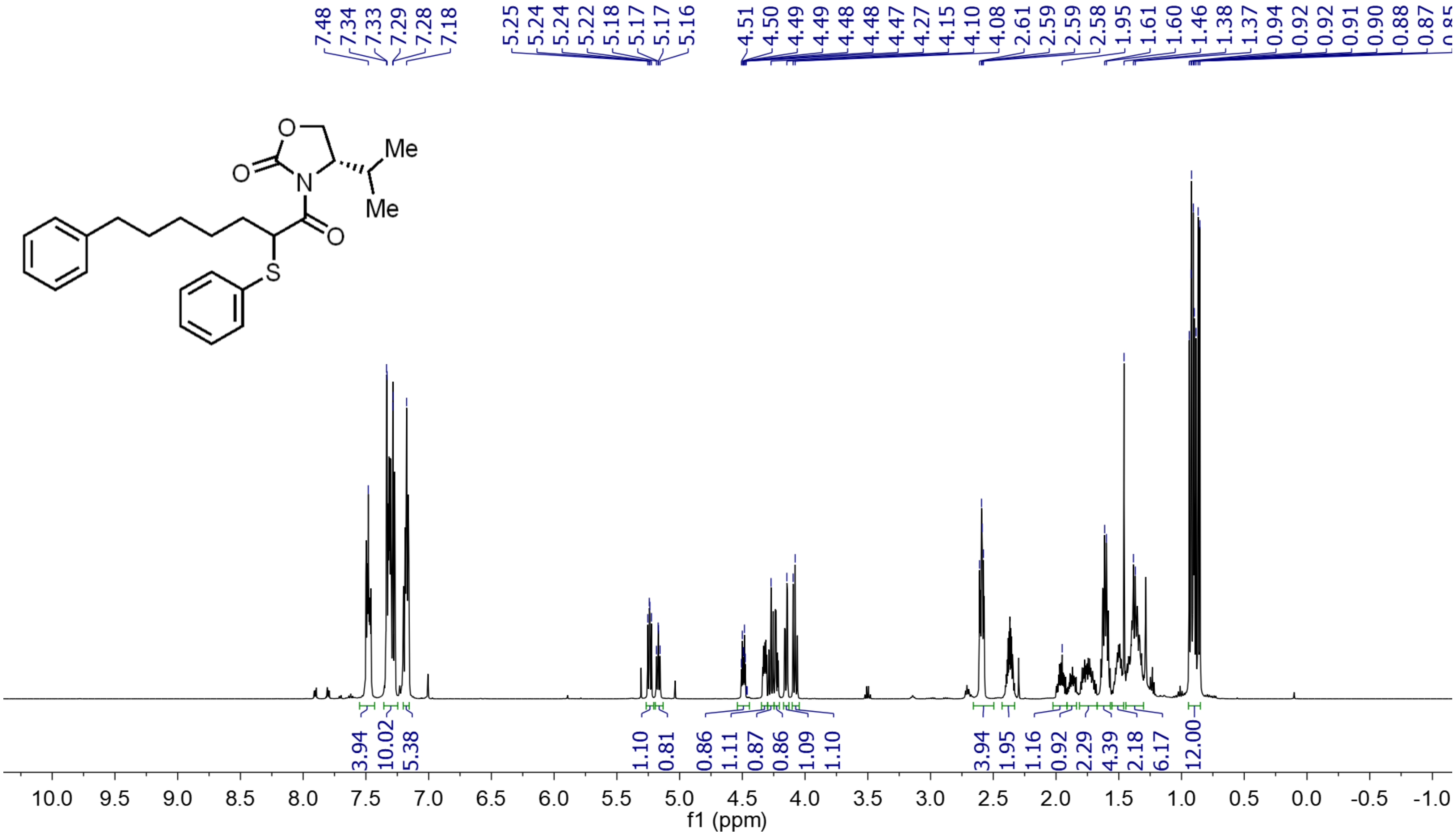

^{13}C NMR of 5-phenylpentanoyl-derived α- phenylthio amide 46

CDCl$_3$, 23 °C

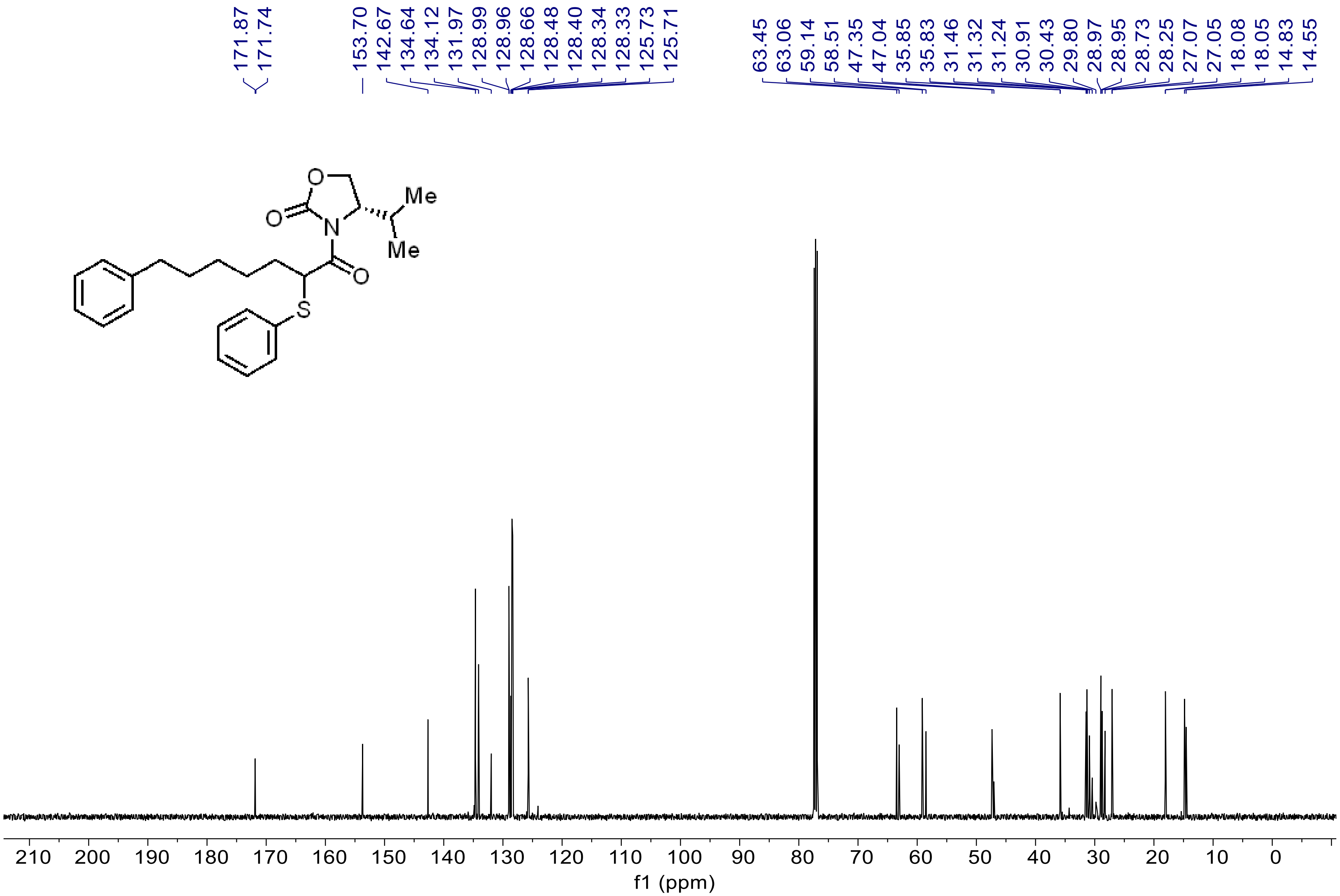

REFERENCES

[1] R. Yonenuma, A. Ishizuki, K. Nakabayashi, H. Mori, *J Polym Sci A Polym Chem* **2019**, *57*, DOI 10.1002/pola.29531.

[2] D. A. Evans, K. T. Chapman, J. Bisaha, *J Am Chem Soc* **1988**, *110*, 1238–1256.

[3] G. Hattori, K. Sakata, H. Matsuzawa, Y. Tanabe, Y. Miyake, Y. Nishibayashi, *J Am Chem Soc* **2010**, *132*, 10592–10608.

[4] I. Guerrero, E. Y. K. Tan, Y. Liu, L. J. Edwards, S. Chiba, *Synthesis (Stuttg)* **2024**, *56*, 3261–3276.

[5] V. C. Polites, S. O. Badir, S. Keess, A. Jolit, G. A. Molander, *Org Lett* **2021**, *23*, 4828–4833.
